# *Fusobacterium nucleatum* is associated with inflammation and poor survival in early-stage HPV-negative tongue cancer

**DOI:** 10.1093/narcan/zcac006

**Published:** 2022-03-04

**Authors:** Sanket Desai, Bhasker Dharavath, Sujith Manavalan, Aishwarya Rane, Archana Kumari Redhu, Roma Sunder, Ashwin Butle, Rohit Mishra, Asim Joshi, Trupti Togar, Shruti Apte, Pratyusha Bala, Pratik Chandrani, Supriya Chopra, Murali Dharan Bashyam, Anirban Banerjee, Kumar Prabhash, Sudhir Nair, Amit Dutt

**Affiliations:** Integrated Cancer Genomics Laboratory, Advanced Centre for Treatment, Research, and Education in Cancer, Kharghar, Navi Mumbai 410210, Maharashtra, India; Homi Bhabha National Institute, Training School Complex, Anushakti Nagar, Mumbai 400094, Maharashtra, India; Integrated Cancer Genomics Laboratory, Advanced Centre for Treatment, Research, and Education in Cancer, Kharghar, Navi Mumbai 410210, Maharashtra, India; Homi Bhabha National Institute, Training School Complex, Anushakti Nagar, Mumbai 400094, Maharashtra, India; Integrated Cancer Genomics Laboratory, Advanced Centre for Treatment, Research, and Education in Cancer, Kharghar, Navi Mumbai 410210, Maharashtra, India; Integrated Cancer Genomics Laboratory, Advanced Centre for Treatment, Research, and Education in Cancer, Kharghar, Navi Mumbai 410210, Maharashtra, India; Integrated Cancer Genomics Laboratory, Advanced Centre for Treatment, Research, and Education in Cancer, Kharghar, Navi Mumbai 410210, Maharashtra, India; Integrated Cancer Genomics Laboratory, Advanced Centre for Treatment, Research, and Education in Cancer, Kharghar, Navi Mumbai 410210, Maharashtra, India; Integrated Cancer Genomics Laboratory, Advanced Centre for Treatment, Research, and Education in Cancer, Kharghar, Navi Mumbai 410210, Maharashtra, India; Integrated Cancer Genomics Laboratory, Advanced Centre for Treatment, Research, and Education in Cancer, Kharghar, Navi Mumbai 410210, Maharashtra, India; Integrated Cancer Genomics Laboratory, Advanced Centre for Treatment, Research, and Education in Cancer, Kharghar, Navi Mumbai 410210, Maharashtra, India; Homi Bhabha National Institute, Training School Complex, Anushakti Nagar, Mumbai 400094, Maharashtra, India; Integrated Cancer Genomics Laboratory, Advanced Centre for Treatment, Research, and Education in Cancer, Kharghar, Navi Mumbai 410210, Maharashtra, India; Homi Bhabha National Institute, Training School Complex, Anushakti Nagar, Mumbai 400094, Maharashtra, India; Department of Biosciences and Bioengineering, Indian Institute of Technology Bombay, Mumbai 400076, Maharashtra, India; Laboratory of Molecular Oncology, Centre for DNA Fingerprinting and Diagnostics, Hyderabad500039, Telangana, India; Integrated Cancer Genomics Laboratory, Advanced Centre for Treatment, Research, and Education in Cancer, Kharghar, Navi Mumbai 410210, Maharashtra, India; Homi Bhabha National Institute, Training School Complex, Anushakti Nagar, Mumbai 400094, Maharashtra, India; Homi Bhabha National Institute, Training School Complex, Anushakti Nagar, Mumbai 400094, Maharashtra, India; Department of Radiation Oncology, Advanced Centre for Treatment, Research, and Education in Cancer, Kharghar, Navi Mumbai 410210, Maharashtra, India; Laboratory of Molecular Oncology, Centre for DNA Fingerprinting and Diagnostics, Hyderabad500039, Telangana, India; Department of Biosciences and Bioengineering, Indian Institute of Technology Bombay, Mumbai 400076, Maharashtra, India; Department of Medical Oncology, Tata Memorial Centre, Ernest Borges Marg, Parel, Mumbai 400012, Maharashtra, India; Division of Head and Neck Oncology, Department of Surgical Oncology, Tata Memorial Hospital, Tata Memorial Centre, Mumbai 400012, Maharashtra, India; Integrated Cancer Genomics Laboratory, Advanced Centre for Treatment, Research, and Education in Cancer, Kharghar, Navi Mumbai 410210, Maharashtra, India; Homi Bhabha National Institute, Training School Complex, Anushakti Nagar, Mumbai 400094, Maharashtra, India

## Abstract

Persistent pathogen infection is a known cause of malignancy, although with sparse systematic evaluation across tumor types. We present a comprehensive landscape of 1060 infectious pathogens across 239 whole exomes and 1168 transcriptomes of breast, lung, gallbladder, cervical, colorectal, and head and neck tumors. We identify known cancer-associated pathogens consistent with the literature. In addition, we identify a significant prevalence of *Fusobacterium* in head and neck tumors, comparable to colorectal tumors. The *Fusobacterium*-high subgroup of head and neck tumors occurs mutually exclusive to human papillomavirus, and is characterized by overexpression of miRNAs associated with inflammation, elevated innate immune cell fraction and nodal metastases. We validate the association of *Fusobacterium* with the inflammatory markers *IL1B*, *IL6* and *IL8*, miRNAs *hsa-mir-451a*, *hsa-mir-675* and *hsa-mir-486-1*, and *MMP10* in the tongue tumor samples. A higher burden of *Fusobacterium* is also associated with poor survival, nodal metastases and extracapsular spread in tongue tumors defining a distinct subgroup of head and neck cancer.

## Graphical Abstract

**Graphical Abstract:**
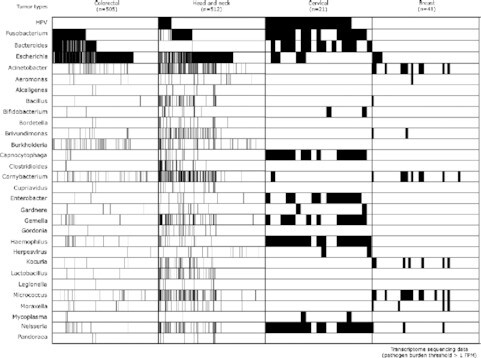
Landscape of microbes associated with human cancers identifies mutual exclusivity of human papillomavirus and *Fusobacterium* in head and neck cancer.

## INTRODUCTION

The Human Microbiome Project has identified 48 microbial habitats in the human body (1). These microbes maintain balanced symbiotic/commensal relationships or a ‘eubiosis’ under normal conditions (2). A shift in the eubiotic balance or a ‘dysbiosis’ can lead to disease. Chronic inflammation, often linked to cancer initiation and progression, is known to result from these dysbiotic events and persistent infections of specific microbes. The infections also elicit host immune responses making the microenvironment tumor-permissive (3). Additionally, genotoxins or processed metabolites from the microbes have also been shown to induce genomic instability and are known to modulate the tumorigenesis (4). Considering the above factors to define possible causality and formulation of models of cancer pathogenesis due to microbes (especially in colon cancer) provide a framework for studying the role of cancer-associated microbes across different tissues (5).

Few well-known associations between microbial infection and cancer are that of *Helicobacter pylori*, which is causally related to 60–90% of all gastric cancer cases (6). Causality between infection and tumorigenesis has been established in various animal model systems for *H. pylori* (7,8). Other reported associations are those of *Salmonella typhi* infection with gallbladder cancer (9,10), *Streptococcus bovis*, enterotoxigenic *Bacteroides fragilis*, genotoxic *Escherichia coli* and *Fusobacterium nucleatum* with colon cancer (11–15), and *Chlamydia pneumonia* with lung cancer (16).

Interestingly, 20% of all human microbial habitats are associated with the head and neck region (17). Multiple studies have investigated the enrichment of distinct microbial species in the head and neck squamous cell carcinomas (HNSCs). They have shown an association of microbial burden with the stage of the disease (18,19). Multiple studies suggest that the association between periodontal bacteria and tumorigenesis is mediated by chronic inflammation. Recently reported systematic meta-analyses highlight role of *F. nucleatum* in head and neck cancer (20). Other inflammatory periodontal bacterial pathogens have also been implicated to be an independent risk factor in HPV (human papillomavirus)-negative HNSCs (21). Currently, minimal information exists regarding the association between such periodontal pathogens and biology, especially in HPV-negative HNSC tumors. Systematic efforts are required to understand the role of specific intratumor microbes and their clinical implications.

Next-generation sequencing (NGS) provides a powerful and unbiased tool for identification of viral and bacterial pathogens from human samples, including tumor microenvironment (22). Microbial composition has been derived from human tissue using various computational approaches, including computational subtraction (23). Building on this computational subtraction approach, we recently developed infectious pathogen detector (IPD) (24), to identify infectious pathogens from heterogeneous NGS datasets. Using IPD, we performed systematic quantification of 1060 infectious pathogens from 239 DNA and 1168 RNA sequencing samples representing six cancer types: breast, cervical, colorectal, lung, gallbladder and HNSC. Along with identifying known tumor-associated microbes, we find enrichment of *F. nucleatum* in the oral tumors, consistent with earlier reports (25). We observed mutual exclusivity of HPV and *Fusobacterium* in HNSCs, with both forming a distinct subclass of tumors. Comparing the gene and miRNA expression data within the HPV-negative HNSC tumors, we identified differentially expressed genes and miRNAs associated with a higher burden of *Fusobacterium*. We further validated the selected inflammation markers and inflammation-linked miRNAs in the in-house tongue tumor patient samples. Correlating with the immune cell signatures, we identify that the *Fusobacterium*-high subgroup of HNSC tumors shows increased innate immunity factors, with pro-tumorigenic potential. Locoregional lymph node metastasis and tumor extension outside lymph node capsule are known poor prognosis factors in head and neck cancer (26). This study defines a distinct subgroup of HPV-negative tongue tumors with higher inflammation, nodal metastases, extracapsular spread and poor prognosis, characterized by potential surrogate miRNA markers.

## MATERIALS AND METHODS

### Patient sample collection and mRNA/miRNA/*F. nucleatum* quantification from tumors

The cervical adenocarcinoma patient samples (*n* = 41) were collected from cervical adenocarcinoma patients at the Tata Memorial Hospital (TMH) and ACTREC biorepository. The study was approved by the ACTREC–Tata Memorial Centre (TMC) institutional review board (IRB) (study protocol #116). From the 41 cervical samples, whole exome sequencing was performed on 17 paired tumor samples and 1 orphan tumor sample (*n* = 35), and whole transcriptome sequencing was performed on 24 tumor and 5 normal samples (*n* = 29).

The retrospectively collected non-small cell lung cancer patient samples (*n* = 52) were obtained from the TMH and ACTREC biorepository. Whole exome sequencing was performed on all the samples (*n* = 52). Sample set and study protocols were approved by the IRB and Ethics Committee (EC) of TMC–ACTREC (study protocol # 900514).

The retrospectively collected oral cancer samples were obtained from the TMH and ACTREC tumor tissue repository, with clinical characteristics as described in Table 1. Fresh frozen primary tumor (*n* = 75) and normal (*n* = 25) oral samples were collected at the TMH and ACTREC, Mumbai, India. The sample set and study protocols were approved by the IRB and EC of TMC–ACTREC (study protocol #88).

**Table 1.** Clinicopathological details of in-house validation set of tongue cancer patients

	Number of patients (*n* = 74)	Percentage
**Age**		
<40 years	21	28.38
40–60 years	23	31.08
>60 years	8	10.81
NA	22	29.73
**Gender**		
Male	46	62.16
Female	6	8.11
NA	34	45.95
**Habit**		
Alcohol history	15	20.27
Tobacco/smoking history	37	50.00
**Clinical stage**		
Early stage (T1/T2)	74	100.00
**Pathological stage**		
T1	12	16.22
T2	34	45.95
T3/T4	15	20.27
NA	13	17.57
**Node stage**		
N0	31	41.89
N1	15	20.27
N2	15	20.27
NA	13	17.57
**Survival**		
≤24 months	19	25.68
>24 months	33	44.59
NA	22	29.73
**Recurrence**		
Yes	14	18.92
No	38	51.35
NA	22	29.73
**Distance metastasis**		
Yes	2	2.70
No	50	67.57
NA	22	29.73

The anatomical sites of all the oral cancer samples collected were of tongue origin and were identified to be early-stage tumors by clinical assessment. Of the total 75 oral tumor samples, 1 sample was ignored due to low RNA concentration. The set of 74 tumor and 25 normal samples was used as a validation set to quantify *F. nucleatum*, human genes and miRNAs associated with the pathogen. The primer sequences for each microRNA, genes and *Fusobacterium* validation used in the study are given in Supplementary Table S1.

### Whole exome and transcriptome sequencing of the patient samples

For exome sequencing of 35 cervical samples, two different capture kits were employed. In brief, SureSelect XT Target Enrichment Kit (Agilent Technologies, Santa Clara, CA, USA) capturing 50 Mb of the genome was used for 13 samples. Genomic DNA (200 ng) was sheared using covaris to generate 150–500 bp fragment size. For remaining 22 samples, SureSelect Human All Exon Kit, v5 (Agilent Technologies, Santa Clara, CA, USA) was used and library preparation was done using 1 μg of genomic DNA. The fragment ends were repaired followed by adenylation at 3′ end and purified using AMPure XP beads. The fragments were ligated to the adaptor and amplified by PCR. The generated libraries were then hybridized with SureSelect Target Enrichment System Kit and hybrids were separated using streptavidin-coated magnetic beads. Then, the samples were PCR amplified using indexing primers and purified. The quality of prepared libraries was assessed on a BioAnalyzer, quantified by qPCR and then loaded on the Illumina flow cell to generate clusters. Libraries were sequenced for 301 cycles on the NextSeq 500 Illumina platform to generate 150 bp paired-end reads to obtain 100× coverage. The prepared libraries were loaded on the Illumina flow cell and sequenced for 201 cycles on the HiSeq 2500 platform to generate 100 bp paired-end reads to obtain 100× sequencing depth. For the non-small cell lung cancer patient samples (*n* = 52), a minimum of 100 ng input DNA was used. SureSelect Human All Exon V6 (capture size 60 Mb) was used for 20 samples [described here (27)] and TrueSeq DNA Exome Kit (capture size 45 Mb) was used for 32 samples. Exome sequencing was performed on the Illumina platform according to the standard protocol using 150 bp paired-end reads to obtain an average coverage of 100× across all the samples. The exome sequencing method used for the 27 paired colorectal samples used in the study is described in (28).

For transcriptome sequencing of 29 cervical samples, library preparation was performed using SENSE Total RNA-Seq Library Prep Kit (Lexogen Inc.). The ribosomal RNA (rRNA) was depleted from total RNA using RiboCop rRNA Depletion Kit V1.2 (Lexogen Inc.) as per the manufacturer’s protocol. Libraries were sequenced in the Illumina HiSeq 4000 platform to yield 100 bp paired-end reads, with total output of >60 million reads per sample. For colorectal transcriptome samples, only tumors harboring >70% of tumor epithelial cells were used for RNA isolation. RNA was isolated from colorectal samples using Trizol reagent (Invitrogen Inc., Carlsbad, CA, USA) as per the manufacturer’s protocol. Five micrograms of total RNA was used to prepare the RNA sequencing library using the TruSeq RNA Sample Prep Kits (Illumina). Removal of abundant rRNA from a total RNA sample was performed with the Epicentre Ribo-Zero family of rRNA depletion kits and libraries were prepared as per the manufacturer’s protocol (Illumina). Generated libraries were sequenced on HiSeq 2000/2500 to obtain 2 × 100 bp paired-end reads to obtain 140 million clean reads.

### Microbiome quantification from whole exome and transcriptome sequencing data

We developed IPD, a computational tool, to quantify pathogens from the whole exome and transcriptome samples, as described earlier (24). IPD performs data pre-processing by filtering the low-quality and ambiguous alignment reads and gives normalized quantification of fragments aligned to pathogen genome, by scaling it to library yield [fragments per million (FPM)] and normalizing to genome length [fragments per kilobase per million (FPKM)]. These normalizations make the pathogen quantification comparable across different samples and across library types (single- and paired-end data). Samples having an FPM of >0 were considered as positive for the bacterial pathogen. Specifically for viral pathogens, a stringent threshold of 1 FPM was used for positivity, as described earlier (29). In total, 239 exomes and 110 transcriptomes of in-house samples representing six cancer types (breast, lung, gallbladder, cervical, colorectal and oral) were analyzed using IPD (details are provided in Supplementary Table S2). In addition, 1058 TCGA RNA-seq sample data consisting of 512 (472 tumors, 40 normal) colon adenocarcinomas (TCGA-COAD) and 546 (502 tumor, 44 normal) head and neck cancers (TCGA-HNSC) were also analyzed using IPD. Sample-wise IPD-based normalized pathogen quantification for these samples was compiled using in-house Python scripts. Heatmap representations were generated using the *pheatmap* package. Alignment files produced using IPD were used to compute the physical genome coverage of specific pathogens using SAMtools (30). Total microbiome quantification, representing archaea, bacteria, plasmids, viruses, fungi and protozoa, was done using Kraken2 (31). The read counts from Kraken2 were normalized by converting the read count to reads per million for each sample and are termed as total microbiome burden (TMiB).

### Mutual exclusivity calculations

HNSC tumor samples (in-house and TCGA-HNSC) were sorted based on the HPV burden. Top and bottom 10% tumors were assigned status as HPV-high and -low, respectively. In the case of matching values at the high and low boundaries, all the tumors having matching values were assigned the same status. A similar assignment of high and low status was done using the *F. nucleatum* burden. Tumors for which both HPV and *Fusobacterium* status was assigned were used for the mutual exclusivity analysis. Statistical significance for the mutual exclusivity was computed using CoMEt package (32).

### TCGA data download and pre-processing

The RNA-seq tier 1 data (aligned BAM) for TCGA-HNSC (*n* = 546) and TCGA-COAD (*n* = 512) project were obtained from the National Cancer Institute Cancer Genome Commons Portal (GDC; http://portal.gdc.cancer.gov). The BAM files were converted to raw fastq files using the SamToFastq utility of the Picard toolkit (https://broadinstitute.github.io/picard/). The raw fastq data were analyzed further for pathogen quantification using IPD. The htseq-count files for individual samples for RNA-seq and miRNA expression data were downloaded from the GDC. Immune and leukocyte fraction data for individual samples were downloaded from the GDC (https://gdc.cancer.gov/about-data/publications/panimmune). The neutrophil-to-lymphocyte ratio (NLR) was computed by dividing the neutrophil fraction by the aggregated lymphocyte fraction [as defined by CIBERSORT (33)] within individual samples.

### Differential mRNA/miRNA expression and statistical analysis

The differential gene and miRNA expression analysis was performed using the DESeq2 package (34). The false discovery rate of <0.05 was considered significant, and genes with log_2_ fold change ≥1.5 or ≤−1.5 were defined as significantly up- and downregulated, respectively. For miRNA comparisons, log_2_ fold changes of ≥0.5 and ≤−0.5 were defined as significantly up- and downregulated, respectively. Pathogen abundance comparison between the cancer types, immune fraction and mutation burden comparison between a group of samples (subtypes of samples based on pathogen abundance) were performed using the Wilcoxon test (Mann–Whitney), where factors showing *P*-value <0.01 were considered significantly different. RSEM quantification of the *MMP10* gene was extracted from the TCGA-HNSC matrix from the GDC. Comparison between the subgroups for the expression was performed using the Wilcoxon test. The survival analysis was performed using the KMPlot web server (35). *Fusobacterium* status was assigned to the samples assessed in the survival analysis based on the ‘Auto select’ option, which selects the best-performing cutoff between the upper and lower quartiles.

### PCR analysis for *F. nucleatum*

The following primer sequences were used for *Fusobacterium* validation as reported earlier (36). PCR was carried out in a 10 μl reaction volume containing 5 μl KAPA 2× ReadyMix Master Mix (Kapa Biosystems; cat. no. KK1024), 10 pmol of each primer and 20 ng of cDNA. The PCR conditions were as follows: an initial denaturation step at 94°C for 2 min, 35 cycles of denaturation at 94°C for 30 s, hybridization at 55°C for 30 s, elongation at 72°C for 30 s and a final extension step at 72°C for 5 min on a PCR machine (HiMedia Prima-Duo™ Thermal Cycler, two blocks of 48 wells; LA948). Sanger sequencing was performed to confirm the presence of *Fusobacterium* in the samples.

### Quantification of miRNA and mRNA genes from tongue tumor samples

Total RNA was extracted from tongue cancer patient samples using AllPrep DNA/RNA/miRNA Universal Kit (QIAGEN, cat. no. 80224) as per the manufacturer’s protocol. Extracted RNA was resolved on 1.2% agarose gel to confirm the RNA integrity. DNase treatment was done using a DNA-free™ kit (Ambion, Foster City, CA, USA; cat. no. AM1906). For analyzing transcript levels of *F. nucleatum* and miRNA target genes, cDNA was synthesized with 500 ng of total RNA using high-capacity cDNA reverse transcription kit (Applied Biosystems, cat. no. 4368814). For miRNA quantification, cDNA synthesis was carried out with 500 ng of total RNA using pooled miRNA-specific stem–loop RT primers [protocol as described (37)]. Quantitative real-time PCR was performed on QuantStudio5 Real-Time PCR instrument (Thermo Fisher Scientific) using Mir-X miRNA qRT-PCR SYBR Master Mix (Clontech Takara, cat. no. 639676) for miRNAs and KAPA SYBR Fast Universal Mix (KAPA Biosystems, cat. no. KK4601) for *Fusobacterium* and miRNA target genes. Expression of candidate miRNAs and genes was calculated as ΔCT. RNU48 was used as an internal control for microRNAs and beta-actin for *Fusobacterium* and genes.

### Analysis of the qPCR validation set oral patient samples

A set of 99 oral patient samples (tumor = 74, adjacent normal = 25) was used to perform real-time PCR-based quantification of *F. nucleatum* and validate the deregulated molecular factors associated with the pathogen levels. The validation set included 19 tumor–normal paired patient samples. qPCR-based levels of *F. nucleatum* were determined in the 74 tumors and the top and bottom quantiles (*n* = 18 in each group) of tumor samples were used as *Fusobacterium*-high and -low tumor samples, respectively. Gene and miRNA quantities were compared between these two groups of patient samples (*n* = 36 tumors) and significance was computed using the Wilcoxon rank-sum test.

## RESULTS

### Genomic landscape of pathogens across cancer type identifies mutual exclusivity of *F. nucleatum* and HPV in HNSC tumors

We reanalyzed TCGA and in-house generated 239 whole exome and 1168 transcriptome samples representing breast (38), lung (27), colorectal (28), gallbladder (39), head and neck (40), and cervical cancers (in-house sample statistics is provided in Supplementary Table S2) using IPD. IPD allows normalized quantification (scaled by FPM) for 1060 infectious pathogens, including the most common cancer-associated bacteria such as *B. fragilis*, *H. pylori* and *F. nucleatum*, and viruses such as HPV, Epstein–Barr virus, hepatitis C virus and others. HPV has been characterized as an etiological factor in cervical cancer (41) and is considered as an independent risk factor in oropharyngeal tumors (42). However, studies from our lab and others have reported no association of HPV with the Indian oral tumors (40,43–44). Our analysis of the exome samples reiterates that there is no evidence of the presence of HPV in the in-house oral cancer samples (*n* = 46); however, the prevalence of HPV 16 and related strains was found in 44.18% cervical tumors (range = 0–254.04 FPM, mean = 14.62 FPM). Among the bacterial species, we observed abundance of *B. fragilis* (range = 0–10.95 FPM, mean = 0.275 FPM) and *F. nucleatum* (range = 0–0.458 FPM, mean = 0.045 FPM) in 11.63% and 48.15% colorectal tumors (12,45), respectively. Enrichment of no known cancer-associated pathogen was observed in lung cancer samples. However, we observed traces of *Pseudomonas* in 82.69% of lung tumors (range = 0–39.34 FPM, mean = 1.27 FPM), which is reported to be enriched in cancer patients with febrile neutropenia (46), a secondary infection observed post-therapy in lung cancer patients. A high abundance of *Escherichia* was detected at DNA level across cervical, colorectal, lung and gallbladder tumors. Inflammatory bacteria such as *Neisseria*, *Pseudomonas*, *Prevotella*, *Streptococcus* and *Fusobacterium* were found to be specifically present in the oral tumor samples (Supplementary Figure S1). The abundance of these and other bacteria has been confirmed by metagenomic sequencing studies in head and neck tumors (19,47).

**Figure 1.**
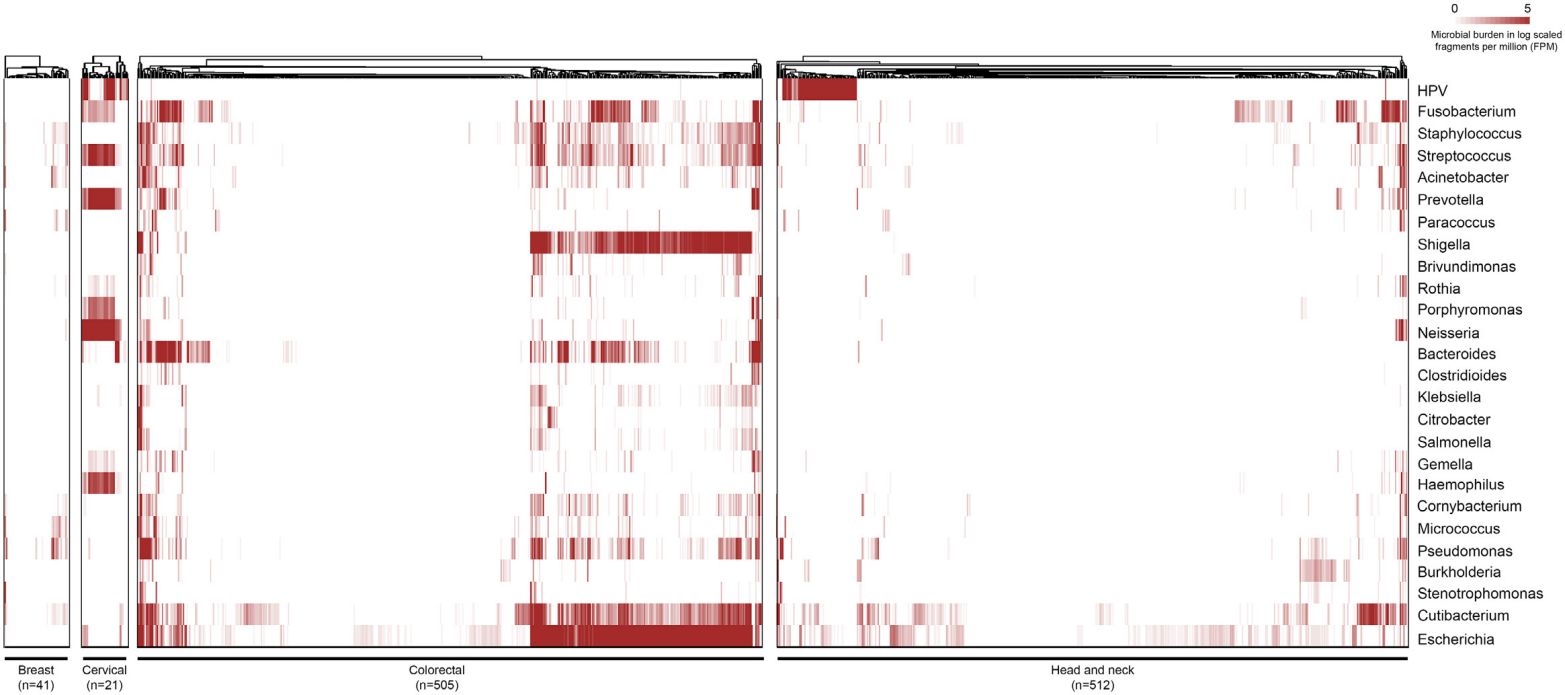
Landscape of infectious pathogens across breast (*n* = 41), cervical (*n* = 21), colorectal (*n* = 505), and head and neck (*n* = 511) tumors. The column width for the breast and cervical tumors is augmented (not to scale). The pathogen burden depicted in the plot is log-scaled FPM, as generated by the IPD tool.

From the transcriptome dataset of the breast, cervical, colorectal, and head and neck cancer samples analyzed using IPD, distinct pathogen signatures were observed among the tumors (Figure 1). Since the transcriptome dataset used in the analysis is generated using two different capture methodologies (polyA and ribo-depleted), we checked for the difference in the pathogen quantification due to sequencing method/protocol. The total microbial read count generated by Kraken2 was found to be comparable across the tumors sequenced using two capture methods (Supplementary Figure S2A and B). Further principal component analysis (PCA) was performed to assess any batch effect due to sequencing protocols in the data. However, no specific clustering of the samples was observed based on the IPD-based pathogen read counts (Supplementary Figure S3). Among the tumor types analyzed, breast tumors were found to bear the least infectious pathogen burden for the 1060 pathogens quantified. Recent studies have reported enrichment of *Fusobacterium* in breast tumors (48); however, we did not find similar enrichment in our in-house breast transcriptome samples. We further computed the TMiB (as described in the ‘Materials and Methods’ section), which indicates the proportion of captured pathogen reads among the reads sequenced per sample. The in-house breast transcriptome samples showed comparable TMiB to other tumor types, suggesting that the low *Fusobacterium* burden certainly depicts the absence of pathogen and may not be due to lack of capture of overall microbiota in these tumors (Supplementary Figure S4A and B). Among the transcriptome of cervical tumors, expression of HPV was observed in 90.48% samples (range = 0–325.79 FPM), consistent with earlier reports (49). The common inflammatory bacterium observed in the DNA samples was also found to be expressed in the transcriptome data across the cervical, colorectal, and head and neck tumors. Colorectal tumors form a subgroup associated with the overabundance of *Fusobacterium* (12,36). From the analysis of TCGA colon adenocarcinoma (TCGA-COAD, *n* = 521) and in-house colorectal (*n* = 33) transcriptome samples, we observed pathogenic *E. coli* (95.33%; range 0–167 90.79 FPM), *Shigella* (94.915%; range 0–2481.066 FPM) and *Cutibacterium* (94.703%; range 0–2129.54 FPM) to be the top abundant pathogens. The known colorectal oncobacteria *Fusobacterium* and *Bacteroides* were found to be expressed in 78.21% (range = 0–2460.4 FPM, mean = 19.16 FPM) and 83.5% (range = 0–4064.5 FPM, mean = 24.88 FPM) in the colorectal tumor samples, respectively.

In the HNSC (*n* = 512) tumor samples, which included in-house oral and TCGA-HNSC samples, we observed the high abundance of HPV (abundance >1 FPM) in 12% (*n* = 63) of the HNSC tumors, consistent with earlier reports (50). HPV is one of the prominent etiological factors of oropharyngeal tumors (51) and is known as the cancer-causing agent in head and neck tumors (52). Among the known pathogens associated with HNSC, *Fusobacterium* traces were present in 75.9% of HNSC tumors (range = 0–98.66 FPM, mean = 2.35 FPM), comparable to the colorectal tumors (78.2%). We observed that HPV and *Fusobacterium* formed distinct clustering of samples in the HNSC tumor. By using the tumor samples having the pathogen high and low status defined (described in the ‘Materials and Methods’ section), mutual exclusivity was calculated for the two pathogens. Interestingly, HPV and *Fusobacterium* were found to be mutually exclusive in the HNSC tumors (*P*-value = 0.0146). A map depicting overlap and prevalence of the four major pathogens (HPV, *Fusobacterium*, *Bacteroides* and pathogenic *E. coli* IAI39), across the transcriptome dataset, has been summarized in Supplementary Figure S5A and D. We further evaluated whether the *Fusobacterium*-rich subgroup of tumors also formed a distinct molecular and/or clinical subtype of the HNSC tumors.

### *Fusobacterium* is associated with an inflamed microenvironment in the head and neck tumors

As various HPV strains are known to impart a significant influence over the gene and immune expression profile in head and neck cancer (53), all the tumors showing HPV expression >1 FPM were annotated as HPV-positive. To correlate *Fusobacterium* abundance with molecular and clinical features, we used the 438 HPV-negative tumor samples from TCGA-HNSC. These were then divided into *Fusobacterium*-high and -low, by sorting the samples based on pathogen burden gradient. Of these, 133 tumors showed no expression of *F. nucleatum* and were considered to be *Fusobacterium*-low and the top 10% quantile (*n* = 44) as *Fusobacterium*-high. We performed differential expression analysis between these two subgroups, which identified 60 upregulated and 198 downregulated genes (Supplementary Table S3). Reactome (54)-based overrepresentation analysis of the upregulated genes showed keratinization/cornified envelope formation (*P*-value = 7.88e−15) and interleukin signaling (*P*-value = 7.88e−15) as the top enriched pathways, which are also reported to be primarily activated in the oral epithelium during bacterial dysbiosis and inflammation (55) (Supplementary Table S4). To get a comparative account of the pathogen-induced expression profile change, a similar analysis was performed between HPV-positive versus -negative TCGA-HNSC tumors. We observed 209 upregulated and 553 downregulated genes in HPV-positive tumors. Comparing the list of deregulated genes among *Fusobacterium*-based and HPV-based differential analysis, 34 genes were found to be commonly downregulated (Supplementary Figure S6A); however, no gene was commonly upregulated among the two subgroups (Supplementary Figure S6B). This suggests a distinct role of the two pathogens in the modulation of the HNSC tumor gene expression profile.

**Figure 2.**
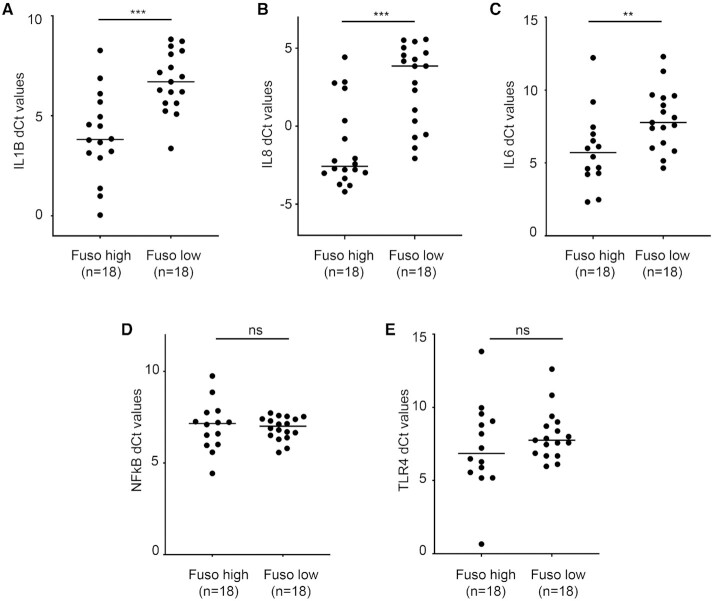
qPCR-based validation of inflammation marker genes (**A**) *IL1B*, (**B**) *IL8*, (**C**) *IL6*, (**D**) *NFκB* and (**E**) *TLR4* in the *Fusobacterium*-high and -low in-house tongue tumor samples (*n* = 18 each). Samples with Ct value greater than 32 were excluded from the analysis.

Among those differentially expressed, *MMP10*, a potential biomarker for prediction of nodal metastases in oral cancer (unpublished data), was found to be significantly higher in the *Fusobacterium*-high subgroup (Supplementary Figure S7), suggesting a potential association between the *Fusobacterium* load and nodal metastases. Additionally, multiple inflammatory pathway genes (*n* = 118) were found to be upregulated (adjusted *P*-value <0.05) in the *Fusobacterium*-high TCGA-HNSC subgroup. Among the inflammatory pathway genes, we selected *IL1B*, *IL6* and *IL8* as representative effector molecules for the real-time qPCR validation. The in-house validation sample set consisted of clinically assessed early-stage tongue tumors. In the validation cohort (*n* = 74), real-time qPCR of 16S RNA gene of *Fusobacterium* was performed and the upper and lower quantile tumors were stratified as *Fusobacterium*-high and -low, based on their pathogen load (described in the ‘Materials and Methods’ section). There were 19 tumor–normal pairs in the validation sample set, in which we compared the levels of *Fusobacterium*. However, no difference was observed in its levels in the tumor and normal samples (Supplementary Figure S8). Analysis showed that the three selected pro-inflammation markers (*IL1B*, *IL8*, *IL6*) were significantly higher in the *Fusobacterium*-high tumor samples (Figure 2A–C), suggesting elevated levels of inflammation in these tumors. Being inflammatory response mediators, overexpression of *TLR4* and *NFκB* is reported in response to *Fusobacterium* infection in colorectal tumors (56). However, we observed no difference in the mRNA expression levels of *NFκB* and *TRL4* in the in-house *Fusobacterium*-high and -low tongue tumor subgroups (Figure 2D and E), unlike colorectal cancer.

**Figure 3.**
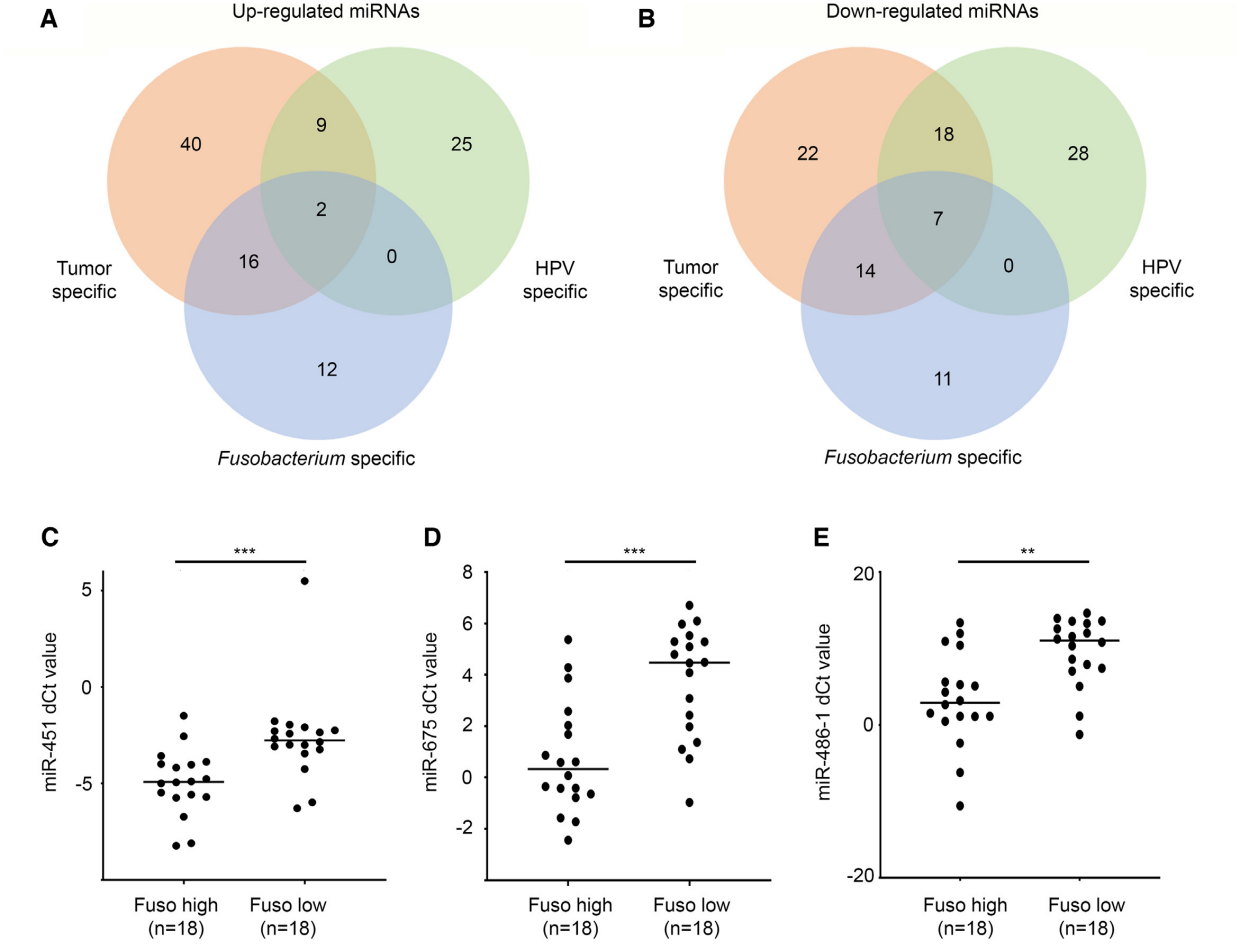
MicroRNAs associated with the load of *Fusobacterium* in the HPV-negative HNSC tumors. (**A**, **B**) Up- and downregulated miRNAs in the tumor versus normal (red), HPV-positive versus -negative (green) and *Fusobacterium*-high versus -negative (blue) tumors, respectively. Differentially expressed miRNAs *miR-451* (**C**), *miR-675* (**D**) and *miR-586-1* (**E**) validated using qPCR in the *Fusobacterium*-high and -low in-house tumor samples (*n* = 18 each).

Inflammatory pathways are also mediated by the expression of specific miRNAs and have been considered as useful biomarkers in cancer, as well as pathogenic infections (57). In the TCGA-HNSC HPV-negative tumors, we performed differential miRNA expression analysis between the *Fusobacterium-*high (*n* = 44) versus *Fusobacterium*-negative (*n* = 130) tumors. Similarly, differential miRNA expression analysis was also performed between the TCGA-HNSC tumor versus normal, and HPV-positive versus -negative tumor samples. In total, 62 miRNAs (30 upregulated and 32 downregulated) were identified to be deregulated (Supplementary Table S5) in the *Fusobacterium*-based comparison. Of the 62, by overlapping with the tumor–normal and HPV positive–negative differential miRNA lists, we identified 12 upregulated and 11 downregulated miRNAs that were specific to the *Fusobacterium*-high subgroup of the HNSC tumors (Figure 3A and B). We selected top five upregulated (*hsa-mir-451a*, *hsa-mir-675*, *hsa-mir-203a*, *hsa-mir-144*, *hsa-mir-486-1*) and three downregulated (*hsa-mir-1269b*, *hsa-mir-9*, *hsa-mir-598*) miRNAs for validation in the in-house tongue cancer samples (details are provided in Table 1). Of the upregulated miRNAs, expression levels of *hsa-mir-451a*, *hsa-mir-675* and *hsa-mir-486-1* were found to be significantly elevated in the *Fusobacterium*-high subgroup of tongue tumors (Figure 3C–E), whereas others were invalidated. Individually, the three validated miRNAs have previously been reported to be involved in inflammation (58–60). Additionally, the experimentally validated targets of these miRNAs have also been reported to be directly involved in the inflammatory pathway. For example, *has-mir-451a* targets major genes in the inflammatory pathway, such as *MIF* (61), *AKT1* (62), *MYC* (63) and *IKBKB* (64), among others. *CD40*, a major mediator of inflammation response toward bacterial infection, is a known target of *hsa-mir-486-1* (65). However, *hsa-mir-675* targets *RUNX1* (66), a major suppressor of *TLR4*-based inflammatory response (67). This suggests direct as well as indirect involvement of the three validated miRNAs in inflammatory response management. Overall, we identified several *Fusobacterium*-associated miRNAs in the head and neck tumors and validated three *hsa-mir-451a*, *hsa-mir-675* and *hsa-mir-486-1* to be positively correlated with *Fusobacterium* burden.

### *Fusobacterium*-high HNSC tumors are associated with a pro-tumorigenic immune microenvironment

**Figure 4.**
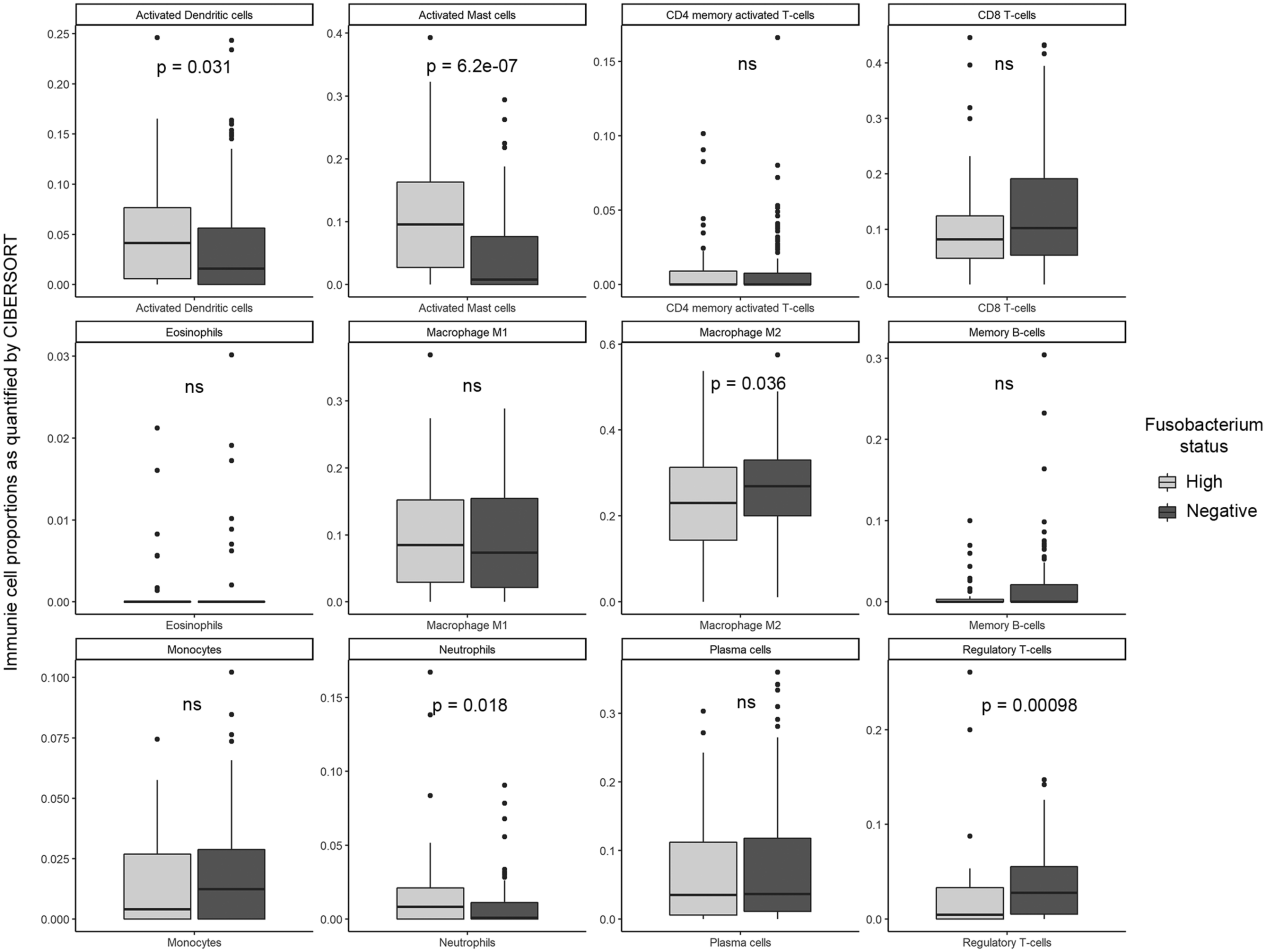
Comparison of individual immune cell type fractions (based on the CIBERSORT scores) among the TCGA-HNSC *Fusobacterium*-high (*n* = 44) versus *Fusobacterium*-negative (*n* = 130) subgroups.

*Fusobacterium* abundance has been shown to influence the tumor immune microenvironment and gene expression profile in colorectal tumors (68,69). Gene expression-based approaches, such as CIBERSORT (33), are used to decipher the fraction of individual immune cells from tumor environment. We thus obtained the precomputed, CIBERSORT-based immune cell fraction scores for the TCGA-HNSC tumor samples from the GDC and compared their levels between the *Fusobacterium*-high and -low TCGA-HNSC subgroups. Among the various immune cell types, activated mast cell (*P*-value = 6.2e−07), neutrophil (*P*-value = 0.018), activated dendritic cell (*P*-value = 0.031) and M2 macrophage (*P*-value = 0.035) fractions (scores) were enriched, whereas regulatory T-cell (*P*-value = 9.8e−04) fractions were significantly low in the *Fusobacterium*-high subgroup (Figure 4). There was no significant difference observed between the major antitumor immune cell (CD8^+^ and CD4^+^ T cells and B cells) composition between the two subgroups, which is in contrast to colorectal tumors (70). Based on the immune cell enrichment scores, we also computed the NLR, which is a marker for systemic inflammation and an important prognostic marker in head and neck tumors (71). NLR was significantly found to be higher in the *Fusobacterium*-high (*P*-value = 0.0084) TCGA-HNSC subgroup (Supplementary Figure S9). Immune signature analysis indicates that *Fusobacterium* load is associated with an inflamed, innate immune cell-enriched and potentially pro-tumorigenic microenvironment, as opposed to the HPV-positive HNSC tumors that display T-cell inflamed phenotype (72).

### *Fusobacterium* burden is associated with poor survival and higher extracapsular spread among tongue cancer patients

**Figure 5.**
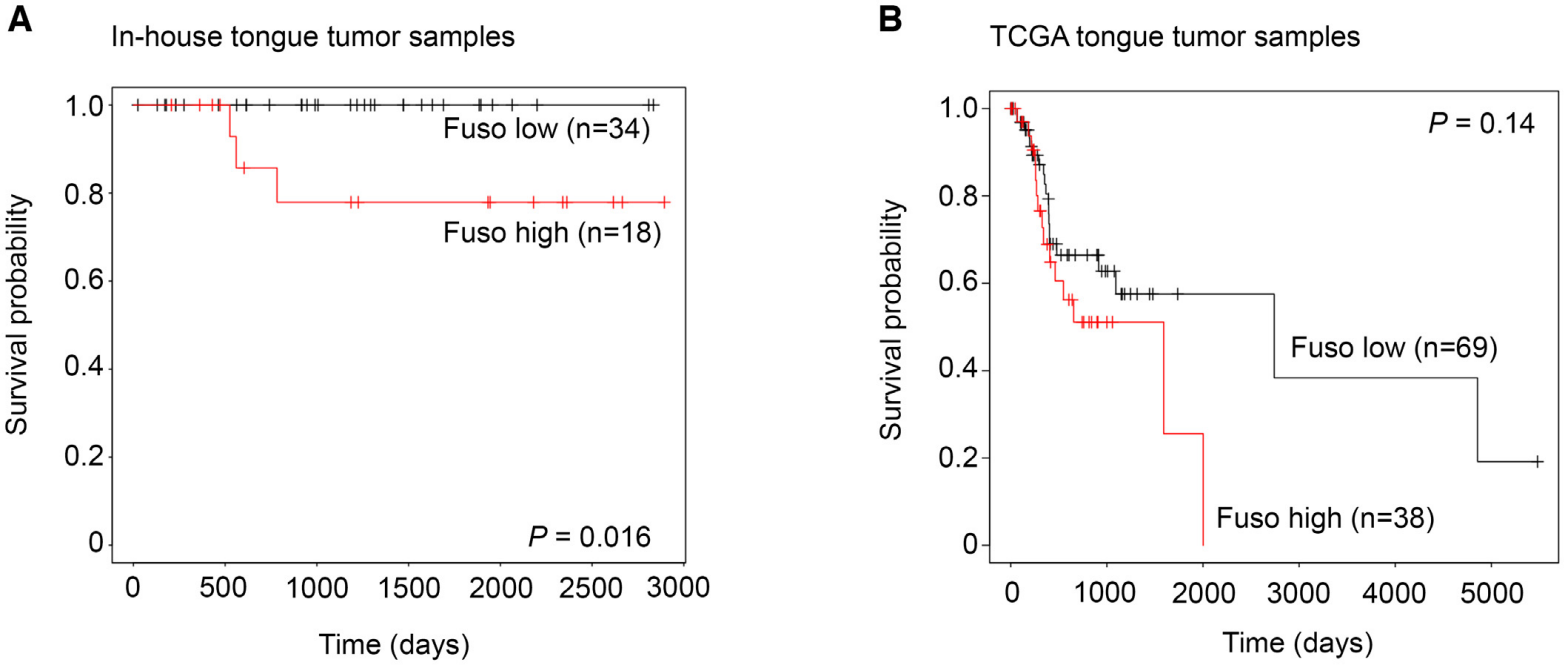
Survival analysis between the *Fusobacterium*-high and -low burden subgroups in the (**A**) in-house (*n* = 62) and (**B**) HPV-negative TCGA oral tongue patient samples (*n* = 108).

Since the *Fusobacterium* levels are shown to have elevated inflammation and a distinct immune profile, we further evaluated its possible relation with clinical features and patient survival. From the validation cohort (detailed in Table 1) of the in-house tongue tumors, the clinical feature and survival details were available for 52 of the 74 patients screened for *Fusobacterium*. For these patients, the *Fusobacterium*-high and -low status was assigned based on the best-performing CT value cutoff of KMPlot. Comparing the overall survival, the in-house patient samples showed that *Fusobacterium*-high patients showed poor survival (log-rank *P*-value = 0.016) (Figure 5A). Since all the in-house patient samples were of tongue origin, we segregated the TCGA-HNSC HPV-negative tumors based on the defined anatomical site and performed survival analysis using the patients with tumor of tongue origin (*n* = 107). Consistent with the observation from the in-house tumor samples, the TCGA tongue cancer patients with *Fusobacterium*-high status showed a trend of poor overall survival (hazard ratio = 1.67, log-rank *P*-value = 0.14) (Figure 5B). We further applied Cox proportional hazard model to describe risk factors. In addition to *Fusobacterium* load status, alcohol usage, smoking status, tobacco usage, nodal status, disease recurrence status, perineural invasion, lymphovascular invasion and extracapsular spread status were used as covariates. Among these, *Fusobacterium*-high status (2.66e+01), smoking (5.24), tobacco (1.34e+01), extracapsular spread (2.59e+01) and disease recurrence status (4.02e+01) showed positive regression coefficients, although none of the factors was statistically significant. Next, we performed association analysis between the *Fusobacterium* burden status (as assigned by KMPlot) with lymph node positivity, lymphovascular invasion, perineural invasion, extracapsular spread and recurrence status of the in-house tongue tumors. Among these, higher *Fusobacterium* was associated with the presence of extracapsular spread (Fisher test; odds ratio = 4.98, *P*-value <0.05). *Fusobacterium* load was not found to be associated with alcohol consumption, tobacco smoking or chewing status in the patient cohort. Overall, *Fusobacterium* burden was found to be associated with poor prognosis and higher extracapsular spread, especially in tongue tumor cancer.

## DISCUSSION

Pathogen-induced inflammation is a major factor determining the disease outcome and response to therapy (73,74). Several pathogens have been categorized as cancer risk factors in different cancers, with studies emphasizing the role of periodontal pathogens in head and neck cancer. Enrichment of specific bacterial populations has been associated with tumors, mainly using metagenomic sequencing datasets. Recently, identification of viral and bacterial pathogens has also been performed from the human DNA and RNA sequencing data, using varied computational tools (22). In this study, we systematically analyzed 239 whole exome and 1168 whole transcriptome sequencing datasets representing six tumor types: breast, cervical, colorectal, lung, gallbladder, and head and neck cancers, to quantify the abundance of 1060 pathogens using our previously published method (IPD). This method allows us to generate a normalized quantification for diverse cancer-associated pathogens among different tumor types, directly from the NGS datasets. From the landscape of pathogens identified across the sequencing dataset, we observed traces of known cancer-associated pathogens such as HPV in cervical and HNSC tumors, *B. fragilis* and pathogenic *E. coli* in colorectal tumors, and *F. nucleatum* in colorectal and HNSC tumors. Interestingly, *Fusobacterium* persistence was found to be in the comparable frequency in colorectal and HNSC tumors. In the HNSC transcriptome samples, HPV was found to be forming a distinct subtype. HPV-associated HNSC tumors have been previously characterized and are reported to form a distinct clinical and molecular subtype (50). Specifically, in the HPV-negative oral tumors, periodontal pathogens have been reported to be a risk factor (21). In our analysis, we observed that *Fusobacterium*-high tumors formed another distinct subgroup in HNSC, which was mutually exclusive to the HPV-high subgroup. *Fusobacterium* has been primarily reported to be associated with poor prognosis and recurrence in colorectal tumors (36,75). Along with other inflammation-inducing pathogens, it has also been reported to be significantly enriched in head and neck tumors (19,76). Further, only considering the HPV-negative TCGA-HNSC samples (*n* = 438), we classified tumors having top 10% pathogen load as Fuso-high and with no expression as Fuso-negative.

To unveil the molecular and clinical features associated with *Fusobacterium* burden in HNSC, we performed differential gene expression analysis between the *Fusobacterium*-high versus -low tumors. The significantly upregulated genes were strikingly different from the genes upregulated in HPV-positive HNSC tumors. *Fusobacterium*-high tumors had 118 inflammatory pathway genes upregulated, suggesting an inflamed tumor microenvironment. Among these, MMP10, a biomarker for lymph node metastases in oral tumors (unpublished data), was also found to be positively associated with *Fusobacterium* burden in TCGA-HNSC tumors. This suggests a possible role of *Fusobacterium* in nodal metastases in oral tongue tumors by induction of chronic inflammation. To further validate the association between *Fusobacterium* burden and inflammation, we evaluated the levels of pro-inflammatory cytokines *IL1B*, *IL8* and *IL6* in *Fusobacterium*-high and -low in-house early-stage tongue tumor samples (*n* = 75). Expression of these cytokines was found to be significantly higher in the tongue tumors having a higher load of *Fusobacterium*. These inflammation markers are known to get activated upon TLR signaling. Monolayer co-infection experiments have shown that *Fusobacterium* induction enhances *TLR* signaling in cell line and mouse models (77,78). However, the transcript levels of *NFκB* and *TLR4* remain unaltered in *Fusobacterium*-high tongue tumors, unlike colorectal tumors wherein the activity as well as transcriptional elevation is observed upon *Fusobacterium* infection. Inflammation is also known to be tightly regulated by the miRNA expression profile in the backdrop of infection and cancer. We identified differentially expressed miRNAs in *Fusobacterium*-high versus -negative, HPV-negative TCGA-HNSC tumors and compared the list of deregulated miRNAs with the ones associated with HPV and HNSC tumor only. From the miRNAs deregulated specifically in the *Fusobacterium*-high group, we selected eight (five upregulated and three downregulated) miRNAs for validation in the in-house tongue cancer patient cohort. Of these, three upregulated miRNAs *miR-451a*, *miR-675* and *miR-486-1* were validated to have significantly higher expression in the *Fusobacterium*-high tongue tumors. These three miRNAs have been previously independently reported to be biomarkers of progression, treatment response and disease prognosis in different tumor types and have also been linked to inflammation (58–60,79).

Viral infection and bacterial dysbiosis have been linked to a distinct immune profile of the tumors. Very little is known about the HPV-negative HNSC tumors in terms of the immune cell composition and function (80). We evaluated the immune cell composition of the HPV-negative HNSC tumors and observed enrichment of innate immunity cell types, including neutrophils, M2 macrophages, mast cells and dendritic cells, in tumors with a higher burden of *Fusobacterium*. These tumors were also found to have a higher NLR, indicating an inflamed state. The expression analysis of the inflammation-related genes and immune cell enrichment analysis suggest *Fusobacterium* burden is a highly inflamed, pro-tumorigenic tumor microenvironment.

Further, we performed a correlation of the *Fusobacterium* burden with various clinical features. We primarily observed that a higher burden of the pathogen is associated with the lower overall survival in in-house tongue cancer patients. We extended the analysis to the HPV-negative TCGA oral tongue tumor patients and observed a marginally significant difference in the survival, consistent with the in-house tongue cancer samples. We also performed an association analysis of the pathogen burden with different clinical features, including perineural invasion, lymph node presentation, and extracapsular and lymphovascular spread. Among these, we observed a significant association of *Fusobacterium* burden with the presence of extracapsular spread in tongue cancer patients. This suggests that a higher *Fusobacterium* burden is a poor prognosis factor and may be associated with higher invasion and metastasis, especially in patients with tongue tumors. Since the clinical association and validation patient samples were primarily clinically assessed for early-stage tumors, this study highlights role of *Fusobacterium* in the early-stage tongue tumors, which are on the rise in the developing countries. The role of *Fusobacterium* in tongue cancer may follow the disease progression models implicated in colorectal and pancreatic cancers, with tumor type and site-specific presentation of the molecular features. Recent studies have also shown *Fusobacterium* burden to be associated with recurrence, smoking and tobacco chewing status among other clinical features in head and neck cancer (81). In our samples, we did not observe these associations, probably because of the smaller cohort size. Another considerable deficiency of this study is unavailability of clinical information regarding treatment history and pre-operative prophylactic antibiotic treatment, if any. Since both of these factors may influence the prevalence of microbes in the tumor microenvironment, inclusion of this information may further add significance, also with respect to the disease outcome in the context of prevailing infection. Additionally, capture of pathogens from the human exome sequencing is primarily dependent on the nonspecific cross-hybridization of the pathogen DNA with the probes. Also, the different types of RNA sequencing capture kits (such as polyA and ribo-depletion) may have differential sensitivities for detecting the microbial transcripts, affecting their quantification in the computational subtraction. Finally, the *Fusobacterium* burden and its tolerance across ethnicities may be driven by oral hygiene, immunity, geographical and dietary factors, which may present a different outcome in different populations.

Our study presents a comprehensive landscape of pathogens identified from the genomic dataset of Indian origin, across six different tumor types. Combining the TCGA data, we identify a subgroup of poor prognosis tongue tumors, which may be primarily driven by the inflammation due to pathogens such as *Fusobacterium* and points to important clinical implications of the burden of the periodontal pathogens, especially in HPV-negative head and neck cancer patients. This may provide a rationale to further design studies to understand the variability in treatment response, disease recurrence and resistance, and adoption of additional measures such as the use of antibiotics for tumors of specific origin, similar to colorectal cancer.

## DATA AVAILABILITY

All the genomic datasets analyzed in the manuscript can be accessed from the ArrayExpress database under the accession numbers E-MTAB-11412, E-MTAB-9766, E-MTAB-6619, E-MTAB-4653, E-MTAB-8801, E-MTAB-11404, E-MTAB-9281 and E-MTAB-11407. The colorectal cancer data have been deposited at the European Genome-phenome Archive, under accession number EGAS00001005970.

## Supplementary Material

zcac006_Supplemental_Files
