# Supplementary material for: Fusobacterium nucleatum is associated with inflammation and poor survival in early-stage HPV-negative tongue cancer
Source: NAR Cancer. 2022 Mar 4;4(1):zcac006. doi: 10.1093/narcan/zcac006 (PMC8894079; doi:10.1093/narcan/zcac006)
Supplement: zcac006_Supplemental_Files [file zcac006_supplemental_files.zip › supplementary_info_with_legends_merged.pdf]

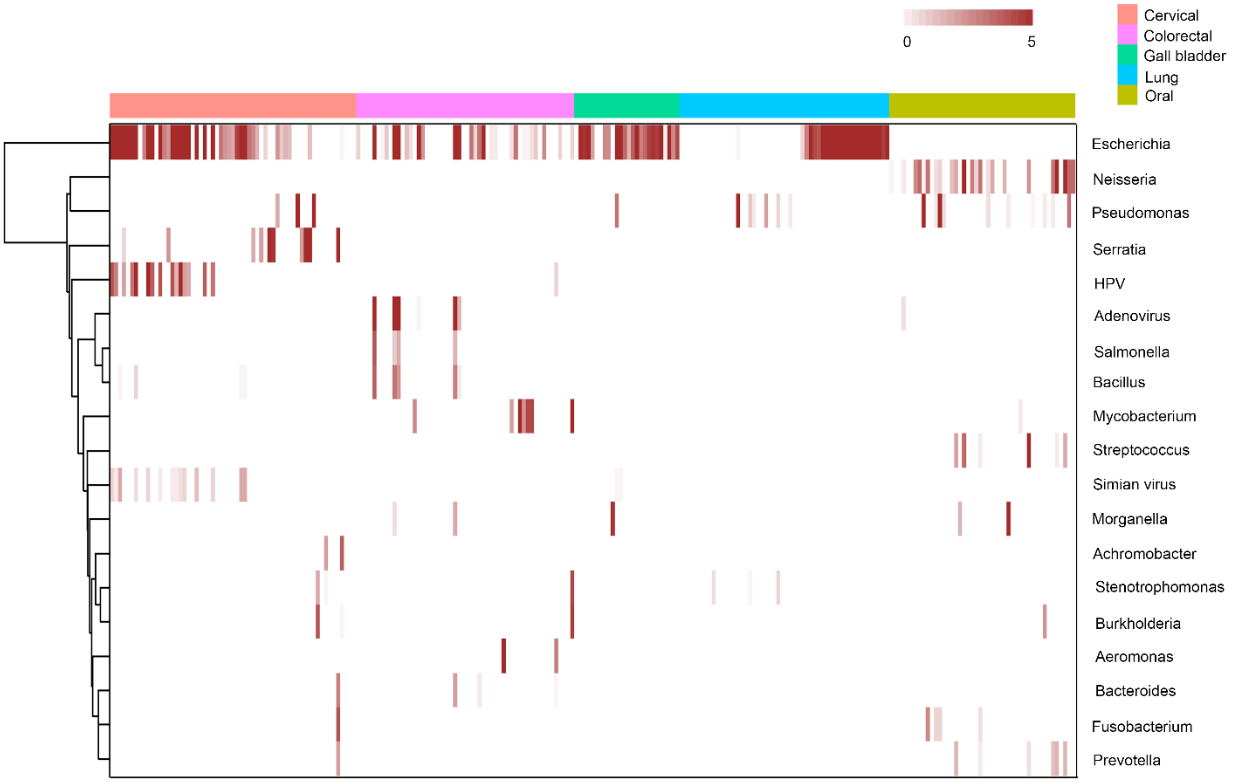

Supplementary Figure S1: Landscape of pathogens across in-house exome sequenced samples representing cervical, colorectal, lung, gall bladder and oral tumor samples.

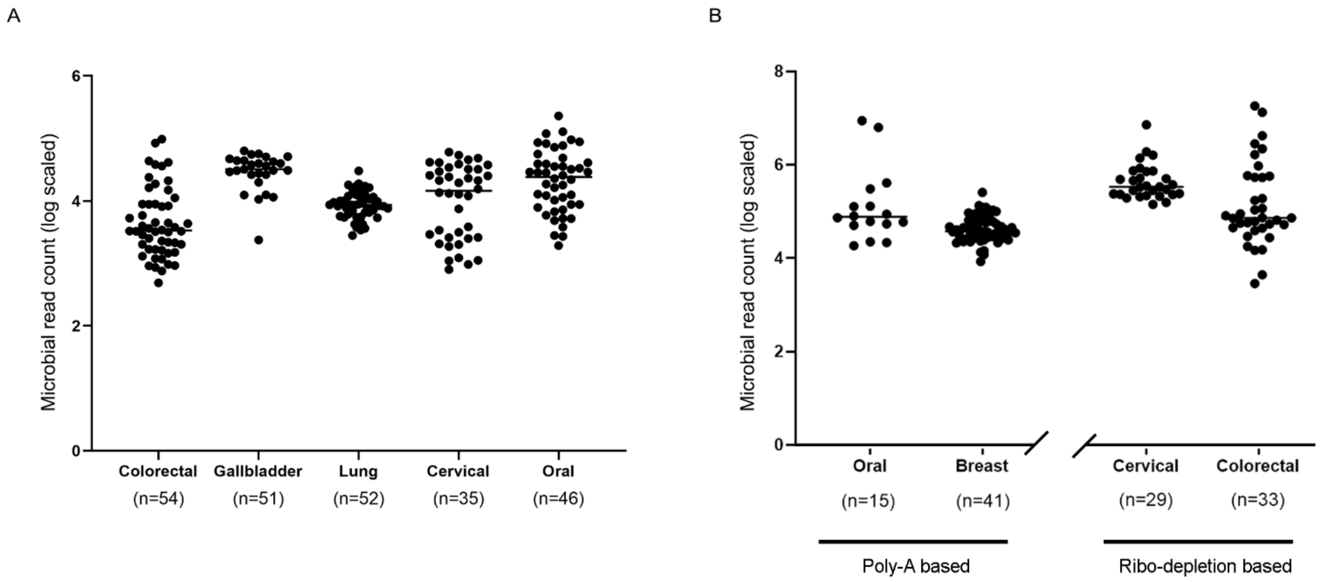

Supplementary Figure S2: Microbial read count across in-house samples using Kraken2 A) exome (n=239) and B) transcriptome (n=110) representing breast, cervical, colorectal, gall bladder and oral tumors. Among the transcriptome samples, the inhouse breast and oral tumors have been sequenced using poly-A based capture, whereas inhouse colorectal (n=33) and cervical (n=29) tumors have been sequenced using ribo-depletion based method. The microbial read count are comparable for data generated using the mentioned capture methodology.

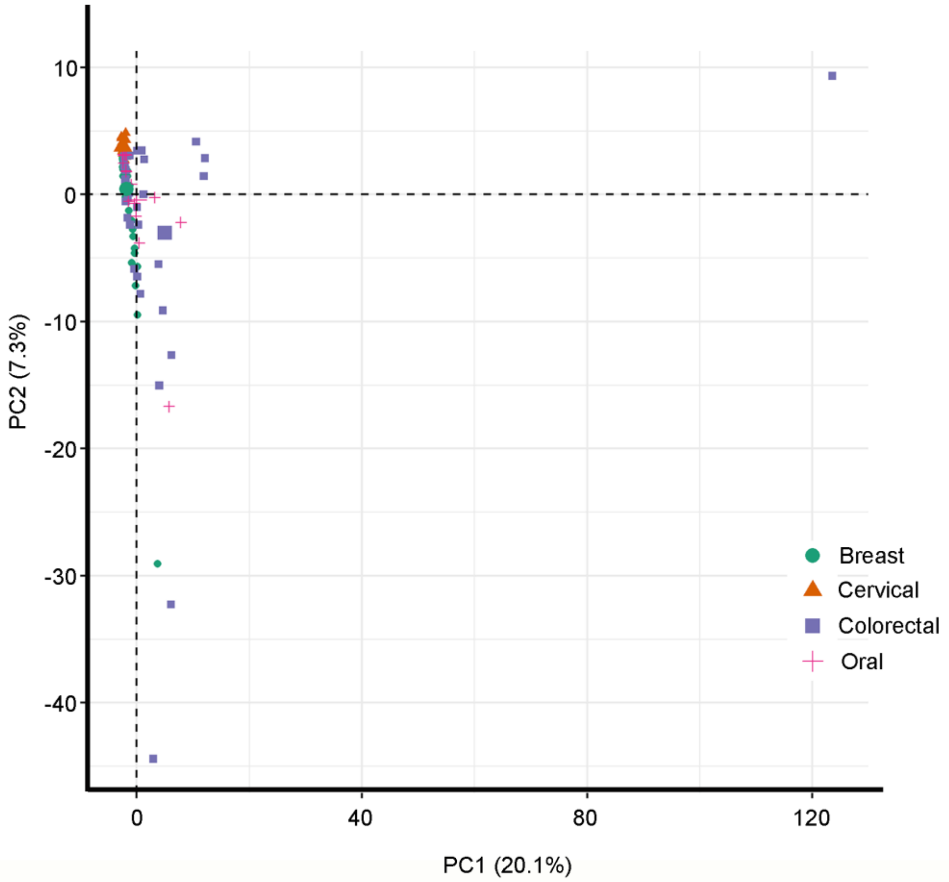

Supplementary Figure S3: Principal component analysis (PCA) plot of the inhouse samples based on IPD microbial counts. Each dot represents individual sample.

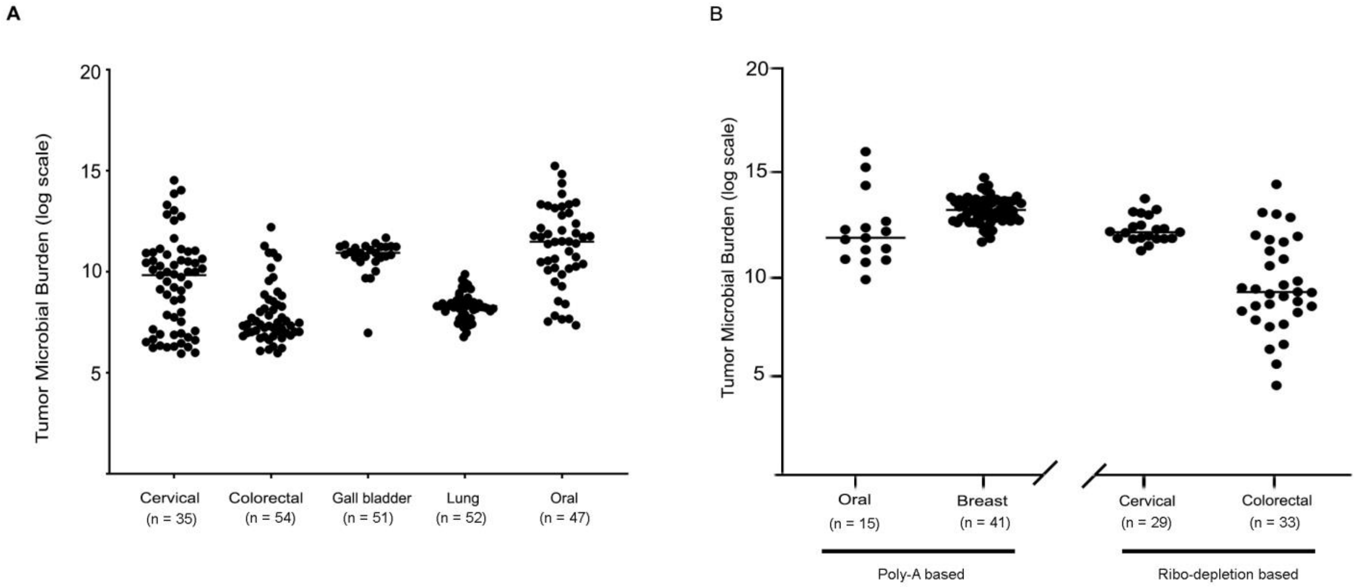

Supplementary Figure S4: Tumor Microbiome Burden (TMiB) across in-house A) exome (n=239) and B) transcriptome (n=110) samples representing breast, cervical, colorectal, gall bladder and oral tumors. Among the transcriptome samples, the inhouse breast and oral tumors have been sequenced using poly-A based capture, whereas inhouse colorectal (n=33) and cervical (n=29) tumors have been sequenced using ribo-depletion based method. The TMiB are comparable for data generated using the mentioned capture methodology.

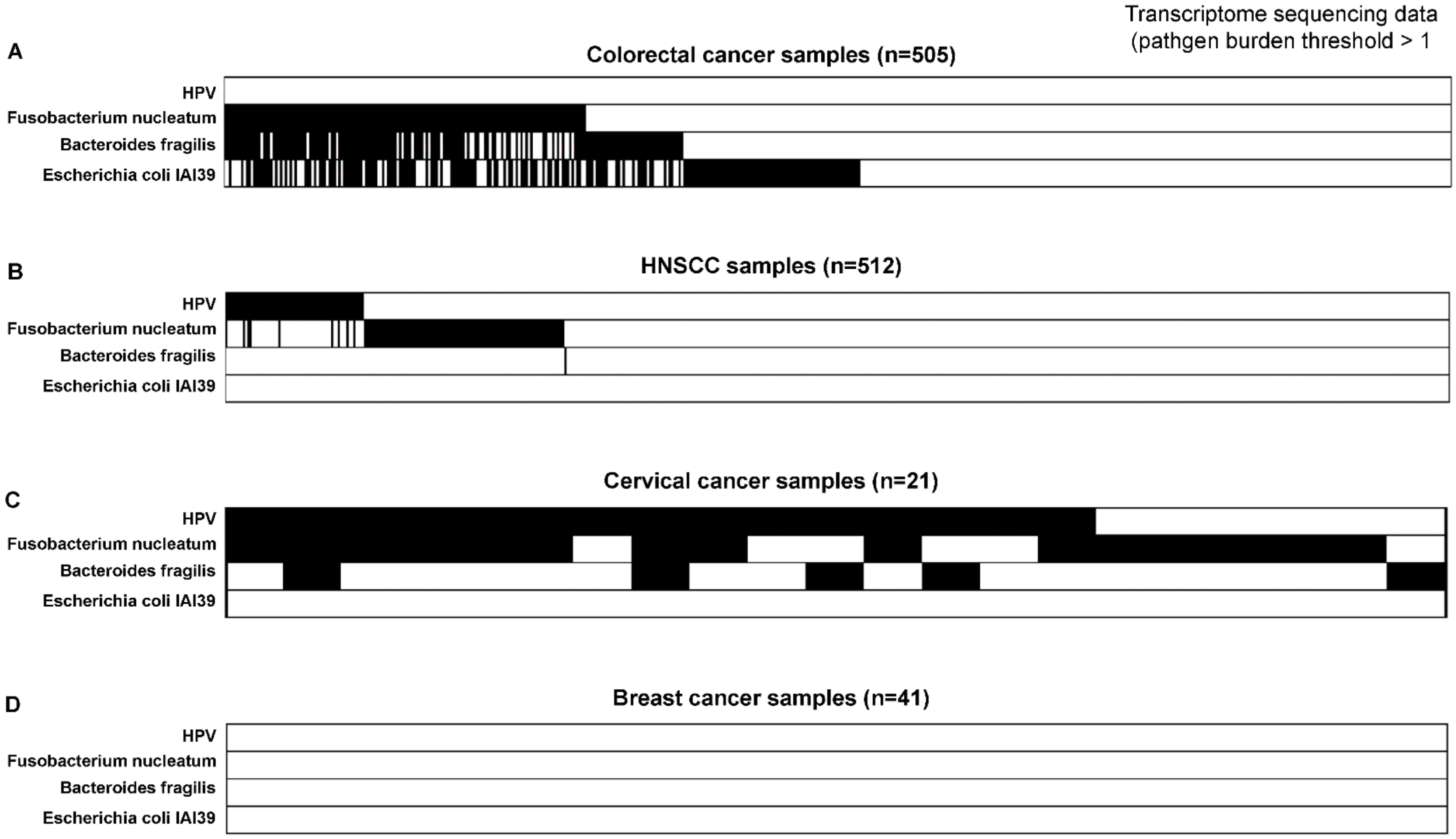

Supplementary Figure S5: Prevalence of the most common cancer-associated pathogens (HPV, Fusobacterium, Bacteroides and pathogenic Escherichia coli IAI39) in the RNA-Seq samples from colorectal (A), HNSC (B), cervical (C), and breast (D) tumors. Black indicates the presence of a pathogen in a particular sample at >= 1 Fragment Per Million (FPM) level. In the pathogen occurrence plot, the sample size for each cancer type has been normalized to 100 percent.

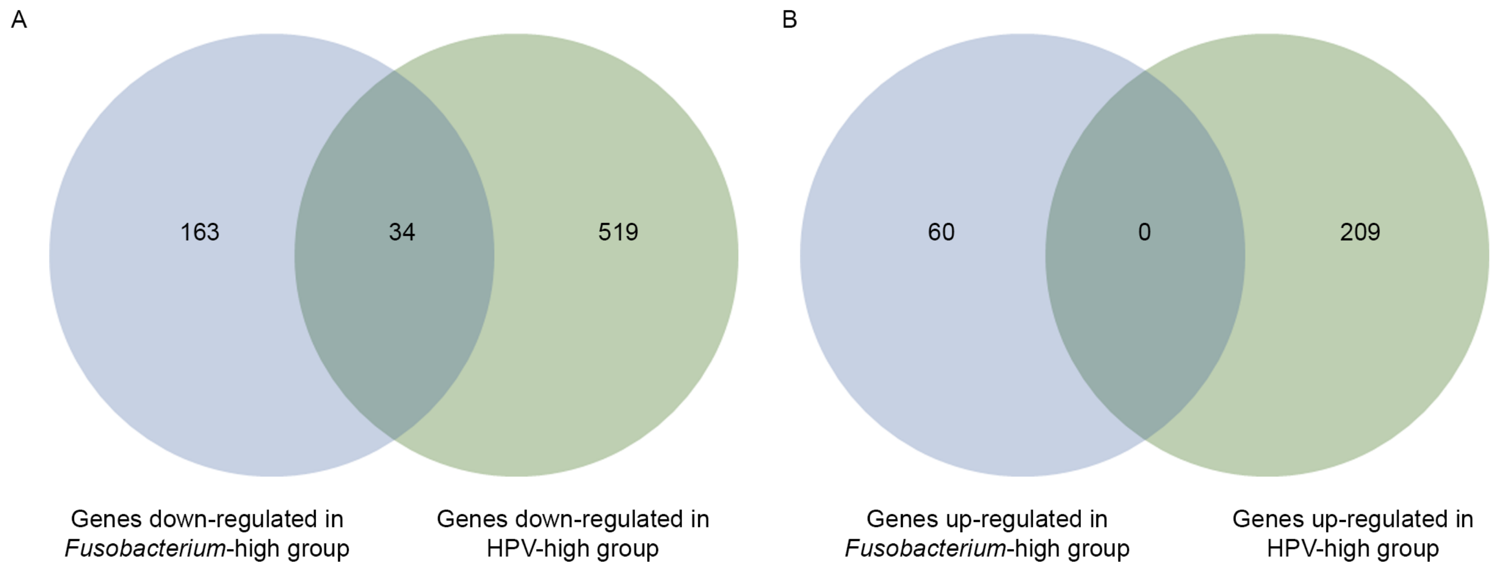

Supplementary Figure S6: Overlap between the down- (A) and up- (B) regulated genes in the Fusobacterium and HPV based comparison. Blue indicates the Fusobacterium group comparison and green indicates HPV based comparison.

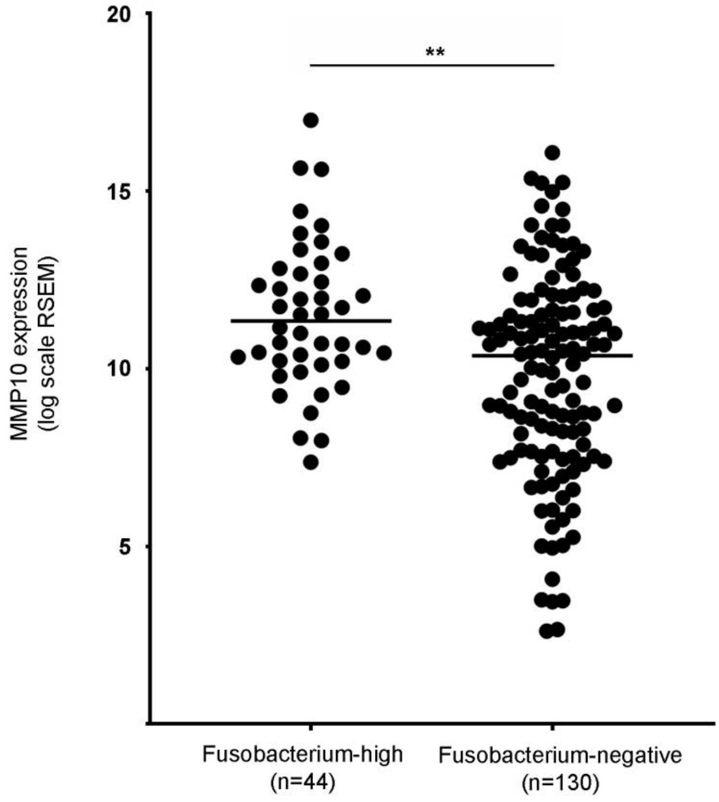

Supplementary Figure S7: RSEM expression of *MMP10* in *Fusobacterium*-high (n=44) and -negative (n=130) sub-group of TCGA-HNSC tumors. A comparison was performed using the Wilcoxon test.

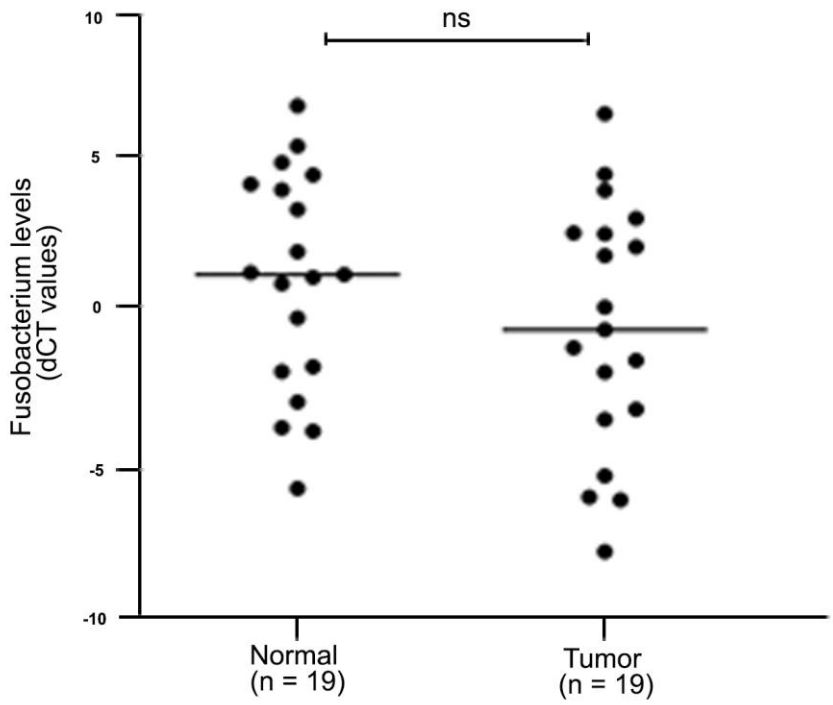

Supplementary Figure S8: qPCR-based comparison between levels of *Fusobacterium*

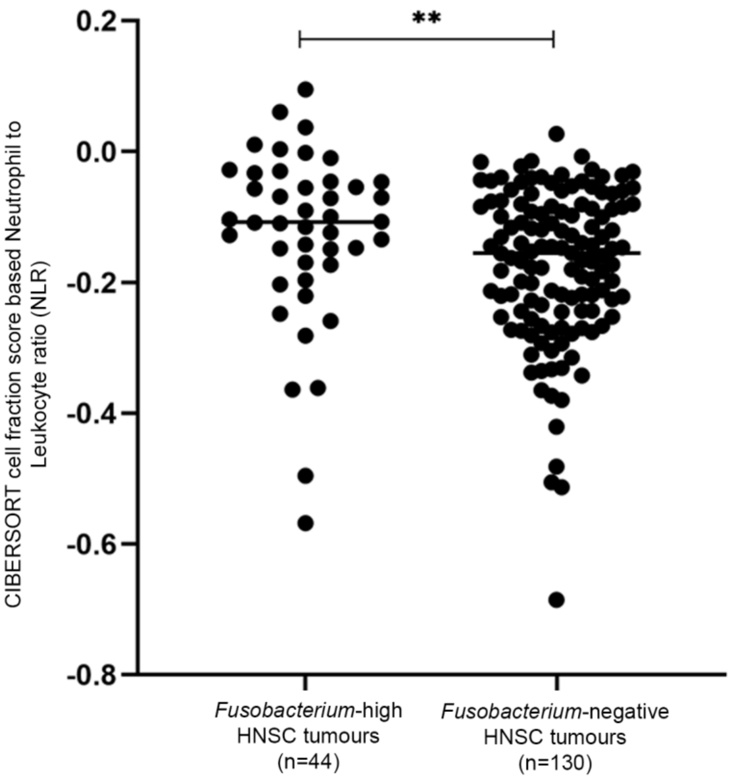

Supplementary Figure S9: Neutrophil-to-leucocyte ratio (NLR) computed using CIBERSORT fractions of individual tumor samples from Fusobacterium high (n=44) and negative (n=130) TCGA-HNSC sub-group.

Supplementary Table S1: List of primers used for *Fusobacterium nucleatum* , gene and miRNA validation

Factor name	Primer	Sequence
hsa-mir-451a	OAD2673_RT	GTCGTATCCAGTGCAGGGTCCGAGGTATTCGCACTGGA TACGACAACTCA
	OAD2599_forward	AAACCGTTACCATTACTGAGTT
hsa-mir-675	OAD2674_RT	GTCGTATCCAGTGCAGGGTCCGAGGTATTCGCACTGGA TACGACCACTGT
	OAD2600_forward	TGCGGAGAGGGCCCACAGTG
hsa-mir-486-1	OAD2675_RT	GTCGTATCCAGTGCAGGGTCCGAGGTATTCGCACTGGA TACGACCTCGGG
	OAD2603_forward	CTGTACTGAGCTGCCCCGAG
hsa-mir-1269b	OAD2676_RT	GTCGTATCCAGTGCAGGGTCCGAGGTATTCGCACTGGA TACGACCCAGTA
	OAD2604_forward	CTGGACTGAGCCATGCTACTGG
hsa-mir-9-2/9-3/9-1	OAD2677_RT	GTCGTATCCAGTGCAGGGTCCGAGGTATTCGCACTGGA TACGACTCATAC
	OAD2605_forward	TCTTTGGTTATCTAGCTGTATGA
hsa-mir-598	OAD2678_RT	GTCGTATCCAGTGCAGGGTCCGAGGTATTCGCACTGGA TACGACTGACGA
	OAD2204_forward	TACGTCATCGTTGTCATCGTCA
Universal reverse primer	OAD2688_Universal reverse	CCAGTGCAGGGTCCGAGGTA
RNU48/SNORD48	OAD2685_RNU48_forward	TTGAGTGTGTCGCTGATGCC
	OAD2686_RNU48 RT	GTCGTATCCAGTGCAGGGTCCGAGGTATTCGCACTGGA TACGAGGTCAGAGCG
IL6	OAD2254_IL6_forward	CAAATTCGGTACATCCTCGAC
	OAD2255_IL6_reverse	GCAAGTCTCCTCATTGAATCC
TLR4	OAD2581-TLR4_forward	ATATTGACAGGAAACCCCATCCA
	OAD2582-TLR4_reverse	AGAGAGATTGAGTAGGGGCATTT
IL1BETA	OAD2296_IL1BETA_forward	CGCAGGGACAGGATATGGAGCA
	OAD2297_IL1BETA_reverse	TTCAACAGCAGGACAGGTACA
IL8	OAD2707_IL8_forward	ATGACTTCCAAGCTGGCCGTGGCT
	OAD2708_IL8_reverse	TCTCAGCCCTCTTCAAAAACTTCTC
NFKB1	OAD2699_NFKB1_forward	GTGACAAAGTTCAGAAAGATGAC
	OAD2700-_NFKB1_reverse	TTGAAGACAATGGCAAATTG
Beta-actin	OAD69_forward	TCCCTGGAGAAGAGCTACGA
	OAD70_reverse	AGCACTGTGTTGGCGTACAG
Fusobacterium_16s	OAD1677_forward	AAGCGCGTCTAGGTGGTTATGT
	OAD1678_reverse	TGTAGTTCCGCTTACCTCTCCAG

Supplementary Table S2: In-house whole exome and transcriptome samples analysed using IPD

SR No	Tumor type	Data type	ADLABID	Tumor_Normal	Public ID
1	Cervical	Exome	AD0689	Normal	E-MTAB-9766
2	Cervical	Exome	AD0690	Tumor	E-MTAB-9766
3	Cervical	Exome	AD0691	Normal	E-MTAB-9766
4	Cervical	Exome	AD0701	Tumor	E-MTAB-9766
5	Cervical	Exome	AD0703	Normal	E-MTAB-9766
6	Cervical	Exome	AD0704	Normal	E-MTAB-9766
7	Cervical	Exome	AD0715	Tumor	E-MTAB-9766
8	Cervical	Exome	AD0716	Normal	E-MTAB-9766
9	Cervical	Exome	AD0717	Normal	E-MTAB-9766
10	Cervical	Exome	AD0700	Normal	E-MTAB-9766
11	Cervical	Exome	AD0705	Tumor	E-MTAB-9766
12	Cervical	Exome	AD0714	Normal	E-MTAB-9766
13	Cervical	Exome	AD0718	Tumor	E-MTAB-9766
14	Cervical	Exome	AD0726	Tumor	E-MTAB-9766
15	Cervical	Exome	AD0729	Tumor	E-MTAB-9766
16	Cervical	Exome	AD0693	Normal	E-MTAB-9766
17	Cervical	Exome	AD0694	Tumor	E-MTAB-9766
18	Cervical	Exome	AD0695	Normal	E-MTAB-9766
19	Cervical	Exome	AD0696	Tumor	E-MTAB-9766
20	Cervical	Exome	AD0706	Tumor	E-MTAB-9766
21	Cervical	Exome	AD0707	Normal	E-MTAB-9766
22	Cervical	Exome	AD0708	Tumor	E-MTAB-9766
23	Cervical	Exome	AD0709	Normal	E-MTAB-9766
24	Cervical	Exome	AD0710	Tumor	E-MTAB-9766
25	Cervical	Exome	AD0711	Normal	E-MTAB-9766
26	Cervical	Exome	AD0713	Tumor	E-MTAB-9766
27	Cervical	Exome	AD0719	Normal	E-MTAB-9766
28	Cervical	Exome	AD0722	Normal	E-MTAB-9766
29	Cervical	Exome	AD0723	Tumor	E-MTAB-9766
30	Cervical	Exome	AD0727	Tumor	E-MTAB-9766
31	Cervical	Exome	AD0728	Tumor	E-MTAB-9766
32	Cervical	Exome	AD0697	Normal	E-MTAB-9766
33	Cervical	Exome	AD0698	Normal	E-MTAB-9766
34	Cervical	Exome	AD0699	Normal	E-MTAB-9766
35	Cervical	Exome	AD0730	Tumor	E-MTAB-9766
36	Cervical	Exome	AD0732	Tumor	E-MTAB-9766
37	Cervical	Exome	AD0735	Tumor	E-MTAB-9766
38	Cervical	Exome	AD0749	Tumor	E-MTAB-11407
39	Cervical	Exome	AD0751	Tumor	E-MTAB-11407
40	Cervical	Exome	AD0753	Tumor	E-MTAB-11407
41	Cervical	Exome	AD0757	Tumor	E-MTAB-11407
42	Cervical	Exome	AD0762	Tumor	E-MTAB-11407

43	Cervical	Exome	AD0763	Tumor	E-MTAB-11407
44	Cervical	Exome	AD0764	Tumor	E-MTAB-11407
45	Cervical	Exome	AD0765	Tumor	E-MTAB-11407
46	Cervical	Exome	AD0766	Tumor	E-MTAB-11407
47	Cervical	Exome	AD0767	Tumor	E-MTAB-11407
48	Cervical	Exome	AD0768	Tumor	E-MTAB-11407
49	Cervical	Exome	AD0769	Tumor	E-MTAB-11407
50	Cervical	Exome	AD0770	Tumor	E-MTAB-11407
51	Cervical	Exome	AD0791	Tumor	E-MTAB-11407
52	Cervical	Exome	AD0792	Tumor	E-MTAB-11407
53	Cervical	Exome	AD0793	Tumor	E-MTAB-11407
54	Cervical	Exome	AD0794	Tumor	E-MTAB-11407
55	Cervical	Exome	AD0794	Tumor	E-MTAB-11407
56	Cervical	Exome	AD0795	Tumor	E-MTAB-11407
57	Cervical	Exome	AD0796	Tumor	E-MTAB-11407
58	Cervical	Exome	AD0798	Tumor	E-MTAB-11407
59	Cervical	Exome	AD0799	Tumor	E-MTAB-11407
60	Cervical	Exome	AD0806	Tumor	E-MTAB-11407
61	Cervical	Exome	AD0807	Tumor	E-MTAB-11407
62	Colorectal	Exome	AD1888	Tumor	E-MTAB-11407
63	Colorectal	Exome	AD1889	Tumor	E-MTAB-11407
64	Colorectal	Exome	AD1890	Tumor	E-MTAB-11407
65	Colorectal	Exome	AD1900	Tumor	E-MTAB-11407
66	Colorectal	Exome	AD1901	Tumor	E-MTAB-11407
67	Colorectal	Exome	AD1902	Tumor	E-MTAB-11407
68	Colorectal	Exome	AD1913	Tumor	E-MTAB-11407
69	Colorectal	Exome	AD1914	Normal	E-MTAB-11407
70	Colorectal	Exome	AD1915	Normal	E-MTAB-11407
71	Colorectal	Exome	AD1899	Tumor	E-MTAB-11407
72	Colorectal	Exome	AD1903	Tumor	E-MTAB-11407
73	Colorectal	Exome	AD1907	Tumor	E-MTAB-11407
74	Colorectal	Exome	AD1912	Tumor	E-MTAB-11407
75	Colorectal	Exome	AD1916	Normal	E-MTAB-11407
76	Colorectal	Exome	AD1920	Normal	E-MTAB-11407
77	Colorectal	Exome	AD1925	Normal	E-MTAB-11407
78	Colorectal	Exome	AD1930	Normal	E-MTAB-11407
79	Colorectal	Exome	AD1934	Normal	E-MTAB-11407
80	Colorectal	Exome	AD1926	Normal	E-MTAB-11407
81	Colorectal	Exome	AD1927	Normal	E-MTAB-11407
82	Colorectal	Exome	AD1928	Normal	E-MTAB-11407
83	Colorectal	Exome	AD1929	Normal	E-MTAB-11407
84	Colorectal	Exome	AD1891	Tumor	E-MTAB-11407
85	Colorectal	Exome	AD1892	Tumor	E-MTAB-11407
86	Colorectal	Exome	AD1893	Tumor	E-MTAB-11407
87	Colorectal	Exome	AD1894	Tumor	E-MTAB-11407
88	Colorectal	Exome	AD1895	Tumor	E-MTAB-11407
89	Colorectal	Exome	AD1896	Tumor	E-MTAB-11407

90	Colorectal	Exome	AD1897	Tumor	E-MTAB-11407
91	Colorectal	Exome	AD1898	Tumor	E-MTAB-11407
92	Colorectal	Exome	AD1904	Tumor	E-MTAB-11407
93	Colorectal	Exome	AD1905	Tumor	E-MTAB-11407
94	Colorectal	Exome	AD1906	Tumor	E-MTAB-11407
95	Colorectal	Exome	AD1908	Tumor	E-MTAB-11407
96	Colorectal	Exome	AD1909	Tumor	E-MTAB-11407
97	Colorectal	Exome	AD1910	Tumor	E-MTAB-11407
98	Colorectal	Exome	AD1911	Tumor	E-MTAB-11407
99	Colorectal	Exome	AD1917	Normal	E-MTAB-11407
100	Colorectal	Exome	AD1918	Normal	E-MTAB-11407
101	Colorectal	Exome	AD1919	Normal	E-MTAB-11407
102	Colorectal	Exome	AD1921	Normal	E-MTAB-11407
103	Colorectal	Exome	AD1922	Normal	E-MTAB-11407
104	Colorectal	Exome	AD1923	Normal	E-MTAB-11407
105	Colorectal	Exome	AD1924	Normal	E-MTAB-11407
106	Colorectal	Exome	AD1931	Normal	E-MTAB-11407
107	Colorectal	Exome	AD1932	Normal	E-MTAB-11407
108	Colorectal	Exome	AD1933	Normal	E-MTAB-11407
109	Colorectal	Exome	AD1935	Normal	E-MTAB-11407
110	Colorectal	Exome	AD1936	Normal	E-MTAB-11407
111	Colorectal	Exome	AD1937	Normal	E-MTAB-11407
112	Colorectal	Exome	AD1938	Normal	E-MTAB-11407
113	Colorectal	Exome	AD1939	Normal	E-MTAB-11407
114	Colorectal	Exome	AD1952	Tumor	E-MTAB-11407
115	Colorectal	Exome	AD1953	Normal	E-MTAB-11407
116	Gall bladder	Exome	AD0440	Tumor	E-MTAB-6619
117	Gall bladder	Exome	AD0743	Tumor	E-MTAB-6619
118	Gall bladder	Exome	AD0437	Normal	E-MTAB-6619
119	Gall bladder	Exome	AD0438	Tumor	E-MTAB-6619
120	Gall bladder	Exome	AD0439	Normal	E-MTAB-6619
121	Gall bladder	Exome	AD0744	Tumor	E-MTAB-6619
122	Gall bladder	Exome	AD0745	Tumor	E-MTAB-6619
123	Gall bladder	Exome	AD0746	Normal	E-MTAB-6619
124	Gall bladder	Exome	AD0747	Tumor	E-MTAB-6619
125	Gall bladder	Exome	AD0477	Tumor	E-MTAB-6619
126	Gall bladder	Exome	AD0736	Tumor	E-MTAB-6619
127	Gall bladder	Exome	AD0738	Tumor	E-MTAB-6619
128	Gall bladder	Exome	AD0739	Tumor	E-MTAB-6619
129	Gall bladder	Exome	AD0740	Tumor	E-MTAB-6619
130	Gall bladder	Exome	AD0741	Tumor	E-MTAB-6619
131	Gall bladder	Exome	AD0742	Tumor	E-MTAB-6619
132	Gall bladder	Exome	AD0748	Tumor	E-MTAB-6619
133	Gall bladder	Exome	AD0750	Tumor	E-MTAB-6619
134	Gall bladder	Exome	AD0752	Normal	E-MTAB-6619
135	Gall bladder	Exome	AD0754	Normal	E-MTAB-6619
136	Gall bladder	Exome	AD0755	Normal	E-MTAB-6619

137	Gall bladder	Exome	AD0756	Normal	E-MTAB-6619
138	Gall bladder	Exome	AD0757	Normal	E-MTAB-6619
139	Gall bladder	Exome	AD0758	Tumor	E-MTAB-6619
140	Gall bladder	Exome	AD0759	Normal	E-MTAB-6619
141	Gall bladder	Exome	AD0761	Tumor	E-MTAB-6619
142	Oral	Exome	AD0495	Tumor	E-MTAB-4653
143	Oral	Exome	AD0500	Tumor	E-MTAB-4653
144	Oral	Exome	AD0501	Tumor	E-MTAB-4653
145	Oral	Exome	AD0485	Tumor	E-MTAB-4653
146	Oral	Exome	AD0486	Tumor	E-MTAB-4653
147	Oral	Exome	AD0487	Tumor	E-MTAB-4653
148	Oral	Exome	AD0490	Tumor	E-MTAB-4653
149	Oral	Exome	AD0492	Tumor	E-MTAB-4653
150	Oral	Exome	AD0502	Tumor	E-MTAB-4653
151	Oral	Exome	AD0503	Tumor	E-MTAB-4653
152	Oral	Exome	AD0507	Tumor	E-MTAB-4653
153	Oral	Exome	AD0515	Tumor	E-MTAB-4653
154	Oral	Exome	AD0516	Tumor	E-MTAB-4653
155	Oral	Exome	AD0721	Normal	E-MTAB-4653
156	Oral	Exome	AD0725	Normal	E-MTAB-4653
157	Oral	Exome	AD0731	Normal	E-MTAB-4653
158	Oral	Exome	AD0733	Normal	E-MTAB-4653
159	Oral	Exome	AD0771	Normal	E-MTAB-4653
160	Oral	Exome	AD0772	Normal	E-MTAB-4653
161	Oral	Exome	AD0773	Normal	E-MTAB-4653
162	Oral	Exome	AD0774	Normal	E-MTAB-4653
163	Oral	Exome	AD0775	Normal	E-MTAB-4653
164	Oral	Exome	AD0781	Normal	E-MTAB-4653
165	Oral	Exome	AD0782	Normal	E-MTAB-4653
166	Oral	Exome	AD0783	Normal	E-MTAB-4653
167	Oral	Exome	AD0784	Normal	E-MTAB-4653
168	Oral	Exome	AD0786	Normal	E-MTAB-4653
169	Oral	Exome	AD0787	Normal	E-MTAB-4653
170	Oral	Exome	AD0789	Normal	E-MTAB-4653
171	Oral	Exome	AD0479	Tumor	E-MTAB-4653
172	Oral	Exome	AD0480	Tumor	E-MTAB-4653
173	Oral	Exome	AD0481	Tumor	E-MTAB-4653
174	Oral	Exome	AD0483	Tumor	E-MTAB-4653
175	Oral	Exome	AD0720	Normal	E-MTAB-4653
176	Oral	Exome	AD0734	Normal	E-MTAB-4653
177	Oral	Exome	AD0737	Tumor	E-MTAB-4653
178	Oral	Exome	AD0739	Tumor	E-MTAB-4653
179	Oral	Exome	AD0529	Tumor	E-MTAB-4653
180	Oral	Exome	AD0532	Tumor	E-MTAB-4653
181	Oral	Exome	AD0533	Tumor	E-MTAB-4653
182	Oral	Exome	AD0777	Normal	E-MTAB-4653
183	Oral	Exome	AD0778	Normal	E-MTAB-4653

184	Oral	Exome	AD0742	Tumor	E-MTAB-4653
185	Oral	Exome	AD0744	Normal	E-MTAB-4653
186	Oral	Exome	AD0779	Normal	E-MTAB-4653
187	Oral	Exome	AD0780	Normal	E-MTAB-4653
188	Cervical	Transcriptome	AD0727	Tumor	E-MTAB-9281
189	Cervical	Transcriptome	AD1088	Tumor	E-MTAB-9281
190	Cervical	Transcriptome	AD1092	Tumor	E-MTAB-9281
191	Cervical	Transcriptome	AD1093	Tumor	E-MTAB-9281
192	Cervical	Transcriptome	AD1095	Tumor	E-MTAB-9281
193	Cervical	Transcriptome	AD1098	Tumor	E-MTAB-9281
194	Cervical	Transcriptome	AD1099	Tumor	E-MTAB-9281
195	Cervical	Transcriptome	AD1100	Tumor	E-MTAB-9281
196	Cervical	Transcriptome	AD1104	Tumor	E-MTAB-9281
197	Cervical	Transcriptome	AD1107	Tumor	E-MTAB-9281
198	Cervical	Transcriptome	AD1109	Tumor	E-MTAB-9281
199	Cervical	Transcriptome	AD1110	Tumor	E-MTAB-9281
200	Cervical	Transcriptome	AD1112	Tumor	E-MTAB-9281
201	Cervical	Transcriptome	AD1808	Tumor	E-MTAB-9281
202	Cervical	Transcriptome	AD1809	Tumor	E-MTAB-9281
203	Cervical	Transcriptome	AD1810	Tumor	E-MTAB-9281
204	Cervical	Transcriptome	AD1811	Tumor	E-MTAB-9281
205	Cervical	Transcriptome	AD1960	Tumor	E-MTAB-9281
206	Cervical	Transcriptome	AD1961	Tumor	E-MTAB-9281
207	Cervical	Transcriptome	AD1962	Tumor	E-MTAB-9281
208	Cervical	Transcriptome	AD1963	Tumor	E-MTAB-9281
209	Colorectal	Transcriptome	AD1888	Tumor	EGAS00001005970
210	Colorectal	Transcriptome	AD1889	Tumor	EGAS00001005970
211	Colorectal	Transcriptome	AD1890	Tumor	EGAS00001005970
212	Colorectal	Transcriptome	AD1891	Tumor	EGAS00001005970
213	Colorectal	Transcriptome	AD1892	Tumor	EGAS00001005970
214	Colorectal	Transcriptome	AD1894	Tumor	EGAS00001005970
215	Colorectal	Transcriptome	AD1895	Tumor	EGAS00001005970
216	Colorectal	Transcriptome	AD1897	Tumor	EGAS00001005970
217	Colorectal	Transcriptome	AD1899	Tumor	EGAS00001005970
218	Colorectal	Transcriptome	AD1900	Tumor	EGAS00001005970
219	Colorectal	Transcriptome	AD1898	Tumor	EGAS00001005970
220	Colorectal	Transcriptome	AD1903	Tumor	EGAS00001005970
221	Colorectal	Transcriptome	AD1904	Tumor	EGAS00001005970
222	Colorectal	Transcriptome	AD1906	Tumor	EGAS00001005970
223	Colorectal	Transcriptome	AD1907	Tumor	EGAS00001005970
224	Colorectal	Transcriptome	AD1908	Tumor	EGAS00001005970
225	Colorectal	Transcriptome	AD1909	Tumor	EGAS00001005970
226	Colorectal	Transcriptome	AD1910	Tumor	EGAS00001005970
227	Colorectal	Transcriptome	AD1911	Tumor	EGAS00001005970
228	Colorectal	Transcriptome	AD1912	Tumor	EGAS00001005970
229	Colorectal	Transcriptome	AD1940	Tumor	EGAS00001005970
230	Colorectal	Transcriptome	AD1941	Tumor	EGAS00001005970

231	Colorectal	Transcriptome	AD1942	Tumor	EGAS00001005970
232	Colorectal	Transcriptome	AD1943	Tumor	EGAS00001005970
233	Colorectal	Transcriptome	AD1944	Tumor	EGAS00001005970
234	Colorectal	Transcriptome	AD1945	Tumor	EGAS00001005970
235	Colorectal	Transcriptome	AD1946	Tumor	EGAS00001005970
236	Colorectal	Transcriptome	AD1947	Tumor	EGAS00001005970
237	Colorectal	Transcriptome	AD1948	Tumor	EGAS00001005970
238	Colorectal	Transcriptome	AD1949	Tumor	EGAS00001005970
239	Colorectal	Transcriptome	AD1950	Tumor	EGAS00001005970
240	Colorectal	Transcriptome	AD1951	Tumor	EGAS00001005970
241	Colorectal	Transcriptome	AD1905	Tumor	EGAS00001005970
242	Oral	Transcriptome	AD0487	Tumor	E-MTAB-4654
243	Oral	Transcriptome	AD0487	Tumor	E-MTAB-4654
244	Oral	Transcriptome	AD0493	Tumor	E-MTAB-4654
245	Oral	Transcriptome	AD0497	Tumor	E-MTAB-4654
246	Oral	Transcriptome	AD0511	Tumor	E-MTAB-4654
247	Oral	Transcriptome	AD0516	Tumor	E-MTAB-4654
248	Oral	Transcriptome	AD0517	Tumor	E-MTAB-4654
249	Oral	Transcriptome	AD0719	Normal	E-MTAB-4654
250	Oral	Transcriptome	AD0723	Normal	E-MTAB-4654
251	Oral	Transcriptome	AD0731	Normal	E-MTAB-4654
252	Oral	Transcriptome	AD0488	Tumor	E-MTAB-4654
253	Oral	Transcriptome	AD0489	Tumor	E-MTAB-4654
254	Oral	Transcriptome	AD0724	Normal	E-MTAB-4654
255	Oral	Transcriptome	AD0501	Tumor	E-MTAB-4654
256	Oral	Transcriptome	AD0507	Tumor	E-MTAB-4654
257	Breast	Transcriptome	1222249	Tumor	E-MTAB-11412
258	Breast	Transcriptome	1222004	Tumor	E-MTAB-11412
259	Breast	Transcriptome	1222108	Tumor	E-MTAB-11412
260	Breast	Transcriptome	1222046	Tumor	E-MTAB-11412
261	Breast	Transcriptome	1221945	Tumor	E-MTAB-11412
262	Breast	Transcriptome	1222332	Tumor	E-MTAB-11412
263	Breast	Transcriptome	1222265	Tumor	E-MTAB-11412
264	Breast	Transcriptome	1222058	Tumor	E-MTAB-11412
265	Breast	Transcriptome	1222149	Tumor	E-MTAB-11412
266	Breast	Transcriptome	1222362	Tumor	E-MTAB-11412
267	Breast	Transcriptome	1222089	Tumor	E-MTAB-11412
268	Breast	Transcriptome	1222013	Tumor	E-MTAB-11412
269	Breast	Transcriptome	1222298	Tumor	E-MTAB-11412
270	Breast	Transcriptome	1222382	Tumor	E-MTAB-11412
271	Breast	Transcriptome	1222418	Tumor	E-MTAB-11412
272	Breast	Transcriptome	1222460	Tumor	E-MTAB-11412
273	Breast	Transcriptome	1222568	Tumor	E-MTAB-11412
274	Breast	Transcriptome	1222805	Tumor	E-MTAB-11412
275	Breast	Transcriptome	1222616	Tumor	E-MTAB-11412
276	Breast	Transcriptome	1222708	Tumor	E-MTAB-11412
277	Breast	Transcriptome	1222817	Tumor	E-MTAB-11412

278	Breast	Transcriptome	1222867	Tumor	E-MTAB-11412
279	Breast	Transcriptome	1223273	Tumor	E-MTAB-11412
280	Breast	Transcriptome	1223283	Tumor	E-MTAB-11412
281	Breast	Transcriptome	1223501	Tumor	E-MTAB-11412
282	Breast	Transcriptome	1223508	Tumor	E-MTAB-11412
283	Breast	Transcriptome	1223547	Tumor	E-MTAB-11412
284	Breast	Transcriptome	1223607	Tumor	E-MTAB-11412
285	Breast	Transcriptome	1223571	Tumor	E-MTAB-11412
286	Breast	Transcriptome	1223626	Tumor	E-MTAB-11412
287	Breast	Transcriptome	1324236	Tumor	E-MTAB-11412
288	Breast	Transcriptome	1324242	Tumor	E-MTAB-11412
289	Breast	Transcriptome	1324248	Tumor	E-MTAB-11412
290	Breast	Transcriptome	1324338	Tumor	E-MTAB-11412
291	Breast	Transcriptome	1324388	Tumor	E-MTAB-11412
292	Breast	Transcriptome	1324148	Tumor	E-MTAB-11412
293	Breast	Transcriptome	1324404	Tumor	E-MTAB-11412
294	Breast	Transcriptome	1324405	Tumor	E-MTAB-11412
295	Breast	Transcriptome	1324408	Tumor	E-MTAB-11412
296	Breast	Transcriptome	1324508	Tumor	E-MTAB-11412
297	Breast	Transcriptome	T1A	Tumor	E-MTAB-11412
298	Lung	Exome	AD2320	Tumor	E-MTAB-8801
299	Lung	Exome	AD2340	Tumor	E-MTAB-8801
300	Lung	Exome	AD2385	Tumor	E-MTAB-8801
301	Lung	Exome	AD2315	Tumor	E-MTAB-8801
302	Lung	Exome	AD2356	Tumor	E-MTAB-8801
303	Lung	Exome	AD2351	Tumor	E-MTAB-8801
304	Lung	Exome	AD2341	Tumor	E-MTAB-8801
305	Lung	Exome	AD2367	Tumor	E-MTAB-8801
306	Lung	Exome	AD2375	Tumor	E-MTAB-8801
307	Lung	Exome	AD2349	Tumor	E-MTAB-8801
308	Lung	Exome	AD2313	Tumor	E-MTAB-8801
309	Lung	Exome	AD2378	Tumor	E-MTAB-8801
310	Lung	Exome	AD2327	Tumor	E-MTAB-8801
311	Lung	Exome	AD2382	Tumor	E-MTAB-8801
312	Lung	Exome	AD2374	Tumor	E-MTAB-8801
313	Lung	Exome	AD2352	Tumor	E-MTAB-8801
314	Lung	Exome	AD2398	Tumor	E-MTAB-8801
315	Lung	Exome	AD2364	Tumor	E-MTAB-8801
316	Lung	Exome	AD2373	Tumor	E-MTAB-8801
317	Lung	Exome	AD2371	Tumor	E-MTAB-8801
318	Lung	Exome	AD1783	Tumor	E-MTAB-11404
319	Lung	Exome	AD1784	Tumor	E-MTAB-11404
320	Lung	Exome	AD1785	Tumor	E-MTAB-11404
321	Lung	Exome	AD1786	Tumor	E-MTAB-11404
322	Lung	Exome	AD1787	Tumor	E-MTAB-11404
323	Lung	Exome	AD1788	Tumor	E-MTAB-11404
324	Lung	Exome	AD1794	Tumor	E-MTAB-11404

325	Lung	Exome	AD1797	Tumor	E-MTAB-11404
326	Lung	Exome	AD1800	Tumor	E-MTAB-11404
327	Lung	Exome	AD1801	Tumor	E-MTAB-11404
328	Lung	Exome	AD1802	Tumor	E-MTAB-11404
329	Lung	Exome	AD1805	Tumor	E-MTAB-11404
330	Lung	Exome	AD1826	Tumor	E-MTAB-11404
331	Lung	Exome	AD1828	Tumor	E-MTAB-11404
332	Lung	Exome	AD1829	Tumor	E-MTAB-11404
333	Lung	Exome	AD2084	Tumor	E-MTAB-11404
334	Lung	Exome	AD2976	Tumor	E-MTAB-11404
335	Lung	Exome	AD2978	Tumor	E-MTAB-11404
336	Lung	Exome	AD2980	Tumor	E-MTAB-11404
337	Lung	Exome	AD2982	Tumor	E-MTAB-11404
338	Lung	Exome	AD2984	Tumor	E-MTAB-11404
339	Lung	Exome	AD2986	Tumor	E-MTAB-11404
340	Lung	Exome	AD2998	Tumor	E-MTAB-11404
341	Lung	Exome	AD3004	Tumor	E-MTAB-11404
342	Lung	Exome	AD3010	Tumor	E-MTAB-11404
343	Lung	Exome	AD3012	Tumor	E-MTAB-11404
344	Lung	Exome	AD3014	Tumor	E-MTAB-11404
345	Lung	Exome	AD3020	Tumor	E-MTAB-11404
346	Lung	Exome	AD3032	Tumor	E-MTAB-11404
347	Lung	Exome	AD3036	Tumor	E-MTAB-11404
348	Lung	Exome	AD3038	Tumor	E-MTAB-11404
349	Lung	Exome	AD3044	Tumor	E-MTAB-11404

Supplementary Table S3: Differentially expressed genes in *Fusobacterium nucleatum* high versus low sub-group of TCGA-HNSC samples (n=174)

Gene	Mean Expression	log2FoldChange	pvalue	padj
KRT2	822.2406713	3.552006136	2.8E-12	1E-09
SPINK7	1818.93597	2.536100321	3.1E-07	1E-05
SPRR2G	10664.51368	2.425870385	2.2E-09	3E-07
PRR9	963.4314131	2.422561924	4.8E-08	3E-06
LCE3E	2150.810726	2.410605651	5.9E-09	6E-07
LCE3A	543.0745945	2.310367429	7.7E-07	3E-05
WFDC12	1073.926269	2.279500117	6.7E-08	4E-06
LINC01527	495.398701	2.213597707	6.7E-10	1E-07
C1orf68	184.0517827	2.175431643	2.5E-07	1E-05
LCE3D	6637.495038	2.157464672	2.3E-07	1E-05
SPRR2B	3840.032558	2.121639656	7.1E-07	3E-05
CLDN17	199.9052413	2.072876391	2.5E-05	0.0004
IL1F10	77.35217701	2.063285877	6.4E-09	7E-07
VSIG8	459.5688044	2.059768754	3.8E-07	2E-05
SPINK6	887.5498885	1.992456422	3.2E-05	0.0005
KRT1	50192.21418	1.932593509	5E-05	0.0007
KPRP	1370.593184	1.925819508	9.3E-06	0.0002
NTSR1	128.738822	1.911823208	1E-05	0.0002
FAM25A	1393.604566	1.901484611	1.3E-06	4E-05
DSC1	2059.016639	1.860023933	7.7E-06	0.0002
CNFN	15169.56728	1.854249592	1E-08	1E-06
SPRR4	717.9335864	1.853763298	6.8E-07	3E-05
CRCT1	3589.092005	1.850251647	9.6E-07	3E-05
TGM3	9338.340964	1.843472378	6.9E-05	0.0009
SLURP1	2396.772897	1.842120737	1.8E-05	0.0003
KRT75	5257.016098	1.823984011	1E-07	6E-06
PTGS2	3885.15393	1.812271754	1.5E-08	1E-06
SPRR2F	2209.004531	1.811810016	1.5E-05	0.0003
CDSN	475.7172287	1.784899959	4.1E-05	0.0006
SPRR2E	29064.5585	1.728443313	7E-06	0.0002
ALOX12B	1720.167191	1.72336571	2.5E-07	1E-05
PLA2G4D	693.610442	1.717319685	1.1E-05	0.0002
LYNX1	8567.06159	1.715669302	1.5E-08	1E-06
SPRR2C	627.2705638	1.695968396	1.8E-06	5E-05
GSDMA	1136.098449	1.674142022	9.6E-08	6E-06
PSORS1C2	358.0616376	1.662203281	1.4E-05	0.0003
RNF222	370.5068636	1.660133179	1.1E-07	6E-06
CSF3	445.707547	1.634329756	2.2E-06	6E-05
TREX2	373.7006509	1.610529385	4.8E-08	3E-06

FLG2	676.10277	1.607788991	0.00456	0.022
BPIFC	262.4323879	1.599610867	2.4E-05	0.0004
IL1B	2169.643965	1.590326946	4.9E-10	8E-08
KRTDAP	21360.92234	1.570327417	9.4E-06	0.0002
PLA2G2F	398.5910759	1.567447897	3.3E-05	0.0005
S100A12	1481.867398	1.563442576	2.9E-07	1E-05
RPE65	226.5395897	1.556440842	0.00031	0.003
DSG1	18965.36827	1.553163883	4.9E-05	0.0007
KLK5	7689.175922	1.54934792	6.5E-06	0.0002
IL36RN	4959.842034	1.54766801	1.4E-07	8E-06
EFR3B	261.5643764	1.541985118	1.5E-09	2E-07
CSF2	249.4602767	1.536813667	4.3E-05	0.0006
LCE1F	160.8928019	1.535731218	0.00053	0.0045
PLA2G4E	3970.743748	1.535132072	4.5E-07	2E-05
PRSS3	953.4348148	1.534766995	2.6E-05	0.0004
11-599B1[illegible]	147.6609638	1.528931178	0.00021	0.0022
ELAVL2	213.5148275	1.525011156	9.3E-06	0.0002
MT1L	244.595191	1.515366034	5.9E-07	2E-05
KRT6C	105355.2195	1.50634775	0.00013	0.0015
ARG1	192.8933654	1.505936799	0.00066	0.0053
SERPINB2	4575.785548	1.500350752	6E-06	0.0001
KRT16P5	201.732764	1.495499463	5.7E-06	0.0001
SPRR2D	24875.62591	1.487650836	1.7E-06	5E-05
LY6G6C	1422.861637	1.485848871	8.4E-06	0.0002
LINC0070[illegible]	360.8709849	1.485788508	1.1E-05	0.0002
GDNF	133.286282	1.483818148	1.1E-06	4E-05
CYSRT1	1802.138658	1.474454997	1.5E-06	5E-05
C1orf204	112.1543661	1.468790867	1.2E-10	3E-08
ACER1	111.7694395	1.468211708	0.0003	0.0029
HAL	368.2106676	1.444114374	0.00052	0.0044
HES5	70.47031335	1.440092063	7.5E-07	3E-05
ALOXE3	861.9670022	1.437745688	1.5E-08	1E-06
CXCL3	223.7957578	1.436111547	7.4E-07	3E-05
SPRR2A	32691.93914	1.432200497	3.3E-05	0.0005
KRT3	142.688409	1.422430578	0.00027	0.0027
IL24	1296.532503	1.420688356	1E-05	0.0002
SPRR1B	72215.86398	1.418504207	1.2E-06	4E-05
SCNN1D	845.785151	1.416726927	3.5E-07	2E-05
KCNS1	155.9428839	1.415331242	0.00059	0.0048
FOLR3	203.8138506	1.405619642	0.00015	0.0017
NRG1	3136.006672	1.405445788	1E-08	1E-06
KRT16P1	376.472612	1.404920958	3.3E-05	0.0005
LYPD5	2780.008479	1.394945029	4.7E-07	2E-05
P4-694A7[illegible]	136.2020045	1.392161558	0.00033	0.0031

CSTA	32258.96158	1.390598705	5.2E-08	4E-06
LGALS9C	268.9358645	1.384109877	6E-06	0.0001
CXCR1	135.1939374	1.370113122	3.6E-06	1E-04
CLIC3	2301.729795	1.369606287	7.6E-07	3E-05
FLG	1648.569509	1.36176792	0.00067	0.0053
KLK8	3421.528726	1.359984976	8.5E-07	3E-05
CASP14	5438.776074	1.357090168	0.00142	0.0093
TGM1	16834.23263	1.350827928	9.7E-06	0.0002
PAPL	536.0264015	1.345386812	2.1E-05	0.0004
SPRR1A	27924.07396	1.338913935	0.00018	0.002
SDR9C7	1307.925208	1.337609287	2.8E-05	0.0005
KHDC1L	229.8511193	1.330383069	0.00016	0.0018
PNPLA1	163.2369685	1.329416606	1E-05	0.0002
RNASE7	1763.287284	1.326705944	2.1E-06	6E-05
KRT10	50199.89191	1.323007527	0.00058	0.0048
DEC1	129.0338415	1.315907915	3.3E-05	0.0005
SBSN	48368.36601	1.31042615	0.00017	0.0019
HEPHL1	6749.125486	1.309166424	0.00011	0.0013
IL36G	2827.868516	1.296739905	2E-06	6E-05
TC-490G23	80.51171228	1.295186116	0.00012	0.0014
FCGR3B	139.9160337	1.294325622	6.1E-05	0.0009
D-2619J13	199.8328016	1.29380243	3.1E-06	9E-05
ENDOU	588.5236905	1.287403048	0.00058	0.0048
IL1A	2892.99031	1.279826265	1.2E-05	0.0002
CDH16	318.0565456	1.277184282	0.00039	0.0035
PI3	99244.1041	1.277026659	0.00067	0.0053
KRT78	3231.029249	1.270658227	0.00104	0.0074
LINC01127	206.9805928	1.270602717	8.3E-05	0.0011
AC115522.	134.9578742	1.264810346	4.7E-06	0.0001
APLN	679.5299427	1.26297446	7.7E-11	2E-08
KRT16P2	1572.754038	1.25994378	0.00073	0.0057
PAEP	114.0228981	1.257081579	0.00037	0.0034
PRSS27	1844.177915	1.251036694	4.5E-05	0.0007
APOBEC3A	1869.824098	1.246723082	1.5E-05	0.0003
KRT6B	203554.6911	1.24400791	4E-06	0.0001
HOPX	4514.853146	1.240208621	1.9E-06	6E-05
KLK12	2048.950424	1.240179639	0.0017	0.0105
ALOX15B	1369.535197	1.239931319	0.00023	0.0024
LINC01322	130.3571997	1.234304392	0.00104	0.0074
ESYT3	215.1812265	1.232308598	4.5E-07	2E-05
GDPD3	552.2694556	1.226627014	8.4E-06	0.0002
UNC93A	143.7161494	1.211456827	0.00026	0.0026
PAQR5	1312.794312	1.208860032	3.3E-07	1E-05
SULT2B1	4700.372078	1.208345707	9.9E-06	0.0002

S100A8	161143.3662	1.206779678	7.1E-05	0.001
CYP26B1	1829.20409	1.204454537	4.7E-08	3E-06
S100A7	46758.25414	1.203598813	0.00049	0.0042
KLK14	1718.444548	1.200953296	0.00563	0.0259
KLK6	5383.689348	1.19775047	0.00027	0.0027
IVL	18631.25454	1.195858514	1.5E-05	0.0003
CDA	2270.043696	1.186932176	1.8E-05	0.0003
IL6	678.0444261	1.186674383	0.00028	0.0028
LGALSL	3797.337725	1.185091579	4E-10	7E-08
G0S2	848.5998656	1.180973963	2.2E-05	0.0004
AJAP1	687.7112352	1.175457406	0.00041	0.0037
ATG9B	406.0612679	1.170235045	4.9E-06	0.0001
PNLIPRP3	803.7742314	1.166815565	0.00394	0.0198
KLK7	7669.539619	1.16667597	0.00028	0.0028
SLC38A4	307.3810719	1.166393667	6.2E-05	0.0009
IGFL2	713.993241	1.162130704	0.00027	0.0027
IGFL1	2681.298641	1.161616832	0.0015	0.0097
CXCL8	6710.10532	1.157511607	0.0003	0.0029
KLK10	23385.32206	1.155861978	4.9E-05	0.0007
NGF	139.9135239	1.155698271	5.3E-05	0.0008
FST	7423.796041	1.15504775	1.3E-06	4E-05
FGFBP1	21348.26391	1.148272132	2.3E-06	7E-05
ASPRV1	3481.846419	1.142244593	0.00569	0.0261
S100A7A	7123.654646	1.140064531	0.00289	0.0156
F3	12177.40003	1.135311575	8.1E-06	0.0002
D-2555C1(	126.8926399	1.127572448	1E-07	6E-06
C1orf177	71.15127217	1.127178745	2.1E-05	0.0004
SERPINB7	1390.807929	1.11893597	0.00021	0.0022
KLK13	5280.775418	1.109140589	0.00198	0.0119
NLRP10	92.25842758	1.107952062	0.00568	0.0261
WFDC5	1175.351083	1.104698956	0.00014	0.0016
RNF223	359.903292	1.100665045	1.6E-06	5E-05
RDH12	898.0530844	1.100013462	0.00129	0.0087
KRT79	377.7692774	1.099943979	0.00902	0.0366
EREG	2662.094468	1.0968872	0.00244	0.0138
SLC6A2	450.9746122	1.092289875	0.00095	0.0069
HMGA2	2609.076495	1.091694114	0.00031	0.003
LCE1C	146.070628	1.081432067	0.0073	0.0313
MICALCL	503.4107306	1.077349501	3.3E-06	9E-05
ELOVL4	849.6095602	1.07172937	6.2E-05	0.0009
KRT16P6	4917.852828	1.057490794	0.00712	0.0307
MMP10	13177.75579	1.051007493	0.00567	0.0261
MYZAP	92.11984881	1.050529841	2.8E-05	0.0005
MYO7B	228.7149105	1.050059986	0.00248	0.0139

USP2	602.5978532	1.049928527	1.1E-05	0.0002
P11-21B23	80.89593959	1.048012538	0.00069	0.0054
RNF39	939.8801546	1.046070872	1.9E-06	6E-05
CXCL5	322.3934346	1.044568831	0.00106	0.0075
TPPP3	3796.021639	1.044178445	2.7E-05	0.0005
TRPV3	918.5880419	1.043342141	1.3E-06	4E-05
KRT80	7813.203001	1.040446089	5.8E-05	0.0008
DLX2	156.5788209	1.038743188	0.00014	0.0016
DHRS9	837.5854526	1.030851253	0.00035	0.0033
MT1E	3956.298448	1.02938544	0.00032	0.0031
ADRB2	1110.889606	1.028589981	3.5E-05	0.0005
FAM83C	3164.321626	1.02738093	5.6E-05	0.0008
RGS20	667.1443981	1.026832862	8.5E-07	3E-05
CARD18	203.0670092	1.02542346	0.00586	0.0266
SH2D5	973.4768846	1.023965727	6.8E-05	0.0009
IRLET7BH	185.6796712	1.022242686	3E-07	1E-05
EPGN	903.0625984	1.019831309	0.00293	0.0158
CCL3L3	117.15968	1.017441837	0.0002	0.0021
AIF1L	1527.806181	1.016290643	9E-06	0.0002
SLPI	36571.7471	1.013548422	0.0007	0.0055
HSPA2	6269.395069	1.012577044	6.7E-06	0.0002
RUNDC3A	260.8803931	1.010133366	2.3E-05	0.0004
DHRS1	2718.011325	1.010128414	1.3E-09	2E-07
DMKN	24883.8527	1.010108143	0.00029	0.0029
SLC5A1	1028.157471	1.008778183	0.00137	0.0091
RHOD	6570.175144	1.007563582	2.6E-07	1E-05
TMEM40	2777.924433	1.005133398	4E-07	2E-05
LETM2	301.1780193	1.001811765	8.4E-06	0.0002
CSTB	58720.38119	1.000235206	2.3E-06	7E-05
DLX3	897.8956715	0.995676209	9.7E-06	0.0002
SLC39A2	571.3258648	0.994314697	0.00029	0.0028
P11-554I8	158.2295865	0.993389843	0.00052	0.0044
LIPK	121.1444444	0.992080531	0.0034	0.0177
KIF17	120.5687286	0.989519005	3.5E-08	3E-06
AREG	4480.831684	0.98946096	0.0002	0.0021
IL1RL1	300.0189887	0.989235353	0.00153	0.0098
IL11	516.2645803	0.98819763	5E-05	0.0007
ANXA1	85923.0269	0.986459224	2.1E-07	1E-05
11-465B2	144.0966222	0.984836977	2.8E-07	1E-05
KRT17	523388.0624	0.984694614	5.8E-06	0.0001
RHCG	24035.3797	0.981754567	0.00753	0.0322
KRT16P4	154.0990334	0.978856664	0.00046	0.004
SLC10A6	528.3031326	0.976505414	5.1E-05	0.0007
CCL20	1138.128029	0.976453159	0.00405	0.0202

ARHGAP40	247.6417161	0.975657914	0.00125	0.0085
LINC00704	87.59321635	0.975620203	0.00036	0.0034
SLC6A14	1905.652293	0.97193023	0.00861	0.0354
CASP1P2	84.86352073	0.966814087	8.1E-05	0.0011
SPNS2	1494.35137	0.965126312	3.2E-05	0.0005
GJB2	66607.56748	0.964573992	8.2E-05	0.0011
CAV1	23603.27793	0.964470869	1.3E-06	4E-05
RIMS3	723.2214762	0.962489672	1.5E-05	0.0003
HIST1H3G	81.94592347	0.960597574	0.00016	0.0018
AM83A-AS	468.146335	0.958958149	0.00011	0.0014
SH3D21	686.1044431	0.957151309	1.6E-08	1E-06
CYP27B1	674.9111792	0.955874954	3.6E-05	0.0006
GJB6	19985.78464	0.955788858	0.00016	0.0018
CD177	1052.161225	0.954856525	0.01212	0.0456
P3H2	6994.356448	0.947859907	0.00031	0.003
ANK1	609.6549774	0.94771843	0.00115	0.0079
TUBB2A	4228.535587	0.944539704	1.9E-06	6E-05
C10orf99	5626.957468	0.943298502	0.00194	0.0117
MEFV	178.0818938	0.940735962	0.00013	0.0015
SPTLC3	890.1753898	0.940380639	0.00017	0.0019
ABCA12	3125.052184	0.93707975	0.00013	0.0016
IFFO2	9044.601456	0.936096869	1.6E-07	8E-06
FAM46B	3746.072767	0.934953015	0.00058	0.0048
GPR110	755.8213388	0.931309859	0.00912	0.0369
UPP1	7789.311981	0.929939692	2E-05	0.0004
TMEM86A	702.4976769	0.926637178	1.4E-05	0.0003
FFAR2	176.2372471	0.924163711	0.00046	0.004
ARL14	171.5744356	0.923020722	0.00615	0.0276
P11-268J15	161.5455777	0.918900588	1.8E-06	5E-05
NAV3	386.680815	0.913314386	0.00011	0.0013
LIPM	114.7040837	0.912891414	0.00073	0.0057
PTGS1	5082.569902	0.912203305	8.6E-05	0.0011
KRT14	857674.8613	0.909911661	0.00049	0.0042
MASP1	506.515313	0.906374962	0.00955	0.0382
LIPE-AS1	144.2129525	0.906219952	5.7E-05	0.0008
ADAMTSL4	1872.294942	0.902379704	1.7E-05	0.0003
IL1R2	1787.660481	0.901116349	0.00144	0.0094
SPINK5	11647.46053	0.898866451	0.00501	0.0236
TMEM45B	1508.920813	0.898429928	0.00197	0.0118
RAET1E	747.0428192	0.89577708	0.00136	0.009
LIPG	846.0608001	0.895719653	0.00133	0.0089
NFE2	75.43089781	0.891469192	0.00039	0.0036
TUBA4A	22674.82252	0.889102889	6.2E-07	2.4E-05
CRABP2	12708.24374	0.885568848	0.00017	0.00188

NCCRP1	5913.934723	0.87864887	0.0039	0.01963
S100A9	239977.5022	0.877639432	0.001531	0.0098
ACKR2	150.1217906	0.870432442	0.000432	0.00385
MFAP5	2219.007916	0.86977324	0.004958	0.02345
CHAC1	428.2387077	0.869455818	6.41E-06	0.00015
MATN3	218.5947355	0.86859314	0.00059	0.00482
FABP5P7	529.1642627	0.86841294	0.005793	0.02644
SLC28A3	1098.343199	0.867827442	9.46E-05	0.00119
THEM5	566.3331434	0.867759206	0.002561	0.01431
PLCXD1	1338.544641	0.866169278	9.61E-09	9.4E-07
MROH6	1744.506927	0.864225856	7.78E-06	0.00017
ECM1	10772.38771	0.859939805	4.48E-05	0.00067
HIST1H2B(	173.0525134	0.857444394	6.24E-05	0.00087
SCEL	3560.376798	0.852998639	0.005749	0.02629
AC108142	117.1901531	0.852973661	0.001038	0.0074
GPR39	285.5971315	0.851617122	0.000914	0.00669
RP11-783K	290.9213309	0.851340472	3.65E-05	0.00057
CPA4	2592.371404	0.851298979	0.002771	0.01517
PI15	595.7913993	0.849939788	0.000185	0.002
EPS8L1	3318.830775	0.849579831	7.19E-05	0.00097
INPP4B	1335.192794	0.843183038	1.74E-05	0.00032
COL2A1	125.3692327	0.84180236	0.005733	0.02624
RP11-93B1	162.9692998	0.841022138	4.7E-05	0.0007
HLA-G	663.4049051	0.837914068	0.003764	0.01911
FABP5P1	92.92235793	0.832608056	0.003779	0.01915
PGLYRP4	1104.263472	0.831385487	0.002174	0.01274
ANKRD22	1175.50016	0.831167988	0.000173	0.0019
FABP5	35857.99373	0.829467689	0.001531	0.0098
KCNK7	497.701614	0.829320462	0.003136	0.01662
ZBED2	1407.720737	0.829077668	0.000289	0.00285
TTC39A	614.0557685	0.826292033	0.000152	0.00174
SLCO4A1	1065.584081	0.826016122	5.42E-05	0.00077
HSPA12A	677.9790441	0.825367583	1.26E-05	0.00025
ALS2CL	2794.710189	0.823573677	1.15E-05	0.00023
CXCR2	255.1776397	0.823372608	0.002921	0.01573
LAMC2	94052.38539	0.8233653	0.000591	0.00483
RRAD	3527.071674	0.822329501	0.011534	0.04401
DSC2	34486.56458	0.821342695	0.000167	0.00186
CXCL2	387.670173	0.821220034	0.004559	0.02202
KCNH3	83.45411859	0.820657295	0.001459	0.00949
TSPAN2	245.9951034	0.820647996	0.00174	0.0107
GOLGA7B	2047.714352	0.818878785	0.000344	0.00323
ADAP2	859.1816202	0.815290585	1.51E-06	4.7E-05
SCG5	261.0549962	0.814283495	0.001476	0.00958
CD164L2	198.1683758	0.814116366	0.00096	0.00693
IL22RA1	1098.475646	0.810664934	9.98E-07	3.4E-05
VSIG10L	2209.425112	0.809724984	0.004523	0.0219

DUSP14	3776.79521	0.80972497	1.34E-10	2.8E-08
FRMD5	105.8634651	0.809451924	0.00591	0.02679
MISP	1641.194008	0.809448728	0.002102	0.01242
CARHSP1	7469.257785	0.805660962	4.44E-08	3.2E-06
SERPINB1	16073.01587	0.802451518	3.11E-05	0.0005
S100A14	34819.17731	0.802385512	0.000108	0.00133
PLEK2	4324.468814	0.800596601	1.7E-05	0.00032
PLCD3	3404.321669	0.799733339	4.07E-07	1.7E-05
POLR3G	460.8071764	0.798198601	4.37E-07	1.8E-05
STEAP4	2379.370201	0.797393703	0.008476	0.03494
MPZL3	1839.501791	0.796542438	1.09E-07	6.2E-06
FLRT3	1734.454492	0.796523145	0.01185	0.04492
CASP4	4401.01756	0.796365998	1.6E-07	8.4E-06
SLC6A11	1139.641743	0.795455991	0.00649	0.02869
VEGFC	1479.142822	0.795307119	0.001768	0.01084
AC124789	425.5880216	0.794338675	0.001489	0.00963
CCBE1	197.5283986	0.789558812	0.011981	0.04523
EFCAB1	89.37636477	0.787827965	0.002718	0.01498
RP11-356I	162.6253941	0.786515001	1.05E-06	3.6E-05
BMP2	1793.01166	0.785728738	0.000185	0.002
CARD17	73.31562236	0.785373221	0.002078	0.01232
RND1	150.6364859	0.785250171	0.000756	0.00584
ADAMTS1	2996.991843	0.7832752	0.000377	0.00347
AQP3	42821.76154	0.781488843	0.005767	0.02636
RP6-65G2	214.6079841	0.777853941	2.11E-05	0.00037
RP11-65J2	88.18905541	0.7770775	0.000135	0.00158
SDR16C5	2771.447685	0.776024726	0.000296	0.00288
AFAP1L1	1335.798127	0.775964826	6.52E-06	0.00015
CYP27C1	734.0898797	0.775511733	0.00128	0.00865
AGPAT4	1076.115615	0.77432174	2.24E-07	1.1E-05
EPPK1	4831.66458	0.771098354	0.000304	0.00294
STXBP5-AS	83.98939673	0.769996688	0.001123	0.00782
WNT10A	1469.096983	0.763630589	0.004048	0.02019
KRT17P1	99.19527956	0.756286259	0.001534	0.00981
C19orf33	5158.167768	0.756218723	0.002271	0.01312
HIGD1A	2327.191911	0.755800538	8.15E-10	1.2E-07
RP11-575F	82.78186048	0.755454948	0.011953	0.04521
OVOL1	2819.612809	0.755115883	0.000384	0.00352
CFAP57	90.92406957	0.753547312	0.009731	0.03869
RP11-54H	2618.116157	0.752858463	0.013332	0.04896
XKRX	209.0144332	0.751725503	0.000328	0.00313
INHBA	8621.178588	0.750707788	0.003772	0.01913
ITGA6	55303.83415	0.750391442	4.38E-05	0.00065
BAIAP2L2	789.7816862	0.748320687	0.000495	0.00425
FEZ1	2470.817817	0.748271183	0.000531	0.00449
DAAM1	4211.562347	0.748070504	6.27E-08	4E-06
ERRFI1	4564.979916	0.740500683	6.35E-06	0.00015

TNFAIP8L3	423.9868392	0.737965208	4.01E-05	0.00061
CITED2	2091.894105	0.736661442	1.94E-05	0.00035
IGFBP6	9064.202511	0.736622658	0.011344	0.04343
RP4-647C1	114.4443666	0.736171076	0.003321	0.01734
GLTP	15119.95954	0.734402376	5.13E-05	0.00074
NELL2	4171.869889	0.733569717	0.008401	0.03473
GRPEL2	1514.406204	0.731580362	1.24E-10	2.6E-08
CD24	21682.75385	0.731554583	0.000282	0.00279
PDLIM2	1679.823733	0.731540073	9.77E-07	3.4E-05
TNFRSF10I	518.9883293	0.731481227	0.001181	0.00811
EGFLAM	482.7240116	0.730736435	2.35E-05	0.0004
SFN	209682.312	0.727015433	0.000417	0.00375
IL1RN	16281.69595	0.725583566	0.001417	0.00933
SEMA7A	1094.396302	0.725583237	2.35E-05	0.0004
TM7SF2	1223.735439	0.72395606	0.001864	0.01133
PTHLH	13188.74695	0.723035033	0.00324	0.01701
PPIF	12190.17225	0.722949888	1.11E-07	6.2E-06
PROCR	2018.919793	0.722668676	1.85E-05	0.00033
RP11-557H	260.7584753	0.721927563	0.009525	0.03809
RP3-395M	80.03496132	0.719031086	0.000782	0.006
SLC6A15	1147.153629	0.7182518	0.011003	0.04241
PPP2R2C	6237.828256	0.717282823	0.000452	0.00399
MYL12B	30852.09599	0.716617408	5.08E-08	3.5E-06
ANGPT2	777.8438803	0.715685955	5.52E-07	2.1E-05
IL18	2322.952604	0.714575168	0.000197	0.00212
ANXA3	2759.585128	0.712220701	0.001156	0.008
NT5E	3264.473161	0.70986994	0.003674	0.01876
DCUN1D3	1116.823341	0.708136777	1.19E-11	3.5E-09
DYNC2H1	626.7787987	0.707243613	1.83E-05	0.00033
LTB4R2	757.0372895	0.70630542	0.000123	0.00148
SLC7A5	42138.18725	0.705776941	7.55E-06	0.00017
APCDD1	3899.501214	0.70513238	0.000137	0.0016
CTD-2547L	70.99130917	0.704384869	0.000579	0.00477
RAPGEF3	1089.380783	0.700655024	0.00014	0.00162
C6orf141	566.7663088	0.697964382	0.002342	0.01336
LPAR3	2078.626757	0.694841207	0.000492	0.00424
PPARD	7117.548656	0.694215322	1.31E-09	1.9E-07
OSM	268.7158831	0.693113023	0.008043	0.03368
AMIGO2	2287.528478	0.690950104	0.001484	0.00961
KCNK6	4914.617554	0.689977436	9.91E-06	0.00021
NKPD1	287.0058207	0.687705732	0.006109	0.02744
NACAD	210.4589946	0.68613825	0.003182	0.0168
PLD2	4114.624486	0.685789174	6.94E-08	4.3E-06
COL17A1	87649.58607	0.685284713	0.00681	0.02965
KRT6A	682244.3335	0.685115392	0.000397	0.00361
CKB	5629.648104	0.684278156	0.005131	0.02406
S100A2	117322.2629	0.683930981	0.00109	0.00765

MTAP	2570.572681	0.682493554	0.001739	0.0107
ELOVL7	861.078972	0.682442823	0.000148	0.0017
IL20RB	7320.368379	0.681857516	0.002896	0.01565
LINC00886	107.4406073	0.680761837	0.002231	0.01296
ACER3	2375.294441	0.679599234	4.42E-07	1.8E-05
SERPINB5	26884.54854	0.679344801	2.95E-05	0.00048
RP13-463N	134.0665245	0.677854666	0.005457	0.02531
TGM5	450.5307307	0.677056561	0.009261	0.03737
C1orf116	6069.806859	0.673766633	0.000222	0.00232
STRIP2	617.4456152	0.673603887	0.000236	0.00243
SERPINB8	1731.385428	0.67308354	1.7E-06	5.2E-05
RN7SK	144.4036162	0.672439138	0.00687	0.02984
FOSL1	5743.926994	0.672317483	0.000711	0.00557
RAET1G	457.4091289	0.671985953	0.010057	0.03969
TNFSF9	563.8221927	0.671528775	0.000816	0.00618
RP11-379B	109.4253593	0.669927931	0.00108	0.00759
ADAM8	2112.359682	0.66939278	0.000479	0.00415
MIR205HG	7516.315775	0.669337025	0.000181	0.00197
NAPRT	5648.062953	0.669138845	0.000345	0.00324
NFKBIZ	2481.199127	0.66863189	0.000814	0.00618
TGFA	4706.626452	0.668073585	5.8E-06	0.00014
OTUD1	1460.469385	0.666944945	6.07E-07	2.3E-05
AVPR1A	195.6485499	0.665859439	0.001577	0.00999
HAS2	713.0647929	0.665045291	0.011832	0.0449
CTD-2357A	98.03852914	0.664511948	0.005952	0.02695
TUBB6	15180.08192	0.664211923	1.29E-06	4.2E-05
DLK2	1032.62554	0.66360709	0.001366	0.00907
CSPG4	9484.021667	0.662846729	0.007004	0.0303
MOB3B	1722.298316	0.66099478	0.00056	0.00467
TMC7	320.8741508	0.66007041	1.58E-05	0.0003
CTSV	5412.827357	0.658198105	0.001071	0.00757
ARG2	486.3937789	0.657207276	0.00223	0.01296
CTD-3099C	62.49828578	0.655377163	0.008538	0.03516
HPSE	1148.532697	0.652851049	4.53E-05	0.00067
TMEM154	4728.050213	0.648501882	0.000621	0.00504
MEDAG	573.769313	0.647952372	0.007357	0.03154
YOD1	1401.799904	0.645542693	3.87E-07	1.6E-05
PCDH7	4210.492823	0.644679829	0.008953	0.03642
ANXA9	368.8117482	0.643574872	0.004243	0.02088
FERMT1	8201.197681	0.643349278	8.76E-05	0.00111
UGCG	4430.329523	0.643346903	1.72E-07	8.8E-06
MST1R	1645.100042	0.643335268	0.000346	0.00325
NDFIP2	5274.27203	0.643281547	9.16E-06	0.00019
CPEB2	1769.708618	0.642375139	0.000115	0.0014
LY6K	1681.257245	0.641923986	0.000877	0.00652
VIPR1	530.3106948	0.641871371	0.002886	0.01563
KLK11	5066.267458	0.640173185	0.012688	0.0472

ALDH1A3	1721.364185	0.639274597	0.005019	0.02364
THSD1	1367.377632	0.639132239	0.001353	0.009
CMB9-22P	279.2345062	0.638954853	0.001664	0.01034
OTUB2	420.8278557	0.638806699	0.000583	0.00479
PNP	6090.817699	0.63743894	8.96E-07	3.1E-05
MCTP1	779.2835985	0.636986443	0.000199	0.00213
DOCK5	1576.783159	0.636038192	7.42E-06	0.00017
LTB4R	4523.51052	0.635137969	0.000419	0.00376
ZNF426	1085.884408	0.633575459	6.53E-06	0.00015
RP11-521E	253.2443606	0.633420554	8.73E-05	0.00111
ALDH3B2	6949.293437	0.633021217	0.010795	0.04187
BNIP3	3545.667152	0.632728776	2.18E-05	0.00038
CTB-63M2	151.9554732	0.63196905	0.0073	0.03133
ENKUR	60.56351577	0.629890708	0.006945	0.03009
BNIPL	1569.434905	0.62893081	0.007844	0.0331
MYO1B	15362.60657	0.627930513	2.37E-05	0.00041
LDHA	69328.033	0.626887171	7.75E-07	2.8E-05
DUOXA1	3054.074755	0.626580812	0.000234	0.00242
LDHAP4	227.5922631	0.625241893	0.001117	0.00778
FCHSD1	1074.259561	0.625141929	0.000259	0.00263
SH3BGRL3	20379.49193	0.624956559	6.84E-07	2.5E-05
EFNA3	1056.269142	0.624578936	6.94E-05	0.00094
DUSP5	2923.930858	0.624324222	0.000386	0.00353
TGFBI	55510.5957	0.62318438	0.010411	0.04082
DYNLL1	15348.62936	0.621168225	9.28E-09	9.2E-07
KLC3	1804.516041	0.619348627	7.82E-05	0.00103
HMOX2	5399.866938	0.618722252	5.55E-08	3.7E-06
PLK2	5258.867303	0.618413666	0.000463	0.00406
TIPARP	4592.988627	0.618303717	8.63E-06	0.00019
CSRP2	2871.588723	0.617842733	0.000568	0.00471
MAFB	7115.177189	0.617629518	0.001264	0.00856
SERPINE2	7933.071515	0.617483805	0.009284	0.03742
STK17A	4474.63451	0.616860396	2.32E-06	6.7E-05
TMEM125	322.4118974	0.615906874	0.009379	0.03771
RTTN	944.869415	0.613941878	0.00058	0.00478
ITGA3	33264.10161	0.612126468	0.000861	0.00643
RP11-204C	109.0622432	0.610644092	0.000552	0.00462
MRPL48	1231.074559	0.607768196	1.48E-07	7.9E-06
IER3	7412.449929	0.607306442	0.001102	0.00771
MOCS2	1342.429109	0.607015583	3.56E-09	4.1E-07
RP1-95L4.4	103.8995805	0.6067786	7.58E-05	0.00101
FAM83A	18287.36778	0.606430644	0.007583	0.0323
FLRT2	2930.480521	0.605218577	0.010047	0.03966
PERP	140783.8608	0.602824171	1.08E-05	0.00022
C15orf62	275.4496497	0.601974088	0.009396	0.03775
ANAPC15	805.8208108	0.600359058	5.2E-06	0.00013
LYPD3	33249.33435	0.599792099	0.004773	0.02287

ATG16L2	800.0710721	0.599790419	0.001446	0.00944
MBOAT2	5345.389919	0.59927235	0.000805	0.00612
DGAT2	1154.138037	0.598428715	0.009077	0.03677
NEK7	3447.208993	0.597795663	4.27E-07	1.8E-05
CAP1	26166.48584	0.597033566	3.5E-09	4.1E-07
P2RY2	1254.504991	0.595621818	0.001285	0.00868
RELT	935.9156484	0.595175889	1.21E-05	0.00024
RRAS2	2095.578749	0.594263307	6.64E-06	0.00015
FAM89A	1853.166212	0.592353581	4.01E-05	0.00061
PLCB3	6258.731798	0.592284565	1.99E-06	5.9E-05
CDK5R1	1362.647124	0.59029679	7.48E-05	0.001
SEC61G	5540.760295	0.590078857	0.00294	0.0158
LYPLA1	4900.181017	0.589312393	3.01E-08	2.3E-06
SDC4	21279.48157	0.588510713	7.78E-05	0.00103
PKP3	20079.30131	0.588054108	0.000176	0.00193
THBS1	19930.47176	0.58640697	0.010793	0.04187
DGCR11	148.7816037	0.585424126	6.85E-05	0.00094
SLC38A2	31860.4663	0.585073155	3.47E-06	9.4E-05
GFOD2	1982.479232	0.585067066	1.6E-08	1.4E-06
DUSP18	359.4537352	0.585046595	5.52E-07	2.1E-05
DUOX1	7095.61957	0.584434571	0.000816	0.00618
CCDC64B	1502.861304	0.584191472	0.010894	0.04212
RAB38	4081.993353	0.583466975	0.001414	0.00932
DNAAF3	106.1207214	0.582677128	0.012072	0.0455
ADAMTS6	139.7760876	0.582540484	0.008237	0.03429
MIR210HG	605.9311403	0.582139041	0.002109	0.01244
GCNT4	216.409569	0.580759315	0.002189	0.01279
ABLIM3	997.1763565	0.580557724	0.00591	0.02679
CCL3	371.5792272	0.579443604	0.01258	0.04689
NMNAT2	185.0362702	0.579377579	0.001171	0.00806
SUCLA2	2051.343781	0.579329658	9.89E-08	5.7E-06
S100A16	35592.34981	0.578880254	8.26E-05	0.00107
EPHA2	9347.416754	0.578556591	0.000317	0.00305
C12orf29	1125.380552	0.578371294	8.17E-10	1.2E-07
BPGM	3445.61785	0.57808066	0.000988	0.00709
SDCBP2	2774.589175	0.576991265	0.003326	0.01736
ZNF365	434.5021168	0.576863349	0.012643	0.04706
MT-RNR1	40187.26826	0.576781293	0.000927	0.00677
ESM1	336.4557149	0.576391415	0.002933	0.01578
SREK1IP1	1783.49735	0.574957422	1.91E-08	1.6E-06
GJB3	7684.071889	0.57358599	0.000123	0.00147
AJUBA	5219.352564	0.573418845	0.000284	0.00281
ACOT11	1093.725795	0.573204277	0.001454	0.00947
RP11-58E2	79.00596376	0.571278782	0.00156	0.0099
PKP2	1767.158522	0.570836275	0.006315	0.02815
STARD10	4377.588379	0.570407079	0.00015	0.00172
TNFAIP3	6901.676642	0.569579333	0.003495	0.01802

FBLIM1	8486.188398	0.569249714	0.000153	0.00175
TNS4	20401.8687	0.568990144	0.000689	0.00544
UCN2	369.5629338	0.568801581	0.012351	0.04623
KRT5	816948.3456	0.567255235	0.000669	0.00533
ZNF185	12038.9968	0.567123378	0.004118	0.0204
UNC13D	2129.792346	0.566834409	0.00118	0.00811
PANX1	2665.705342	0.566688894	2.74E-05	0.00046
S100A3	350.6534347	0.565617985	0.00306	0.01633
RHBDL2	752.6420285	0.564683177	0.000407	0.00368
CDH3	34569.28085	0.562941184	0.000907	0.00665
CAV2	8140.376522	0.56239178	3.56E-05	0.00055
DEPDC7	427.7019558	0.56224998	0.001235	0.00841
SOX15	3867.531013	0.561827333	0.000572	0.00473
PEAR1	389.0111071	0.559226244	0.000639	0.00515
FKBP1B	193.4604716	0.557845713	0.001694	0.01048
SUGCT	551.7425974	0.557308454	0.011243	0.04312
ELOVL1	7453.732964	0.556752921	1.2E-08	1.1E-06
CERS3	4591.251666	0.5567523	0.003746	0.01903
DLX1	225.5716567	0.555182639	0.011703	0.04451
C9orf84	82.35195125	0.554953494	0.000471	0.0041
GPR153	3723.006203	0.554522944	0.00046	0.00405
ESRP2	3953.958466	0.55388443	0.000122	0.00147
PSMG1	2015.558447	0.553561197	5.81E-07	2.3E-05
RN7SL2	1896.158315	0.552920847	0.008063	0.03372
NDEL1	4491.891581	0.552185899	7.44E-07	2.7E-05
GJB4	852.5856829	0.551325039	0.009237	0.03732
RPS7P1	283.2806268	0.550222532	0.000891	0.00657
NARS2	893.4938025	0.549883551	7.96E-06	0.00018
RP11-550F	75.08345086	0.549545953	0.000986	0.00709
CAB39	7746.891908	0.549437078	2.35E-06	6.8E-05
AMPD3	1532.040908	0.549397443	3.93E-05	0.0006
BCAR3	1767.514151	0.548754282	0.003576	0.01837
CYB5R1	8293.953422	0.548000922	0.000719	0.00563
CDH13	3838.142759	0.547584942	0.004307	0.02113
NRBP2	1576.801767	0.547097199	0.000212	0.00224
TRIP13	1821.598677	0.546420424	0.000422	0.00378
NIP7	2710.080276	0.546365602	2.88E-09	3.5E-07
DENND2C	1681.941619	0.545332889	0.000491	0.00423
BZW1	7655.044069	0.544961806	1.14E-08	1.1E-06
PEX3	849.7198837	0.544423681	6.9E-08	4.3E-06
RPS6KA4	6751.277392	0.544284831	6.76E-06	0.00016
RGS2	1697.222818	0.543785054	0.000822	0.0062
HBEGF	4173.54168	0.542319632	0.004495	0.02178
LRRC20	759.9418887	0.54157663	0.003417	0.01773
DYNLL1-AS	486.211973	0.53964964	1.21E-08	1.1E-06
PRRG1	555.143969	0.539301502	0.000271	0.00272
TMEM79	6098.867182	0.538930865	0.003971	0.01988

FLVCR2	545.3219189	0.538322264	0.00251	0.01407
METRNL	4087.808249	0.537325908	0.000394	0.0036
CTNNBIP1	5714.654142	0.536829422	1.93E-05	0.00035
NT5C3A	1876.946153	0.536070297	6.73E-07	2.5E-05
PFKFB2	1877.712942	0.535104717	8.62E-06	0.00019
PXN	10348.52431	0.534655643	3.69E-05	0.00057
MRPL39	990.7472837	0.533771305	3.95E-08	2.9E-06
EIF6	11900.42371	0.533582989	5.15E-06	0.00013
C1orf74	1136.832607	0.533450938	0.000538	0.00454
ADK	3213.741639	0.533176496	1.39E-07	7.6E-06
ATP6V1D	4240.295992	0.532486392	2.05E-07	1E-05
VSIG1	94.7011064	0.531797548	0.005675	0.02605
PTK6	3012.344558	0.531721112	0.003098	0.01649
RP11-800A	1211.54387	0.531445509	0.009264	0.03737
PSMA6	2138.517231	0.531417549	2.44E-07	1.2E-05
CDK7	1473.084681	0.531311173	2.77E-08	2.2E-06
GLS	4691.146954	0.530962752	1.94E-05	0.00035
GPR126	998.7119611	0.530815965	0.007783	0.0329
MPHOSPH	1585.824017	0.52926672	7.2E-07	2.6E-05
TNFRSF21	12005.68028	0.52764366	0.001045	0.00743
GALNT6	5749.719615	0.526804138	0.012891	0.04774
CTNNAL1	2696.15762	0.52529388	0.006895	0.02994
TMEM184	2203.681418	0.524780031	0.004366	0.02133
PLK3	1512.072465	0.524279126	9.75E-05	0.00122
TXNDC17	5238.769128	0.522487991	0.001414	0.00932
DBNDD2	175.7142632	0.522172375	0.001104	0.00771
PHLDA1	10942.37826	0.52190542	0.000822	0.0062
CENPT	1742.652273	0.521399455	3.29E-05	0.00052
IRS1	3928.518939	0.520969465	0.001921	0.0116
EVPL	14521.62365	0.520608826	0.00362	0.01853
DUSP1	14735.24811	0.520096955	0.00563	0.02592
CTB-89H12	841.5978067	0.519488934	0.000439	0.00389
PYGL	12252.75351	0.519203235	0.000752	0.00581
OLFML2A	5195.953474	0.518675006	0.009718	0.03868
ANO9	1241.511324	0.518158994	0.004468	0.02169
SLC7A1	12249.58887	0.517877931	0.000502	0.0043
GLO1	8593.288798	0.5166962	3.4E-07	1.5E-05
KCTD21	780.8602118	0.515596131	3.57E-05	0.00056
NCF2	1901.451896	0.515521698	0.000426	0.0038
TM4SF19-A	108.3544688	0.515307533	0.010701	0.04166
PTRF	29358.17981	0.515247815	4.18E-05	0.00063
DOK4	1992.525149	0.515241293	0.000267	0.00269
NOD2	1412.043054	0.51487446	0.007506	0.03208
CEACAM19	1636.041555	0.514559847	0.004693	0.02253
DNMT3B	665.964081	0.514555473	0.001303	0.00877
EHD1	5974.695728	0.513914548	2.13E-05	0.00038
FMNL2	3612.788188	0.51307373	2.87E-05	0.00047

ANO7P1	277.5427506	0.512973148	0.002737	0.01504
RAB25	6814.692967	0.512946328	0.003511	0.01809
S100A11	62759.75896	0.512313912	7.85E-06	0.00017
GRHL1	3798.892012	0.510817348	0.007916	0.03333
ARSJ	1254.619979	0.508482643	0.009381	0.03771
PLCD1	1888.690205	0.507721821	0.00561	0.02586
YAP1	8981.102275	0.507556685	0.00199	0.0119
PIM1	5934.769474	0.507276373	0.000217	0.00228
F2RL1	2956.144791	0.507219018	0.000632	0.0051
SFR1	460.3201403	0.506928034	2.86E-05	0.00047
ELL2	7103.958425	0.506077785	0.000365	0.00338
BDKRB2	2159.733709	0.505484344	0.000265	0.00268
CASP1	2230.285918	0.505244761	0.001774	0.01087
MAP7D1	15322.36168	0.505231931	4.34E-05	0.00065
RASA4CP	127.7065601	0.504958591	0.004689	0.02252
GLIPR1	1960.518973	0.503357302	0.007971	0.03349
PRDM1	3295.385539	0.503236902	0.000947	0.00688
PIP5KL1	248.7581515	0.50310409	0.004934	0.02338
GANC	1129.57408	0.502415467	1.86E-06	5.6E-05
GPSM2	2611.408596	0.502359397	0.000506	0.00432
JUP	125114.591	0.501831607	0.000298	0.0029
BNC1	6863.26666	0.500926868	0.001787	0.01093
FAT1	17625.83779	0.500851982	0.012255	0.04599
SLC27A4	5510.206343	0.499793836	0.000118	0.00143
MYL12A	16883.18615	0.499782964	2.26E-05	0.00039
DHRS7	3647.223089	0.499398767	3.51E-05	0.00055
CD47	9366.771355	0.499390251	8.26E-05	0.00107
SUB1	9133.955101	0.499274422	7.24E-05	0.00098
WFDC1	140.5656696	0.498708179	0.009774	0.03882
FER	1254.036534	0.498267424	0.000108	0.00133
NAB1	4737.900411	0.497646986	9.62E-06	0.0002
ACAA1	1971.17962	0.495812567	1.47E-05	0.00028
AIG1	1936.274411	0.49473089	7.84E-05	0.00103
RAB31	10209.65138	0.493654035	0.000781	0.006
AVPI1	1826.154865	0.492555136	0.00153	0.0098
UCHL3	861.547035	0.491892004	0.000426	0.0038
RP11-703I	147.6323102	0.491584767	0.008551	0.03517
CARD10	3507.938596	0.49154435	0.002709	0.01495
PPP4R1	11647.35765	0.491457359	0.000116	0.00142
AMMECR1	2546.241674	0.489941303	0.000477	0.00414
STON2	4016.962595	0.489703728	0.001165	0.00803
C6orf132	5987.483536	0.489584812	0.0018	0.011
HS3ST1	662.546184	0.488153323	0.01303	0.04813
RND3	7700.668728	0.48811991	0.001122	0.00782
SOWAHC	6307.373211	0.487802913	0.000118	0.00143
ITGB1	20192.13866	0.487764137	0.002315	0.01329
RP5-1085F	318.540943	0.487723103	0.003735	0.01899

DSG3	55602.94813	0.487653051	0.008408	0.03474
RP11-73M	497.3948714	0.487252102	0.000569	0.00472
GM2A	16513.14264	0.487165289	0.000476	0.00414
SPRY4	1568.36895	0.486227437	0.000399	0.00362
YWHAZ	92931.98048	0.486188235	3.55E-07	1.5E-05
MMP17	553.6147301	0.486182583	0.009726	0.03869
PLCD4	329.9627139	0.485255341	0.001969	0.01183
SMTN	5397.25634	0.484765879	0.000384	0.00352
SPAG1	773.0341321	0.484755879	4.91E-05	0.00072
OXSR1	3401.280388	0.484645066	8.87E-07	3.1E-05
ARL4D	3058.844267	0.483764353	0.002638	0.01463
ORMDL2	2468.774697	0.483357114	4.78E-06	0.00012
COL13A1	372.7180984	0.482989937	0.002112	0.01245
TEC	132.7761555	0.482957569	0.003567	0.01834
ZNF860	110.4223786	0.482585537	0.003749	0.01904
ETS2	15062.81891	0.48200087	0.000167	0.00186
CDKN1A	18845.24182	0.481697723	0.00047	0.0041
SLC19A2	752.3128416	0.480956021	6.95E-05	0.00094
PLEKHG5	3125.404316	0.47981359	0.000145	0.00167
HIST1H2BI	971.9059941	0.479672207	0.012222	0.04591
CLCA2	40731.57469	0.479585872	0.012435	0.04648
CBLC	2880.023507	0.47942326	0.009804	0.03889
SGPP1	1118.088461	0.479097445	0.000452	0.00399
C4orf32	662.4632734	0.479057183	0.000968	0.00697
MAP3K9	1674.505133	0.477815022	0.001872	0.01137
RPS2P46	369.4009622	0.476637459	0.011972	0.04523
C12orf75	2583.242159	0.476382236	0.010765	0.04181
NUDT15	1781.493239	0.475625167	3.71E-06	9.9E-05
MED31	413.0185436	0.4747646	8.66E-06	0.00019
FJX1	1361.028846	0.474668964	0.00188	0.0114
PPIAP22	1907.578393	0.474419081	5.58E-06	0.00014
CLCF1	499.3860544	0.473857297	0.011007	0.04241
LIG4	729.6171658	0.473729134	0.00023	0.00239
REEP3	3571.385809	0.473605144	9.26E-06	0.00019
LRRC8C	1873.232697	0.473247568	0.001436	0.00941
HSBP1L1	583.1939106	0.472765981	0.001329	0.00889
PORCN	1246.52551	0.471848467	0.002861	0.01554
ZC3H12A	4319.168555	0.471013425	0.008922	0.03634
PLIN3	9097.232214	0.470595249	5.23E-05	0.00075
EIF2S1	6481.901738	0.46993359	2.3E-08	1.9E-06
POLB	1130.108487	0.468741057	0.000886	0.00656
GGH	3201.173965	0.468686805	0.000378	0.00347
CENPK	367.5682501	0.468669516	0.001173	0.00807
PAG1	1067.695127	0.467209124	0.00513	0.02406
CTD-2510F	183.3064332	0.466484403	0.001928	0.01163
SLC16A1	10126.28005	0.466078211	0.001097	0.00768
DSE	4482.650057	0.46587996	0.003121	0.01657

ARHGAP5-	259.0240148	0.46572742	0.001233	0.00841
YWHAZP5	178.83463	0.465660094	0.010731	0.04172
ACOT7	4761.544356	0.465342738	6.83E-05	0.00094
TMEM54	7366.778193	0.464419732	0.005641	0.02596
GJB5	4388.65968	0.463472373	0.00137	0.00909
CCT5	18063.65976	0.463308928	5.85E-06	0.00014
TCAF2	645.505528	0.46280142	0.005154	0.02412
NRBF2	1888.415068	0.461459068	1.44E-06	4.6E-05
NUTF2	6610.833986	0.461364139	7.97E-06	0.00018
DUS2	1203.67372	0.460301284	2.66E-05	0.00044
SPTSSA	3824.211551	0.459142882	0.000179	0.00195
ARHGEF10	933.5913211	0.458875953	0.002356	0.01341
CHORDC1	2010.92114	0.457726064	0.000122	0.00147
AK4	2693.629452	0.456719569	0.001558	0.0099
DCUN1D5	2376.529886	0.456627039	0.002759	0.01512
MREG	2089.109145	0.456333317	0.001589	0.01003
ZDHHC13	1648.033104	0.455702004	1.29E-05	0.00025
RP11-73M	70.14114392	0.455690875	0.0004	0.00363
KLF6	11067.27631	0.455678078	0.002337	0.01335
PTPRE	1835.349172	0.45556674	0.000266	0.00269
CDC20	5231.013549	0.455516279	0.000664	0.0053
RP11-158L	87.58929545	0.454698639	0.006454	0.02857
PFDN6	2066.166949	0.454339682	0.001071	0.00757
NRAS	4907.6882	0.453910665	2.24E-05	0.00039
RIOK3	7128.649793	0.453772385	0.000277	0.00276
CYB5B	4510.481349	0.452987847	2.88E-07	1.3E-05
TM4SF18	205.8005851	0.452345276	0.001874	0.01137
RABGGTA	2566.51141	0.452145607	6.05E-05	0.00085
RHOB	12788.49068	0.451066369	0.002106	0.01244
CH17-360D	551.626521	0.450472994	0.002142	0.01259
MAL2	17192.70283	0.449993822	0.013072	0.04823
ADAT2	406.1283365	0.449728556	0.000122	0.00147
TFAP2A	5098.461945	0.449625815	0.000256	0.0026
MCU	2098.791472	0.449458501	1.98E-05	0.00035
MT-TP	1548.806508	0.4481645	0.001472	0.00956
SERINC2	17096.57209	0.448093366	0.005856	0.02662
PRNP	23957.84682	0.447942595	0.001348	0.00897
TUBA1C	19222.19794	0.446166128	3.58E-06	9.6E-05
TMCC3	1864.166013	0.445912799	0.003404	0.01769
RPS6KB2	4526.155884	0.445904904	0.001311	0.0088
TTPAL	2612.511918	0.445430744	0.00019	0.00205
SQRDL	4938.02905	0.443613836	0.001715	0.01057
JHDM1D-A	956.7777073	0.443102182	0.006381	0.02836
DDX60L	1952.377316	0.443016938	0.007461	0.03192
RAB6A	8516.914943	0.442809071	2.28E-05	0.00039
NUAK2	2099.279315	0.442603551	0.002348	0.01338
FAM210A	1482.791403	0.44198679	0.000283	0.0028

COA4	2386.418302	0.441697958	9.01E-05	0.00114
FAM83G	10290.99423	0.440921622	0.003799	0.01923
LUZP1	4671.357133	0.44070692	0.000147	0.00169
SNAI2	4732.595101	0.440323262	0.000299	0.00291
DUSP6	5242.934254	0.440185712	0.011332	0.04339
PPIA	25713.15232	0.439821113	2.61E-05	0.00044
PRMT3	1217.270279	0.439592949	2.01E-05	0.00036
PRR11	1777.859532	0.439328007	0.0007	0.00551
ACTN1	29683.1756	0.439225111	0.001504	0.00969
GALE	2294.796351	0.43862982	0.000914	0.00669
CLTB	14953.00381	0.437789664	0.009996	0.03949
PPP1R14C	4571.250502	0.437528701	0.003451	0.01786
CES2	6476.124508	0.436491319	0.010946	0.0423
SLC35E4	570.4408753	0.436016803	0.00047	0.0041
RPS3A	16888.31759	0.435888841	0.001492	0.00963
IMPA2	7319.160885	0.435275316	0.010863	0.04206
MDFI	4717.332914	0.433300503	0.006048	0.02724
ETHE1	2491.716165	0.43250288	0.000831	0.00626
NABP1	2161.637271	0.432063767	0.002731	0.01502
TGFBR1	4737.825414	0.431857975	0.000295	0.00288
TES	5683.726002	0.431713996	0.000102	0.00127
WWC1	1862.032553	0.431693404	0.000234	0.00242
USP15	3529.419562	0.431293597	1.56E-06	4.9E-05
S100A6	51618.99139	0.431161383	0.013169	0.04856
LPCAT2	2521.477736	0.430660828	0.004777	0.02287
TLDC1	3916.161155	0.430639157	0.001981	0.01187
NEIL3	283.6011631	0.430443711	0.008914	0.03633
UBE2Q2	3291.976678	0.430144214	8.3E-05	0.00107
VPS29	3225.593198	0.429167081	3.15E-06	8.6E-05
ARNTL2	6355.133995	0.429127201	0.003481	0.01798
SLC25A30	747.5526086	0.428981621	3.78E-05	0.00058
MYO5A	5234.644849	0.427914658	0.002173	0.01274
RAB32	1659.367208	0.427760988	0.00617	0.02761
PITPNC1	1247.332424	0.427721698	0.000681	0.00539
ABLIM1	13555.10765	0.42748499	0.004649	0.02238
FASTKD3	459.5523176	0.426988297	0.000149	0.0017
BAIAP2	8041.954307	0.426791365	0.000223	0.00234
PIK3CD	2132.231883	0.426419979	0.002066	0.01227
CAPRIN2	776.6493253	0.426119476	0.004085	0.02029
RAB10	16011.4777	0.425625023	1.66E-05	0.00031
CD44	61930.98756	0.423460699	0.000471	0.0041
INO80C	1083.236375	0.423265754	0.000759	0.00586
SH2D2A	641.2709264	0.423210356	0.007703	0.0327
STARD3NL	1110.400196	0.423093499	0.001326	0.00888
FAM162A	4404.230657	0.423014331	0.006344	0.02823
PRSS8	5926.360992	0.421791657	0.009057	0.03671
TUBB4B	33211.22695	0.420342145	0.001988	0.0119

MRPS15	3918.436922	0.419365582	0.000168	0.00186
LAMTOR1	5830.85261	0.418424373	0.000366	0.00339
WDR66	3213.29937	0.418410056	0.006793	0.0296
ANXA2P2	947.8567564	0.418371646	0.001534	0.00981
AP3S1	3177.082402	0.41791218	0.000108	0.00134
STRN3	2435.184982	0.417548872	3.8E-05	0.00058
SLC7A6	806.5503331	0.41748511	0.000172	0.00189
MAPK6	8471.193781	0.416974858	0.000954	0.0069
LRP11	4134.816757	0.415046946	0.000146	0.00167
PAAF1	944.448489	0.414902613	0.001471	0.00956
C16orf74	1301.19102	0.414868089	0.008881	0.03622
MPP5	1839.957627	0.414494877	2.84E-05	0.00047
CDC42	14003.4487	0.41437771	8.27E-08	5E-06
PGAM1	5031.557265	0.414224047	3.04E-05	0.00049
AMOTL1	8198.634354	0.413033471	0.002896	0.01565
NOP14-AS	1212.681376	0.41297153	0.002987	0.01601
WSB1	3601.770327	0.412459048	8.74E-05	0.00111
NPEPPS	6365.468999	0.412024594	1.96E-05	0.00035
TXNDC9	1956.069691	0.411874783	2.59E-06	7.3E-05
MAPKAPK3	2996.273064	0.411278189	0.000171	0.00188
ZCCHC9	865.8885674	0.410425522	1.03E-07	5.9E-06
TPMT	2136.883079	0.410338308	0.000123	0.00147
EIF4E	1923.256318	0.409882874	3.86E-07	1.6E-05
RPS6KA1	5071.841685	0.409866961	0.000287	0.00283
RAN	21067.32126	0.408485756	6.91E-07	2.5E-05
VDR	3546.995032	0.408387826	0.00282	0.01539
NUP37	1540.471762	0.407723964	2.53E-05	0.00043
TPCN2	1438.035185	0.407589379	0.013596	0.04976
LAD1	30541.38957	0.407261765	0.001442	0.00944
TMA7	2897.838039	0.407119043	0.003393	0.01766
FSCN1	41516.62115	0.406339349	0.00088	0.00654
TSC22D2	3370.041291	0.406071534	0.002189	0.01279
NME2	92.25484598	0.405695452	0.005843	0.02657
ANKRD13A	2941.247361	0.405367923	6.2E-06	0.00015
MARK3	5569.044112	0.405336339	4.93E-06	0.00012
KIFC3	3999.676891	0.405303034	0.00771	0.03271
DSTN	21005.50417	0.404323155	5.17E-05	0.00074
DRAP1	7549.935561	0.404275879	0.003553	0.01828
RPS3	63585.74768	0.40414385	0.003889	0.01958
NPM1P27	155.3581802	0.40384928	0.007369	0.03157
MALAT1	4974.526596	0.40367564	0.006382	0.02836
GNG12	9335.296275	0.402862525	0.000467	0.00409
AMOTL2	3783.867975	0.402566279	0.001144	0.00793
TAF1D	3117.764261	0.402369457	0.002289	0.0132
EIF3EP1	137.5450986	0.4022281	0.005825	0.02651
CMTR2	1408.820151	0.401894771	7.69E-05	0.00102
CD58	1010.945664	0.40153819	0.000182	0.00197

PPIL1	2277.275696	0.40115776	2E-05	0.00036
GLRX3	4995.516687	0.40095887	1.03E-06	3.5E-05
GNA15	6867.971004	0.400881065	0.001555	0.0099
S100A10	31948.41418	0.399709485	0.002695	0.01489
RNF11	6057.26431	0.399704342	0.000136	0.00159
DUSP7	8004.169197	0.399442893	0.000505	0.00432
UBE2N	4850.217929	0.399026741	2.64E-07	1.2E-05
CNEP1R1	691.3125533	0.398811474	2.62E-06	7.4E-05
TMEM256	73.51169648	0.398653759	0.00308	0.01643
MRPL32	2052.890187	0.398492133	4.84E-05	0.00071
ARPC2	23199.62709	0.398415199	8.7E-06	0.00019
SKP2	2103.883881	0.397795938	0.000991	0.00711
QSOX1	13594.59417	0.397550391	0.000202	0.00216
RAB11A	9808.539578	0.39683054	3.86E-05	0.00059
C14orf119	3369.138017	0.396719015	1.31E-05	0.00026
GFOD1	2166.104928	0.396577669	0.001243	0.00845
ZCCHC17	1884.911706	0.396270872	3.09E-06	8.4E-05
OBFC1	1258.992242	0.395294493	1.68E-05	0.00031
UBE2F	2067.356233	0.395147972	0.001498	0.00966
STX2	600.481346	0.394019494	0.001131	0.00787
PAPD5	1075.306808	0.393220364	1.1E-05	0.00022
ATPIF1	4048.81541	0.393207947	0.001648	0.01027
DNAJB6	7149.016178	0.393138066	8.32E-05	0.00107
MRPS10	3171.414834	0.392321405	5.07E-05	0.00073
RP5-857K2	81.375353	0.391631938	0.007578	0.03229
NME1	3873.297856	0.391461437	0.006245	0.0279
MRTO4	3786.202052	0.391332409	0.000192	0.00207
FBXL2	221.4182869	0.390894388	0.00408	0.02029
UBE2V2	3778.687222	0.390756379	6.88E-05	0.00094
ROBO4	883.6175492	0.390403047	0.002107	0.01244
IMPDH1	6232.679725	0.390377378	0.00231	0.01328
TSTA3	7036.938013	0.39007946	0.002654	0.01471
RAB3D	3857.425525	0.38930541	0.00407	0.02027
BACH1	3828.972631	0.38926136	0.002079	0.01232
TPD52L2	10557.81927	0.389184486	0.000115	0.0014
PA2G4	11170.14562	0.3886947	3.24E-05	0.00052
TRIP10	3927.521941	0.388682094	0.000374	0.00345
RNF144B	2567.557475	0.388400317	0.005595	0.0258
RP1-191J1	77.10739619	0.388303744	0.003681	0.01878
CCNB1	4155.014054	0.388086231	0.001665	0.01035
GALNT3	3503.908671	0.387502483	0.006112	0.02745
LYST	1980.175015	0.38744811	0.001007	0.00721
TEX30	451.9918617	0.387442153	0.001094	0.00767
RBMS2	2285.260817	0.386877259	0.00082	0.00619
HSDL2	2263.557095	0.386867476	0.000179	0.00195
HEBP2	3956.483876	0.386644673	0.001059	0.00751
CCNYL1	1699.445648	0.385888867	0.003662	0.01871

SLIRP	2119.153638	0.385372515	0.008956	0.03642
AP000648	145.7221374	0.384957561	0.002572	0.01435
RPL27	31498.18249	0.384679907	0.001684	0.01043
APBB2	2474.728043	0.384448021	0.004365	0.02133
USB1	5276.712361	0.384440639	0.000102	0.00127
GATB	1088.308552	0.384438284	0.00011	0.00135
NEDD8	4267.65184	0.38425469	0.000932	0.00679
TMBIM1	20623.89579	0.384241806	0.000515	0.00439
PLEKHF2	1998.542505	0.38347368	5.72E-05	0.00081
CLNS1A	4679.24228	0.383325855	0.001118	0.00779
ATP5H	6748.530674	0.382427383	0.000516	0.00439
FHOD1	2053.97875	0.382385161	0.000681	0.00539
SLC25A37	1820.868907	0.382231425	0.002849	0.01549
ZMAT5	782.4474453	0.381905085	0.009936	0.03929
RP11-29G8	87.43890962	0.380211851	0.008642	0.03545
CD3EAP	513.7003776	0.380026721	7.09E-05	0.00096
DEPDC1	964.7907387	0.379870617	0.004724	0.02267
USP53	1663.660193	0.379792422	0.002739	0.01504
PPP2R5B	1325.66698	0.379628409	0.00013	0.00154
TAF13	2050.942546	0.379360733	0.001314	0.00882
CIRH1A	3308.670153	0.37894315	7.26E-05	0.00098
ATP5F1	6692.067627	0.378876779	7.45E-06	0.00017
LDLRAD3	1104.508254	0.378479444	0.001237	0.00842
MET	6494.832867	0.378456547	0.003224	0.01697
ODF2L	1310.51202	0.378270614	0.000728	0.00566
FOXN2	1541.765674	0.37760086	0.001334	0.00891
FAM103A1	658.7324378	0.377562373	5.28E-06	0.00013
CAPZA1	10909.73831	0.377177133	8.67E-05	0.00111
PHLDA2	1686.555126	0.377158178	0.013196	0.04865
C1GALT1	1582.232099	0.376978614	0.000399	0.00362
USMG5	3131.727597	0.376805302	0.005945	0.02692
DEPDC1B	602.2666527	0.376189119	0.004747	0.02276
PPP1R14B	1267.058546	0.375890573	0.010265	0.04034
CHMP5	4132.380978	0.37586154	0.001431	0.0094
RP11-582E	396.8409218	0.375817459	0.008403	0.03473
ALG8	1583.485297	0.375800161	0.001927	0.01163
PIGL	335.7082893	0.375662269	0.000888	0.00656
SLC26A6	968.2759507	0.375565855	0.001073	0.00758
EFNB1	12518.32476	0.375402788	0.003237	0.01701
HIF1A	19651.68665	0.375359648	0.00598	0.02703
FLNB	27680.20411	0.37501169	0.004801	0.02296
SPCS2	1862.656612	0.373846175	2.91E-05	0.00048
MT-CO3	199045.7952	0.373280789	0.005981	0.02703
EIF5A	38416.47316	0.372740114	0.000476	0.00414
VPS37B	4116.945623	0.372709706	0.000568	0.00471
RAP1GDS1	2561.514513	0.372552494	0.000134	0.00157
CCT8	9828.347663	0.372462246	2.76E-06	7.7E-05

CDCP1	8758.147333	0.372407959	0.00412	0.0204
MVK	1834.832104	0.372402136	0.003824	0.01933
PPME1	4090.77317	0.371456871	0.000786	0.00602
CENPQ	429.8527736	0.371269733	0.000898	0.0066
SNRPF	2964.564919	0.371131645	0.004169	0.02057
GLMN	476.129551	0.371063053	0.000184	0.002
DPM1	2032.407361	0.370810767	1.86E-05	0.00034
EHD2	13704.70298	0.37075607	0.004719	0.02265
KIF1C	12389.11393	0.370090417	0.001843	0.01123
ERO1L	12070.17182	0.370053742	0.005768	0.02636
SCFD1	2662.451779	0.369947601	4.97E-07	2E-05
UBA6	5248.594648	0.369831278	0.001004	0.00719
TUBG1	3358.21288	0.369556569	3.73E-06	9.9E-05
NDUFC2	1603.563352	0.369281896	0.007554	0.03223
C16orf72	4591.742708	0.369244091	6.84E-06	0.00016
MT-CO2	175771.4469	0.369097491	0.003682	0.01878
RPS18	73325.41609	0.368844987	0.006655	0.02916
PDSS1	383.7344465	0.368792953	0.001327	0.00888
SLC3A2	24270.80777	0.368322262	0.002674	0.01481
CCDC109B	1487.71994	0.368233461	0.003966	0.01986
RNF141	3865.105939	0.368112853	0.001491	0.00963
NEDD4	1555.221656	0.367401114	0.00659	0.02898
NOP9	2976.630273	0.36585035	0.000162	0.00182
PHLDB3	1464.100256	0.365713118	0.010154	0.03997
SNX6	4289.751506	0.365604252	7.37E-05	0.00099
TWF1	4299.64855	0.36536531	0.000937	0.00682
LRRC59	11496.81767	0.36531486	2.56E-06	7.3E-05
WDFY2	2335.615512	0.364694848	0.000539	0.00454
ITGB1BP1	2754.836922	0.363702947	3.76E-05	0.00058
TOB1	2916.582231	0.363404674	0.002498	0.01401
ALG14	465.1842491	0.362855322	0.000281	0.00279
INTS4	1192.353343	0.362791844	0.00088	0.00654
CAT	2707.100089	0.36215643	0.007766	0.03285
MRPL22	1020.48645	0.362053159	0.000542	0.00456
TMOD3	7271.529152	0.361971263	0.001169	0.00805
CRIM1	4846.175441	0.361746121	0.006328	0.02818
FRMD4B	2366.145204	0.361433871	0.002213	0.01288
BCL2L2	3336.08325	0.36111665	0.000456	0.00401
KLF7	3514.467913	0.36077911	0.012635	0.04706
KATNBL1	1363.072811	0.360634231	0.000196	0.00211
CIAPIN1	3128.799787	0.359910052	7.79E-06	0.00017
EIF3I	12944.58591	0.359681012	8.74E-05	0.00111
TMEM194	579.9380249	0.359621358	0.002538	0.0142
FARSB	3146.825028	0.358792348	0.000179	0.00195
MYO1E	5873.09626	0.358706455	0.001346	0.00896
FAM83H	17940.83312	0.358690852	0.000758	0.00586
AC093818	76.5371656	0.357841776	0.008255	0.03433

PRDX5	15435.96047	0.35713419	0.009745	0.03873
HN1	10124.09599	0.357077913	0.003491	0.01802
TRAPPC3	3678.062756	0.357014096	0.000154	0.00175
FBXL6	1587.918916	0.355954038	0.004179	0.02061
BLZF1	1729.739081	0.35573851	0.000133	0.00157
MAPK3	3701.815106	0.355596141	0.000426	0.0038
ANXA5	15598.84281	0.355593329	0.000572	0.00473
ACTB	347637.8094	0.355189958	2.58E-05	0.00043
LRTOMT	303.8875841	0.354894944	0.009477	0.03799
MAP2K4	2297.379319	0.354789882	1.13E-05	0.00023
DLGAP4	7137.483704	0.354650165	0.000327	0.00313
UBE2V1	556.3401043	0.354516904	0.000204	0.00217
BAK1	3796.798307	0.35415474	0.006195	0.02771
EBNA1BP2	5035.922693	0.353708613	0.00125	0.00849
PICALM	10154.25917	0.353400181	0.000725	0.00565
SLC35F2	1756.653556	0.352969125	0.008846	0.03611
SPCS3	6345.447908	0.352963962	8.95E-06	0.00019
CCDC85C	3430.781547	0.352489356	0.00445	0.02163
PKMYT1	2088.705727	0.352002938	0.01081	0.0419
RFX2	876.2039006	0.35189996	0.003543	0.01824
ZCCHC6	3024.377128	0.351726722	0.002424	0.0137
LYRM1	824.7334215	0.351713043	0.001864	0.01133
MTFR1	2239.472615	0.351698547	0.000708	0.00555
IFNGR1	7992.783852	0.351368269	0.002239	0.01299
CDC42EP2	893.5790149	0.35129779	0.006411	0.02844
CYCS	8650.791174	0.351262624	0.000984	0.00708
ANKEF1	991.4463861	0.350030498	0.006702	0.02933
TPT1	114943.4785	0.349445509	0.000865	0.00646
SLC25A39	11342.98068	0.348939058	0.000306	0.00296
CORO1C	12654.55465	0.348934937	0.000134	0.00158
MRPL15	3239.143508	0.348904496	0.000803	0.00611
BTBD3	1405.294486	0.34809895	0.001206	0.00826
SHCBP1	1122.239152	0.34800962	0.00311	0.01653
HERC4	2283.164313	0.34795744	2.67E-05	0.00044
CCNC	3235.102393	0.347695102	0.000122	0.00147
MINK1	11319.2394	0.347600065	0.000455	0.00401
RAB22A	4833.611767	0.347596607	0.00014	0.00163
RPL29	22680.43755	0.34689664	0.01338	0.0491
VMP1	7947.873258	0.346766506	0.002177	0.01275
SLMO2	4386.145497	0.346736211	0.000142	0.00165
KDM1B	1943.007384	0.346573256	0.001527	0.00979
RPE	1879.557914	0.346315857	1.51E-05	0.00029
ZNF841	406.7542197	0.346211959	0.008685	0.03558
CSNK1A1	9161.506814	0.345511654	0.003847	0.01941
ABCE1	5023.83272	0.345295737	0.000104	0.0013
PPP1R14B	7706.147694	0.345060691	0.009633	0.03844
ENO1	140510.1277	0.344639718	0.000551	0.00461

PAQR4	1385.207139	0.34434294	0.013667	0.04991
MAST4	4740.535508	0.344223154	0.007981	0.0335
POLD3	1207.156131	0.343851125	0.000895	0.00658
RPL5	38641.26949	0.342992832	0.000651	0.00522
REXO2	2653.226679	0.342878215	0.001432	0.00941
ITGB3BP	566.0811831	0.342739957	0.001887	0.01144
NAA20	5307.497593	0.342246296	0.0041	0.02035
NPM1	28163.17602	0.342223563	0.001047	0.00744
SRD5A1	2706.277185	0.341971872	0.00843	0.03479
ZCCHC4	367.7926355	0.341678185	0.000169	0.00187
KLHL18	1220.012384	0.34163271	0.000446	0.00395
EIF4E2	3820.602721	0.341525094	0.000786	0.00602
TNFRSF10I	4047.958584	0.340186815	0.009342	0.03761
RWDD4	644.889827	0.34011616	3.68E-05	0.00057
BICD2	12300.37752	0.340115796	0.007161	0.03083
PRELID1	3025.618768	0.33976614	0.002334	0.01334
EXT2	7320.482105	0.339601061	0.011175	0.04295
TIMM8B	1794.664692	0.339204997	0.01335	0.04901
C3orf52	648.993738	0.338959	0.012986	0.04803
RER1	5545.067544	0.338923134	0.000118	0.00143
CLPB	1254.026154	0.338791941	0.008058	0.03371
GSKIP	1760.791038	0.338744285	0.000258	0.00262
PHACTR2	1682.670676	0.338287095	0.010017	0.03957
CDC25B	9369.923249	0.338284215	0.009992	0.03949
C9orf85	595.0996786	0.337749722	0.000794	0.00606
UBE2D3	14105.90554	0.337305446	1.21E-06	3.9E-05
CPNE1	8380.988359	0.337057892	0.006936	0.03008
OSTC	4477.086922	0.336915402	0.000645	0.00517
TRMT112	8059.785316	0.336839242	0.002872	0.01557
HN1L	9467.650576	0.336709463	5.04E-05	0.00073
SHC1	11334.62746	0.336638296	0.00141	0.0093
YAE1D1	430.9482269	0.336203118	0.000796	0.00607
FARP2	2178.129954	0.336144018	0.00106	0.00751
VAMP3	6733.471692	0.335896088	0.00012	0.00145
AMD1	5746.25194	0.335798335	6.56E-05	0.00091
NOP16	1813.987049	0.335662007	0.008424	0.03479
RIMKLB	1128.201281	0.335654871	0.007738	0.03275
MFSD10	4821.021967	0.335614365	0.012728	0.04732
NUP35	725.1253522	0.335148366	0.002028	0.01208
ACTR5	712.9249588	0.335043718	0.000587	0.0048
ALDH4A1	2087.188975	0.334237554	0.008931	0.03637
NDUFA12	2212.486223	0.333955497	0.000626	0.00507
MAPK14	3933.907084	0.333531737	2.58E-06	7.3E-05
XRCC4	490.5345513	0.333434842	0.001012	0.00725
EPS8L2	6387.410051	0.333297507	0.008072	0.03375
RP3-523K2	2522.431358	0.333016179	0.010969	0.04233
GART	5031.818752	0.332812536	2.85E-05	0.00047

EHD4	5922.741594	0.332380451	0.0008	0.00609
SRP72	6501.85826	0.332270755	2.87E-05	0.00047
GNAI3	7902.715994	0.331824271	0.000128	0.00152
DBNL	10273.68622	0.331777078	0.00036	0.00335
SLC38A1	10628.61515	0.331482424	0.004244	0.02088
NDUFAF2	700.6305939	0.331130049	0.012263	0.046
CPTP	2625.820221	0.330910755	0.003904	0.01963
RBX1	3505.825576	0.330721597	0.009829	0.03895
LPIN3	1154.901553	0.330248695	0.012221	0.04591
MRPS18C	748.7788748	0.329806025	0.001672	0.01038
ATL3	6752.873879	0.329773228	0.005155	0.02412
WDR4	1095.45283	0.329686923	0.008058	0.03371
FBXO3	2282.914353	0.329246416	0.000485	0.00419
MLKL	950.4120458	0.329241256	0.009462	0.03794
GPR180	1002.00352	0.329132697	0.000796	0.00607
SPOPL	1708.510191	0.328949851	0.000495	0.00425
ATP5J	3409.087829	0.328796223	0.001335	0.00891
NSUN2	6283.188361	0.32862447	0.000957	0.00692
MKKS	2792.881392	0.328247753	0.011006	0.04241
C4orf3	7713.289831	0.328161609	0.004144	0.02048
EIF2B2	2052.086881	0.327212197	0.000286	0.00282
PDLIM1	17831.35227	0.326646152	0.004979	0.02352
MED8	2655.82436	0.326494293	0.000243	0.0025
GPRIN1	1153.290817	0.326347734	0.009672	0.03855
ALAS1	2754.471084	0.32582912	3.27E-05	0.00052
RCOR1	4874.831076	0.32530668	0.004152	0.02051
MRPL1	981.6385299	0.325051785	0.008736	0.03573
TRNAU1AI	992.6456527	0.324950261	0.003478	0.01797
ANXA2	93625.64846	0.324778259	0.003162	0.01671
FEM1B	6049.257056	0.324671763	0.000425	0.0038
HSD17B12	3970.643674	0.324403898	0.009448	0.0379
TDG	1789.260676	0.324390681	0.000143	0.00165
SLK	7651.189118	0.324158763	0.002159	0.01267
SEC23A	3870.914158	0.324117708	0.00312	0.01656
ITM2B	37647.24341	0.323762859	0.002324	0.01331
RPL17	2010.282757	0.32339498	0.005816	0.0265
FAM96B	3217.76824	0.323363687	0.004493	0.02178
ARNTL	765.331673	0.323353904	0.00529	0.02465
MAPKBP1	3866.86849	0.323188319	0.004916	0.02333
GRAMD3	799.3044641	0.323110748	0.010251	0.0403
IL4R	8174.658904	0.322647011	0.010386	0.04075
DDX19B	680.3274658	0.322580511	0.003449	0.01786
EED	1013.398209	0.322157833	0.00029	0.00285
SNRNP35	1048.063593	0.321960654	0.006812	0.02965
UTP11L	2152.248156	0.321668339	4.21E-05	0.00063
FBXO45	3526.360616	0.321465521	0.009575	0.03826
FKBP14	1507.024458	0.321431797	0.011059	0.04258

KCTD5	3373.473388	0.321405291	5.11E-06	0.00013
PKM	138167.8934	0.321388242	0.00149	0.00963
TMEM126	791.2372385	0.321308109	0.011178	0.04295
PLS3	10423.52517	0.320941171	0.001477	0.00958
COX7B	5221.165387	0.320812437	0.008604	0.03534
CALM2	18083.55744	0.320807691	0.000207	0.0022
RAB8B	1354.041836	0.320679343	0.006388	0.02838
PTP4A1	10416.55602	0.320331559	0.003321	0.01734
FNTA	3298.600556	0.319993325	0.010506	0.04109
ATP6V1H	3491.304902	0.319293413	0.002447	0.01381
MALT1	2441.31017	0.318955201	0.000803	0.00611
BTRC	1576.065223	0.318846041	9.42E-05	0.00119
SNX18	1645.326173	0.318818197	0.003241	0.01701
BBS7	647.6767697	0.317901327	2.63E-05	0.00044
CALM1	24646.98104	0.317566359	4.73E-05	0.0007
TAF12	1189.933778	0.317259579	8.2E-05	0.00107
PTPN12	6135.061097	0.316928961	0.001687	0.01044
PLEKHM1	2820.024061	0.316530722	0.002276	0.01315
MAEA	3959.616959	0.315898932	0.001251	0.0085
GTPBP2	3192.226628	0.315700049	0.00029	0.00285
SKA3	670.4594906	0.315508432	0.008282	0.03438
SNX16	696.582867	0.315494198	0.003452	0.01786
RALA	5191.34008	0.315475868	1.58E-05	0.0003
EHBP1L1	8567.347749	0.314681628	0.006004	0.02711
PDXK	8571.376003	0.314543855	0.006619	0.02905
BRI3BP	1906.65566	0.314415921	0.005508	0.02551
C11orf73	1352.141332	0.314337207	0.003089	0.01647
ANO10	1483.263907	0.313798077	0.000977	0.00703
PDCD6	5430.922451	0.313779593	0.005182	0.02422
TBC1D23	2663.87795	0.313437332	0.000788	0.00603
ESRP1	8609.35183	0.313064538	0.006429	0.02849
NAPG	1874.263104	0.31274471	0.002725	0.015
PCMT1	3893.507499	0.312677468	0.000267	0.00269
SH3GL1	9284.381544	0.312465121	0.000269	0.0027
THOC7	2064.754645	0.311865268	0.001031	0.00737
VDAC1	12557.67149	0.311819773	0.000145	0.00167
EEF1B2	12988.52394	0.311567555	0.009261	0.03737
NAA15	3840.007098	0.311386617	0.000285	0.00281
COMMD8	1175.321573	0.311082909	0.003955	0.01983
TMEM106	3537.757684	0.310630073	0.004408	0.02149
PWP1	3512.497445	0.310418582	3.36E-05	0.00053
TOLLIP	6036.304075	0.310157044	0.003739	0.01901
OSBPL10	1216.32864	0.310118077	0.010343	0.04059
TMEM50A	6445.373684	0.310111652	1.21E-05	0.00024
ARMC1	2541.108662	0.309726157	0.00048	0.00415
AURKA	1803.049384	0.309618624	0.01304	0.04814
EXT1	8693.296517	0.309279114	0.00955	0.03817

LPPR2	1730.706201	0.309161338	0.012725	0.04731
LYAR	1465.418084	0.309154009	0.008905	0.0363
KIF13A	5553.020267	0.309086355	0.005798	0.02644
MRPS6	1901.450371	0.308961197	0.010787	0.04187
BCL2L1	5250.626853	0.308828997	0.000336	0.00319
DNAJC5	9818.791419	0.308767025	0.001331	0.0089
UBE2E1	2805.892758	0.308660892	7.82E-05	0.00103
ZFP36L2	9819.097124	0.308481356	0.006723	0.02939
GRPEL1	2588.017857	0.30804832	0.002587	0.01441
MRS2	1437.532518	0.307627389	0.000159	0.00179
RAB9A	1455.516991	0.307462871	0.000974	0.00701
RPS13	17777.28218	0.307139149	0.010746	0.04177
CAMSAP2	4672.58222	0.307132992	0.002297	0.01323
EXOC6B	4395.096698	0.306805807	0.008414	0.03476
MYD88	4032.33956	0.306229468	0.00715	0.03079
AKTIP	949.1894736	0.306203354	0.001633	0.01022
KLHL2	1060.790263	0.306174553	0.005011	0.02363
KIF5B	9821.167149	0.305902579	0.001082	0.0076
COMMD9	1931.536944	0.305795363	0.002323	0.01331
CDCA4	3110.365609	0.305709324	0.002763	0.01513
PSMD13	8258.208912	0.305257103	0.007414	0.03174
RNH1	13929.44258	0.305112497	0.003476	0.01796
RPS2	57647.57536	0.305058204	0.008726	0.0357
MRPL13	2460.500488	0.304759082	0.004299	0.02112
PSMA3	5288.207773	0.30437676	0.002571	0.01435
CDS1	2238.948798	0.303902435	0.011829	0.0449
ERH	5005.144743	0.303438961	0.001654	0.01029
MRPS23	2304.808189	0.30317629	0.001092	0.00766
CEP57L1	360.3655763	0.303099678	0.001216	0.00831
BRIX1	2516.347914	0.303002411	0.009331	0.03757
RTN4	18962.07352	0.302309376	0.000139	0.00162
PDLIM5	7522.900779	0.302214947	0.003729	0.01897
KCTD10	4522.741911	0.301953659	4.75E-05	0.0007
MTCH2	5145.386217	0.301819222	0.000159	0.00179
OTUB1	7809.707204	0.301799952	0.002452	0.01382
GNPNAT1	1935.880174	0.301572438	0.005213	0.02433
AK2	6654.480755	0.301554532	0.000329	0.00313
CFL1	65513.64384	0.301408495	0.001591	0.01004
UEVLD	1362.228354	0.301344785	0.000611	0.00497
LSM12P1	91.24827262	0.301327986	0.004435	0.02157
PIGU	1813.983409	0.301261865	0.008542	0.03516
TMED7	4183.403898	0.300581496	0.001632	0.01021
KARS	10232.60299	0.300553217	0.000244	0.0025
MED4	1993.791844	0.299969296	6.67E-05	0.00092
PDCD6IP	7438.744237	0.299765507	0.000837	0.00629
MLLT4	5273.420382	0.299242885	0.005891	0.02673
VAPA	9640.293982	0.299212107	0.002188	0.01279

ARPC5L	4518.063109	0.299106104	0.012846	0.04762
IDH3A	1795.324493	0.299083871	0.005016	0.02363
ERI1	822.7963884	0.298812493	0.002238	0.01299
ARAP1	6459.46205	0.29875837	0.00494	0.02338
ACOT9	2012.946545	0.298609421	0.003145	0.01666
PAK1IP1	1288.663788	0.29855439	0.002244	0.013
GEMIN2	515.5569945	0.29854465	0.006042	0.02724
API5	5479.354067	0.298349089	0.001033	0.00738
STRN	3010.828227	0.298317058	0.004417	0.02151
RSL24D1	3973.925037	0.297958344	0.001913	0.01157
EZR	30574.36743	0.297762325	0.002938	0.0158
CWC15	2288.033609	0.297446012	0.005545	0.02565
EIF4A3	7539.156717	0.29682039	0.000464	0.00407
DNAJC8	4843.771184	0.296481825	8.12E-05	0.00106
ZFYVE21	2389.184132	0.296336298	0.000115	0.0014
MNAT1	1114.411683	0.295783069	0.003393	0.01766
TNFAIP8	2149.517144	0.295694104	0.00641	0.02844
C18orf21	668.2016083	0.295285446	0.008918	0.03633
RPS23	21455.47029	0.295113469	0.007958	0.03346
SERBP1	22312.17548	0.295104108	8.04E-05	0.00105
ARPC3	11713.40473	0.294723003	0.001164	0.00803
CTNNBL1	3205.508523	0.294360736	0.001323	0.00887
MORF4L2	11767.92846	0.29391901	0.000368	0.0034
NPTN	6817.354708	0.293816451	0.000549	0.0046
FAM177A1	2726.737131	0.292571508	0.000274	0.00274
HARS	3557.961544	0.28984077	0.000396	0.00361
MRPS31	745.488675	0.289769918	0.001157	0.008
MTRR	1615.419621	0.289337796	0.00476	0.02281
RANBP10	1847.714916	0.289297632	0.000311	0.003
SLC25A32	1735.59111	0.288675287	0.000449	0.00396
STX7	2304.689381	0.288577391	0.000494	0.00425
SP100	4320.983064	0.288081648	0.012448	0.0465
N4BP1	6589.75871	0.287607734	0.005672	0.02605
YWHAB	22694.08876	0.287464977	0.000337	0.0032
ELMSAN1	3611.788463	0.286315832	0.011547	0.04404
EFTUD1	1593.826902	0.286296377	0.003584	0.01839
HCCS	1586.899357	0.285786532	0.001301	0.00876
KPNA2	10012.01753	0.2852157	0.003028	0.01618
RARS	4096.615591	0.285180672	0.000339	0.00321
PHF11	1146.607149	0.285112369	0.012558	0.04685
COG4	4047.16392	0.284699833	0.000894	0.00658
DUSP22	2038.414309	0.284402326	0.007894	0.03328
EIF2S2	6646.008514	0.283942727	0.002088	0.01236
RPS6KB1	1930.087278	0.283647887	0.00029	0.00285
SNRPB2	2633.147852	0.283560656	0.011222	0.04309
SEP15	6773.735291	0.283305828	0.000424	0.0038
PSMA5	5857.009969	0.28317009	0.003108	0.01653

BCS1L	1035.940253	0.282685005	0.0064	0.0284
DNAJA2	4512.34584	0.281793012	2.9E-05	0.00047
CCT2	10019.59547	0.281676367	0.003395	0.01766
DNAJC24	389.1992749	0.281581411	0.002761	0.01513
FAM126B	1129.792589	0.281554809	0.010081	0.03976
RCC1	3588.868397	0.281546394	0.001286	0.00869
LCMT1	1717.771634	0.281531754	0.006129	0.02749
ACTR1A	8875.240244	0.281386637	0.000941	0.00684
TMED2	15505.35697	0.281152588	0.000599	0.00488
DENR	4727.495904	0.280884577	1.72E-05	0.00032
TNKS1BP1	23221.4305	0.280660116	0.008428	0.03479
RAC1	21822.53215	0.28060252	0.000422	0.00378
TMCC1	2701.757511	0.280391802	0.002816	0.01539
E2F4	4707.303222	0.279828687	0.000346	0.00325
MTHFD1	5311.197963	0.279804507	0.00521	0.02433
PMM2	1033.7799	0.279600544	0.00446	0.02167
CNKSR1	1648.217296	0.279392512	0.009035	0.03664
HSPA8	68380.38243	0.279140673	0.004222	0.02079
PFDN1	2709.334142	0.278744676	0.000342	0.00322
SRPK2	3385.805659	0.278502963	0.006937	0.03008
NAE1	2450.478109	0.278501065	0.001642	0.01024
ZHX3	1847.194099	0.278240689	0.007943	0.03341
TCFL5	977.5609076	0.277572054	0.005705	0.02613
ANKRD13C	1129.434061	0.277116617	9.92E-05	0.00125
MTF1	1843.624141	0.277034886	0.009458	0.03793
NSMCE2	1190.344502	0.276796267	0.004175	0.02059
GABARAPL	3169.220166	0.276680114	0.00036	0.00335
GSPT1	9395.121092	0.276400415	0.000143	0.00165
PPP2CA	8296.294206	0.276318257	0.000132	0.00156
ATP5C1	7342.904487	0.276185116	0.004462	0.02167
MRPS36	689.399892	0.27599176	0.004407	0.02149
MED6	1305.346786	0.275474205	0.000524	0.00444
SMYD2	2305.42693	0.275427106	0.000897	0.00659
TMEM33	4587.505653	0.275376481	0.007656	0.03254
PSMD6	3062.116283	0.275274913	0.000209	0.00221
RPL6	34632.11982	0.274948291	0.010081	0.03976
NOC2L	7891.335167	0.274874619	0.00408	0.02029
TIMM23	3052.874123	0.274622278	0.001361	0.00905
FAM49B	5084.836177	0.274504364	0.000872	0.00649
C1orf52	778.2417515	0.27416048	0.000949	0.00688
ZNRF2	1257.665226	0.273739968	0.002195	0.01281
ERP44	5260.836823	0.273639302	0.001259	0.00854
USP32	2363.290431	0.273444065	0.002726	0.015
MED19	803.0028038	0.273355242	0.005015	0.02363
NIT1	1505.636402	0.273268029	0.001983	0.01187
YWHAQ	20985.52458	0.273253102	0.004069	0.02027
ARFIP2	2911.330104	0.273070058	0.001746	0.01072

GTF2F2	1814.554771	0.272983731	0.007715	0.03271
KIAA1033	2701.290439	0.272847925	0.004536	0.02194
PAWR	3789.460031	0.272560053	0.003792	0.01921
MED18	869.9382192	0.272559005	0.007889	0.03327
YARS	7499.916881	0.272430008	0.000556	0.00465
TCEA1	3919.969318	0.272258103	0.003873	0.01951
AKIRIN1	3187.874664	0.272106453	0.001911	0.01156
GARS	9421.938093	0.271899324	0.001525	0.00979
RABGGTB	2209.892489	0.271850072	0.005369	0.02495
MEMO1P1	94.73935031	0.271617981	0.012632	0.04706
FUCA2	2823.359866	0.271559006	0.008992	0.03653
UQCRC2	8356.305086	0.270474536	0.000616	0.005
ACTR10	2871.87692	0.270037392	0.001246	0.00847
PPIB	13665.93933	0.269990436	0.006781	0.02957
APIP	599.1028819	0.269697522	0.012773	0.04745
LRRC42	2419.475791	0.269515786	0.001839	0.01121
PGM3	2094.320654	0.269172438	0.001979	0.01186
MED21	1693.824845	0.268842011	0.004124	0.02041
CNIH1	4748.613851	0.268403367	0.004005	0.02003
LRRC40	796.6706742	0.268206486	0.004799	0.02296
ZMPSTE24	3990.302226	0.267866162	0.000683	0.00541
UBE2K	5209.240326	0.267840332	0.000837	0.00629
ADPRHL2	2575.83622	0.267793546	0.00737	0.03157
GTF2B	1320.394707	0.267754427	0.000325	0.00312
POLR3B	924.6499081	0.267589193	0.001381	0.00914
ALG5	1292.153633	0.267451133	0.006422	0.02847
IFRD2	2455.190377	0.26698346	0.002066	0.01227
BCL10	1782.838784	0.266937466	0.006288	0.02806
RNASEK	384.1908645	0.266265109	0.005156	0.02412
LRRFIP2	2459.040485	0.266138117	0.001326	0.00888
FPGT	703.4481432	0.266040801	0.002859	0.01554
NSA2	3060.053301	0.265871709	0.005536	0.02562
TINF2	3428.138703	0.265737411	0.001179	0.00811
SGMS1	2084.425989	0.265735196	0.0079	0.03329
COMMD1(	690.9034791	0.265630701	0.008571	0.03521
EXOSC8	1012.79006	0.265599434	0.013655	0.04989
FBXO22	1430.954462	0.26501488	0.00131	0.0088
PPP1R21	1363.170972	0.264872992	0.00036	0.00335
NFS1	1394.95115	0.264480068	0.003115	0.01655
LEO1	1889.763255	0.263851186	0.001593	0.01004
VTA1	3285.206211	0.26376539	0.000337	0.0032
ZNF416	335.973896	0.263375469	0.012259	0.046
PPT2	591.3459191	0.262810447	0.011394	0.0436
JKAMP	2038.550607	0.262681526	0.002284	0.01319
CLP1	888.5901188	0.262393398	0.000318	0.00305
SDHB	3821.768296	0.262311496	0.000386	0.00353
COG3	1483.600109	0.261871969	0.001522	0.00978

LTA4H	5091.447907	0.261356349	0.010969	0.04233
ZNF143	879.2398806	0.261109155	3.85E-05	0.00059
GTF2H1	2385.310715	0.260756797	0.000839	0.0063
CSE1L	8751.042287	0.26067398	0.001147	0.00794
TFDP1	6848.231016	0.260135195	0.012466	0.04656
MBD1	3673.541323	0.259917165	0.010816	0.04191
TMEM165	4923.138682	0.259701684	0.001545	0.00985
SNAP23	2053.410012	0.259685604	0.001582	0.01
TARS	8060.63966	0.25925921	0.007187	0.03091
TMEM57	1924.167966	0.259231888	0.001336	0.00892
HSBP1	6543.30182	0.259200569	0.006298	0.02808
KIN	543.40515	0.259146144	0.001344	0.00895
TMED5	2849.989598	0.259083865	0.004308	0.02113
ERCC6	878.6471232	0.259076511	0.011702	0.04451
TCHP	1254.750517	0.259048065	0.010524	0.04113
MRPL49	3797.794558	0.258999978	0.002717	0.01498
EIF3M	8154.351671	0.258938375	0.01072	0.0417
ASUN	1999.363257	0.258651024	0.004967	0.02347
VAPB	4097.94469	0.258423654	0.001582	0.01
MRRF	2035.623702	0.258293523	0.005582	0.02577
FAM114A2	848.8683123	0.257822985	0.003025	0.01617
OGFOD1	2735.335703	0.257753721	0.000248	0.00253
ITCH	3754.60603	0.257749526	0.003651	0.01867
RDH11	4651.056951	0.257565672	0.012272	0.04602
ESRRA	3700.863145	0.257535297	0.006281	0.02803
EIF3J	4569.752706	0.257422647	0.000271	0.00271
CWC27	1113.39021	0.257056822	0.001675	0.01039
ZFAND6	2821.40621	0.25702926	0.000353	0.0033
STK4	2571.604072	0.256558698	0.004317	0.02114
DHX15	6550.914887	0.256350019	0.000145	0.00167
ZNF823	244.7982246	0.256265579	0.011951	0.04521
AP1G1	6022.096832	0.255840872	0.003284	0.01719
TANGO6	1051.651941	0.25573675	0.001594	0.01004
SBDS	4924.231914	0.255473928	0.004961	0.02345
PRMT5	3780.549098	0.255384616	0.01064	0.04146
TRAF3IP2	3852.098792	0.255305953	0.006555	0.02887
YKT6	10354.16153	0.254804778	0.005111	0.02398
SMN1	218.7491366	0.25479117	0.011646	0.04434
RAP1A	3041.811702	0.254691285	0.002477	0.01393
RAB5A	2633.736571	0.254554756	0.003007	0.01609
ARF6	12295.06945	0.254341981	0.00586	0.02662
NUDCD1	1677.40403	0.254303702	0.007815	0.03299
RBMS1	4747.06016	0.254056716	0.001049	0.00746
CRYZL1	578.2765631	0.2536056	0.002601	0.01448
GTF2H3	2208.045687	0.253564341	0.004411	0.02149
FAM98A	2786.581514	0.253354858	0.000272	0.00272
STIP1	13041.67019	0.253196993	0.003434	0.0178

IPO5	8145.804701	0.253026802	0.007175	0.03088
ATP6V0A1	3521.973217	0.252791436	0.004665	0.02243
PSMC3	8902.443243	0.252449796	0.012142	0.04569
TMEM69	1711.900987	0.252314518	0.000451	0.00398
ABCD3	1978.849222	0.251801925	0.002314	0.01329
INTS6	2141.183835	0.251634512	0.004655	0.02239
GPN1	2751.987448	0.251573688	0.003612	0.0185
TOR1AIP2	4532.941295	0.251558784	9.46E-05	0.00119
RIC8A	6946.080449	0.251088156	0.004111	0.02039
NUP93	3475.824282	0.250792504	0.004593	0.02215
RAB1A	9601.062827	0.250355235	4.28E-05	0.00064
LRRFIP1	8725.083886	0.250182531	0.013048	0.04816
COPS5	3599.080306	0.250084879	0.002825	0.01541
FBXO46	1751.664956	0.250025066	0.010996	0.04241
UQCRC1	11197.05488	0.249915447	0.011277	0.04321
STAM	1644.668546	0.249844377	0.009512	0.03806
ELP4	710.671601	0.249717664	0.004582	0.02211
BCAP29	2782.058672	0.249344641	0.005042	0.02372
PPP1CA	13894.71929	0.249244962	0.010803	0.04189
ARHGAP10	1088.043333	0.24893258	0.006481	0.02867
RTCA	1759.888047	0.248799987	0.001935	0.01166
DPH5	850.5306233	0.248417178	0.007538	0.03219
HARBI1	258.5435532	0.247706263	0.013306	0.0489
SARS	8359.943373	0.247176355	0.009096	0.03683
CSNK2A2	4718.129957	0.247069037	0.001106	0.00773
MAP4K4	12102.54944	0.246755693	0.007789	0.03292
FKBP1A	14259.50484	0.246551928	0.011148	0.04289
MARCH7	4966.901484	0.246286225	0.001524	0.00979
PITHD1	3058.63661	0.245595125	0.005801	0.02645
ZRANB2	2398.370208	0.245125385	0.000726	0.00565
UQCC1	1578.676309	0.245070461	0.003591	0.01841
PTPN1	5842.917103	0.244974748	0.00926	0.03737
PEX2	1902.727813	0.244882871	0.007075	0.03059
CLPX	2861.52813	0.244769258	0.006944	0.03009
TPM4	41891.72543	0.24454829	0.00636	0.02829
PSMB2	9700.982368	0.244398393	0.004125	0.02041
MAPKAP1	5487.556231	0.244366059	0.000281	0.00279
YRDC	1251.000819	0.244311465	0.002181	0.01277
GMFB	3401.252459	0.244232912	0.00912	0.03689
RNF139	2375.708961	0.243623511	0.001195	0.0082
TRIP4	1531.829615	0.243594794	0.000402	0.00364
SUCLG1	3816.612919	0.243544295	0.009119	0.03689
DIAPH1	12351.14736	0.243230134	0.008489	0.03497
SHQ1	789.9666697	0.243202201	0.001211	0.00828
DUSP11	2094.238033	0.24314904	0.004839	0.02308
CTPS1	3523.09607	0.242679517	0.008563	0.0352
DHX32	2596.147795	0.242669018	0.001169	0.00805

VASP	6301.806414	0.242637307	0.00234	0.01336
PPP2R5E	3617.463155	0.242504945	0.002241	0.013
PHB	6138.194213	0.242391745	0.0071	0.03067
PSMC6	4146.096935	0.242006047	0.004775	0.02287
PLRG1	2849.275318	0.241468863	0.000522	0.00443
AAGAB	3401.015296	0.241422851	0.003241	0.01701
TRIM25	3847.532565	0.240986442	0.00925	0.03735
CRLF3	1193.250579	0.240771702	0.006998	0.03029
NDUFAF6	602.2849389	0.240684832	0.009508	0.03805
ZMYM1	621.7000558	0.240654685	0.002941	0.0158
PI4K2A	2921.39197	0.239816304	0.012876	0.04771
HNRNPC	20876.77043	0.239797627	0.0002	0.00213
ESD	3709.787121	0.239555935	0.013648	0.04989
CACUL1	4333.66872	0.239545066	0.00291	0.01569
SNX14	1922.586369	0.239187185	0.002244	0.013
SNX3	7390.843584	0.238881015	0.002034	0.01211
EMC4	3946.738356	0.23878749	0.001993	0.01191
TMA16	1006.294588	0.238698456	0.007554	0.03223
CISD2	1652.162606	0.23862132	0.006639	0.02911
MOB4	1595.27419	0.238168393	0.008545	0.03516
OSBPL2	3562.049943	0.237811628	0.008964	0.03643
NUMB	3855.144475	0.23745564	0.002357	0.01341
BTF3	20908.04096	0.237086818	0.008939	0.03638
DCAF13	3472.336419	0.23692902	0.010524	0.04113
OXSM	370.2012094	0.23658846	0.005547	0.02565
SAR1A	6181.500931	0.236383011	0.000373	0.00344
PPP4C	7345.423409	0.235991902	0.013055	0.04818
ALG1	1128.008713	0.235834462	0.001467	0.00954
MAPK1	8636.875916	0.235671387	0.012534	0.04678
RCAN3	1843.98591	0.235399238	0.004374	0.02135
RAB1B	13322.75775	0.235221356	0.008956	0.03642
TRPC4AP	5778.923186	0.235138556	0.001078	0.00759
UTP6	2562.333436	0.234203659	0.000237	0.00244
PSMD14	5061.761411	0.234034787	0.001814	0.01107
SEC22A	970.4768464	0.233948024	0.008005	0.03357
SRSF1	9197.843317	0.233929336	1.21E-05	0.00024
KIAA0391	290.4326563	0.233735327	0.004314	0.02114
LZIC	1318.22664	0.233462756	0.002753	0.01511
DEF8	2171.509843	0.233454542	0.004854	0.02313
GUF1	1447.37866	0.233173653	0.009649	0.03848
GNG5	3892.966069	0.233041964	0.005346	0.02487
CDK5RAP1	1375.818376	0.232970255	0.008764	0.03582
COA7	1678.222181	0.232689183	0.005574	0.02574
DFFA	3080.738986	0.232672547	0.002388	0.01355
RABEPK	1413.235556	0.23252006	0.011864	0.04496
DNAJC7	4658.250317	0.232334215	0.001659	0.01032
NDC1	2452.957948	0.23160641	0.006498	0.02871

CTBP2	3134.408155	0.230810162	0.002704	0.01493
CYB5R4	1238.300476	0.230635426	0.008795	0.03594
SRP54	3330.486714	0.229324637	0.002026	0.01207
UFM1	3271.716984	0.229297543	0.007649	0.03252
NUS1	1996.694926	0.229277553	0.0029	0.01566
LAMTOR3	1741.468078	0.229010761	0.002969	0.01593
KPNA3	3365.908674	0.227982858	0.010256	0.04031
LACTB	1219.65799	0.227595906	0.008258	0.03433
ADIPOR1	9356.991091	0.227113031	0.006806	0.02965
EXOSC10	3763.133216	0.227006253	0.003156	0.01669
SPAG9	6138.277807	0.226961823	0.010487	0.04106
VTI1B	3989.07859	0.226700216	0.002846	0.01549
SIKE1	1761.003919	0.22662092	0.003933	0.01974
ZNF598	4535.806056	0.226450607	0.009281	0.03742
PSMD12	4366.122932	0.226384201	0.000953	0.0069
STK3	1998.105813	0.225358979	0.007577	0.03229
HEATR3	1320.305796	0.225255031	0.002395	0.01356
COQ5	1187.657857	0.225144643	0.007764	0.03285
BTF3L4	2639.668245	0.225056799	0.003398	0.01767
MYCBP	1341.657508	0.225046076	0.006648	0.02914
UTP18	2225.541799	0.224663877	0.002735	0.01503
C10orf76	1961.381106	0.22442652	0.002864	0.01554
MPRIP	6999.120104	0.223077857	0.010538	0.04115
RAB7A	20729.89261	0.222810412	0.00319	0.01682
ARHGDIA	16345.15543	0.222548656	0.003843	0.0194
TAF1B	815.4034056	0.221704006	0.012222	0.04591
RSU1	3530.140817	0.22165373	0.012956	0.04793
DR1	4517.347137	0.22162443	0.002634	0.01462
ZNF706	4743.312965	0.221445359	0.008994	0.03653
MRPS22	2195.36927	0.221240394	0.013388	0.04911
ZNF35	325.1023539	0.220953727	0.007573	0.03229
RNF149	4751.149799	0.22084305	0.009495	0.03802
DDX52	2076.881851	0.220733509	0.000671	0.00533
MAPRE1	8641.453506	0.220559515	0.003188	0.01682
GNL2	3991.601487	0.219637294	0.005319	0.02476
ZCCHC10	751.0873149	0.219249344	0.006063	0.02729
MMADHC	5232.405882	0.218982434	0.002517	0.0141
H3F3B	40424.06292	0.21889461	0.007646	0.03252
MTRF1L	460.8179191	0.218594725	0.001684	0.01043
LTV1	1322.485031	0.218490974	0.008959	0.03642
XPO6	7243.699785	0.218408128	0.001539	0.00982
MRPL45	1806.185535	0.216553819	0.003964	0.01986
PIGH	662.1690621	0.216472018	0.010753	0.04179
FAM91A1	4405.316036	0.2160363	0.013228	0.04874
CCNH	893.472474	0.214875054	0.008847	0.03611
GMPR2	2491.321381	0.213642536	0.005968	0.02699
RHOA	22045.68921	0.213429395	0.001909	0.01155

CREB1	2379.877914	0.213350111	0.003491	0.01802
FASTKD2	1872.100573	0.213184322	0.006617	0.02904
RBM39	8062.533882	0.213033645	0.004859	0.02313
CDC27	3736.843995	0.212684678	0.001084	0.00762
PAFAH1B2	6376.64384	0.212479522	0.010624	0.04143
PKNOX1	1155.493729	0.211571309	0.000827	0.00623
EIF3E	15291.72512	0.210871219	0.013578	0.04972
RNF185	2080.217615	0.210760468	0.004258	0.02093
ALG2	1838.469894	0.210575289	0.0082	0.03417
TMEM167	3974.523829	0.210310807	0.003608	0.01849
UBA3	1880.499387	0.210127508	0.005817	0.0265
UCHL5	2044.878444	0.209682676	0.010462	0.04097
SEPT7	7783.590549	0.209650761	0.002561	0.01431
CAPZB	14997.15599	0.209369589	0.005106	0.02398
PPP6C	3939.570718	0.2076306	0.004959	0.02345
SEPT2	16145.35335	0.207502057	0.006603	0.02901
SPRYD3	4467.139401	0.207353257	0.00531	0.02473
FCF1	2985.153045	0.207254934	0.004118	0.0204
FTSJ1	3106.998381	0.206222313	0.011151	0.04289
WDR36	2157.340495	0.206156443	0.01228	0.04603
VEZT	3417.54722	0.206028269	0.009589	0.0383
PDHB	2077.581841	0.205894112	0.008134	0.03394
FAM204A	1638.723709	0.205865372	0.002298	0.01323
SAR1B	2523.325382	0.20549388	0.012337	0.04621
ETF1	8421.4592	0.203306826	0.003594	0.01842
INTS12	894.9989566	0.202447234	0.003341	0.01743
FAM104A	1206.800506	0.202255548	0.002245	0.013
AP3B1	3386.797334	0.20198669	0.013283	0.04885
RQCD1	3447.088269	0.201953516	0.004987	0.02354
PHF20	2486.675823	0.200765305	0.008432	0.03479
C11orf58	5815.652779	0.200275751	0.010282	0.04038
ATP5SL	2255.408306	0.19904985	0.002177	0.01275
SNRNP40	2431.992386	0.198751976	0.008288	0.03438
DLST	6553.476417	0.197620584	0.011484	0.04384
AIMP1	2520.921106	0.19693547	0.00401	0.02004
C6orf211	1144.661437	0.196827342	0.011013	0.04242
PPP1R11	3698.22494	0.196470451	0.002098	0.0124
LAPTM4A	15445.05755	0.195702123	0.013282	0.04885
METAP2	5474.664836	0.195688834	0.002551	0.01426
ELL	1722.649498	0.195240659	0.012887	0.04774
MEMO1	193.1141862	0.191579386	0.007674	0.03261
POLR3A	1847.086961	0.190592164	0.010962	0.04233
TCEB3	3854.085181	0.189954191	0.003735	0.01899
DDOST	13475.92953	0.188746877	0.005671	0.02605
PSME3	8191.420842	0.188584745	0.005428	0.02519
GABPA	1187.558164	0.187501109	0.009519	0.03808
BUB3	5411.664757	0.186526906	0.010996	0.04241

CRK	4795.498827	0.182532604	0.005822	0.02651
PSMD1	7892.799545	0.180714597	0.006942	0.03009
PSMD7	5645.795613	0.179921652	0.011231	0.04311
DDX1	6342.403753	0.174354381	0.012931	0.04787
SRSF6	6404.363656	0.174022963	0.011767	0.04473
SRSF3	12833.09758	0.171791788	0.010881	0.04208
PREP	3480.207371	0.171606119	0.01185	0.04492
DDX19A	2431.836273	0.167479854	0.006826	0.0297
HNRNPH3	5315.73007	0.166524961	0.011427	0.04369
RTFDC1	5166.265615	0.16609107	0.0122	0.04587
CCDC43	1365.692827	0.165756358	0.004326	0.02117
SZRD1	8513.036068	0.165638323	0.010824	0.04193
CHTF8	5268.829592	0.16124356	0.010033	0.03962
ZDHHC6	1529.297382	0.160496145	0.009722	0.03868
FAM175B	1178.060428	0.153125414	0.010437	0.0409
SNIP1	1061.905915	0.15153333	0.012793	0.04749
IST1	6659.764109	0.151202257	0.012732	0.04732
ZNF207	7989.484891	0.147775996	0.006038	0.02723
TIAL1	3778.514882	0.147290658	0.007627	0.03245
SRSF10	3921.885594	0.141059898	0.004607	0.0222
RAB5C	5399.555501	0.137737926	0.008858	0.03615
UBTF	7288.820158	-0.14512505	0.010648	0.04148
GABPB1	1511.406628	-0.147725801	0.013277	0.04885
SPOP	2339.93747	-0.149308867	0.004024	0.0201
NRF1	932.7100742	-0.152750971	0.001623	0.01018
RTF1	3498.547908	-0.158205852	0.007811	0.03299
VPS39	3531.822972	-0.158831637	0.011128	0.04284
NR1H2	4554.425923	-0.159455475	0.013264	0.04883
PCIF1	2584.424034	-0.159626986	0.010923	0.04222
HNRNPA0	8468.802485	-0.164299657	0.003727	0.01897
ZNF263	1543.840814	-0.166408691	0.01064	0.04146
C7orf55-LU	1658.692078	-0.172432168	0.009746	0.03873
ADO	1473.45916	-0.177209838	0.005307	0.02472
TMED4	4063.540506	-0.178020466	0.010872	0.04207
FXR2	2557.886764	-0.178820682	0.013625	0.04984
USP19	2594.843039	-0.179246411	0.007994	0.03354
UBR7	2191.183279	-0.179315812	0.002652	0.0147
COG7	1135.522787	-0.18048592	0.005899	0.02676
C2orf42	450.3385301	-0.184453992	0.002238	0.01299
FBXO7	4620.459742	-0.1874511	0.00079	0.00605
THADA	2356.599744	-0.188357895	0.004137	0.02046
MAU2	2858.926521	-0.190023535	0.008326	0.0345
CDK16	5825.617394	-0.192691451	0.007549	0.03222
KLHL12	1984.333903	-0.193056518	0.012998	0.04806
WDR48	1385.806922	-0.193104482	0.004369	0.02133
OS9	15360.87445	-0.193367243	0.00455	0.02199
UBE2Q1	4426.797273	-0.193742675	0.000815	0.00618

SMG7	6422.50915	-0.194353801	0.013317	0.04891
UPF1	7842.139701	-0.198982536	0.002838	0.01546
POLDIP3	4979.683454	-0.199034979	0.003447	0.01786
PI4KB	4632.469021	-0.200296441	0.012007	0.0453
USF2	5606.170038	-0.20091403	0.012112	0.04559
USP30	552.4056242	-0.201081848	0.005312	0.02473
DHX30	3564.223196	-0.202120274	0.001374	0.00911
FAM193A	1272.665081	-0.206907311	0.007905	0.0333
ZNF174	472.2620753	-0.210460484	0.007982	0.0335
ABCF3	4508.945582	-0.211325454	0.010507	0.04109
EPC1	1052.37584	-0.211474892	0.002828	0.01541
COG2	1341.125822	-0.212396026	0.000742	0.00574
ZNF324B	256.3846318	-0.21317762	0.012073	0.0455
ATXN2L	7310.446025	-0.215277444	0.006102	0.02743
NAGA	2544.315757	-0.216239123	0.007303	0.03133
SMAD4	2297.269587	-0.217864839	0.006717	0.02938
DCAF16	1301.038605	-0.218787779	0.012081	0.0455
PTPN9	2183.83921	-0.219898051	0.006126	0.02749
SNAP47	1782.792137	-0.219950698	0.003496	0.01802
ARID1B	4179.729226	-0.220211058	0.009444	0.0379
SETD1B	2626.6215	-0.220913539	0.009638	0.03845
CEP63	1110.416091	-0.221080505	0.003062	0.01634
B4GALT3	2492.598576	-0.221534988	0.004852	0.02313
GON4L	1934.474519	-0.222121497	0.00438	0.02136
ZNF316	1862.706169	-0.222590762	0.009865	0.03907
SH2B1	1906.458164	-0.222635641	0.012076	0.0455
YY1AP1	2763.779703	-0.222691577	0.001034	0.00738
SETDB1	1774.837706	-0.222788471	0.005991	0.02707
C2orf68	1349.976958	-0.22377189	0.005728	0.02622
RP11-135F	193.5483389	-0.224038206	0.005429	0.02519
TMEM248	6047.551464	-0.224461083	0.000211	0.00223
CIC	5659.303129	-0.22472411	0.006133	0.02749
OTUD5	3591.758337	-0.225681189	0.002848	0.01549
TYW1	922.4554577	-0.226528969	0.00377	0.01913
CENPBD1P	1239.476663	-0.227319847	0.001559	0.0099
CSTF2T	1761.883133	-0.227623903	0.001062	0.00752
LIMK1	4495.030534	-0.227646058	0.013655	0.04989
ADAR	21560.23509	-0.228037091	0.011576	0.04413
LRCH3	1947.322639	-0.228121637	0.007349	0.03151
TMED1	641.6369128	-0.228304951	0.011371	0.04352
RBM48	537.2515763	-0.228493571	0.010148	0.03997
DIEXF	1605.154181	-0.229123871	0.002618	0.01455
ZFP62	728.8915652	-0.229300346	0.006956	0.03013
ANKMY1	416.1343337	-0.231695113	0.01087	0.04207
FIG4	663.3288123	-0.23273439	0.001625	0.01018
CCDC115	2156.010663	-0.232845307	0.001781	0.01091
PIP5K1C	3224.41856	-0.233004618	0.006047	0.02724

CRCP	2152.228916	-0.233155763	0.00271	0.01495
SIN3A	3895.372056	-0.233527138	0.004147	0.02049
SPG11	2921.817628	-0.233537563	0.008157	0.03402
CCDC22	1315.214939	-0.233853526	0.01216	0.04573
KAT7	2030.633815	-0.234703159	0.003662	0.01871
MSL2	2059.595528	-0.235709846	0.008643	0.03545
FANCL	632.3092129	-0.235813254	0.011255	0.04315
DCAF7	5848.848428	-0.235919864	0.003962	0.01986
USP42	1019.063401	-0.236382261	0.002289	0.0132
EHMT1	3538.173521	-0.236399932	0.002931	0.01578
IRF2BP2	7304.417157	-0.236426319	0.001313	0.00881
DSTYK	1427.462796	-0.23677172	0.002774	0.01518
MAP2K5	852.7859339	-0.237585701	0.001469	0.00955
L3MBTL2	1811.763891	-0.238474884	0.003585	0.01839
PRKRIP1	1326.223391	-0.23971897	0.013262	0.04883
SPECC1L	1658.975485	-0.239759563	0.002908	0.01569
WDR6	3774.621256	-0.240251932	0.004939	0.02338
ORMDL3	3112.799289	-0.240870605	0.008694	0.0356
MCM9	797.3348992	-0.241032599	0.005918	0.02681
TAF6L	711.8423705	-0.241537967	0.013556	0.04967
ZNF740	1788.90882	-0.241576244	0.00128	0.00865
EML4	3667.685737	-0.241947049	0.003251	0.01706
PLEKHA3	790.6827467	-0.24208534	0.003	0.01607
CYP20A1	999.6151247	-0.242494015	0.002755	0.01511
TBC1D25	1359.89284	-0.243212318	0.001075	0.00758
BRAF	1190.922541	-0.243852022	0.00196	0.0118
UBL4A	3625.059318	-0.244176305	0.008222	0.03425
FKRP	795.1300638	-0.24429497	0.002823	0.01541
SCAMP3	4023.575207	-0.24495563	0.002122	0.01249
WDTC1	3955.725666	-0.2454691	0.005668	0.02605
C7orf49	1521.969596	-0.246178803	0.002317	0.0133
ZC3H10	364.1897529	-0.247019295	0.00153	0.0098
NUCKS1	17024.94995	-0.247199619	0.004155	0.02051
KANSL1	2354.643686	-0.24873529	0.001288	0.00869
MAN2B1	4672.85242	-0.248737792	0.007112	0.03069
PHIP	2491.424189	-0.249045962	0.00825	0.03432
DEPDC5	743.8743189	-0.249781746	0.000415	0.00374
ABCC10	1639.483863	-0.250377565	0.008167	0.03405
FOXK1	5077.156773	-0.250886175	0.012388	0.04634
LIMD1	1256.404999	-0.251209602	0.005282	0.02463
TTC3	5807.664744	-0.251398236	0.007797	0.03294
KDM3B	3812.940788	-0.251457598	0.007215	0.03101
APBA3	860.1871911	-0.251935635	0.006569	0.02891
SEPHS2	3659.601655	-0.252039459	0.002906	0.01568
TOP2B	4945.221834	-0.252124164	0.003151	0.01667
RCOR3	1259.621256	-0.252492544	0.002195	0.01281
CHD3	9776.623941	-0.252965337	0.010727	0.04172

BAZ1B	6087.529903	-0.253110723	0.004194	0.02066
SRGAP2	1864.837743	-0.253405989	0.002746	0.01508
ZNF225	164.3660903	-0.254169966	0.010577	0.04128
CERS2	5114.284295	-0.254796184	0.012083	0.0455
ZNF189	727.1634868	-0.254929346	0.001728	0.01064
FAM200A	519.4457804	-0.255321151	0.008284	0.03438
KDM4B	2367.929609	-0.255323355	0.001506	0.00969
PPM1B	1869.178557	-0.255477631	0.000198	0.00212
SLC38A10	6607.294494	-0.25551954	0.002577	0.01436
GGA2	3749.419891	-0.255534848	0.001984	0.01188
CXorf40A	551.0778276	-0.255905671	0.000659	0.00527
CDKN1B	2318.574457	-0.257115799	0.005538	0.02562
EHMT2	3495.741757	-0.257955712	0.006094	0.0274
KMT2B	3399.640901	-0.258086709	0.003107	0.01653
SETDB2	451.9466765	-0.258522716	0.001059	0.00751
GMIP	1184.494139	-0.258533087	0.002362	0.01342
TAPT1	718.3980671	-0.258602155	0.001143	0.00792
ELK1	2042.637669	-0.259210007	0.000925	0.00676
NIPSNAP1	3574.686218	-0.260502797	0.007992	0.03353
CHERP	2724.829763	-0.261348777	0.001143	0.00792
C1orf216	872.195612	-0.262227292	0.003617	0.01852
ZNF227	405.9123162	-0.2622693	0.001206	0.00826
PPP1R13B	2087.269145	-0.26268373	0.008616	0.03537
TAB1	1657.521144	-0.2627406	0.000149	0.00171
VEZF1	2092.058661	-0.263297116	0.002397	0.01357
RNF113A	685.173437	-0.26411196	0.006271	0.028
METTL16	2005.266661	-0.264239418	0.009361	0.03766
MFSD1	4352.521733	-0.264605907	0.005457	0.02531
VAMP2	2241.115509	-0.264632967	0.009807	0.03889
SCMH1	1231.590291	-0.264751178	0.01144	0.04373
PAN2	1175.258377	-0.265191096	0.007523	0.03214
SUSD6	6056.373024	-0.265205468	0.00685	0.02978
PMS2CL	266.3614178	-0.265558886	0.005631	0.02592
NSUN3	433.6874594	-0.266189221	0.010529	0.04113
PHF21A	1830.66903	-0.26620419	0.002685	0.01485
RSRC1	1646.261791	-0.267405233	0.006812	0.02965
GLTSCR1	872.4970924	-0.267547726	0.00047	0.0041
GCC2	1131.378959	-0.267678406	0.002319	0.0133
CEP162	325.8522844	-0.267916105	0.004554	0.02201
TBL1XR1	9715.977105	-0.268124427	0.013397	0.04914
ELAC1	233.8647364	-0.268723478	0.009219	0.03726
TMC6	3584.03929	-0.269465743	0.00831	0.03445
GRIPAP1	2399.898424	-0.269527949	0.001027	0.00734
PIGM	725.3375202	-0.269791243	0.002964	0.01592
DNAJC27	205.9589886	-0.270215987	0.00337	0.01756
ANGEL2	912.8339444	-0.270521454	0.00048	0.00415
TRIM4	1689.166365	-0.271224266	0.004686	0.02251

MORC4	1739.243607	-0.272120903	0.007716	0.03271
TAF1	1944.75489	-0.272142091	0.009488	0.03801
NUB1	3652.882525	-0.272357389	0.008038	0.03367
CASP2	2347.822507	-0.272396562	0.000884	0.00654
ZNF746	1258.165961	-0.272779758	0.004538	0.02195
MDM4	1101.708018	-0.273277478	0.01198	0.04523
ADSS	3868.843576	-0.273339089	0.001768	0.01084
HMG20B	3849.619909	-0.274233656	0.009399	0.03776
RBM12B	707.7726322	-0.274249148	0.006872	0.02985
IKBKG	454.8324177	-0.274369322	0.007947	0.03342
SEPSECS	408.7696651	-0.274761669	0.001704	0.01052
LRRC37B	246.96534	-0.27487757	0.002005	0.01197
MIF4GD	1076.683839	-0.274996713	0.007098	0.03067
CENPC	761.0824993	-0.275004725	0.002679	0.01483
NAGLU	1974.439469	-0.275133372	0.001399	0.00924
MARCH8	1510.26303	-0.275697656	0.004862	0.02314
ATP6AP1	9109.384007	-0.275804119	0.000326	0.00312
DAK	2230.989844	-0.276532485	0.006162	0.02759
MSH6	3846.00734	-0.276534704	0.002536	0.0142
CIDECP	266.4556063	-0.276592423	0.008384	0.0347
ABHD15	980.5341042	-0.277151096	0.000437	0.00388
FOXN3	3682.589273	-0.277170616	0.01167	0.04442
NHLRC3	684.8886093	-0.277337021	0.002205	0.01286
TNRC18	7745.509942	-0.277674703	0.001653	0.01029
ZBTB41	1258.855329	-0.277700025	0.011161	0.04292
PYGO2	3002.212811	-0.277785548	9.67E-05	0.00122
ZBTB45	805.2940247	-0.278218229	0.000489	0.00422
GLTSCR1L	1021.783926	-0.278491518	0.007771	0.03286
C19orf12	1106.07668	-0.278796717	0.00232	0.0133
ZNF500	596.5146257	-0.278807096	0.000329	0.00314
PAXIP1	899.5244237	-0.27901075	0.006869	0.02984
KDM3A	3632.395818	-0.279107384	0.013288	0.04885
EPM2AIP1	965.9524647	-0.279486797	0.002701	0.01491
TYK2	3488.29858	-0.279952347	0.002936	0.01579
TRIOBP	1226.615164	-0.280208761	0.008936	0.03638
VKORC1L1	2869.801884	-0.28048764	0.001589	0.01003
ARHGEF2	2887.324919	-0.280500079	0.003941	0.01977
RNF216	3005.961204	-0.281034849	2.79E-05	0.00046
CDK19	1192.717907	-0.281474355	0.002121	0.01249
ZNF839	290.4399097	-0.281532061	0.011578	0.04413
DEAF1	1418.071223	-0.281605519	0.007876	0.03322
ADCK4	1436.141729	-0.281710861	0.00116	0.00802
ZSCAN21	234.8496586	-0.282028433	0.00387	0.01949
ZER1	3516.569868	-0.282299125	0.001745	0.01072
ARL8A	2581.222043	-0.282907041	0.00035	0.00328
RNF135	1182.300652	-0.283108198	0.002577	0.01436
TTC21B	589.719967	-0.283195856	0.004193	0.02066

CASP9	655.4469231	-0.283318112	0.001899	0.0115
ARAF	3505.158162	-0.283537744	5.23E-05	0.00075
PSAP	67409.7671	-0.283642752	0.001307	0.00879
DUSP28	139.0166604	-0.284436299	0.006703	0.02933
FOXP4	3039.334637	-0.284465617	0.005041	0.02372
ZNF507	1142.667257	-0.284646478	0.001368	0.00908
ZNF641	909.0886404	-0.285681224	0.000839	0.0063
S1PR2	643.37477	-0.285683364	0.010775	0.04184
AC074117	216.3885077	-0.285698298	0.01185	0.04492
FIZ1	782.907894	-0.286165684	0.000231	0.0024
SEPT11	4517.520929	-0.286858392	0.013015	0.04808
EIF3J-AS1	181.8826046	-0.286966225	0.004998	0.02358
ZNF397	740.0271772	-0.287076973	0.010162	0.03999
FAM98C	649.3238305	-0.287482954	0.012842	0.04762
PDIK1L	556.8445493	-0.287624843	0.004334	0.0212
INPP5E	830.2096185	-0.28855202	0.008092	0.0338
RP11-283I	274.3323357	-0.289061636	0.010269	0.04034
PPOX	483.3465247	-0.289276099	0.010635	0.04146
TMCO4	877.7077281	-0.289733432	0.012638	0.04706
RNF44	3151.577123	-0.289842319	0.003799	0.01923
RFX7	1347.292839	-0.290039987	0.006015	0.02714
WWOX	514.2415283	-0.290148364	0.010494	0.04108
DTNBP1	747.2248396	-0.290212469	0.007103	0.03067
RNF24	1472.420268	-0.291496626	0.007617	0.03242
KANSL1L	345.9615649	-0.291637588	0.001579	0.01
SPR	1724.865472	-0.291689377	0.0093	0.03747
ZNF780B	329.9159251	-0.291945698	0.011957	0.04521
SLC24A1	424.977765	-0.292137657	0.004965	0.02347
HEIH	789.2573431	-0.292526872	0.002805	0.01533
RFX1	959.8970581	-0.29278367	7.09E-06	0.00016
PIAS2	845.2620169	-0.292855001	0.006832	0.02971
ZNF550	305.3467859	-0.293067319	0.012694	0.04721
ZNF92	327.7721447	-0.293082328	0.003273	0.01715
TTC19	1587.762782	-0.294206684	0.001558	0.0099
GRAMD1A	2468.291261	-0.294599765	0.002901	0.01566
EMC10	7798.791274	-0.295151484	0.004011	0.02004
TBC1D17	1942.654131	-0.295364166	0.000907	0.00665
BRAT1	3658.385268	-0.295474848	0.004675	0.02247
ENTPD4	1923.989355	-0.295915789	0.00292	0.01573
ZKSCAN2	290.2825477	-0.296162299	0.007147	0.03078
MOAP1	910.3003335	-0.296824308	0.000574	0.00475
B4GALT7	1539.819221	-0.297285691	0.009851	0.03902
RRNAD1	1149.967342	-0.297495699	0.000759	0.00586
MAP3K10	850.5415738	-0.297640712	0.004124	0.02041
IQCH-AS1	170.245908	-0.298005878	0.00513	0.02406
ARHGEF11	3007.383914	-0.298419294	0.002762	0.01513
USP49	296.3211131	-0.298842704	0.008116	0.03388

POGK	3597.601345	-0.298889264	0.002728	0.01501
BCL7A	1404.231579	-0.299070589	0.001105	0.00772
VGLL4	2125.120435	-0.29917221	0.008284	0.03438
ALKBH4	699.2257663	-0.299257864	0.002082	0.01233
FECH	1114.773427	-0.299286326	0.007614	0.03242
CCDC28A	705.317467	-0.299508603	3.44E-05	0.00054
C11orf95	1019.561143	-0.300145597	0.003146	0.01667
TTC38	953.2927129	-0.300354673	0.003995	0.01999
LBR	2923.645695	-0.300920243	0.006864	0.02984
H1FX	7215.715293	-0.300963507	0.012936	0.04787
PRR12	2902.337475	-0.3012328	0.00071	0.00557
SEPN1	6715.963897	-0.301414106	0.000968	0.00697
NCKAP5L	1574.358517	-0.302113533	0.005963	0.02698
ZNF513	850.3955897	-0.30231952	0.000513	0.00438
ALG3	4563.915222	-0.302654991	0.005491	0.02545
TRIM52	228.3719557	-0.302672024	0.009276	0.03741
HCFC1	6353.113151	-0.302690829	0.000126	0.0015
CPSF4	1626.290233	-0.302870929	0.005968	0.02699
SMG9	1587.541467	-0.303387753	0.000718	0.00562
FAM122B	1664.327993	-0.303478887	0.002416	0.01367
ZXDB	562.398038	-0.303677134	0.00233	0.01333
DENND4B	2010.876002	-0.304625512	5.75E-05	0.00081
EIF2B5	3791.78745	-0.304944288	0.001218	0.00831
KCTD3	2632.340294	-0.305069519	0.006453	0.02857
RBM43	386.5397095	-0.305157161	0.009295	0.03746
RPRD2	2955.877347	-0.305376483	0.002348	0.01338
CTAGE5	224.5312057	-0.305385919	0.006671	0.02921
CABLES2	621.5028558	-0.305608645	0.005376	0.02498
GMCL1	1292.057437	-0.306317066	0.001138	0.0079
KPNA5	277.3502978	-0.306976712	0.003269	0.01714
AC005562	84.76158413	-0.306977802	0.006773	0.02955
MIA3	3192.545695	-0.307041359	2.43E-05	0.00042
C19orf54	669.2985786	-0.307056082	0.000569	0.00471
BRI3	3529.818287	-0.307064022	0.009599	0.03833
ZNF619	145.3167398	-0.307179346	0.003858	0.01945
TIRAP	281.0468227	-0.307272319	0.006444	0.02854
CHIC1	399.2629239	-0.307554807	0.005036	0.02371
ZNF786	503.0906884	-0.308331551	0.001624	0.01018
PARP1	9867.836815	-0.309002843	0.000131	0.00156
ZKSCAN5	880.949176	-0.309058365	0.000145	0.00167
TP53I13	1888.882987	-0.309175053	0.007229	0.03107
SEMA4C	2680.075981	-0.309350333	0.013	0.04806
FAM120A(	1876.801611	-0.309948288	7.79E-05	0.00103
CABIN1	3588.5067	-0.310279135	9.73E-05	0.00122
GTF2IP1	65.05477175	-0.310463041	0.011597	0.04419
POMT1	1070.760512	-0.310520738	0.012149	0.0457
SMAD3	6851.750836	-0.31060599	0.011696	0.0445

ZNF653	256.5765545	-0.310703359	0.004492	0.02178
PKD2	1060.431336	-0.31141259	0.011873	0.04499
TCEANC	99.06358895	-0.311479931	0.001682	0.01042
CUEDC2	1863.41581	-0.311826433	0.004303	0.02112
PHF10	2326.351552	-0.31217889	6.17E-05	0.00087
FBXO31	1903.059541	-0.312338244	1.3E-05	0.00025
BTG1	9874.063256	-0.313244556	0.006754	0.02948
MPND	895.6015054	-0.314211241	0.009611	0.03836
RERE	3990.728366	-0.314698402	0.000419	0.00376
HDAC6	1829.399034	-0.314843451	2.92E-05	0.00048
DENND1C	732.0176965	-0.31485178	0.011	0.04241
ZNF747	529.7079114	-0.315880276	0.000254	0.00259
BRPF3	1677.820521	-0.316446525	0.00639	0.02838
ZNF462	1435.151545	-0.316505281	0.007143	0.03077
SMPD1	1623.345346	-0.316546039	0.006927	0.03006
PSMD5-AS	606.5111378	-0.316684671	0.003299	0.01726
ZSCAN25	967.3733313	-0.316852711	0.000385	0.00353
MYPOP	491.8563924	-0.317546358	0.000248	0.00253
RFX5	2131.639928	-0.317558367	0.001098	0.00769
POGZ	2706.218999	-0.317722534	0.000153	0.00175
CXCL16	3626.442464	-0.318301871	0.009306	0.03748
CCDC121	95.26522864	-0.31847259	0.005175	0.02419
ZNF18	514.4429817	-0.318942515	0.000196	0.00211
TYSND1	2095.781684	-0.31925903	0.001635	0.01022
IFT22	824.2326558	-0.319309973	0.002804	0.01533
ZNF629	1630.361268	-0.319633494	0.000895	0.00658
RCN1	4354.974664	-0.320457727	0.005079	0.02387
TRMT2B	937.5771157	-0.321207258	0.001053	0.00748
PASK	520.6947365	-0.321968867	0.00784	0.03309
POR	6906.405907	-0.322116667	0.002393	0.01355
ERAP1	3734.810339	-0.322205696	0.010291	0.04041
PRKAG2	731.3201014	-0.322306826	0.006251	0.02791
POM121C	2382.650692	-0.322604963	0.000422	0.00378
SREBF1	9645.945093	-0.323018641	0.009374	0.03771
MZF1	543.3923749	-0.323093515	0.009024	0.03661
RP11-83A2	149.1290134	-0.323260803	0.00412	0.0204
RP11-326I	140.8766012	-0.324101498	0.00682	0.02968
WBP1	411.7046117	-0.324616062	0.002252	0.01303
ZKSCAN8	989.7067013	-0.324653799	0.002003	0.01197
PCCA	570.689471	-0.325865416	0.001827	0.01114
TMEM194	1581.090845	-0.326128739	0.007447	0.03187
ZNF25	352.7073135	-0.327204264	0.008032	0.03366
CTBP1-AS2	762.6315822	-0.327351101	0.00074	0.00573
RP11-226L	126.4184003	-0.32786258	0.006731	0.0294
TMEM109	7693.752479	-0.328230623	0.000236	0.00243
INTU	387.3255476	-0.328348176	0.006533	0.0288
SIX5	594.7515987	-0.328389271	0.000394	0.00359

TP53BP1	1787.772086	-0.328867636	0.002896	0.01565
C11orf84	1503.953799	-0.328876711	0.006051	0.02725
CNOT6L	1924.285803	-0.329165947	0.00067	0.00533
RUNX1	5322.656981	-0.32948093	0.001642	0.01024
OTUD7B	1762.738625	-0.329531443	8.56E-05	0.0011
MCM7	10367.73155	-0.329649658	0.005569	0.02573
ELOVL5	4285.37209	-0.329665109	0.01196	0.04521
DHX35	819.3298662	-0.329678046	0.007699	0.03269
ACYP2	391.516144	-0.329760366	0.003098	0.01649
PACS2	2978.232365	-0.329974636	6.3E-05	0.00088
ADCK1	453.8766008	-0.330002525	0.001425	0.00938
IFT140	908.0522214	-0.330185286	0.005697	0.02612
SLC25A40	747.162278	-0.330265604	0.000438	0.00388
INSR	2156.918083	-0.330378673	0.00409	0.02031
ZNF672	1816.231005	-0.330426589	0.000398	0.00362
AGO4	988.234804	-0.330747015	0.000706	0.00554
PELP1	4682.120693	-0.330778408	0.004307	0.02113
IFT172	808.1276794	-0.330934517	0.000728	0.00566
KLHL15	413.7365169	-0.331459364	0.003769	0.01913
PLXND1	4280.565412	-0.33169801	0.00993	0.03928
CHST12	1199.293993	-0.332127279	0.000705	0.00554
CERS6	2331.182173	-0.332273193	0.011899	0.04507
CCPG1	553.7587797	-0.332337311	0.005407	0.02511
BAIAP2-AS	712.4531639	-0.332523742	0.00514	0.02409
POLR3GL	751.794634	-0.333066662	0.000955	0.0069
AC093323	627.8164113	-0.33307027	0.007739	0.03275
MPC1	872.0528842	-0.333162089	0.001362	0.00905
KANSL3	2660.9493	-0.333362071	3.69E-06	9.8E-05
PRRC2B	9995.313938	-0.333454287	0.011621	0.04426
SUV39H1	788.0115118	-0.334500135	0.002391	0.01355
METTL21B	471.4880576	-0.334583812	0.000357	0.00333
ZMAT3	1573.149444	-0.334669127	0.004055	0.02021
RP11-785H	1396.810318	-0.334681466	0.0084	0.03473
ZKSCAN1	3842.804588	-0.33509644	0.001433	0.00941
MAN2B2	2941.066732	-0.335803703	0.001526	0.00979
COQ10A	262.9868953	-0.335812105	0.011234	0.04312
ATAT1	357.8986725	-0.335849046	0.006363	0.0283
CUL7	2362.619454	-0.336040642	0.001391	0.0092
CASD1	631.6196574	-0.336041719	0.001168	0.00805
CASC4	2940.106778	-0.336149528	0.000548	0.0046
CTD-2619J	301.7591581	-0.336156276	0.003929	0.01973
NR1D2	1891.944199	-0.33628964	0.005822	0.02651
TRIM56	3667.231673	-0.336477702	0.001393	0.00921
ZHX2	2126.372026	-0.336746337	0.005597	0.0258
NFATC1	1065.338908	-0.337042975	0.008334	0.03452
ACO2	4952.213067	-0.337112856	0.00056	0.00467
ZBTB26	332.2489	-0.337777303	0.000434	0.00386

ZNF766	820.4089033	-0.338474829	9.55E-06	0.0002
SLC25A38	1104.540156	-0.338706894	3.2E-05	0.00051
ZNF84	746.583679	-0.338804032	0.000731	0.00567
AUTS2	2607.962389	-0.338811879	0.00493	0.02338
ATXN7L1	422.0060279	-0.338952246	0.000299	0.00291
MTERF1	537.7671704	-0.339154106	0.000585	0.0048
SEMA4A	2543.907805	-0.339365394	0.001686	0.01044
ASH1L-AS1	83.56226313	-0.339421483	0.013159	0.04853
CBFA2T2	1631.083894	-0.339631666	9.33E-05	0.00118
LSG1	3676.383547	-0.339633543	0.000912	0.00669
ASPSCR1	1548.703248	-0.339929457	0.010718	0.0417
TCF7L2	1195.279137	-0.340713632	0.000508	0.00434
BCL9	1232.029139	-0.340849051	0.0014	0.00924
SPPL2B	1686.228787	-0.340871148	0.002308	0.01327
FAM20B	3457.816153	-0.341274271	0.000164	0.00183
DET1	140.9427328	-0.341822551	0.004317	0.02114
OPHN1	383.050227	-0.341887348	0.008639	0.03545
SHROOM1	388.4597459	-0.341906381	0.01129	0.04325
GIGYF1	2909.379205	-0.343160325	0.000891	0.00657
ZNF691	378.4100853	-0.343197016	0.000232	0.00241
PIK3CA	2130.071156	-0.343519159	0.002021	0.01205
LINC00476	129.2575097	-0.344005044	0.005363	0.02494
UNC93B1	2082.165906	-0.344133566	0.001587	0.01002
WDR81	2677.951269	-0.344476895	7.73E-05	0.00102
SAP30	357.73622	-0.344723704	0.007697	0.03269
FAM13B	777.8990794	-0.344884699	0.001261	0.00855
LGMN	6681.955613	-0.344889238	0.000847	0.00634
SLC25A35	212.0504354	-0.345060852	0.006586	0.02897
AC004381	197.6531499	-0.345070507	0.007324	0.03141
FBXW4	2605.035411	-0.345172961	0.00043	0.00383
SLC12A9	2526.331195	-0.34558305	6.39E-05	0.00089
TCTN1	716.015459	-0.34596712	0.008539	0.03516
XPC	1138.795424	-0.346140604	0.000333	0.00317
ARHGEF9	580.2925145	-0.346176131	0.009158	0.03703
PCGF5	1949.627791	-0.347305515	0.001035	0.00738
DENND4A	1364.430876	-0.347337617	0.000248	0.00253
CACFD1	1180.163686	-0.347484261	0.012781	0.04746
KLHL26	543.2759149	-0.347648734	0.001205	0.00826
GUSB	2873.991078	-0.348829301	0.000141	0.00164
PHF12	2084.951364	-0.349035245	1.77E-05	0.00033
TFEB	1410.148224	-0.349862746	0.005885	0.02672
KCTD17	844.5192561	-0.350130458	0.010391	0.04076
PPM1K	589.50831	-0.350305178	0.011546	0.04404
STARD7-AS	176.8782686	-0.350335952	0.000243	0.0025
P2RX4	834.8121564	-0.350927806	0.008031	0.03366
NBPF12	204.4344221	-0.351016902	0.002442	0.01378
C1RL	2370.160577	-0.351365365	0.001491	0.00963

ALDH3A2	6173.780888	-0.351569436	0.007117	0.0307
MAGED2	5914.337478	-0.352186906	0.000637	0.00513
PIGX	2237.890069	-0.352938373	0.011277	0.04321
ZNF414	506.0775662	-0.353071118	0.001504	0.00968
CBLN3	117.2492694	-0.353625323	0.009785	0.03884
MECP2	2598.594785	-0.353980267	2.39E-06	6.8E-05
SLC39A11	2301.180067	-0.354622696	0.000722	0.00564
RGL1	982.4166131	-0.35470037	0.011981	0.04523
B3GNTL1	182.6566922	-0.354727689	0.002604	0.01449
ZNF652	1366.04958	-0.354902142	3.18E-05	0.00051
NFIC	5435.194329	-0.354933503	0.001667	0.01035
CREBL2	1941.226405	-0.354999939	4.19E-05	0.00063
TECPR1	1369.304584	-0.355452982	0.000549	0.0046
ZNF575	101.9336723	-0.35558264	0.001556	0.0099
MED9	792.7788169	-0.355891748	4.72E-07	1.9E-05
ZNF273	300.3964663	-0.3559405	0.004985	0.02354
TMEM243	528.5031683	-0.356493462	0.000563	0.00469
DSTNP2	158.8956524	-0.35657702	0.008753	0.03579
ABI2	1551.964941	-0.356600092	0.000242	0.00249
APOBEC3C	3649.007403	-0.357272241	0.001724	0.01062
FAM122C	101.3802524	-0.357275353	0.001416	0.00932
TMEM260	905.3847547	-0.358506533	0.000403	0.00364
CROCCP2	583.8779154	-0.358635778	0.012689	0.0472
ZADH2	1212.621617	-0.358879921	8.6E-05	0.0011
TESK2	479.7777551	-0.358935671	0.001709	0.01054
ANKMY2	720.0198755	-0.359132051	5.1E-05	0.00074
MR1	2170.80609	-0.359426903	0.001582	0.01
TMEM132	7678.966908	-0.359623887	0.008649	0.03547
ZBTB44	2072.006204	-0.359876907	0.000542	0.00456
EZH2	1288.156283	-0.359897259	0.003909	0.01965
MEPCE	3645.692694	-0.360275215	0.000107	0.00132
TARBP1	1742.405164	-0.361472998	0.00383	0.01934
TBX3	943.6128135	-0.361811273	0.010865	0.04206
UNC119	1457.068786	-0.361852524	0.000586	0.0048
USP54	1262.939543	-0.362232708	0.010795	0.04187
GOLPH3L	1361.690965	-0.362586858	0.004376	0.02135
HPS3	1400.364361	-0.36334043	0.004034	0.02014
SLCO3A1	3158.091518	-0.363506278	0.011928	0.04516
ZNF512B	2083.391434	-0.363515348	0.000368	0.0034
ZFAT	767.627897	-0.363594003	0.006238	0.02788
LINC-PINT	133.4203978	-0.363851705	0.01196	0.04521
ZNF37BP	283.8568161	-0.363860884	0.007735	0.03275
FOXD2-AS1	178.1135563	-0.363901003	0.010417	0.04084
PHKA2	1197.241599	-0.364203588	0.000296	0.00288
ARHGAP19	735.0052118	-0.364597827	0.000585	0.0048
ZNF669	209.4390268	-0.365746458	0.000344	0.00323
TTC30B	364.6335117	-0.366029624	0.002066	0.01227

SLC45A3	368.8423907	-0.366176673	0.011615	0.04425
TBC1D8	1441.338773	-0.366283711	0.003963	0.01986
AKAP1	2998.023622	-0.366392761	0.000385	0.00353
CUL9	1581.708499	-0.366525415	0.001331	0.0089
ZNF302	517.6782126	-0.366583764	0.00155	0.00988
KEAP1	5810.72225	-0.366750317	5.89E-05	0.00083
ALKBH7	1480.876331	-0.366795134	0.01224	0.04595
LRRC37BP1	287.9789609	-0.366933589	5.19E-05	0.00075
PMS2P1	366.7389353	-0.367500375	0.000435	0.00386
B3GALT6	1583.096439	-0.367643434	0.005586	0.02578
ZNF678	268.2961301	-0.367831223	0.000409	0.00369
C17orf100	154.8525763	-0.368098853	0.009433	0.03786
RAVER1	1541.056758	-0.368675611	0.005401	0.02509
DDAH2	1621.278123	-0.369765938	0.009077	0.03677
LDOC1L	1909.466177	-0.369996963	0.00084	0.0063
RNASET2	1528.366815	-0.37057592	0.008433	0.03479
RP13-1032	114.5873767	-0.370920885	0.012348	0.04623
ATG4D	1432.292618	-0.371168946	0.000146	0.00168
IFT122	1255.552183	-0.372509987	0.000519	0.00441
ZNF599	172.3987282	-0.3731027	9.04E-05	0.00115
USP21	1222.632211	-0.374086909	1.23E-05	0.00024
PHF2	2676.203648	-0.374401437	0.000142	0.00164
ZNF827	908.8689284	-0.374748791	0.001457	0.00948
ZNF181	248.7175077	-0.374767548	0.00013	0.00154
DNAL4	908.0344408	-0.375041578	0.00047	0.0041
TRAPPC9	1795.880319	-0.375451867	4.49E-05	0.00067
PPM1M	731.3185445	-0.375748596	0.00072	0.00563
BCKDHB	442.0245357	-0.375807836	0.000829	0.00624
ZNF496	1670.369474	-0.376548057	8.74E-05	0.00111
TSNARE1	780.3618478	-0.376606004	0.002362	0.01342
TMEM136	145.4615895	-0.376622784	0.011843	0.04492
NRSN2	1597.656405	-0.376806711	0.003675	0.01876
ECE2	1362.021543	-0.376813243	0.008289	0.03438
CRTC3	1985.428671	-0.377073763	4.08E-05	0.00062
RP11-458F	231.0734186	-0.37740123	0.009194	0.03717
WDSUB1	348.331783	-0.377509671	0.000647	0.00519
RNASEL	786.2225547	-0.377696533	0.000375	0.00345
C14orf159	1593.793422	-0.377736535	0.000418	0.00376
DVL2	2243.546264	-0.377912135	6.88E-05	0.00094
SLC43A2	2206.195253	-0.378196417	0.004001	0.02001
MARCKSL1	5827.464109	-0.378306728	0.009702	0.03863
RAVER2	877.9469271	-0.379355717	0.002404	0.0136
IKZF4	339.958066	-0.379573409	0.000111	0.00136
GPX4	8010.243463	-0.37970742	0.002615	0.01454
PHF1	1823.3961	-0.380016392	2.45E-05	0.00042
POMGNT2	605.9040544	-0.380123315	0.003189	0.01682
RP11-420L	247.6369546	-0.380446419	0.010154	0.03997

CNPY3	3351.973146	-0.381915768	0.001076	0.00759
RP11-319(	72.76902826	-0.382309985	0.009819	0.03892
ALMS1	1189.332272	-0.382524938	8.65E-05	0.00111
SUN2	8318.081847	-0.382813523	0.000304	0.00294
PRR5	1194.924969	-0.382916434	0.006161	0.02759
NCK1-AS1	211.035473	-0.383407204	0.011183	0.04296
PTCD1	474.5034528	-0.383586408	0.001813	0.01107
JADE1	739.7658942	-0.383862951	0.000286	0.00282
MLLT3	727.270327	-0.383872752	0.007973	0.03349
CBX8	484.9881628	-0.384452461	0.000941	0.00684
TMX4	2265.320522	-0.384658909	0.007051	0.03049
NBPF3	259.9376488	-0.384851885	0.012565	0.04686
ZBTB42	716.6631223	-0.384953981	0.000379	0.00349
VANGL2	3418.022844	-0.38496295	0.011403	0.04362
ZBTB33	2034.125201	-0.385338396	1.95E-05	0.00035
USP46-AS1	82.78084267	-0.385402383	0.002294	0.01322
ZNF639	1943.979545	-0.385528231	0.000638	0.00514
ACADS	989.0367809	-0.385886535	0.004913	0.02332
MSTO2P	73.66186559	-0.385922073	0.006599	0.02901
ACTR1B	2879.084847	-0.385984686	2.53E-06	7.2E-05
AGFG2	958.8929632	-0.386888547	0.001556	0.0099
ZNF774	109.1774443	-0.387271316	0.004453	0.02164
TNRC6B	2173.295961	-0.387396726	0.000159	0.00179
RRAGB	470.8578943	-0.387675245	0.001242	0.00845
STS	572.5861351	-0.387845561	0.00964	0.03845
ANKRD39	561.945078	-0.388726513	0.00196	0.0118
GMDS-AS1	82.69309537	-0.388777049	0.000501	0.00429
WDPCP	204.6980913	-0.38886124	0.000506	0.00432
RSBN1L	1371.131663	-0.388864718	3.67E-05	0.00057
ZNF687	2167.896201	-0.389923005	8.61E-06	0.00019
C2CD5	1594.685551	-0.390039048	0.001442	0.00944
KCTD7	357.3782342	-0.390106146	0.000575	0.00475
SPHK2	599.812757	-0.390446029	8.34E-06	0.00018
CDPF1	345.1668012	-0.390506604	0.002095	0.01239
HLTF	2015.94886	-0.391073749	0.012568	0.04686
SLC25A33	443.974091	-0.391278442	0.003311	0.0173
FOXRED2	1756.963475	-0.391452413	0.005357	0.02491
FLJ37453	185.4710411	-0.391651693	0.002918	0.01573
PHC1	236.2032505	-0.391829664	0.004064	0.02025
THNSL1	370.445604	-0.39193768	0.010082	0.03976
SLC26A11	491.8867115	-0.392712975	0.003932	0.01974
HEXIM2	83.99013331	-0.39292304	0.000211	0.00223
ZNF821	137.9476692	-0.393964513	9.49E-05	0.0012
RP11-212F	320.6578264	-0.394089789	0.004578	0.02209
NLGN2	1784.573229	-0.394094393	0.008565	0.0352
SIPA1L3	3668.949682	-0.394247512	0.002087	0.01236
ZNF124	133.2145836	-0.394468969	0.003329	0.01737

RP5-882C2	76.03400729	-0.394712454	0.012279	0.04603
LBH	3593.629607	-0.394986556	0.009776	0.03882
GDF11	556.1539845	-0.395369112	0.006727	0.0294
THBS3	1191.131697	-0.395553682	0.003055	0.01631
DVL3	8491.636019	-0.397257313	0.000343	0.00323
ECE1	11948.48224	-0.397975383	0.001999	0.01194
RP11-110C	137.664215	-0.398114881	0.001605	0.0101
LAMB2	6987.232197	-0.39836099	0.002285	0.01319
RP11-352M	396.0579466	-0.39838284	0.000232	0.0024
RIT1	2209.281199	-0.398481252	0.001163	0.00803
AP001258	160.417138	-0.39870029	0.000156	0.00177
SLC9A8	1157.374814	-0.398873826	6.8E-05	0.00094
ZNF689	490.4189734	-0.398899529	2.75E-06	7.6E-05
SETMAR	363.7213405	-0.399534747	0.000525	0.00444
TP53TG1	948.5122789	-0.399606056	0.008255	0.03433
UNKL	646.5853513	-0.399635021	0.002481	0.01394
SUOX	800.5060943	-0.399747631	0.000302	0.00293
VAMP1	222.7318561	-0.399760726	0.010432	0.04089
CCT6B	69.84591288	-0.400105568	0.010635	0.04146
THAP7	1455.19056	-0.401038716	0.003111	0.01653
BBS5	191.5561215	-0.401079852	0.0026	0.01448
IQSEC2	941.5649327	-0.401698928	5.68E-05	0.00081
RNF168	2623.356237	-0.401784743	0.001786	0.01093
DZIP3	405.525595	-0.40201155	0.001186	0.00814
OGFRL1	2523.402778	-0.402013001	0.002354	0.01341
TSHZ1	1090.045678	-0.402401937	0.00104	0.0074
NT5DC3	626.6618532	-0.402638715	0.004369	0.02133
TMPO-AS1	173.8015779	-0.402953045	0.003169	0.01674
ASH2L	2839.266198	-0.40306473	0.002341	0.01336
MTMR1	2755.977777	-0.403561607	7.14E-05	0.00097
PRR19	113.7654419	-0.404976184	0.012804	0.04752
PRRT1	103.41808	-0.40499344	0.007132	0.03074
AIFM2	971.0212436	-0.406744598	0.002477	0.01393
ZSCAN2	185.806313	-0.406869382	0.00012	0.00145
NFE2L2	12729.18275	-0.406955162	0.002108	0.01244
C6orf226	211.8696237	-0.407218304	0.006718	0.02938
FGFRL1	942.6903091	-0.407405118	0.000625	0.00507
CRNDE	397.5334995	-0.407446969	0.005914	0.0268
PHYH	691.0032964	-0.407598911	0.006147	0.02755
MTUS1	3532.620565	-0.408166604	0.003109	0.01653
DDR1	24849.11195	-0.408469516	0.000763	0.00589
RMI2	1005.113107	-0.40898385	0.004825	0.02304
ZNF436-AS	78.43664009	-0.409323519	0.003305	0.01729
C7orf26	1473.240918	-0.40960769	3.86E-06	0.0001
MED12	2301.248358	-0.409844616	7.36E-05	0.00099
CCDC171	117.3298987	-0.4100584	0.001977	0.01186
ALDH3B1	550.5359823	-0.410107896	0.007725	0.03272

ZC2HC1C	79.19346179	-0.410111705	0.003779	0.01915
ZNF182	252.2700877	-0.410147863	9.15E-06	0.00019
FRAT2	866.9114287	-0.410255059	0.003018	0.01614
TMEM143	246.4842473	-0.410611182	0.000806	0.00612
DTX4	1299.779804	-0.410888953	0.010576	0.04128
NCOA1	2374.968305	-0.411146483	0.000281	0.00279
SNHG20	174.8578193	-0.411261451	0.006416	0.02845
ERMAP	695.0063702	-0.411273576	8.23E-05	0.00107
C7orf55	257.9548267	-0.412639958	0.006471	0.02864
CTPS2	692.4164175	-0.413313567	7.83E-05	0.00103
PARP16	535.2727603	-0.413336146	1.15E-05	0.00023
HS6ST1	4268.717534	-0.413630792	0.000275	0.00275
C1orf56	271.3903699	-0.413749822	0.00176	0.0108
ST3GAL3	657.9037562	-0.413949823	0.001967	0.01183
KLC4	928.0436649	-0.414437426	0.000228	0.00238
ZNF785	357.3026484	-0.414447726	0.000588	0.00481
BCL6	3573.035795	-0.414507562	0.000766	0.00591
CD83	994.2055296	-0.414920526	0.007111	0.03069
HIP1	2290.391481	-0.415888718	0.003393	0.01766
TTC30A	427.0797673	-0.416083984	0.005888	0.02673
ZNF497	69.88515271	-0.416536135	0.003136	0.01662
BCL2L11	1759.947636	-0.417569861	3.06E-05	0.00049
ZNF322	229.3655382	-0.417572892	0.003494	0.01802
ZNF248	384.7505058	-0.417833918	0.000961	0.00693
ZMYM3	1556.687988	-0.417951993	1.8E-07	9.2E-06
NPIPA1	109.0421283	-0.418759365	0.012783	0.04746
RFX3	278.5567377	-0.419039888	0.003465	0.01791
C7orf31	196.3853302	-0.419400404	0.003181	0.0168
PHOSPHO1	71.66108002	-0.419492982	0.005662	0.02604
SYTL4	361.0716063	-0.419754981	0.006236	0.02788
CHD6	2406.156233	-0.420441508	0.000172	0.00189
TNS2	1346.539411	-0.421283054	0.001554	0.0099
LAGE3	1185.243033	-0.422036533	0.010502	0.04109
PGAP3	1605.004114	-0.422070251	0.001698	0.0105
DOK3	377.2417792	-0.422298551	0.011784	0.04478
RPL32P3	199.143762	-0.422446899	0.007178	0.03088
ACTRT3	162.1897324	-0.422739097	0.008445	0.03483
ATP6V1E2	356.3852926	-0.422862466	0.000645	0.00517
PTPN18	1809.465763	-0.422874175	0.000105	0.0013
ARMCX3	1511.956761	-0.423161219	0.012526	0.04677
F8A1	518.5727446	-0.42352761	0.008227	0.03426
MBLAC1	93.37979741	-0.423670295	0.008723	0.0357
HAPLN3	1696.347492	-0.423994051	0.008868	0.03618
C9orf91	1146.929367	-0.42421237	7.35E-05	0.00099
MAPRE3	1063.825672	-0.42425365	0.010181	0.04006
CLSTN3	2169.879726	-0.425355193	0.000965	0.00696
APTR	289.5758739	-0.425372622	0.001593	0.01004

RAD51D	674.6711234	-0.425661748	1.11E-05	0.00022
PLEKHA4	1711.745802	-0.425689685	0.002608	0.01451
RNASEH1-/	128.8580554	-0.426577863	0.004011	0.02004
DUSP19	126.9940633	-0.426926711	0.000667	0.00532
LRP5	3898.834988	-0.427048421	0.001481	0.0096
USP27X	178.2308283	-0.427099503	6.88E-05	0.00094
TMEM44	1040.180627	-0.427241054	0.005295	0.02467
ACAP2	4436.389418	-0.427587697	0.000848	0.00634
RP5-821D1	217.8378289	-0.42823269	0.000522	0.00443
GLCCI1	376.49121	-0.428539251	0.006393	0.02838
RP11-567N	119.0839877	-0.428738124	0.006107	0.02744
MAGEE1	183.8757568	-0.428785188	0.011901	0.04507
FAM127C	1087.019544	-0.429648671	0.000985	0.00708
NEDD4L	2634.977102	-0.429766136	0.003573	0.01836
ZNF362	1468.45691	-0.43007428	6.2E-06	0.00015
EPM2A	228.4944982	-0.430235971	0.001435	0.00941
CREBRF	737.4502261	-0.431327204	6.37E-05	0.00089
BRD3	1906.766113	-0.431487886	8.54E-05	0.0011
C17orf96	716.6047473	-0.432224649	0.011968	0.04523
HEXDC	799.9520474	-0.432383627	0.003925	0.01972
VSIG10	838.6949614	-0.432748968	0.006117	0.02746
ATN1	9181.493397	-0.432781897	7.06E-05	0.00096
MPP1	782.2194791	-0.433606352	0.003816	0.0193
FRS3	207.0990506	-0.43424627	0.000868	0.00647
FAM161A	164.3676271	-0.434749622	0.002728	0.01501
ECHDC2	1089.692945	-0.435369986	0.009429	0.03786
STRBP	1598.132916	-0.435377616	0.002184	0.01277
AC002310	66.7144533	-0.435813311	0.003239	0.01701
CALHM2	764.4085592	-0.435947162	0.002173	0.01274
MLLT6	5230.46849	-0.435947889	7.97E-06	0.00018
FAM172A	762.476163	-0.437017005	3.04E-05	0.00049
ERO1LB	365.2215117	-0.437147751	0.002496	0.01401
U47924.6	95.88987849	-0.437461195	0.002452	0.01382
ATF5	2434.47818	-0.437507957	0.001054	0.00748
TPRN	1083.567101	-0.438281362	0.002904	0.01568
MLYCD	515.5187263	-0.438433976	6.25E-07	2.4E-05
TTYH2	464.2540319	-0.439514831	0.010703	0.04166
ZNF768	2069.979921	-0.439708716	5.63E-06	0.00014
SPATA6	121.1016813	-0.439860683	0.0078	0.03295
ZNF566	194.3749575	-0.44002744	0.001494	0.00964
DNASE2	3201.905775	-0.440531122	4.46E-07	1.8E-05
FAM86JP	303.1292875	-0.440912381	0.002504	0.01404
RP11-44M	167.0281423	-0.44135829	0.002244	0.013
GATAD1	1578.716647	-0.441394448	8.37E-06	0.00018
IRF2BP1	1266.579683	-0.442060204	1.16E-06	3.8E-05
DIS3L	1170.089381	-0.442559521	8.24E-05	0.00107
ZNF865	944.6446345	-0.442907569	7.29E-06	0.00017

RNF32	75.00139007	-0.443057838	0.000528	0.00446
ARHGAP31	1076.40753	-0.443095446	0.009867	0.03907
PYCR2	3636.372989	-0.444182484	3.51E-05	0.00055
LRCH4	555.0611989	-0.444319617	0.000129	0.00154
MEGF8	2734.343719	-0.444518522	0.000204	0.00217
ZNF764	369.1877133	-0.44470565	2.68E-09	3.4E-07
BOLA1	540.7533824	-0.444910744	0.001845	0.01123
GSN	28952.93979	-0.4450795	0.000421	0.00377
CCDC149	498.7945781	-0.44527637	0.002936	0.01579
PHC3	2234.202526	-0.445382688	0.00054	0.00455
ARL11	191.1609389	-0.445559298	0.004315	0.02114
NOTCH1	5509.596501	-0.446415272	0.001445	0.00944
GPR155	615.8279356	-0.447317111	0.008058	0.03371
OBSL1	2334.13451	-0.447444746	0.000893	0.00658
SFMBT2	408.0595553	-0.447567031	0.005788	0.02642
FAM228B	122.4828486	-0.448545181	0.001167	0.00805
GBAS	3551.93457	-0.448878948	0.008686	0.03558
SLC45A4	1228.505947	-0.449017748	0.00161	0.01011
RPARP-AS1	292.656302	-0.44925306	0.002182	0.01277
REPS2	298.1861793	-0.449581212	0.005063	0.02381
WFS1	1796.893357	-0.450103211	0.000108	0.00133
FUCA1	1327.656333	-0.450462548	0.000439	0.00389
CRTC1	646.5067597	-0.450827627	0.000106	0.00132
CENPM	754.5850341	-0.450943042	0.004903	0.0233
PLEKHG1	1104.291687	-0.451212199	0.004303	0.02112
PPAP2B	1942.171886	-0.451769759	0.008263	0.03434
TAPT1-AS1	63.97006097	-0.452253198	0.004525	0.0219
EVL	2506.6273	-0.452547375	0.000188	0.00203
ARSB	709.930497	-0.453242857	0.000349	0.00327
LINC01560	88.83155583	-0.453567675	0.000115	0.0014
LZTS2	3488.588618	-0.453645291	1.31E-05	0.00026
RP11-649A	74.03326105	-0.454178451	0.001578	0.01
PSD	171.3472362	-0.454744718	0.002369	0.01345
DGCR6L	2372.164516	-0.454904824	0.002252	0.01303
PRR3	537.6718764	-0.455596375	0.000163	0.00183
NINJ1	2657.900044	-0.455898479	0.001159	0.00801
CYP51A1	429.8592058	-0.456528503	0.011812	0.04484
CCT6P1	143.1251395	-0.456545081	0.001444	0.00944
C19orf44	246.4752286	-0.457205723	7.14E-06	0.00016
H2AFY2	1660.689962	-0.457280733	0.000905	0.00664
SLC46A1	225.9000379	-0.457427307	0.005741	0.02626
CNNM3	1026.447247	-0.457697778	2.4E-05	0.00041
SPIDR	2635.143807	-0.458034872	1.73E-05	0.00032
CARF	167.4910373	-0.458440403	2.51E-05	0.00043
INCA1	86.07838176	-0.458710928	0.000162	0.00182
C10orf35	396.8876791	-0.458818703	0.006658	0.02917
OSBPL7	314.7566495	-0.459041278	0.001521	0.00978

TRRAP	4331.487867	-0.459114833	7.55E-05	0.00101
HSD17B11	926.7231212	-0.459252136	0.002877	0.0156
PRX	255.0930598	-0.45952264	0.000948	0.00688
RP11-1017	78.62423207	-0.459822931	0.00744	0.03184
HCK	1256.624463	-0.459882796	0.008657	0.03549
LINC00265	145.4037733	-0.460540063	0.001473	0.00956
BMF	757.4125251	-0.460771135	0.001495	0.00964
PTPN6	2516.380966	-0.460862148	1.64E-05	0.00031
TLR5	417.2927029	-0.461312502	0.013599	0.04976
SVEP1	735.1337548	-0.461407387	0.013652	0.04989
CAMK1D	1765.808612	-0.461575922	0.00263	0.0146
TTC28-AS1	415.6915065	-0.461879975	0.000489	0.00422
FAM102A	6600.961863	-0.462022009	0.001066	0.00754
ING4	1096.120857	-0.462140647	0.000135	0.00158
PRRT3	129.8057023	-0.46227491	0.001606	0.0101
C11orf71	234.7700735	-0.462764118	0.001922	0.0116
ABHD4	2746.878428	-0.462790103	0.000893	0.00658
DFNB31	554.8043805	-0.462877803	0.00959	0.0383
LINC00324	119.9668572	-0.462882852	0.002391	0.01355
TRPV2	706.259939	-0.463093963	0.009011	0.03657
TMEM147	232.0146033	-0.463123084	0.005787	0.02642
GRASP	363.5984273	-0.46323354	0.006497	0.02871
ZNF32	1011.633135	-0.463345284	0.000724	0.00565
RP11-705C	228.2778746	-0.464013048	0.000819	0.00619
CASK	3918.156926	-0.464172058	0.000132	0.00156
GPRC5C	1313.175096	-0.464772486	0.008815	0.03601
NFIX	3910.028003	-0.464795553	0.000823	0.00621
INSIG1	2494.640263	-0.465380839	0.003515	0.0181
SLC25A45	244.7750502	-0.465429486	0.001648	0.01027
C6orf48	2723.129404	-0.465606912	0.000404	0.00365
ETV6	2683.274054	-0.466641095	5.38E-06	0.00013
TIGD1	232.0418781	-0.466717872	0.001208	0.00827
AKNA	1529.09041	-0.466921417	0.002059	0.01224
NEK8	260.3268675	-0.467044766	3.37E-05	0.00053
SEPT6	1287.510179	-0.467557509	0.000724	0.00565
ZNF446	363.3376966	-0.467948599	1.18E-06	3.9E-05
ARHGAP30	1266.444148	-0.468175676	0.005656	0.02602
KIAA1107	80.90074305	-0.468368187	0.00094	0.00684
KIAA0195	3057.802768	-0.469536824	9.32E-09	9.2E-07
AKAP9	3098.382255	-0.470130084	0.00014	0.00163
LINC01410	104.8775827	-0.47028551	0.002818	0.01539
KLHL22	1093.356173	-0.470493132	0.000802	0.0061
HYKK	117.0334707	-0.470524077	0.000622	0.00505
ZNF512	741.7702735	-0.470706501	3.26E-05	0.00052
SQSTM1	12520.23065	-0.470991565	0.000176	0.00193
GLUL	25940.25401	-0.471010665	0.004599	0.02217
FAM13A	719.7456281	-0.471531492	0.000353	0.0033

MFSD7	205.1776143	-0.472368107	0.01181	0.04484
SIPA1L2	3464.54075	-0.472958864	0.008451	0.03484
CTSF	1405.346267	-0.473536878	0.006505	0.02872
FBXO27	1183.288312	-0.474444728	0.006554	0.02887
LINC00674	83.60422279	-0.475087073	0.000399	0.00362
RBMS3	475.8991937	-0.475088019	0.00638	0.02836
SVIL	7747.552148	-0.475378205	0.00077	0.00593
FRAT1	193.1548024	-0.476003703	0.000383	0.00352
ASNS	2123.309695	-0.476558603	0.006429	0.02849
RP11-396K	231.2572273	-0.47656361	3.29E-05	0.00052
BHLHB9	221.2759316	-0.477056021	0.000844	0.00632
ZNF425	112.9129991	-0.47747732	0.001022	0.00731
CCDC146	258.6810205	-0.477519605	0.012326	0.04619
ZNF618	983.0729218	-0.47791671	0.005257	0.02452
NBPF11	144.5736054	-0.478151153	0.000244	0.0025
DLGAP1-AS	224.5969014	-0.478237563	0.00492	0.02333
MMP25-AS	204.4021239	-0.478458887	0.002976	0.01596
URB1-AS1	208.4482239	-0.478624639	0.000869	0.00648
NEURL1B	2266.706665	-0.478710306	0.000282	0.0028
ASB16-AS1	292.313205	-0.478729356	1.12E-06	3.7E-05
PRICKLE1	492.4886616	-0.479156241	0.013632	0.04985
SLC27A1	963.8819625	-0.480247343	9.61E-05	0.00121
WASH2P	101.4960096	-0.480640029	0.0067	0.02933
VASH1	924.6246591	-0.480794328	0.002567	0.01434
MAP3K14-	69.09997139	-0.481042169	0.000344	0.00323
ZMYND15	154.0645728	-0.481300118	0.004663	0.02242
TNFRSF1B	2286.659157	-0.481482676	0.008103	0.03384
F8	295.0049023	-0.482341417	0.000701	0.00551
DPP7	4490.764551	-0.482545411	0.00067	0.00533
TRAM2-AS	157.133041	-0.482606385	0.000122	0.00147
IDH1	4641.61159	-0.482860659	0.000105	0.00131
C12orf57	2872.81433	-0.482866801	0.006243	0.0279
LCA5	175.8353783	-0.483298801	0.001602	0.01008
ACBD4	235.6306173	-0.483417706	0.000792	0.00605
ARSG	260.0232435	-0.483661196	0.000169	0.00187
MPDZ	493.0312947	-0.484391366	0.011317	0.04335
SPAST	1633.358102	-0.4844095	2.33E-05	0.0004
LRP6	1344.875215	-0.484899499	0.000623	0.00505
CYB5A	1293.954381	-0.485306687	0.005519	0.02555
AC004967	132.5636023	-0.485327291	0.00034	0.00322
LRP1	23201.71508	-0.485478102	0.001274	0.00862
KCNJ2	414.0910556	-0.48580905	0.009417	0.03782
TCTEX1D2	286.933805	-0.485836672	0.007714	0.03271
SUZ12P1	231.7493032	-0.486004058	0.000553	0.00463
RP11-289I	149.4155427	-0.486076998	0.009241	0.03733
DTX2P1	139.0243872	-0.486104273	0.002514	0.01409
ZIK1	86.80605982	-0.486144533	0.012563	0.04686

ENDOV	560.9053665	-0.486601186	0.000369	0.00341
ACVR2B	387.4998514	-0.486771222	1.36E-05	0.00027
IGFLR1	79.04839324	-0.486916477	0.006531	0.0288
UTRN	3086.02151	-0.487303434	0.003411	0.01771
ARMCX6	444.9582267	-0.487833386	0.010102	0.03983
ILF3-AS1	869.5025605	-0.48903623	0.000441	0.00391
FTLP3	86.00098302	-0.489190368	0.00839	0.03471
SIGIRR	822.5028001	-0.489235203	0.000891	0.00657
SPRN	185.4800227	-0.489256243	0.00404	0.02016
SLC37A1	659.4371116	-0.489562193	0.00082	0.00619
PLCB1	863.7791803	-0.489719689	0.004808	0.02299
MYO1F	899.1021514	-0.489834499	0.007539	0.03219
TCF4	3737.84454	-0.490423732	0.001063	0.00753
CLEC2D	299.7672429	-0.490497377	0.005812	0.02649
SPRY2	947.3244385	-0.490927102	0.010143	0.03997
CTB-58E17	120.8367131	-0.491271799	0.000927	0.00677
RNF166	683.0231249	-0.491297039	1.07E-05	0.00022
ZNF428	1023.618711	-0.491533257	0.00078	0.00599
AATBC	267.0185893	-0.4916139	0.006995	0.03028
TUBA1A	5308.634139	-0.491855801	0.010314	0.04049
RP11-196(	270.7042732	-0.49230007	0.001647	0.01027
APBB1	490.8698577	-0.492700445	0.009118	0.03689
CDKN1C	477.4128673	-0.492830229	0.007502	0.03207
ZBED1	4349.048737	-0.49311006	0.000172	0.00189
RP11-46C2	114.7054106	-0.493538916	0.000366	0.00339
APBA1	270.0826605	-0.493606686	0.006507	0.02872
LINC00888	129.4740488	-0.493867066	0.012433	0.04648
TMEM231	200.1958133	-0.494012786	0.008014	0.0336
LIPE	275.1587372	-0.494526144	0.004372	0.02134
H6PD	5036.911811	-0.494846914	3.74E-05	0.00058
CTB-25B13	255.8825537	-0.495011065	0.000291	0.00285
PPP1R3E	301.7972441	-0.495314289	0.000155	0.00176
MORN4	162.9324743	-0.49540426	0.008309	0.03445
EFCAB2	152.4914265	-0.495971614	0.000628	0.00508
NIFK-AS1	149.7227959	-0.49612449	0.000108	0.00133
JAK3	881.0166392	-0.496481632	0.008841	0.03611
SLC6A8	11135.29725	-0.49714012	0.002473	0.01393
KSR1	1287.092383	-0.497163096	0.000595	0.00485
LINC01089	186.7656167	-0.497610574	0.012808	0.04752
RHBDD3	1463.261586	-0.497782799	6.79E-05	0.00094
ZNF354C	150.7462208	-0.49862244	0.011421	0.04368
FAM198B	1960.266965	-0.498849862	0.012435	0.04648
RP11-1212	65.91756588	-0.499270923	0.010968	0.04233
CTB-31O2(	74.13352023	-0.499596758	0.005482	0.02541
RASSF4	1013.152454	-0.499789887	0.005703	0.02613
NUDT16	1409.149979	-0.499808414	1.73E-07	8.9E-06
SCAMP5	428.4217065	-0.499907089	0.009793	0.03886

TMEM170	121.1766521	-0.50000864	0.008484	0.03496
PI4KA	3969.153244	-0.500035212	2.84E-05	0.00047
ZNF74	787.3242637	-0.500150082	5.58E-05	0.00079
NBPF1	393.1362975	-0.500499711	5.27E-05	0.00076
SOX12	1628.607996	-0.500981027	0.000632	0.0051
ARHGEF6	628.1717529	-0.501105195	0.008551	0.03517
RP11-705C	108.4806621	-0.501492149	0.001773	0.01087
ZNF554	174.5185138	-0.501577475	5.86E-05	0.00083
AKT3	1081.457381	-0.501848899	0.004047	0.02019
TTLL3	252.49691	-0.502020051	0.007911	0.03331
ADD3	3773.455568	-0.502546112	0.000874	0.0065
BBC3	405.4294095	-0.503128337	0.000586	0.0048
ZFAND4	240.1284596	-0.503435107	2.47E-05	0.00042
FADS1	2670.807985	-0.503540849	0.007718	0.03271
ZNF275	1029.841273	-0.504951938	0.00024	0.00247
PHACTR1	206.6918256	-0.505373237	0.002725	0.015
AC004893	115.7095605	-0.505491197	5.9E-05	0.00083
RP11-258C	110.0020495	-0.50550016	0.005146	0.0241
PLCE1	420.2727472	-0.505517453	0.003426	0.01777
FZD8	427.534834	-0.505534695	0.006789	0.02959
RP11-81A1	70.44421968	-0.506019985	0.00058	0.00478
ZNF3	1191.323357	-0.506152041	1.5E-08	1.4E-06
PLXNA3	2146.216561	-0.506622052	0.000932	0.00679
ZXDA	140.2119106	-0.507391764	7.24E-06	0.00016
GALNT11	2024.650505	-0.508048146	0.000592	0.00484
IL17RB	100.8183101	-0.509705664	0.00839	0.03471
CUEDC1	1702.448141	-0.509997783	2.3E-06	6.7E-05
DGCR6	242.1396726	-0.509998386	0.004156	0.02051
RASAL3	451.7102665	-0.510154643	0.010506	0.04109
CTA-293F1	79.55383789	-0.510186136	0.004885	0.02323
RASSF2	842.62435	-0.510398766	0.009804	0.03889
PCDHB9	158.1410349	-0.511050384	0.005839	0.02657
ZSCAN16-A	215.7199253	-0.511502929	0.008265	0.03434
FAM171A2	185.3731684	-0.511547304	0.003298	0.01726
ATP9A	3747.115205	-0.511648223	0.001534	0.00981
GRID1	93.01266499	-0.512136573	0.012355	0.04623
PLXNB3	818.1290099	-0.512983794	0.000881	0.00654
MRAS	785.1984742	-0.51342179	0.008354	0.03458
CEP68	1374.326101	-0.513588502	1.44E-07	7.8E-06
RPP25	1281.984302	-0.514012393	0.003126	0.01658
SPDYE3	269.2955327	-0.514427552	3.41E-08	2.6E-06
CTSO	1113.599481	-0.515170324	0.000797	0.00607
DNAH1	183.2629418	-0.515390362	0.001115	0.00778
AIF1	859.8398246	-0.515493309	0.012336	0.04621
CYP4V2	470.7015275	-0.516393885	0.002128	0.01252
MAFG-AS1	226.7532547	-0.516567485	0.006615	0.02904
NPR1	302.6497926	-0.517060992	0.013661	0.0499

ACVR2A	634.1556931	-0.517350127	2.17E-06	6.4E-05
C19orf57	188.5145198	-0.517654628	0.002016	0.01203
FAM222A	209.435217	-0.517857121	0.006609	0.02903
EVI2A	255.9566768	-0.518419855	0.009751	0.03874
RUNX3	1998.700032	-0.518760018	0.002392	0.01355
FRMD3	82.63115422	-0.519465889	0.009955	0.03935
CROCC	1827.186144	-0.519728588	0.000694	0.00547
GPER1	183.4646382	-0.520144124	0.007933	0.03339
RP11-504F	116.8190317	-0.52050556	0.003222	0.01696
TOR3A	2715.818583	-0.520621064	7.55E-08	4.6E-06
LXN	345.0350912	-0.520906144	0.004919	0.02333
NCBP2-AS2	1174.594957	-0.520979683	0.000266	0.00269
FOXP3	443.940447	-0.520996059	0.012026	0.04535
RP13-582O	138.4702314	-0.523357476	0.004229	0.02082
SPRY3	85.83187343	-0.523544749	0.001583	0.01001
DAB2	2077.416645	-0.523638497	0.003825	0.01933
TMEM25	606.1343337	-0.523687756	0.006628	0.02908
LIMS2	552.548924	-0.523738493	0.001783	0.01092
SLC9A7	1315.77939	-0.52410084	0.001445	0.00944
DENND5B	312.1103321	-0.524363756	0.00483	0.02306
TRIB2	2446.410716	-0.524955741	0.001217	0.00831
CD300A	430.181108	-0.525233331	0.004653	0.02239
CSF1	2077.459782	-0.525650388	0.001584	0.01001
PIGZ	339.8963257	-0.525803894	0.00363	0.01857
GABARAPL	2670.967888	-0.525861565	0.000226	0.00236
DCAKD	1438.227403	-0.526142715	4.45E-06	0.00011
SSBP2	660.2978863	-0.526219227	0.000145	0.00167
RFTN2	166.6121768	-0.526514486	0.002624	0.01457
ZNF736	248.9366984	-0.52706411	0.005846	0.02658
GAB3	146.2373314	-0.527458367	0.002088	0.01236
CTA-445C9	112.6755163	-0.527645781	0.000388	0.00355
SPATA18	247.9169219	-0.528128364	0.004894	0.02326
ADAMTS17	267.9627334	-0.528701693	0.007683	0.03264
RP5-1074L	73.46507054	-0.528728799	0.007501	0.03207
FAM117A	563.0233287	-0.528741527	7.35E-06	0.00017
LINC01003	138.3555785	-0.528792616	0.000341	0.00322
KIAA1467	623.0490171	-0.529289088	0.000594	0.00485
HSPA12B	269.9329726	-0.529480702	0.003812	0.01928
PTPRJ	1010.429134	-0.529833231	0.000873	0.0065
CD99L2	1795.304594	-0.530292447	9.06E-06	0.00019
LMF1	523.9684537	-0.530443457	0.000535	0.00451
ZFP3	250.217628	-0.530584977	0.006489	0.02869
SERPINF1	5942.712312	-0.530647726	0.009348	0.03762
RP11-147L	87.3545389	-0.53112061	8.01E-05	0.00105
XXYLT1	2219.802136	-0.531436407	7.77E-06	0.00017
GPNMB	42555.21676	-0.531459333	0.011747	0.04466
SLA	728.8221523	-0.532544543	0.007969	0.03349

TBL1X	2198.184995	-0.532598113	0.003343	0.01743
CYP2U1	216.6087646	-0.533076163	0.00109	0.00765
NRIP2	83.31524753	-0.533151696	0.000547	0.00459
NINL	671.8925794	-0.53455113	0.000868	0.00647
UGDH	4552.057024	-0.534608866	0.001982	0.01187
LTBP4	6222.979676	-0.534647505	0.000144	0.00167
SDC2	2292.518345	-0.535134058	0.013287	0.04885
LINC01569	93.70339419	-0.53587125	0.002192	0.0128
PDE4A	925.8058412	-0.535897512	3.16E-05	0.00051
SLC25A4	1182.566155	-0.536058606	0.004907	0.02331
AZIN2	259.1400091	-0.536067713	0.007294	0.03132
TSPAN12	160.1442692	-0.536152755	0.008163	0.03403
TMEM158	729.7755227	-0.5363927	0.008991	0.03653
RP3-439F8	84.42435792	-0.536525428	0.006127	0.02749
SIGLEC9	104.3052097	-0.53655977	0.004087	0.0203
RP11-390F	87.1368825	-0.536615237	0.005726	0.02622
RHBDD2	5301.396348	-0.536760574	1.73E-07	8.9E-06
FAM47E-ST	100.2838363	-0.53707462	0.001102	0.00771
FLVCR1	629.755642	-0.538074716	0.000364	0.00338
LRRC37A1(	202.8146061	-0.538436028	0.000291	0.00285
RP11-147L	817.3695786	-0.538643912	6.33E-08	4E-06
DPEP2	88.27021277	-0.539187556	0.004936	0.02338
SPSB2	588.5638849	-0.539640437	0.000256	0.0026
RP11-277F	354.5083258	-0.539836807	0.011947	0.04521
TRIM46	97.59060317	-0.540036567	0.002078	0.01232
TRIM59	649.5324178	-0.540197678	4.38E-05	0.00065
FRG1B	368.7016857	-0.54046017	0.003088	0.01647
WIPF1	2410.739371	-0.540596236	0.001309	0.0088
EPB41L4A	259.2446806	-0.541026891	0.006991	0.03027
ZNF671	86.13878435	-0.541070789	0.002332	0.01333
FAM49A	611.1244387	-0.541573664	0.001707	0.01053
KAT6B	1305.767989	-0.542321805	4.37E-06	0.00011
DOK2	470.5643492	-0.542616715	0.007939	0.0334
RP11-44F2	139.3492787	-0.542642515	0.013316	0.04891
RP11-359E	70.17549002	-0.542758172	7.29E-05	0.00098
RP11-1246	109.1180724	-0.543221784	0.004184	0.02063
LRRC23	363.4549024	-0.543911786	0.000261	0.00265
TNFRSF14	1606.468201	-0.544050988	0.000711	0.00557
SLC41A2	361.2678309	-0.544280949	0.002024	0.01207
GIMAP1	208.8419979	-0.544416019	0.006234	0.02788
ZNF253	130.9068941	-0.544467989	0.005562	0.02571
KCTD13	639.2765609	-0.544491845	1.13E-06	3.7E-05
CDKN2C	756.5491336	-0.545550159	0.000474	0.00412
ELMO1	973.0955375	-0.546252852	0.003427	0.01777
LDLRAD4	789.9272412	-0.54645107	0.007087	0.03063
DZIP1L	354.8648152	-0.546485117	0.006011	0.02713
PHF8	1811.085126	-0.547184993	5.87E-06	0.00014

PARD3B	294.9821665	-0.547512607	0.004873	0.02318
CRMP1	1003.952622	-0.547565213	0.004855	0.02313
PCDHB16	230.4663961	-0.547686256	0.01353	0.04962
FAM120C	577.247991	-0.547704145	4.72E-06	0.00012
LPIN1	1199.728434	-0.54781517	7.52E-05	0.001
LMOD1	310.107799	-0.54848418	0.011475	0.04383
TXNDC16	371.2540724	-0.548514356	7.99E-05	0.00105
TMEM44-A	203.7415366	-0.548798167	0.001069	0.00756
PEX1	921.7121413	-0.548908488	1.1E-05	0.00022
FAM78A	391.6307102	-0.549184486	0.001845	0.01123
AMER1	681.2108558	-0.549632283	4.46E-05	0.00067
PBXIP1	5306.296474	-0.549720213	6.27E-06	0.00015
ARHGAP44	163.9480427	-0.550454251	0.004491	0.02178
MAGEF1	2250.870719	-0.55104006	3.78E-05	0.00058
MAP1S	1660.197105	-0.551756081	8.12E-08	4.9E-06
TRPM2	874.096302	-0.551963615	0.008076	0.03376
TYROBP	1734.551304	-0.552221843	0.005771	0.02636
FOXS1	116.1329497	-0.552364971	0.005736	0.02624
ST6GALNA	92.94278386	-0.55262286	0.011466	0.04381
DDIT3	1017.861174	-0.553074184	0.001406	0.00928
IFT27	831.8406444	-0.553127092	0.000247	0.00253
ALDH6A1	703.5793785	-0.554667688	1.8E-05	0.00033
CTD-2368F	70.7104784	-0.554724012	0.011484	0.04384
RELB	2267.95589	-0.554828474	1.46E-05	0.00028
TMEM26	69.05671988	-0.555022544	0.009713	0.03867
RP3-510D1	65.5472432	-0.555104416	0.002318	0.0133
TNRC6C-AS	294.6093111	-0.55660921	0.000157	0.00178
SGMS1-AS	115.7187876	-0.556704232	1.54E-05	0.00029
OSER1-AS1	162.4826242	-0.556711334	0.00055	0.00461
ZC3H12D	103.2474381	-0.557070251	0.006306	0.02811
FZD3	441.2330151	-0.557159083	0.001217	0.00831
AKAP7	185.0845669	-0.557346287	0.000248	0.00253
B4GALT4	2307.999418	-0.557677467	0.001099	0.0077
AKAP5	98.08610091	-0.557722033	0.001572	0.00997
LINC00847	483.6751458	-0.558051003	3.55E-08	2.7E-06
CD6	450.5872239	-0.55917431	0.009498	0.03802
HLA-DPB1	9181.336241	-0.559480124	0.013219	0.04872
CLYBL	209.4963959	-0.559573826	0.000686	0.00542
IVD	1743.489264	-0.560392264	0.000563	0.00469
RP11-617F	181.1826707	-0.560867196	0.002885	0.01563
GPRIN3	439.8171389	-0.561252527	0.004345	0.02124
HID1	491.1551267	-0.561491374	0.012535	0.04678
ZNF48	516.8749755	-0.561563244	2.93E-06	8.1E-05
C2	994.0899675	-0.562140419	0.002825	0.01541
AASS	326.7914282	-0.562780958	0.002236	0.01299
CD247	313.6997099	-0.562877856	0.01326	0.04883
TNFSF15	193.8002098	-0.562891591	0.008895	0.03627

ABCC1	11448.32003	-0.562985166	0.001844	0.01123
ABI3	553.271765	-0.563562063	0.001512	0.00972
CAMK4	219.0485125	-0.563967139	0.004993	0.02356
KCNK13	56.24100351	-0.564053727	0.002268	0.01311
MAGED1	8653.258826	-0.564652088	0.000461	0.00405
PARD6A	93.06275781	-0.565177056	0.003158	0.0167
FAM50B	551.5433271	-0.565184905	0.000469	0.0041
CPLX1	103.2456297	-0.565455516	0.012642	0.04706
MGAT4A	572.1907737	-0.566194625	0.000922	0.00674
ANKRD13E	700.5670423	-0.566240437	0.000629	0.00509
IGSF9	2799.004718	-0.566427717	0.000681	0.0054
PIK3IP1	2161.539347	-0.566601893	5.26E-05	0.00075
KCND1	122.5858382	-0.567076891	0.001588	0.01003
CMTM8	191.2552034	-0.567472602	0.000514	0.00438
ZNF579	456.1773022	-0.567799663	0.00032	0.00307
TNFSF12	541.7792301	-0.567829913	0.000136	0.00159
PRSS36	127.7603487	-0.568781769	0.001793	0.01097
PCDHB15	105.5711717	-0.56973432	0.004727	0.02267
SATB1	719.4704667	-0.569791423	0.00017	0.00188
GGTA1P	199.0931251	-0.570177247	0.007564	0.03226
MRC2	8705.829544	-0.570445791	0.001564	0.00992
ATP2A3	1798.354062	-0.570807141	0.004428	0.02155
STEAP2	739.9116979	-0.570938667	0.0038	0.01923
ZNF789	282.6886831	-0.571850492	1.64E-05	0.00031
EXOC6	567.5124954	-0.572060689	1.76E-05	0.00032
TBXAS1	465.3367222	-0.572084184	0.000609	0.00496
MILR1	86.8671993	-0.572157524	0.003188	0.01682
CCDC102A	463.2127194	-0.572186162	2.98E-05	0.00048
MARC2	203.5449854	-0.572713763	0.002718	0.01498
TMEM140	1036.885888	-0.573013132	8.27E-06	0.00018
APBA2	559.8092138	-0.573235789	0.007976	0.0335
DNMT3A	1588.25091	-0.573791123	9.64E-08	5.6E-06
EGFL6	748.7732308	-0.573861732	0.011992	0.04526
FRY	417.0656794	-0.574068991	0.005795	0.02644
ENPP1	268.9156739	-0.574346846	0.011194	0.04299
SASH3	730.8929152	-0.574836324	0.007632	0.03246
TGFB3	2377.709653	-0.575382518	0.007138	0.03076
FBXL7	386.5858333	-0.575619408	0.004782	0.02289
KCNIP3	225.0834611	-0.576327344	0.010783	0.04186
PSPH	1422.296079	-0.576340253	0.003447	0.01786
SOX13	1799.993206	-0.576501774	0.001002	0.00719
GALM	482.3857804	-0.576599927	0.000961	0.00693
RP4-639F2	69.44849075	-0.577102637	0.002429	0.01372
KIF26B	1739.002523	-0.577625167	0.00408	0.02029
HIC1	415.7650503	-0.577857168	0.00063	0.00509
ZNF280C	316.6177112	-0.577878268	8.14E-05	0.00106
CTD-2267D	486.6156757	-0.577955295	7.8E-05	0.00103

NNT-AS1	450.8196345	-0.578643856	0.000306	0.00296
GOLM1	3611.001348	-0.578977833	0.000372	0.00343
PLXNC1	995.9745854	-0.579035153	0.004324	0.02116
KIAA1147	1729.318914	-0.579287649	0.000168	0.00187
IRS2	1258.748727	-0.579418509	0.000918	0.00672
TCEA2	1032.367291	-0.579989955	0.000121	0.00146
ARHGEF19	2246.929556	-0.580187611	3.17E-05	0.00051
DISP1	180.7556903	-0.580572246	8.53E-06	0.00018
BCAS3	1475.482268	-0.580639757	2.78E-09	3.4E-07
UBBP4	260.4028838	-0.580921642	0.000207	0.0022
PIK3R1	2390.512837	-0.582527369	0.000309	0.00298
NRROS	202.3560972	-0.582577197	0.001687	0.01044
CD300C	79.78853388	-0.582590452	0.004249	0.0209
PPAPDC1B	1299.219596	-0.584024423	0.000875	0.00651
SPI1	1116.556332	-0.584053997	0.002225	0.01295
ZFPM2	186.3229737	-0.584096694	0.004858	0.02313
SPATA13	550.3047422	-0.584311219	0.000577	0.00476
CREB3L4	487.9754607	-0.584921624	3.99E-06	0.0001
KANSL1-AS	66.08655088	-0.584938212	0.009731	0.03869
LZTS3	596.7662601	-0.585301771	0.001124	0.00782
ZC3H6	368.0581756	-0.585411918	6.19E-07	2.4E-05
PPAP2A	982.6317768	-0.58549407	4.72E-06	0.00012
CLCN2	492.9701662	-0.585596146	0.000132	0.00156
KCNJ14	111.5493481	-0.586640256	7.7E-05	0.00102
KIAA1324L	553.1587705	-0.586778563	0.000679	0.00538
ATP1B1	12767.84369	-0.586984909	0.000138	0.00161
ARHGAP15	196.8966787	-0.587087272	0.003148	0.01667
TEAD2	1958.768757	-0.587209305	1.1E-06	3.7E-05
KBTBD11	133.3635099	-0.587264365	0.003278	0.01717
PTGR1	4176.097854	-0.587320544	0.011238	0.04312
CRAT	1582.199275	-0.5876455	0.008225	0.03426
OR2A1-AS	142.3341297	-0.58852791	0.004518	0.02188
NR1H3	633.7880024	-0.588776225	0.000133	0.00157
ZNF546	75.06559123	-0.588805975	6.82E-05	0.00094
CD3E	829.6275649	-0.589021795	0.011174	0.04295
TMEM97	1541.890995	-0.589297964	0.000357	0.00333
CLUAP1	895.2837088	-0.589587164	4.27E-06	0.00011
ZNF775	452.6928671	-0.589675001	2.28E-05	0.00039
SSC4D	122.3640363	-0.59014323	0.003903	0.01963
RDH10	3420.335414	-0.590364925	0.005025	0.02366
SERPINF2	353.5935856	-0.590860974	0.000229	0.00239
SLC25A42	472.4484205	-0.590967418	3.19E-05	0.00051
FKSG48	71.66286873	-0.593058889	0.012867	0.04769
LIMD2	1023.366063	-0.593954154	0.000857	0.0064
KLHL29	530.7508936	-0.594013537	0.000779	0.00599
AC005537	71.55827459	-0.594084467	0.004946	0.0234
GPR162	77.79991212	-0.594272854	0.001141	0.00792

CD300LF	134.1499293	-0.594476838	0.005142	0.02409
SEMA3G	283.2017274	-0.594757118	0.002862	0.01554
ACTR3B	259.4813177	-0.594760328	1.2E-06	3.9E-05
LRRK2	296.0772402	-0.594973427	0.009894	0.03916
AC009506	85.97870886	-0.595078036	5.58E-06	0.00014
chr22-38_	966.308544	-0.59517837	0.000852	0.00637
KLHL13	692.679394	-0.595536583	0.001933	0.01166
WAS	485.4783828	-0.596689517	0.002572	0.01435
FTL	97789.73453	-0.59688696	3.3E-05	0.00052
CDK20	166.4337594	-0.596923386	0.000725	0.00565
JPH1	632.1737099	-0.596958499	0.008673	0.03555
DPY19L1P	74.17749454	-0.598234225	6.68E-06	0.00015
AP001062	75.43226599	-0.598467207	0.001116	0.00778
RNASE6	389.715567	-0.59906305	0.002196	0.01281
TNFSF4	236.6440511	-0.599263437	0.009675	0.03855
TIGIT	375.9613466	-0.599682527	0.010759	0.0418
CD53	1414.333495	-0.599726221	0.004812	0.023
ENPP2	744.6649879	-0.59994524	0.004084	0.02029
SEMA4D	1971.673828	-0.600077466	0.000103	0.00128
RP11-1275	112.4379238	-0.600086321	0.001217	0.00831
IL2RB	1063.045863	-0.600912605	0.003571	0.01835
FAAH2	276.9005962	-0.601154135	0.001431	0.0094
MEGF6	1748.862653	-0.601603045	0.000796	0.00607
MYH7B	72.73561869	-0.602180372	0.004911	0.02332
SPIN2B	75.50415901	-0.60237618	2.39E-06	6.8E-05
ARHGAP26	887.4371158	-0.603138699	6.57E-05	0.00091
NOTCH3	20736.43705	-0.603298126	0.000184	0.002
FZD10	1149.779475	-0.603381124	0.009003	0.03655
MARCH1	404.9580298	-0.603576374	0.000111	0.00136
BCL11A	1210.64639	-0.604196233	0.000184	0.002
CEBPA-AS1	101.9225606	-0.604344149	0.000657	0.00525
SLC7A7	658.5188931	-0.604456769	0.002212	0.01288
LRCH2	75.10952248	-0.604508659	0.013543	0.04964
EIF4EBP3	109.6097292	-0.604512854	0.002011	0.012
RNF130	2152.081614	-0.604621396	5.5E-07	2.1E-05
RP11-448A	78.88085408	-0.605550092	8.08E-05	0.00106
IL27RA	1358.333132	-0.605817323	0.000693	0.00546
CTA-29F11	177.6404311	-0.605885372	0.003703	0.01888
RASGRF2	309.5887028	-0.606177763	0.002216	0.0129
MDM1	356.5578773	-0.606508487	3.95E-06	0.0001
FAM174B	336.5770396	-0.606518581	5.42E-05	0.00077
PSMG3-AS	151.7752767	-0.607172129	0.00016	0.0018
PRAF2	932.2943186	-0.607240182	1.93E-05	0.00035
USP11	3431.689278	-0.60855876	4.17E-09	4.7E-07
CD96	316.6631064	-0.608624484	0.004081	0.02029
CARD11	764.1487615	-0.608738596	0.01058	0.04128
ADHFE1	110.554055	-0.608859264	0.01202	0.04534

ITGB8	4424.569107	-0.608992154	0.000199	0.00213
MSI2	2179.547237	-0.609613476	1.43E-06	4.5E-05
TESPA1	109.435603	-0.609798734	0.008078	0.03376
DMTN	1201.63115	-0.609861939	0.001079	0.00759
PAX6	200.6114697	-0.611173432	0.004832	0.02306
LRRC27	182.8110215	-0.611828932	1.19E-05	0.00024
CD33	94.98949924	-0.611985891	0.001641	0.01024
LILRB1	269.7423586	-0.612046187	0.006092	0.0274
PLEKHO1	2142.422305	-0.613359088	0.000245	0.00251
LINC00638	66.89343834	-0.614003623	0.000331	0.00315
MEX3B	251.7549851	-0.615444441	0.000545	0.00458
ARSD	1688.168722	-0.615529622	6.21E-06	0.00015
TMTC4	657.3708319	-0.615776793	5.03E-06	0.00012
PLA2G4C	391.5607784	-0.616101554	0.002571	0.01435
ZNF521	470.2266806	-0.616180341	0.013351	0.04901
ATP2B1	3938.236665	-0.616239136	0.000111	0.00136
TENM4	1933.812685	-0.616694842	0.000558	0.00466
HHIPL1	247.4645537	-0.616820746	0.002119	0.01249
TSPYL2	737.3759753	-0.617623222	1.51E-06	4.7E-05
P2RY8	260.4280645	-0.618040469	0.008324	0.0345
TMEM176	1076.755699	-0.618668559	0.003458	0.01788
SIMC1	400.631434	-0.618744462	0.000818	0.00619
HOXC6	133.8674003	-0.618948702	0.00286	0.01554
ZNF594	194.496969	-0.619022635	3.14E-05	0.00051
LRRC25	257.5531963	-0.619049963	0.002612	0.01452
RCSD1	536.4376024	-0.619088753	0.002979	0.01598
CADPS2	561.0721356	-0.619383085	0.001482	0.0096
GYPC	554.1196514	-0.619490986	0.001905	0.01153
CLIP3	457.849409	-0.619494578	0.001173	0.00807
SIX4	546.5730307	-0.619634995	0.003584	0.01839
HLA-DQA1	5460.357622	-0.619677104	0.012347	0.04623
HLA-DMB	1498.296455	-0.619769555	0.006328	0.02818
U91328.19	129.117839	-0.619992925	0.001291	0.00871
TSHZ2	759.0949259	-0.620012289	0.001341	0.00894
SLC25A29	1056.587885	-0.620183947	2.02E-06	6E-05
GRB10	2114.149207	-0.620221843	0.000882	0.00654
ECM2	447.4424547	-0.620696713	0.002661	0.01474
KLF12	601.0295546	-0.62108828	0.000521	0.00442
ASB9	83.48214084	-0.621118154	0.00608	0.02736
FAM110B	174.8287458	-0.621686288	0.002493	0.014
TIAM2	322.8343179	-0.622098362	8.79E-08	5.2E-06
RCOR2	239.6131112	-0.622319087	0.012385	0.04633
GPD1L	899.2073858	-0.622532498	0.000223	0.00234
TBC1D30	296.93067	-0.623009266	0.003918	0.01969
APOBR	687.8783596	-0.623082663	0.000491	0.00423
FOXE1	3390.9974	-0.623084347	0.008343	0.03455
G6PD	9941.910388	-0.623124988	0.002141	0.01259

TNFRSF19	722.9030586	-0.62334267	0.001795	0.01097
ITGA4	531.2132907	-0.623390179	0.011638	0.04432
CH17-340I	97.28927889	-0.62345661	0.001444	0.00944
NR2F1-AS1	147.7158808	-0.623560687	0.000867	0.00647
SEMA6A	645.7897397	-0.624178753	0.00197	0.01183
DNALI1	92.81957291	-0.624545348	0.003123	0.01657
SPIN3	61.87545226	-0.624985859	0.004885	0.02323
SNX20	341.0727835	-0.625085699	0.003275	0.01716
VWA2	138.244583	-0.625096019	0.009055	0.03671
EPB41L2	1932.580179	-0.62539902	0.000127	0.00151
U2AF1L4	288.5554402	-0.625785743	1.63E-05	0.00031
DCHS1	943.7420286	-0.625861654	0.005674	0.02605
MATK	239.9377474	-0.626189383	0.006329	0.02818
BIN2	329.9102605	-0.627047017	0.002429	0.01372
HOXA5	117.9014369	-0.627085548	0.005149	0.02411
IL2RG	1367.438218	-0.627189185	0.003111	0.01653
RP11-225E	71.28437434	-0.627623192	3.25E-07	1.5E-05
RAB37	89.69533927	-0.62841309	0.004447	0.02163
GPR133	129.8285571	-0.629763307	0.013239	0.04877
GSR	4985.72629	-0.630905922	0.001703	0.01052
NPL	1406.591345	-0.632504237	7.24E-05	0.00098
TMC8	1023.090725	-0.632556375	0.000214	0.00225
PCDHGA6	80.73722675	-0.632678715	0.000882	0.00654
PLD3	11878.83276	-0.6327862	6.21E-07	2.4E-05
IL16	635.6646262	-0.632913176	0.001632	0.01021
CPVL	978.880288	-0.633653071	0.001484	0.00961
CD180	187.7266336	-0.633788951	0.008052	0.03371
CADM1	803.2079329	-0.634660373	0.005088	0.02391
FGFR1	2516.715111	-0.635148388	0.002374	0.01348
LYPD6B	485.1475928	-0.635987921	0.009091	0.03682
AMZ2P1	209.3120332	-0.636069773	0.000183	0.00199
MPP2	142.3864811	-0.636260437	0.0066	0.02901
PTPRC	1874.541188	-0.636442147	0.009914	0.03923
RP11-644F	175.3674076	-0.636641171	3.14E-08	2.4E-06
NLRC3	212.5967623	-0.63722397	0.001601	0.01007
PLCL2	242.3513059	-0.63785129	0.001078	0.00759
CAMK1	114.349583	-0.63847127	3.38E-05	0.00053
RP11-1277	65.55585577	-0.63847421	1.31E-05	0.00026
CHST1	586.4441938	-0.638982771	0.005201	0.02429
MAN1B1-A	117.7883484	-0.639301066	1.81E-05	0.00033
ZEB1-AS1	140.1836619	-0.640292268	2.14E-05	0.00038
PLAC9	117.8802297	-0.640558861	0.003412	0.01771
SLC29A3	434.5991969	-0.640655171	6.67E-07	2.5E-05
AEBP1	21167.75997	-0.640912994	0.004112	0.02039
IGSF21	79.90223482	-0.641108299	0.006045	0.02724
PCDHGA4	66.07766746	-0.641423613	0.008115	0.03388
ZNF517	538.8332185	-0.641791235	1.53E-06	4.8E-05

DUSP2	711.4667097	-0.643333966	0.002864	0.01554
ZNF837	87.05979841	-0.643350572	5.57E-05	0.00079
TCEA3	1269.507933	-0.643438243	0.003829	0.01934
RP11-440D	153.9681291	-0.643643529	0.000604	0.00492
SNAI3	68.97679933	-0.644075845	0.001096	0.00768
SUSD3	162.1586524	-0.644254148	0.000728	0.00566
POLR2J3	314.0338648	-0.644334411	0.005074	0.02385
PIK3R6	87.77160649	-0.644735712	0.000167	0.00186
PSD3	1497.332043	-0.645783122	0.000264	0.00267
TBX2	689.0842137	-0.645816607	0.002158	0.01266
C3	11609.61324	-0.647444269	0.00815	0.034
TRIM24	1394.566633	-0.647494258	2.49E-07	1.2E-05
TNFAIP2	8162.12021	-0.647570205	0.000703	0.00552
ARSE	91.06228194	-0.64790332	0.011473	0.04383
HVCN1	198.2780291	-0.648553082	0.000224	0.00235
DKK2	92.80966403	-0.648614922	0.009672	0.03855
ABHD2	6870.417781	-0.648660228	7.27E-05	0.00098
MS4A6A	1209.389432	-0.650005259	0.001937	0.01167
TMPRSS4	7496.128327	-0.650608438	0.003548	0.01826
CDHR3	84.14588456	-0.650857868	0.000938	0.00683
MZF1-AS1	74.58623372	-0.651145006	1.49E-05	0.00029
FOXF1	200.9956701	-0.651377652	0.00126	0.00854
C3orf70	175.414374	-0.652065141	0.00692	0.03004
CTSS	3463.02197	-0.652350937	0.002156	0.01266
CBFA2T3	367.039865	-0.653377054	0.001499	0.00966
FAM71E1	100.9605673	-0.653729623	0.003799	0.01923
CDH23	233.4660242	-0.653922586	0.003659	0.01871
MYO15B	462.1943385	-0.654112013	0.001891	0.01146
MBNL1-AS	156.6097706	-0.654597082	0.000301	0.00292
MXRA8	3143.650266	-0.654850454	0.000766	0.0059
BTK	252.0771664	-0.655030073	0.000834	0.00628
ZNF862	325.3224373	-0.655285303	6.82E-06	0.00016
NOTCH4	776.3295281	-0.655536171	0.000159	0.00179
SMPDL3B	157.9723239	-0.655715018	0.006537	0.0288
FILIP1	284.1003994	-0.655993444	0.008734	0.03573
PLA2G6	517.4500338	-0.656062279	7.27E-06	0.00017
FREM1	62.5103099	-0.65688389	0.012839	0.04762
RRAGD	796.1850684	-0.657001938	0.00114	0.00791
ORAI2	1443.522965	-0.657783261	1.5E-05	0.00029
CYSLTR2	88.06507266	-0.658249229	0.004301	0.02112
CCDC184	71.1656635	-0.658360738	0.004074	0.02028
SMARCA2	4238.317853	-0.659260012	3.83E-06	0.0001
EVI2B	472.8519603	-0.659946095	0.002324	0.01331
SLCO2B1	1535.977524	-0.660130817	0.003001	0.01607
KCNMA1	804.7290544	-0.660629019	0.006298	0.02808
GPR161	1549.636245	-0.660666439	0.000109	0.00134
ZNF713	119.4062679	-0.661184022	0.000344	0.00323

VMAC	191.4558661	-0.662305068	4.27E-08	3.1E-06
CACNB1	669.4846084	-0.662334958	0.007895	0.03328
TFDP2	2104.391583	-0.662374001	2.14E-07	1.1E-05
TM6SF1	67.2016994	-0.66311623	0.000883	0.00654
DACT1	528.4126817	-0.663256504	0.01301	0.04808
PTPRS	3635.898055	-0.663560771	6.39E-05	0.00089
DIAPH2	1127.851149	-0.663609636	0.000444	0.00393
LIFR	593.4695032	-0.663722333	0.00457	0.02206
TMEM98	1296.257136	-0.6653319	0.000429	0.00383
TSPY26P	174.6170873	-0.665950895	0.002684	0.01484
ST3GAL1	2933.34585	-0.666125276	0.000131	0.00155
DRAXIN	62.90365724	-0.66628828	0.002486	0.01396
INPP5D	1060.687311	-0.666313252	0.000574	0.00475
CD5	348.1302331	-0.666917341	0.007248	0.03114
CD27	306.0761158	-0.667200415	0.011135	0.04285
LINC00963	2010.938095	-0.668315036	1.95E-08	1.7E-06
ISYNA1	1408.125421	-0.668782313	0.001618	0.01015
PKD1P5	65.43185594	-0.668846486	0.011244	0.04312
TNFSF13	291.0317004	-0.669218498	2.07E-06	6.1E-05
ZBTB10	576.8972048	-0.669389286	0.001341	0.00894
SH3BP5	281.2100921	-0.669800394	0.000464	0.00407
SYNGR3	131.5191569	-0.670482784	0.004433	0.02157
SEMA5B	137.4648428	-0.670619567	0.007585	0.0323
MIR600HG	168.4211924	-0.67064397	0.00204	0.01214
GVINP1	145.5169884	-0.670740751	0.010832	0.04196
IPCEF1	102.0546774	-0.670935369	0.000887	0.00656
TP73-AS1	474.0573781	-0.671009729	6.31E-05	0.00088
MAP10	61.02926849	-0.671197191	0.006533	0.0288
LAPTM5	7277.743258	-0.671205643	0.000579	0.00477
DOK5	79.71868848	-0.671423743	0.010697	0.04166
CCR5	367.3178128	-0.67157054	0.005894	0.02674
HTRA3	4052.741153	-0.671650173	0.003096	0.01649
MCCC1	1760.054071	-0.672073956	9.97E-07	3.4E-05
SLC43A1	239.4804645	-0.672088307	0.000118	0.00143
CCNO	219.2318277	-0.672775877	0.000232	0.0024
RP11-764K	71.74132177	-0.673156583	2.16E-05	0.00038
IRF8	605.155404	-0.673760101	0.003388	0.01765
SSPN	849.1483427	-0.673992272	0.001165	0.00803
CPXM1	1356.550829	-0.674086947	0.003316	0.01733
ACADSB	715.0617795	-0.674411566	9.11E-06	0.00019
CD48	478.7190027	-0.674494958	0.005569	0.02573
ZNF43	124.888054	-0.675405141	0.000779	0.00599
NTNG2	80.27753931	-0.675480599	0.000294	0.00288
MAN2A2	1331.078668	-0.675951608	6.66E-06	0.00015
ARHGEF25	493.2413558	-0.676243652	0.000262	0.00266
TTLL1	236.8506387	-0.676256581	6.49E-07	2.4E-05
TET1	142.2790095	-0.676522752	0.001422	0.00936

DACT3	180.9108019	-0.676659732	0.002522	0.01412
GYG2	132.8566291	-0.677991303	0.00229	0.0132
FBLN1	9937.877582	-0.678619577	0.000204	0.00217
ZNF423	300.175493	-0.678768544	0.00298	0.01598
CHST14	1325.285959	-0.680271278	9.8E-08	5.7E-06
RAB3IL1	536.6490005	-0.680319531	5.08E-05	0.00074
IL34	287.8977312	-0.680928299	0.002689	0.01486
FCGR1A	123.1261656	-0.681630825	0.00613	0.02749
SPN	542.5513963	-0.681960417	0.002684	0.01484
C21orf2	656.6214016	-0.682297067	2.44E-06	6.9E-05
NFE2L3	1383.605177	-0.683119922	0.000267	0.00269
SH2B2	244.3842171	-0.683687323	5.93E-06	0.00014
NOS1AP	164.3347704	-0.683764912	0.005109	0.02398
IL17RD	372.5298696	-0.683935094	8.89E-05	0.00113
PXYLP1	640.8597246	-0.684122611	0.000361	0.00336
CLDN15	254.009191	-0.684306949	6.11E-06	0.00015
PRIMA1	254.3879905	-0.685073391	0.00673	0.0294
UNC5C	125.0777454	-0.685097801	0.013037	0.04814
SH2D1A	107.8199549	-0.685234123	0.01198	0.04523
RERG	106.7867562	-0.685452817	0.005589	0.02579
SELENBP1	488.5767857	-0.685507316	0.002249	0.01302
NEIL1	205.4413968	-0.685925729	0.000503	0.0043
DNAH14	165.4400894	-0.686295383	0.01097	0.04233
HMGN5	204.8705427	-0.68633933	0.001067	0.00755
KHDRBS3	332.8815537	-0.68701138	0.003351	0.01747
ITGAL	834.708631	-0.687015443	0.004548	0.02199
FCGRT	2984.401827	-0.687018048	3.49E-06	9.4E-05
PLCB2	483.6460673	-0.687319678	8.64E-05	0.00111
SCCPDH	1277.202721	-0.68737347	2.2E-05	0.00038
ARHGAP4	1215.517582	-0.688175601	0.000818	0.00619
CSF1R	2470.020506	-0.688177679	0.000931	0.00679
HLA-DOA	1084.346415	-0.688888711	0.011523	0.04398
TLDC2	69.87561291	-0.690664496	4.88E-05	0.00071
CLEC11A	874.8723256	-0.690892751	0.001441	0.00944
SPOCK2	1534.730801	-0.6911079	0.002476	0.01393
PIR	1406.956212	-0.691448709	0.000633	0.00511
PLEKHG4	1138.78439	-0.691920814	0.001635	0.01022
C3orf33	182.0525923	-0.691994895	1.63E-05	0.00031
RP11-160C	86.58525402	-0.692204436	0.003527	0.01816
CD200	366.2599881	-0.69226671	0.00095	0.00689
LINC00087	78.2139126	-0.692639013	0.000338	0.0032
DLX6-AS1	126.216069	-0.692641902	0.003307	0.01729
POU6F1	216.0757994	-0.692777542	4.67E-06	0.00012
FBXL14	519.2118175	-0.693127143	2.88E-05	0.00047
EEPD1	450.6585811	-0.693525281	6.25E-05	0.00087
CD1C	96.64828442	-0.693994983	0.002846	0.01549
TMEM176	2038.579656	-0.694242026	0.001342	0.00895

ACSS1	1812.216131	-0.694349193	4.65E-05	0.00069
GNG7	148.1407093	-0.695137992	0.00245	0.01382
RP11-539I	111.1974098	-0.69726696	0.000203	0.00217
APOBEC3F	373.2104821	-0.697372919	1.22E-05	0.00024
PIK3CG	201.5391964	-0.697746879	0.006028	0.02719
PNPLA7	163.1190193	-0.697999551	0.000251	0.00256
TUB	279.0047874	-0.698916225	0.002326	0.01331
ORAI3	644.2099504	-0.699321593	6.47E-07	2.4E-05
RABL2A	173.3479114	-0.699569482	1.06E-06	3.6E-05
IQGAP2	450.8949825	-0.699818094	0.001332	0.0089
ANKRD44	362.929484	-0.700658981	5.79E-05	0.00082
OLFML3	1595.659823	-0.700839888	0.002208	0.01287
PAQR8	319.4375352	-0.700878207	1.76E-05	0.00032
IKZF1	630.4939359	-0.701559566	0.00319	0.01682
MITF	304.1708104	-0.702421439	0.000385	0.00353
RP11-799D	66.72178249	-0.702460918	4.86E-07	1.9E-05
CIART	289.9707501	-0.702734712	0.002071	0.01229
FES	531.7461297	-0.703108968	4.78E-06	0.00012
ABCA2	1629.981793	-0.704154206	2.2E-05	0.00039
IRF5	1025.704844	-0.704708895	3.94E-05	0.0006
GPRASP1	106.293909	-0.704727165	6.86E-05	0.00094
VCAN	9526.708318	-0.704816129	0.007365	0.03156
RP11-983F	151.9540155	-0.705032781	8.39E-05	0.00108
FOXP2	427.6688062	-0.705505335	0.005471	0.02537
STOX2	585.8453351	-0.7059933	1.85E-05	0.00033
METTL7A	1427.085981	-0.706297884	0.00205	0.0122
PRR22	87.8639622	-0.706705549	0.001729	0.01064
GXYLT2	396.8163765	-0.707065569	0.002342	0.01336
LPP-AS2	138.2966105	-0.707232064	1.22E-06	4E-05
TMEM121	80.00310765	-0.707879308	0.000288	0.00284
EFEMP2	1856.257215	-0.708193357	0.000206	0.00219
GKAP1	73.05367046	-0.708492731	0.000399	0.00362
FERMT3	1095.380523	-0.708665777	0.000213	0.00225
ITPKB	2896.534107	-0.708780371	2.34E-08	1.9E-06
ASTN2	450.0308883	-0.708799541	0.006507	0.02872
GAMT	901.9720144	-0.709512859	0.000498	0.00427
RP11-774O	124.0766782	-0.709933635	1.45E-05	0.00028
ST8SIA1	251.1260777	-0.710466105	0.000357	0.00333
ALPK3	866.4768883	-0.710864827	0.010587	0.0413
EPHB6	1281.414718	-0.711563804	0.004266	0.02097
FZD1	2848.062429	-0.712023264	1.84E-05	0.00033
LGI2	136.9137718	-0.712210182	0.00674	0.02943
NUDT11	406.3472083	-0.712263316	0.006637	0.02911
SLC27A5	265.1427327	-0.71263053	0.002307	0.01327
TXNIP	15927.42253	-0.712633614	0.000403	0.00364
MTL5	246.0258775	-0.713240083	0.002362	0.01342
IL4I1	505.0449263	-0.713811245	0.001219	0.00831

CPXM2	1082.133244	-0.713998496	0.002887	0.01563
LOXL1-AS1	253.6027869	-0.714148895	7.31E-05	0.00098
CD27-AS1	512.0078673	-0.715469064	2.05E-06	6.1E-05
CD3G	190.3611692	-0.715924406	0.004113	0.02039
FRK	1372.305219	-0.716181748	1.04E-05	0.00021
SHF	225.419339	-0.717454401	0.000212	0.00224
TMTC1	1585.703705	-0.717783279	0.003627	0.01856
SLC9A9	789.2651794	-0.717784906	0.000229	0.00238
MECOM	734.0167739	-0.71810908	0.000404	0.00365
LINGO1	182.2492948	-0.718158821	0.000425	0.0038
PARM1	759.3983087	-0.718464544	0.000919	0.00672
TTC3P1	122.9574388	-0.71859083	0.000688	0.00543
GMPR	293.40241	-0.718795475	0.001442	0.00944
CACHD1	801.6942675	-0.718952792	9.05E-06	0.00019
HAVCR2	614.1663754	-0.719779369	0.000238	0.00245
ELFN1	315.6631566	-0.720148703	0.003042	0.01625
SERPINA1	2470.508704	-0.72103732	0.004618	0.02224
ACP6	322.0061457	-0.721483366	1.8E-05	0.00033
MANEAL	239.1742956	-0.722051337	0.002132	0.01254
RAB15	922.9342742	-0.722467021	1.55E-05	0.00029
DOCK2	775.4283293	-0.722601652	0.001251	0.0085
DHRS3	3952.5368	-0.722703891	1.29E-05	0.00025
UST	956.2673073	-0.724687398	2.87E-06	7.9E-05
TMEM8B	497.8852699	-0.7248042	0.00022	0.00231
CD4	2181.513937	-0.72491114	0.000478	0.00414
CCDC78	75.03937853	-0.725127046	0.004752	0.02278
ARHGAP33	316.8730714	-0.72551894	0.000104	0.0013
TRO	290.4491929	-0.726277303	0.00239	0.01355
TNFSF8	62.56187085	-0.726952424	0.004323	0.02116
TNFRSF18	1168.29644	-0.727311005	0.002966	0.01592
RSPO3	68.75383108	-0.727534586	0.006487	0.02869
AMPH	107.6472295	-0.727825844	0.003672	0.01876
TMTC2	742.1341266	-0.727887533	6.63E-06	0.00015
GAL3ST4	838.0263917	-0.728237405	5.32E-07	2.1E-05
AC074286	275.9338158	-0.728284108	0.000611	0.00497
TFEC	159.6962724	-0.728391219	0.001478	0.00958
SLC1A1	169.9841751	-0.728439318	0.001714	0.01057
GAA	7048.297939	-0.728745909	7.23E-07	2.6E-05
PTGER2	83.75231305	-0.728972186	0.005193	0.02427
SLC22A17	556.6903632	-0.730384086	0.000242	0.00249
BACH2	341.5349241	-0.730497267	0.000703	0.00552
LY9	109.8511114	-0.730863432	0.006528	0.0288
RAI2	210.3384502	-0.731019711	0.000967	0.00697
PCOLCE	4002.358573	-0.731733646	0.000465	0.00407
DTNA	312.4308989	-0.731886755	0.010959	0.04233
LMO4	3040.420295	-0.732238377	3.91E-07	1.7E-05
SCARA3	1469.222912	-0.732746374	0.000186	0.00201

CCR2	106.1143752	-0.733748032	0.012217	0.04591
BAMBI	550.9252638	-0.735717611	0.003723	0.01896
CITF22-92/	69.14456368	-0.735914425	6.49E-06	0.00015
SLC40A1	2203.869486	-0.736364499	0.000418	0.00376
DICER1-AS	95.05336666	-0.736527858	0.000103	0.00128
QRICH2	143.7523754	-0.737215198	0.000235	0.00243
ZFP14	194.3027659	-0.73721881	1.06E-07	6E-06
DTX3	856.6377151	-0.737540961	2.84E-06	7.8E-05
RALGPS1	278.2527137	-0.738266244	1.81E-05	0.00033
DPYSL3	6642.2791	-0.738863458	0.00034	0.00321
ABHD11	1109.259703	-0.738878401	9.01E-08	5.3E-06
ADSSL1	713.817876	-0.738982194	0.000452	0.00399
PGD	15110.3828	-0.739257309	0.000198	0.00212
C10orf91	97.98177799	-0.739703913	0.003239	0.01701
SCARF2	668.8404269	-0.739741579	0.000671	0.00533
C1QC	5524.786112	-0.739791836	0.000954	0.0069
INHBB	711.3289482	-0.741220875	0.000685	0.00542
MSR1	793.7536929	-0.742509643	0.001827	0.01114
JADE2	4285.777036	-0.742575147	0.000228	0.00238
CDH11	3730.358736	-0.743040657	0.001546	0.00986
OLMALINC	196.4376337	-0.743670326	2.74E-07	1.3E-05
GSPT2	255.7727442	-0.743689001	0.002475	0.01393
RNF180	65.56474142	-0.743816972	0.000177	0.00193
GPR98	560.646941	-0.744102983	0.000106	0.00131
SIT1	122.6111792	-0.74420332	0.003456	0.01788
GCHFR	123.5769616	-0.74548724	7.41E-05	0.00099
LPHN1	2588.983751	-0.745835964	2.96E-05	0.00048
ZDHHC11E	201.4678036	-0.746446689	0.012905	0.04778
RIMS2	286.2122074	-0.747393855	0.011787	0.04478
CDH8	180.1069826	-0.748003808	0.01011	0.03985
NFAM1	449.4644113	-0.748079223	0.000429	0.00383
LINC00997	158.5022702	-0.748572713	1.4E-07	7.7E-06
GPRASP2	317.2151546	-0.748659885	2.02E-08	1.7E-06
FIBIN	392.0521354	-0.748999343	0.005958	0.02697
SIX2	334.8436257	-0.749348904	0.006564	0.0289
ZNF358	1657.72989	-0.749511937	9.28E-06	0.0002
KLRD1	134.19713	-0.749831528	0.002783	0.01522
OLFML2B	2817.036697	-0.75000636	0.000775	0.00596
C1QA	5235.698285	-0.750083995	0.001489	0.00963
SYNM	1472.205375	-0.750255226	0.001676	0.01039
SLAMF6	196.1158736	-0.750299773	0.005513	0.02553
DOK1	400.9957272	-0.750648913	1.01E-06	3.4E-05
PGAP1	1237.291804	-0.751075219	5.66E-07	2.2E-05
PTPRN2	244.0871962	-0.751080053	0.001642	0.01024
UCP2	2320.565782	-0.751367817	7.31E-05	0.00098
RPH3AL	393.427972	-0.751471248	0.00167	0.01037
EGF	128.5666291	-0.751829763	0.010203	0.04013

RP11-742E	98.69435008	-0.752682241	0.01044	0.0409
MGP	2312.763586	-0.752785042	0.00197	0.01183
COL23A1	202.655929	-0.753050733	0.000523	0.00443
HAP1	609.6831799	-0.753173434	0.00824	0.03429
SMCO4	503.6605809	-0.753684227	1.9E-06	5.7E-05
TMEM255	121.3116312	-0.753842605	0.001979	0.01186
ZBTB12	211.3142929	-0.754354904	2.45E-07	1.2E-05
ATP1A3	65.64162051	-0.754442369	0.001375	0.00911
PLEKHA7	870.5042945	-0.755156312	4.89E-05	0.00071
SLC2A5	528.0107255	-0.755490476	0.00066	0.00527
TNRC6C	623.250448	-0.755677767	1.17E-10	2.6E-08
VSIG4	545.7225374	-0.756853125	0.004822	0.02304
LY86	144.183754	-0.757608004	0.000325	0.00311
PTH1R	63.64362266	-0.758704615	0.001867	0.01134
CREB3L1	1188.75494	-0.75944036	0.001637	0.01023
KIAA1211	68.14227023	-0.759454273	0.002353	0.01341
PIK3R3	979.9713409	-0.759737256	2.36E-07	1.2E-05
SOX4	5364.350773	-0.759762139	7.38E-07	2.7E-05
RP11-254F	100.5127387	-0.760190079	3.58E-07	1.5E-05
DUOX2	2785.101324	-0.760756735	0.012445	0.0465
SIDT1	217.1206413	-0.763402499	0.002254	0.01303
RP11-295M	107.6128137	-0.763496834	0.004835	0.02307
GADD45G	253.9757333	-0.76409172	0.001278	0.00864
PTP4A3	1815.477217	-0.764124833	8E-05	0.00105
P3H3	1761.367889	-0.764421354	0.000686	0.00542
COL9A2	673.4915361	-0.764600048	0.001444	0.00944
NEO1	2842.616645	-0.76568241	3.14E-08	2.4E-06
FOLH1	185.2807999	-0.765738319	0.001607	0.0101
COL8A1	2359.096333	-0.765846352	0.003257	0.01708
COL1A2	190554.5408	-0.766478018	0.003708	0.01889
ARHGAP2(	87.45022583	-0.767384528	0.001077	0.00759
WNT2B	840.4047223	-0.767610498	0.002893	0.01565
ZCWPW1	188.8800794	-0.767884041	1.8E-06	5.5E-05
ALOX5	634.2107053	-0.768076095	0.000154	0.00175
LAIR1	751.46686	-0.768249571	0.000268	0.0027
STAR	185.880239	-0.769745851	0.010876	0.04207
EDIL3	1819.494203	-0.769992507	0.000896	0.00659
PPP1R3F	232.8322381	-0.771278383	9.94E-07	3.4E-05
ROR1	168.9851439	-0.77231339	0.000896	0.00659
MYLIP	1107.584536	-0.773186956	8.54E-06	0.00018
LSAMP	446.3782865	-0.774367407	0.006085	0.02738
HLA-DQB2	1462.399618	-0.774839163	0.005993	0.02707
SLC4A11	1143.385353	-0.775387997	0.000567	0.00471
SEMA6C	571.9532558	-0.775419782	0.000564	0.00469
DCLK1	427.9987211	-0.775828902	0.005224	0.02438
ST6GAL1	1932.655722	-0.775948028	0.000838	0.0063
SDSL	391.0988727	-0.776420305	5.35E-05	0.00076

NAP1L3	58.29957873	-0.776518604	0.00287	0.01557
GZMK	134.619337	-0.777475727	0.012629	0.04706
WDFY4	446.0665672	-0.777824568	0.002537	0.0142
ZNF711	223.8248892	-0.778230504	0.001295	0.00872
GLI2	803.4484127	-0.779256745	0.007024	0.03038
GNGT1	120.7880791	-0.779392488	0.001037	0.00739
EPB41L1	3381.875283	-0.779878789	5.99E-05	0.00084
STK31	129.7594427	-0.780101717	0.002209	0.01287
RENBP	346.9993814	-0.780385198	3.76E-05	0.00058
DEPTOR	393.3366234	-0.781397455	0.000627	0.00507
PDZD4	186.583546	-0.781745545	0.000341	0.00322
VAV1	478.2685291	-0.781916916	4.05E-05	0.00061
NAPSB	330.4305922	-0.782792108	0.001292	0.00871
PRKAR2B	205.0042416	-0.783628175	2.92E-05	0.00048
BRSK1	118.9406494	-0.783845915	9.3E-05	0.00118
SEZ6L2	1660.896269	-0.784037497	0.001554	0.0099
SULF1	8124.694696	-0.784626255	0.001435	0.00941
HCLS1	2068.327709	-0.785030431	6.63E-05	0.00092
PKIB	182.3664919	-0.785993184	0.000187	0.00202
APBB1IP	376.1848238	-0.7865365	0.000155	0.00176
NCKAP1L	1149.48568	-0.786599109	0.000295	0.00288
CCDC80	5066.452393	-0.786837767	0.000415	0.00374
GALNT5	776.7815573	-0.786936334	0.007263	0.03119
ETV5	1440.981961	-0.787084504	3E-05	0.00049
SIGLEC14	82.92236921	-0.787085268	0.003865	0.01948
TRIM45	228.2901712	-0.78732484	1.82E-05	0.00033
VASH2	138.7826606	-0.788004619	0.001538	0.00982
CBX7	849.7351533	-0.789767864	1.61E-07	8.4E-06
CHPT1	686.2122043	-0.790312918	1.48E-05	0.00029
NPR3	692.1661666	-0.790534706	0.008563	0.0352
PAXIP1-AS	263.7387993	-0.790658936	2.93E-07	1.3E-05
RYR1	2823.646797	-0.791302285	0.001538	0.00982
EN2	153.8502325	-0.791633574	0.009395	0.03775
TXNRD1	8446.287458	-0.791696222	0.000779	0.00599
MMP11	7657.48169	-0.791734207	0.00772	0.03271
MAN1C1	271.1215076	-0.79253956	4.96E-05	0.00072
FAM117B	1209.919718	-0.792848273	1.09E-06	3.6E-05
LONRF1	731.1054016	-0.79335068	1.1E-07	6.2E-06
RP11-147L	107.8665798	-0.793554589	1.78E-06	5.4E-05
RASD2	449.2269228	-0.793641817	0.003235	0.01701
PYHIN1	73.38024512	-0.793716265	0.001654	0.01029
TMEM132	90.1274386	-0.794186324	0.000327	0.00313
ISLR	4595.522276	-0.794275269	0.001453	0.00947
PCDHB10	151.1302931	-0.796380871	4.66E-06	0.00012
TLR1	331.3904747	-0.797243368	4.81E-05	0.00071
CX3CR1	84.18973923	-0.798013774	0.001537	0.00982
GAREML	402.6953574	-0.799096373	7.66E-06	0.00017

APOD	814.4619563	-0.799258203	0.003854	0.01944
CD1A	390.5834346	-0.799329924	0.005697	0.02612
SNED1	394.8185171	-0.799584215	0.000796	0.00607
GNAZ	114.3465385	-0.800871948	0.000883	0.00654
CECR1	1783.389553	-0.801464818	0.000524	0.00444
ZNF385C	77.18998639	-0.801496075	0.001033	0.00738
CLSTN2	555.3993372	-0.802413417	0.004481	0.02174
TUBBP5	177.8503045	-0.802617182	0.002675	0.01481
TIGD7	68.56241931	-0.802957319	1.11E-06	3.7E-05
SERTAD4	763.1819196	-0.802980241	3E-05	0.00049
ANKRD6	295.6009587	-0.804147846	0.000107	0.00132
SYNGR1	858.8467197	-0.804238695	0.000326	0.00312
CYBB	1862.733403	-0.804337549	0.001133	0.00788
IKZF3	639.7672403	-0.804795394	0.0021	0.01241
KRT8P12	446.1260608	-0.805008701	4.36E-08	3.1E-06
MAP1A	1226.980332	-0.805089774	0.000645	0.00517
DDN	393.1186234	-0.805264989	0.013584	0.04973
ADAMTSL1	169.2751129	-0.806662775	0.000467	0.00409
CENPV	238.7285948	-0.806719587	0.000303	0.00294
PEG3	72.04426719	-0.806883239	0.004932	0.02338
KCNQ1	380.6679746	-0.80805654	1.9E-06	5.7E-05
FCER1A	189.4549624	-0.80823524	0.006756	0.02948
C14orf37	260.1159253	-0.810061951	9.88E-06	0.00021
USP13	1061.005405	-0.81039683	4.51E-06	0.00011
ENPP4	201.4284422	-0.810920333	0.001456	0.00948
AC144652	75.3606623	-0.810972654	3.57E-06	9.6E-05
AP3B2	153.1310792	-0.811089503	0.009489	0.03801
GPR85	71.18577033	-0.811246309	3.73E-05	0.00058
VAX2	60.05461557	-0.813215695	0.002355	0.01341
C1QB	6167.740933	-0.813648352	0.000667	0.00532
ITGAM	648.4211493	-0.814304061	0.000475	0.00413
RP11-620J	113.3436182	-0.814669211	4.09E-05	0.00062
SIX1	550.7170769	-0.815510165	7.22E-06	0.00016
P4HTM	532.191557	-0.815882723	2.61E-08	2.1E-06
CBX2	1536.53751	-0.81609604	1.38E-05	0.00027
ETNK2	1110.540257	-0.816223921	8.72E-05	0.00111
TMEM56	156.4660807	-0.816390699	0.000953	0.0069
FAM69B	171.6073654	-0.816747855	1.24E-05	0.00025
CYP2S1	4543.059063	-0.816997507	0.000301	0.00292
C4B	140.597803	-0.817099298	0.003211	0.01692
CBX6	3704.987645	-0.818522987	1.05E-06	3.5E-05
EFNB3	318.4891753	-0.819431237	0.000567	0.00471
RASL10B	92.63882238	-0.819729721	0.001452	0.00947
MEGF10	280.1970477	-0.819823779	0.009491	0.03801
SARDH	108.7829227	-0.820513277	8.09E-05	0.00106
CYP2E1	184.2669109	-0.822294497	0.008275	0.03437
ACSS3	84.5069964	-0.822527915	0.000395	0.0036

F5	139.6413766	-0.823239194	0.001304	0.00877
N4BP2L1	254.3585143	-0.823576081	8.4E-07	3E-05
LILRB4	790.71763	-0.823630845	0.001595	0.01004
ZNF771	179.6259798	-0.824512826	3.87E-07	1.6E-05
MFI2	2017.702453	-0.82669768	0.00029	0.00285
PLEKHG4B	658.9228799	-0.827133826	0.001233	0.00841
PRR15L	127.2552115	-0.82787148	0.009927	0.03927
PRKCB	380.9227863	-0.828788291	0.000356	0.00333
TRPV4	1232.585216	-0.828922713	5.57E-06	0.00014
OBSCN	2728.825935	-0.829069644	0.000252	0.00257
CORO2B	105.7437713	-0.829684145	0.00192	0.0116
NR2F1	280.5129256	-0.830144261	1.6E-05	0.0003
TLR7	123.7159115	-0.831140703	0.002322	0.01331
ACACB	387.4152337	-0.831927226	6.38E-06	0.00015
FCRLA	95.77233671	-0.833202056	0.005841	0.02657
MAP4K1	370.2363005	-0.833318447	0.000159	0.0018
RP11-134C	66.98615758	-0.833412562	3.45E-07	1.5E-05
GTF2IRD2E	88.98947785	-0.833803835	1.87E-10	3.6E-08
SPIRE2	311.0729567	-0.835161406	0.000181	0.00197
CYS1	223.0989697	-0.836357607	0.000476	0.00414
ATP8A1	247.9632781	-0.836954854	0.000171	0.00188
YJEFN3	89.12803883	-0.837013648	0.001573	0.00997
ATP6V1B1	74.41954595	-0.840759384	0.002353	0.01341
DLGAP1-AS	156.3150927	-0.841561091	0.000357	0.00333
GPR173	79.41184839	-0.84168933	0.000174	0.00191
PCDHB12	103.3569036	-0.842104772	0.000162	0.00182
RASL11B	249.5284063	-0.842778939	0.001208	0.00827
COL1A1	349299.5431	-0.843671108	0.001278	0.00864
PGPEP1	595.6157569	-0.843970684	5.76E-09	6.2E-07
C5	194.9588087	-0.844417791	5.82E-06	0.00014
ROR2	959.9961318	-0.844789436	0.000619	0.00502
AC093627	70.23376841	-0.845397052	2.23E-05	0.00039
TMEM38A	399.276222	-0.845428105	0.000468	0.00409
CACNA2D4	89.53005604	-0.848928817	1.09E-06	3.7E-05
IDUA	444.0157748	-0.84952319	3.75E-07	1.6E-05
TCN2	960.5845698	-0.850976472	8.11E-06	0.00018
KIAA1755	188.2055053	-0.851367126	0.000274	0.00274
PATZ1	1307.161947	-0.851693068	7.26E-15	5.5E-12
FOXO6	99.62313841	-0.852216506	0.000276	0.00276
KCNC3	190.9912552	-0.854261798	0.000181	0.00197
CGNL1	391.6296584	-0.854593711	0.000627	0.00508
SLC2A11	199.4383388	-0.854775479	2.65E-07	1.2E-05
PLD4	147.7871529	-0.855501368	0.00033	0.00314
LZTS1	552.9067568	-0.855675998	7.08E-06	0.00016
CSRNP3	88.87163742	-0.855717012	0.000163	0.00183
CARD9	207.463078	-0.85598452	1.74E-05	0.00032
DPT	813.0200037	-0.856104379	0.003713	0.01891

KCNJ5	207.2819152	-0.856560324	0.00121	0.00828
FN1	137436.3911	-0.858205817	0.004858	0.02313
ADAM28	357.6203315	-0.858879784	0.000463	0.00407
CD1E	93.46686512	-0.86138007	0.001612	0.01012
HOXA10	258.4976282	-0.861986391	0.000279	0.00278
GCLM	3084.143403	-0.863442582	0.000231	0.0024
RARRES2	1695.285954	-0.863585066	0.000845	0.00633
MAPK8IP2	533.738029	-0.864536255	0.000685	0.00542
GZMH	163.0056241	-0.865051492	0.005704	0.02613
TMEM52	111.7448417	-0.865678559	0.000923	0.00674
ANKRD65	879.8562348	-0.866102894	8.71E-05	0.00111
SSTR2	87.53786463	-0.866161654	5.72E-05	0.00081
ITGA11	1419.23259	-0.866745333	0.00145	0.00946
ALDH5A1	791.748621	-0.869049717	3.8E-06	0.0001
LTBP3	3842.367111	-0.869289438	5.67E-09	6.1E-07
RAB42	124.018185	-0.869651412	1.7E-05	0.00032
MXRA5	10956.67115	-0.871821282	0.000267	0.00269
TFR2	129.5908289	-0.872456155	0.000133	0.00157
ARHGAP28	383.4599122	-0.873049722	0.000156	0.00177
ATP10A	351.7235969	-0.873104542	2.96E-05	0.00048
SYT17	320.7236639	-0.873476774	0.000362	0.00336
KLHL24	2927.247703	-0.874299836	1.84E-08	1.6E-06
ZBED3	230.9554102	-0.874313063	8.63E-08	5.1E-06
WBSCR17	117.8353104	-0.87472926	0.00072	0.00563
ADCK3	1732.877162	-0.876435872	6.86E-07	2.5E-05
CH25H	337.5231534	-0.876919577	0.000658	0.00526
HK3	307.3000511	-0.877967463	0.000545	0.00458
NRN1	554.1939088	-0.878831202	0.00187	0.01136
RGAG4	135.8463184	-0.878994081	1.66E-05	0.00031
C17orf97	134.4573548	-0.879472326	0.000127	0.00151
GLB1L2	336.2574458	-0.879593319	0.006778	0.02956
RARB	272.1023548	-0.879983844	0.000645	0.00517
MAP2K6	161.2824663	-0.880792518	7.32E-06	0.00017
CNIH3	159.6953515	-0.881078759	4.69E-05	0.0007
FN3K	283.0752765	-0.881884136	2.69E-06	7.5E-05
CTA-221G9	70.09957203	-0.882456652	0.004423	0.02154
GABRR1	87.59184875	-0.884082579	0.006167	0.0276
MARC1	350.1365962	-0.885296219	0.000652	0.00522
FARP1	1713.665989	-0.885704653	4.81E-06	0.00012
SRXN1	323.2610168	-0.88633026	0.000118	0.00143
BDNF	126.3696064	-0.886553799	0.000731	0.00567
MUC20	703.2084031	-0.886587428	0.010205	0.04013
SYCP2	130.4618137	-0.887241496	0.002124	0.0125
HOXA-AS2	133.0783489	-0.890464656	0.000747	0.00578
TRAPPC6A	670.9735014	-0.892199268	5.42E-08	3.6E-06
NMNAT3	472.9970313	-0.892289416	3.38E-06	9.1E-05
RP11-395C	152.0758196	-0.892726838	0.00033	0.00314

TLE2	404.2973432	-0.893247409	3.97E-05	0.0006
NTN1	1949.324354	-0.893800085	9.24E-06	0.00019
BEGAIN	86.38876192	-0.894280618	0.00016	0.0018
ICK	1721.866127	-0.894456436	2.65E-08	2.1E-06
SARM1	135.1047182	-0.894530128	2.27E-06	6.6E-05
RAB40B	885.0382279	-0.895121075	3.12E-10	5.7E-08
HOXA4	66.85211658	-0.895237728	0.000478	0.00414
POU2AF1	690.9840209	-0.895413558	0.001796	0.01098
PRKD1	178.3868138	-0.896776904	4.28E-05	0.00064
CD200R1	77.42527388	-0.896965042	5.29E-05	0.00076
EGR2	903.4483515	-0.898001643	0.000328	0.00313
FZD10-AS1	292.4271704	-0.898278168	3.99E-05	0.00061
BNC2	425.0342222	-0.898469283	0.000492	0.00424
DAPL1	1221.084673	-0.899442815	0.012237	0.04595
GFI1	163.4553413	-0.901608558	1.04E-05	0.00021
HOXA3	232.9979109	-0.901706227	6.14E-06	0.00015
FAM221A	81.05798257	-0.902217824	2.71E-05	0.00045
KHK	256.12105	-0.902503834	8E-06	0.00018
MEG3	711.9616955	-0.903203679	0.001744	0.01072
RP11-51F1	95.63942184	-0.903836813	5.46E-06	0.00013
CD37	680.7169258	-0.904059205	3.14E-05	0.00051
CXCR4	1844.073529	-0.904592356	1.56E-05	0.0003
C6orf183	115.4489291	-0.905763029	0.000562	0.00468
CHADL	135.9589032	-0.906024875	1.04E-05	0.00021
FGFR2	2713.30803	-0.907303523	2.37E-06	6.8E-05
ITGB2	4149.814601	-0.908047054	2.6E-05	0.00044
LINC00519	210.0591806	-0.909347019	0.000523	0.00443
SSC5D	615.7565911	-0.909785053	0.000135	0.00158
NEURL1	199.8739802	-0.910879576	0.000893	0.00658
FTCDNL1	87.52810243	-0.911593137	1.07E-05	0.00022
HHEX	233.7313111	-0.913107351	3.97E-06	0.0001
LGR5	308.7573978	-0.913400481	0.010154	0.03997
SLC12A7	2742.589752	-0.914205811	2.28E-06	6.6E-05
BANK1	123.6887129	-0.914887303	0.000158	0.00179
APOBEC3D	242.7798568	-0.915397727	4.37E-06	0.00011
OPRL1	86.31930217	-0.916201627	7.62E-07	2.7E-05
FNDC5	77.66399675	-0.916612727	0.002313	0.01329
SVIP	278.9358958	-0.917546004	0.000163	0.00183
PLEKHB1	115.6923306	-0.918113045	0.000178	0.00195
CA11	455.5323733	-0.919592348	1.06E-07	6E-06
RGMA	1838.276105	-0.919941127	8.54E-05	0.0011
ADM2	392.2135011	-0.920696577	2.6E-05	0.00044
ANK2	320.9900084	-0.920817978	0.000712	0.00557
HOXB6	74.27679575	-0.920835976	0.000169	0.00187
CXorf57	256.9242852	-0.92101615	8.12E-05	0.00106
WIPF3	313.9747014	-0.922926338	6.78E-05	0.00094
RNFT2	154.3709099	-0.923400937	0.002066	0.01227

EMILIN1	3784.724229	-0.924924197	0.0001	0.00126
SOX21-AS1	294.3533268	-0.926362735	0.001293	0.00871
CTB-113P1	266.2801358	-0.926653832	0.000644	0.00517
RP4-665J2	66.44312951	-0.927167628	4.57E-05	0.00068
SKAP1	148.4689962	-0.929347643	4.91E-05	0.00072
WDR86	100.0428396	-0.929642067	8.26E-05	0.00107
GABRA3	322.4148206	-0.929908718	0.00014	0.00163
SERPINI1	231.3147669	-0.930173486	1.57E-05	0.0003
ZBTB18	659.6870104	-0.930216508	4.66E-11	1.2E-08
PRAM1	80.77192673	-0.930667146	1.14E-05	0.00023
GPT	104.4711762	-0.932090228	0.000226	0.00236
RADIL	101.7216565	-0.932408905	3.69E-05	0.00057
GATM	698.5258478	-0.932980161	4.73E-05	0.0007
CD79A	727.8830136	-0.933216555	0.012748	0.04736
TRIM9	149.2254232	-0.933967401	0.000279	0.00278
ZSWIM5	161.4840941	-0.934045102	0.000135	0.00158
MAP6	70.00812722	-0.93469795	0.000112	0.00137
AR	132.5402593	-0.93490415	0.000291	0.00285
AC009501	70.06364874	-0.935052024	0.000221	0.00232
DPYSL4	246.9391618	-0.935289363	0.00191	0.01156
KCNMB3	70.81007991	-0.93663249	1.38E-05	0.00027
NBEA	128.1844399	-0.937846342	0.000283	0.0028
AMIGO1	221.5244207	-0.938055992	1.46E-06	4.6E-05
EPHA3	238.9979466	-0.939192572	0.000517	0.0044
PRKG2	83.5844645	-0.939521552	4.62E-06	0.00012
C6orf223	175.1220354	-0.940856482	0.007132	0.03074
REPIN1	2521.774057	-0.941849222	2.43E-10	4.6E-08
ICA1	210.3246784	-0.942212634	9.23E-06	0.00019
SIGLEC10	379.8311796	-0.943978339	3.68E-05	0.00057
GBGT1	243.0624982	-0.944190499	3.45E-07	1.5E-05
SNCAIP	406.3474237	-0.950184081	1.39E-05	0.00027
ICOSLG	61.81217955	-0.950262252	0.000664	0.0053
LRRC37A3	99.45012582	-0.951704528	1.83E-08	1.6E-06
HRASLS	91.55799428	-0.951866589	0.002057	0.01224
PLA1A	76.77742947	-0.95254908	0.000232	0.0024
LDOC1	747.0668764	-0.953594569	0.000559	0.00466
LEF1	737.6613952	-0.954409746	6.97E-08	4.3E-06
ADAMTS16	72.06248949	-0.954773644	0.005597	0.0258
C1orf115	546.8027083	-0.955818002	1.37E-06	4.4E-05
C3orf58	1885.244888	-0.956175044	4.05E-08	3E-06
ADRA2A	305.4503163	-0.956481667	0.001904	0.01153
MANSC1	1393.237965	-0.957251106	7.44E-06	0.00017
RP11-54O7	122.6264889	-0.957697129	0.000569	0.00471
LRRC15	3169.751762	-0.957709559	0.002883	0.01563
TSPAN7	1468.598377	-0.95961568	0.000562	0.00468
PARVG	495.6043566	-0.959706854	1.3E-05	0.00026
MAPK10	327.6352498	-0.960724729	0.00022	0.00231

GPC4	1562.371424	-0.960743704	0.000724	0.00565
GREB1	125.4036474	-0.962618858	1.27E-06	4.1E-05
CTF1	252.0633075	-0.965019254	7.41E-06	0.00017
TRPM2-AS	98.93224653	-0.965171947	0.003007	0.01609
SLITRK4	95.97039035	-0.965909684	0.005639	0.02595
PODN	759.2369479	-0.967969781	0.000837	0.00629
RP4-568C1	376.6123214	-0.968259259	0.000139	0.00162
NPDC1	1396.994028	-0.968394251	2.47E-06	7E-05
CABYR	254.339376	-0.971342423	0.00014	0.00163
PEG10	886.5624883	-0.971469511	0.011808	0.04484
SLC29A2	633.4980123	-0.973003853	1.71E-06	5.2E-05
ATP2A1	460.0326547	-0.97574027	0.008698	0.03561
SMARCD3	556.2238501	-0.978062951	3.51E-07	1.5E-05
FAHD2CP	120.1049394	-0.978077652	5.02E-06	0.00012
FADS2	3462.143116	-0.978220081	1.26E-05	0.00025
PRDM16	99.6383207	-0.978343385	0.000158	0.00179
SIM2	269.9175686	-0.979925059	7.5E-06	0.00017
PDE9A	268.2499439	-0.980951974	8.34E-05	0.00108
SCML2	88.40143185	-0.981345892	4.12E-05	0.00062
GAS6-AS1	106.4964043	-0.981361612	8.31E-05	0.00107
ARHGAP24	533.4010594	-0.98361404	6.23E-08	4E-06
HOXC8	100.185197	-0.984843156	0.000255	0.0026
KLHL23	349.2317454	-0.985336125	7.1E-06	0.00016
SYT14	67.92173511	-0.985342193	0.00279	0.01526
LRFN1	208.6213174	-0.985976486	1.6E-05	0.0003
TNN	113.3537014	-0.989678922	0.001385	0.00917
ABI3BP	563.0646276	-0.991685225	0.000799	0.00609
PTPRO	134.1070665	-0.993124553	1.18E-05	0.00024
SLC2A12	409.1585538	-0.99336073	0.000101	0.00127
USP51	75.22344849	-0.993683411	4.58E-09	5.1E-07
C16orf95	86.97440829	-0.993731173	6.31E-08	4E-06
VCAM1	1089.248856	-0.99390707	0.000232	0.00241
CYP1B1	1110.521835	-0.994353497	0.001849	0.01125
P2RX5	91.53480418	-0.994789971	5.06E-05	0.00073
ADAMTS13	126.9015544	-1.001434219	1.85E-05	0.00033
PHGDH	3150.804078	-1.004622288	2.52E-05	0.00043
MFAP4	1773.586505	-1.005876068	0.001022	0.00731
TMEM119	867.8776114	-1.00613703	0.000118	0.00143
BEX4	612.5326292	-1.007247075	7.69E-06	0.00017
DIRAS1	357.6494521	-1.007661699	0.000964	0.00695
CXCL17	1392.491966	-1.007680013	0.004614	0.02223
LRP3	917.8004433	-1.008223488	6.92E-08	4.3E-06
CD8B	114.3621931	-1.009246687	0.000998	0.00716
PKN1	3092.271631	-1.009848776	1.58E-10	3.1E-08
PPM1L	882.878021	-1.009936981	9.28E-07	3.2E-05
MAP1B	2995.937765	-1.009991802	1.6E-05	0.0003
NOTUM	153.3071348	-1.011231696	0.000277	0.00276

SDPR	431.0342117	-1.011423503	1.66E-05	0.00031
SLC52A1	350.0390504	-1.014788157	2.72E-06	7.6E-05
B3GNT7	467.0370498	-1.015776059	1.59E-06	4.9E-05
PDE3B	314.7459561	-1.017310151	2.25E-06	6.6E-05
SYDE2	79.76355043	-1.019213382	8.01E-07	2.8E-05
DISC1	243.0259857	-1.020103435	2.66E-08	2.1E-06
DYRK1B	847.9430892	-1.02337435	1.83E-09	2.4E-07
LHX2	101.5324901	-1.02703485	0.000495	0.00425
TENM1	149.0790805	-1.028106525	0.01053	0.04113
LYRM9	126.302216	-1.02810875	2.88E-08	2.3E-06
BCL2	456.1150227	-1.028258751	6.85E-09	7.1E-07
TRIM16L	1499.385975	-1.028906618	7E-05	0.00095
PRKX	2634.867056	-1.0292135	4.86E-09	5.3E-07
TMEM150	273.8658835	-1.030230768	5.71E-08	3.8E-06
CD84	494.6082058	-1.031021282	2.31E-05	0.0004
TTC28	725.6222123	-1.031705256	1.36E-07	7.5E-06
BHLHE41	950.1602662	-1.033121884	5.26E-06	0.00013
PDGFRL	439.7748288	-1.037229257	0.000112	0.00137
CHST10	378.8207809	-1.039556747	4.38E-06	0.00011
MVB12B	523.9720885	-1.041445443	1.55E-10	3.1E-08
PLEKHS1	96.98702833	-1.044893177	0.012948	0.04791
HRK	87.16185648	-1.047025332	0.000435	0.00386
LGALS9	2156.805616	-1.047825539	4.16E-08	3.1E-06
DGCR5	105.1808217	-1.04830396	3.7E-05	0.00057
GALNT12	441.4114763	-1.048336078	5.34E-07	2.1E-05
SLIT2	485.1864466	-1.048947844	2.25E-05	0.00039
DLX6	163.5111103	-1.048971692	0.000165	0.00184
PRR15	165.0888985	-1.049214341	0.000341	0.00322
PCDHB8	73.40275882	-1.049494482	0.000642	0.00517
FMO3	192.9248745	-1.050537086	0.000225	0.00235
SPSB4	69.76136354	-1.054029815	0.000416	0.00374
SLCO1A2	144.9170094	-1.056060801	0.005109	0.02398
TRPV6	73.41015155	-1.056739975	0.00137	0.00909
SMO	1651.015794	-1.058003134	1.63E-07	8.4E-06
MMP7	2875.295641	-1.061330547	0.001114	0.00778
RNF165	239.4066909	-1.063757652	1.37E-05	0.00027
EPHX1	5553.873983	-1.064829537	4.34E-08	3.1E-06
MFI2-AS1	122.1387982	-1.064846731	4.1E-06	0.00011
SLAIN1	74.71703081	-1.065740662	4.21E-05	0.00063
GALNT13	120.3580231	-1.065783095	0.000592	0.00484
MYH14	9418.878934	-1.068444249	3.38E-05	0.00053
PRELP	1162.390995	-1.068451189	0.000554	0.00463
ZNF703	2882.766478	-1.06884289	1.74E-08	1.5E-06
SAMD5	245.8994405	-1.069242137	0.000184	0.002
PELI2	428.3895237	-1.069849644	1.83E-06	5.5E-05
NUPR1	4289.207423	-1.070423295	6.73E-06	0.00016
LRRN1	240.4444047	-1.071156892	0.003202	0.01687

RP3-323A1	83.57209375	-1.071562849	0.001074	0.00758
GSTM5	66.74787645	-1.071824639	1.59E-05	0.0003
PTGDS	748.357264	-1.072200657	6.82E-05	0.00094
MIR4697H	79.59555828	-1.073969721	0.000268	0.00269
KLHDC9	64.87420986	-1.075430231	8.41E-05	0.00108
CLDN11	89.88618735	-1.076892541	5.59E-06	0.00014
CACNA2D1	890.4609333	-1.07703583	2.75E-05	0.00046
PNMAL1	252.7431722	-1.077087334	0.002754	0.01511
DAGLA	183.2738622	-1.077503361	1.49E-07	7.9E-06
PBX1	1718.359498	-1.078599447	2.15E-06	6.3E-05
MUM1L1	99.59350319	-1.079204259	0.002828	0.01541
KIAA1683	81.49439175	-1.081150699	2.57E-05	0.00043
GATS	417.14104	-1.081353827	2.77E-09	3.4E-07
HOXB4	113.7210988	-1.082069948	0.000342	0.00322
LRMP	250.8976737	-1.082491561	6.92E-05	0.00094
SLC7A2	800.73072	-1.083188348	8.02E-05	0.00105
ADAMTSL3	83.45150942	-1.083689266	0.000166	0.00185
MYO5C	583.8881186	-1.083775394	2.53E-05	0.00043
CCDC144N	168.1671711	-1.084668964	0.003411	0.01771
CHODL	208.7422692	-1.086684439	6.88E-05	0.00094
FMO2	804.2830634	-1.086940181	0.00462	0.02225
MEX3A	507.1422593	-1.088765466	6.07E-07	2.3E-05
PCBP3	140.1851952	-1.090129645	3.23E-06	8.8E-05
EXOC3L4	140.0083344	-1.091066271	0.00084	0.0063
AKR1C3	7813.753983	-1.091634475	0.002302	0.01325
PAMR1	497.8814112	-1.092869142	2.61E-05	0.00044
BMP7	3292.997256	-1.093291681	2.12E-06	6.2E-05
RP11-284F	76.66022186	-1.093543129	0.000613	0.00498
HOXB2	262.0047226	-1.095911237	2.01E-05	0.00036
TNIK	440.9376159	-1.097109919	1.93E-06	5.8E-05
HHAT	412.3076845	-1.097445681	3.48E-13	1.5E-10
EPS8	1396.808642	-1.097754263	2.6E-06	7.3E-05
REEP2	118.5452902	-1.099055602	4E-06	0.0001
KCNMB2-A	199.8726016	-1.103890027	1.87E-05	0.00034
CST1	672.1748441	-1.104068353	0.012083	0.0455
NSUN7	84.77989201	-1.105634042	5.69E-05	0.00081
ITGBL1	396.79009	-1.107848487	0.000543	0.00457
COLEC11	90.45572786	-1.112955905	0.000296	0.00288
MAOB	613.9982804	-1.114639543	0.000391	0.00357
LDB3	426.4761736	-1.114898731	0.004464	0.02167
GCLC	6579.988948	-1.11540451	2.78E-07	1.3E-05
ALDH1L1	329.1061796	-1.116411743	0.000778	0.00598
CNTN4	83.76063873	-1.118585314	0.000235	0.00243
KCNMB4	203.2627657	-1.119425047	2.8E-06	7.7E-05
CHP2	224.0921189	-1.119537407	0.002425	0.0137
CORIN	115.2645646	-1.119657456	4.19E-06	0.00011
EDAR	147.0945841	-1.120676261	7.54E-05	0.001

PPARG	149.2887457	-1.122085119	2.48E-05	0.00042
CAND2	202.6525319	-1.122244307	5.33E-05	0.00076
NT5M	121.8376584	-1.122287787	3.66E-06	9.8E-05
EBF4	493.4150544	-1.12330678	7.82E-09	7.9E-07
GULP1	342.8716201	-1.124281046	1.82E-05	0.00033
SULT1A1	339.34359	-1.124624756	2.54E-07	1.2E-05
C15orf59	220.4383865	-1.125578305	4.85E-05	0.00071
AC005077	97.72072672	-1.126208203	0.000133	0.00157
MS4A1	112.0899886	-1.12776246	0.004514	0.02186
CHL1	1205.403019	-1.128055908	0.001639	0.01024
ADRA2C	233.6814555	-1.128398847	3.31E-05	0.00052
ITPKA	129.2879558	-1.128750564	1.45E-07	7.8E-06
PKDCC	231.8586166	-1.130769587	0.000174	0.00191
TDRD5	180.6303554	-1.132835818	0.013284	0.04885
TRIM2	2251.632046	-1.132934307	5.79E-08	3.8E-06
SEMA6D	458.9920383	-1.135012066	2.18E-05	0.00038
CAPS	431.4929937	-1.135123457	5E-08	3.4E-06
MERTK	500.3217665	-1.136071838	1.58E-07	8.3E-06
SEPT3	196.4417376	-1.137240189	2.23E-05	0.00039
RHPN1	416.0295654	-1.137929166	9.62E-06	0.0002
NPBWR1	215.5331556	-1.139869018	0.002452	0.01382
SMTNL2	86.69020647	-1.142954529	0.000791	0.00605
TMEM108	85.54190241	-1.143040733	2.54E-05	0.00043
LINC00925	184.4481053	-1.143155664	2.28E-05	0.00039
CD72	135.3048854	-1.145898048	2.31E-06	6.7E-05
MCF2L	777.7073108	-1.150173716	1.2E-08	1.1E-06
CILP2	253.6561522	-1.150548873	0.000114	0.00139
FAM149A	73.81255205	-1.151983111	5.06E-07	2E-05
ULBP1	144.0790607	-1.152006318	0.000142	0.00165
PIK3AP1	921.3759671	-1.155029627	1.26E-06	4.1E-05
GNG4	602.8910754	-1.157084362	2.11E-05	0.00037
CHST7	487.3879672	-1.158728341	3.9E-09	4.4E-07
FCGBP	3524.07565	-1.159223751	0.000269	0.0027
NKX1-2	156.1227017	-1.16023626	0.000219	0.00231
AC093850	71.59426885	-1.160644353	0.000726	0.00565
CCDC74A	165.4345495	-1.165279412	1.69E-06	5.2E-05
AIFM3	109.0097654	-1.166295178	1.67E-06	5.2E-05
IGHV4-39	2045.076464	-1.168588407	0.007208	0.03099
GPC2	91.06595339	-1.169081869	4.06E-07	1.7E-05
PCDHB11	278.1093725	-1.17631961	6.61E-05	0.00092
IGSF9B	65.55058424	-1.178031473	7.87E-06	0.00017
GJB7	87.90344686	-1.178092028	0.00094	0.00684
GPRC5B	889.6709043	-1.181859209	4.06E-08	3E-06
LRIG1	1878.338508	-1.184938513	1.41E-09	1.9E-07
RP11-284F	453.5909196	-1.185593933	0.000304	0.00294
MDK	5672.226745	-1.186020802	5.61E-09	6.1E-07
KCNS3	1233.241634	-1.186633458	8.4E-11	2E-08

CHRDL1	200.8797245	-1.186841349	0.006535	0.0288
OTX1	456.2415589	-1.187918048	3.86E-08	2.9E-06
KCNJ11	101.9338277	-1.188137789	2.78E-05	0.00046
GSTM4	1035.331601	-1.188521916	8.31E-08	5E-06
CDH2	315.6986594	-1.18881939	0.000343	0.00323
IGLC7	153.610634	-1.189431648	0.009814	0.03891
BTNL9	130.8439954	-1.189661457	3.24E-05	0.00052
ETV1	394.8567826	-1.191034563	2.17E-07	1.1E-05
CTD-2021H	87.35497233	-1.191507978	0.003586	0.0184
PCDHB2	237.67296	-1.196276341	2.37E-05	0.00041
TSPAN33	826.7896804	-1.197834808	7.55E-10	1.2E-07
GPX2	8788.072541	-1.199102773	0.000783	0.006
EPCAM	3736.960168	-1.199260093	8.41E-06	0.00018
GPR27	127.9720904	-1.199630999	0.000478	0.00414
ABCC5	6796.631228	-1.200410231	1.07E-09	1.6E-07
KRT19	38439.1328	-1.202259495	0.005247	0.02448
IGLV2-8	695.6130038	-1.202967755	0.005165	0.02416
SDS	377.9751222	-1.204577723	4.14E-07	1.7E-05
HSD17B14	170.2489687	-1.205222999	4.61E-10	7.9E-08
PLA2G16	620.1216359	-1.205902224	7.89E-07	2.8E-05
TMC4	1348.014542	-1.207800828	1.37E-07	7.6E-06
IGHV3-33	696.6219261	-1.20868358	0.004824	0.02304
CX3CL1	2891.9109	-1.209327434	7.56E-07	2.7E-05
PTCH1	671.6285635	-1.209521069	5.86E-10	9.5E-08
OLFM1	1478.077313	-1.213886023	0.00012	0.00145
GDF15	440.7581708	-1.214823228	0.000124	0.00149
ILDR1	376.4675318	-1.216227421	1.35E-07	7.5E-06
CYP27A1	701.3919832	-1.216265916	2.96E-07	1.4E-05
LINC01006	213.4535345	-1.21664	1.47E-07	7.9E-06
SV2A	335.2009918	-1.217076203	8.03E-07	2.8E-05
SHC2	525.0469971	-1.217392171	9.08E-07	3.1E-05
AOAH	429.3875297	-1.217393777	3.59E-07	1.5E-05
SALL2	236.8318777	-1.217884239	1.46E-07	7.8E-06
CLU	8258.166616	-1.217888939	2.88E-06	7.9E-05
MAP7D2	173.4103692	-1.219688314	0.002624	0.01457
CCAT1	307.0686657	-1.219881885	0.013568	0.04969
KIAA1549	441.7514999	-1.221212608	2.5E-07	1.2E-05
SPP1	11035.85587	-1.221262835	0.000101	0.00126
RP4-794I6	135.906365	-1.222613499	6.38E-07	2.4E-05
PYGO1	228.0700948	-1.225640863	6.28E-07	2.4E-05
MGAT3	240.8375835	-1.227642578	3.5E-06	9.4E-05
STAG3	149.685145	-1.22852333	6.04E-07	2.3E-05
SCIMP	120.5791728	-1.228999969	3.85E-06	0.0001
CHST6	259.2593546	-1.22921966	2.37E-06	6.8E-05
BEND7	145.4965707	-1.231506652	5.97E-07	2.3E-05
UGT8	423.2780475	-1.233795771	0.001521	0.00978
NRTN	72.87046276	-1.23491693	8.41E-06	0.00018

IGHV3-43	167.309674	-1.238718659	0.009687	0.03858
ADH7	2949.662262	-1.239411229	0.003151	0.01667
MYL3	87.47723216	-1.250987693	0.002231	0.01296
PALM	674.7227394	-1.252854432	4.52E-07	1.8E-05
ARNT2	444.3806867	-1.254243345	3.46E-07	1.5E-05
ADAM22	99.26031012	-1.257563499	1.52E-07	8E-06
CCDC136	85.99672207	-1.259912121	6.87E-09	7.1E-07
IGHV3-15	1470.828901	-1.260268488	0.004141	0.02047
PCDHGB1	100.6508542	-1.261293012	0.00025	0.00255
SOX21	627.8994586	-1.262104945	0.000196	0.00211
FOXL2	195.0244427	-1.263256616	7.56E-05	0.00101
RASL11A	244.5500553	-1.266773725	2.17E-07	1.1E-05
LRRC37A4	97.80121147	-1.267671977	6.36E-08	4E-06
HOXB3	350.5882372	-1.267731642	4.22E-07	1.8E-05
FAM171A1	692.9122187	-1.268389715	4.98E-08	3.4E-06
MAGI2	138.6410886	-1.268605906	5.85E-14	3.3E-11
NUP210	3376.458222	-1.271153996	1.92E-07	9.6E-06
HSPB6	549.0870173	-1.272243286	0.000206	0.00219
MDGA1	914.4913908	-1.274514927	8.62E-08	5.1E-06
PCSK4	100.1188828	-1.275017074	7.56E-07	2.7E-05
BZRAP1	240.7631437	-1.275696355	1.37E-07	7.5E-06
ICA1L	113.0068186	-1.277349869	2.12E-12	7.6E-10
NPTXR	570.670107	-1.278657415	9.23E-06	0.00019
TG	105.2200366	-1.280671122	1.24E-06	4E-05
SLC35G1	552.4617448	-1.2841142	4.19E-07	1.7E-05
IGKV3D-20	243.4827017	-1.284917925	0.003693	0.01883
SULT4A1	56.93364268	-1.285151783	0.003865	0.01948
GLI1	277.178958	-1.285592658	4.4E-06	0.00011
RNF150	260.3602007	-1.289716364	6.02E-07	2.3E-05
FER1L6	161.7795649	-1.28974209	0.000506	0.00432
FRAS1	752.6621646	-1.289862823	3.69E-06	9.8E-05
TNFRSF13C	163.7633051	-1.290810152	4.53E-07	1.8E-05
ZBTB7C	1866.574975	-1.292040893	1.58E-06	4.9E-05
PPM1H	316.0191767	-1.292629099	2.98E-07	1.4E-05
WFDC2	1872.415424	-1.293290431	0.003012	0.01611
GPRC5D	132.6426547	-1.293297566	3.01E-05	0.00049
LINC01451	102.1155959	-1.294866312	7.25E-06	0.00016
STRA6	810.3007241	-1.295389002	2.8E-06	7.7E-05
SCIN	316.4655197	-1.298416795	4.85E-06	0.00012
RP11-443P	961.9318478	-1.298586834	0.000814	0.00618
LGR6	354.1415438	-1.299643422	2.56E-05	0.00043
LYZ	9169.578252	-1.301175789	4.86E-05	0.00071
GSTM3	3207.850719	-1.303280111	7.87E-05	0.00104
SLC16A14	377.7701708	-1.312841776	7.01E-08	4.3E-06
SYTL5	185.8312242	-1.313555351	0.000206	0.00219
MIAT	507.2910641	-1.314714774	1.16E-06	3.8E-05
SNX31	97.73000156	-1.315750374	0.000159	0.00179

IGHV3-13	116.6646369	-1.319431202	0.00377	0.01913
CHIT1	585.630623	-1.320352539	0.000741	0.00574
STXBP6	146.4128607	-1.321355572	4.55E-05	0.00068
ADAM23	2214.503915	-1.321700247	2.15E-05	0.00038
RND2	132.686567	-1.327528594	3.78E-06	0.0001
CYP4X1	294.9072756	-1.32803679	1.09E-05	0.00022
HAAO	148.9802735	-1.328843014	1.53E-10	3.1E-08
RYR2	174.9896571	-1.338715442	5.17E-05	0.00074
GPR160	325.5650042	-1.339733188	7.87E-09	7.9E-07
PRDM6	85.55558449	-1.343965155	5.28E-08	3.6E-06
OSGIN1	1273.501086	-1.344904817	2.66E-07	1.2E-05
NAT8L	94.9172382	-1.345813901	2.38E-05	0.00041
SPEF2	87.37624142	-1.3502293	2.36E-08	1.9E-06
PANX2	835.0006859	-1.351490801	2.68E-06	7.5E-05
RASSF9	722.5958559	-1.353674553	3.22E-07	1.5E-05
CHDH	123.6297025	-1.354384169	3.88E-06	0.0001
MSMB	203.1465359	-1.357892885	0.001397	0.00923
CGREF1	280.8847056	-1.358804963	1.03E-05	0.00021
TNXB	557.3846767	-1.359485542	1.09E-05	0.00022
MYOZ1	344.8679824	-1.361684091	0.002118	0.01248
TRIL	233.2082957	-1.362661371	5.3E-08	3.6E-06
ELOVL2	77.22169005	-1.363419549	1.87E-07	9.4E-06
CECR2	305.1472585	-1.365483867	2.14E-05	0.00038
ASRGL1	123.4537354	-1.368739993	2.88E-08	2.3E-06
EPHB1	184.9983302	-1.372763564	1.61E-07	8.4E-06
LINC00865	79.49887064	-1.373660831	8.38E-10	1.3E-07
PCDHB7	105.2134526	-1.373971912	1.77E-08	1.5E-06
NR3C2	94.98261333	-1.382815458	7.5E-07	2.7E-05
PIP5K1B	98.19277981	-1.386581793	3.46E-07	1.5E-05
ENPP5	109.0558537	-1.387106852	2.57E-05	0.00043
CAPN13	88.77553832	-1.387262921	0.000645	0.00517
GRP	81.69019691	-1.389185793	0.000164	0.00183
PCP4L1	338.0988643	-1.389879559	3.45E-05	0.00054
ASPN	2833.646898	-1.393136862	4.05E-06	0.00011
SLC47A1	266.6332365	-1.394475225	8.54E-07	3E-05
CNTFR	98.71814635	-1.39946525	0.000104	0.0013
NPM2	95.65154714	-1.409604911	9.75E-07	3.4E-05
MYH7	1761.66923	-1.410344145	0.013562	0.04968
OR2I1P	289.8880602	-1.411124104	3.85E-05	0.00059
PPP1R1B	222.7330993	-1.411680856	0.001624	0.01018
RTN1	172.8071051	-1.411770978	3.51E-09	4.1E-07
HOXB13	72.258409	-1.412135715	0.003497	0.01802
TSPYL5	442.0323904	-1.415683451	3.02E-07	1.4E-05
NAALADL2	179.658926	-1.416885985	2.07E-08	1.7E-06
AMOT	545.521761	-1.417454957	3.21E-06	8.7E-05
PTGIS	364.9105176	-1.41749235	9.67E-05	0.00122
CNNM1	169.7077945	-1.418598235	0.000311	0.003

L3MBTL4	234.1556523	-1.422079919	7.11E-07	2.6E-05
DGKG	280.872657	-1.422954442	2.59E-08	2.1E-06
PNMAL2	84.60570741	-1.423987068	3.53E-09	4.1E-07
ACTA1	5182.683718	-1.426828232	0.006578	0.02894
UBD	326.2997976	-1.427041185	1.02E-05	0.00021
WNT11	320.8837578	-1.427311594	1.66E-05	0.00031
WBSCR27	112.2670146	-1.432376891	1.89E-08	1.6E-06
FAM19A5	238.5417357	-1.435154854	3.8E-08	2.9E-06
PCDHB6	93.00805211	-1.437296347	0.000127	0.00152
RP11-76C1	120.091876	-1.443311812	0.013538	0.04963
SCUBE2	239.9298375	-1.447468787	2.13E-08	1.8E-06
HLF	350.7136046	-1.453326182	6.25E-08	4E-06
ROBO2	173.7044733	-1.458585013	1.63E-05	0.00031
MFSD4	285.83448	-1.458837202	2.71E-06	7.6E-05
SEMA3D	272.2032785	-1.462466048	7.07E-07	2.6E-05
IGHM	7207.641351	-1.463235151	0.000203	0.00217
ATP6V0E2	502.8519021	-1.464896965	3.82E-12	1.3E-09
PRKAA2	258.2273934	-1.472597199	2.08E-05	0.00037
IGLV5-45	191.1497316	-1.479720171	0.002144	0.01259
SUSD4	1446.064322	-1.481542067	1.32E-08	1.2E-06
SLC29A4	426.5047672	-1.481584408	8.71E-09	8.7E-07
IGFBP5	18213.90098	-1.483955577	5.39E-08	3.6E-06
POU6F2	135.7390487	-1.484667927	0.001137	0.0079
MYEF2	92.97882178	-1.490231192	3.54E-08	2.7E-06
OMD	266.9627931	-1.49532944	8.14E-06	0.00018
UCHL1	1983.245421	-1.496583035	5.26E-05	0.00075
TNFSF18	150.4263131	-1.500630713	2.17E-05	0.00038
PNCK	394.6543687	-1.506101929	1.84E-05	0.00033
PCDHB5	119.0505379	-1.508216132	1.52E-05	0.00029
COL21A1	315.1838849	-1.510041602	4.86E-06	0.00012
CA3	312.2095717	-1.513877661	0.000882	0.00654
IGSF10	215.172871	-1.514053533	4.03E-05	0.00061
APOE	7928.801919	-1.514936673	1.17E-09	1.7E-07
SAMD12	963.9767255	-1.517375476	2.03E-07	1E-05
TMEM163	98.4566482	-1.518711694	5.84E-09	6.2E-07
SEC14L4	56.27735606	-1.526294687	8.26E-05	0.00107
IGLV1-44	1761.022993	-1.529842847	0.00033	0.00314
CLIC6	337.3564208	-1.532762017	4.86E-08	3.4E-06
PDE6B	72.40430643	-1.535222638	8.46E-10	1.3E-07
NKX2-5	145.0711502	-1.536602277	0.000109	0.00134
SCN2A	147.8340874	-1.537136848	0.000502	0.0043
PODXL2	984.1235072	-1.537402446	4.45E-07	1.8E-05
SMOC2	715.2127056	-1.538354538	4.99E-10	8.4E-08
CES3	158.1268542	-1.539828759	4.87E-07	1.9E-05
FAM83E	454.5803295	-1.540074738	1.42E-09	1.9E-07
FAR2P1	122.4274058	-1.540646374	0.003234	0.01701
PIPOX	139.6237798	-1.543836743	2.34E-08	1.9E-06

KRT15	34069.19117	-1.546390166	6.31E-08	4E-06
CLDN8	172.3997282	-1.547911457	0.001963	0.01181
FIBCD1	137.738881	-1.549225768	4.06E-06	0.00011
FZD7	1783.727932	-1.550613799	6.29E-13	2.6E-10
SDK1	2110.066936	-1.551813476	3.8E-11	9.6E-09
RP11-551L	128.1491891	-1.552453755	2.46E-05	0.00042
RGS6	87.04251489	-1.554329466	1.17E-06	3.9E-05
AADAC	88.86675377	-1.554813833	4.79E-05	0.00071
ZNF853	234.6641309	-1.555476581	3.12E-11	8.1E-09
SYT1	248.3962562	-1.555592553	4.59E-08	3.3E-06
CHRM3	548.897169	-1.556976929	4.52E-06	0.00011
STK32B	170.1940524	-1.57032106	3.73E-09	4.3E-07
FBP1	681.5189852	-1.572259737	3.36E-07	1.5E-05
IGSF11	159.2586753	-1.572465282	4.32E-07	1.8E-05
PI16	131.5551204	-1.575198405	0.000161	0.00181
HMCN2	122.097982	-1.57666202	6.02E-08	3.9E-06
FSTL4	536.1085683	-1.577471725	4.19E-08	3.1E-06
LRRN2	123.6026756	-1.577660163	1.5E-07	8E-06
SYNDIG1	198.5649308	-1.578056919	1.35E-06	4.3E-05
CYP4F11	2338.149632	-1.58282856	2.3E-05	0.0004
TSPAN18	1549.022996	-1.590095068	2.42E-10	4.6E-08
FEZF1-AS1	76.81754383	-1.593094945	0.000347	0.00325
PCSK1N	98.68299695	-1.598352047	1.24E-05	0.00025
ZDHHC2	824.3654277	-1.605209834	2.36E-11	6.4E-09
MB	2282.697942	-1.606663853	0.000465	0.00407
FAXC	291.828778	-1.608344691	7.3E-09	7.5E-07
EYA2	733.2416186	-1.613687622	1.28E-08	1.2E-06
TMEM116	496.1113572	-1.615642948	1.65E-11	4.6E-09
THBS4	2484.881951	-1.617223576	6.79E-05	0.00094
DYNC1I1	268.6043096	-1.621619142	3.21E-09	3.8E-07
CP	791.5013986	-1.623051566	3.87E-05	0.00059
ABCA4	197.5927619	-1.623107892	1.69E-06	5.2E-05
NWD1	58.30628016	-1.624679484	2.94E-08	2.3E-06
GAP43	157.9551599	-1.626767587	2.59E-06	7.3E-05
SHISA2	852.28688	-1.63225243	7.48E-08	4.6E-06
B4GALNT4	501.1849921	-1.632794281	7.23E-08	4.5E-06
IGKV2-24	364.7066022	-1.633389818	0.000448	0.00396
KCNE3	363.1270448	-1.635462578	1.2E-10	2.6E-08
FAM3B	304.0973427	-1.638667907	6.82E-05	0.00094
COL11A1	3751.913671	-1.643851083	4.09E-06	0.00011
UGT1A6	341.8099774	-1.645474644	4.85E-05	0.00071
MYH11	1725.135854	-1.646866013	8.54E-09	8.6E-07
SBSPON	251.6634472	-1.654587982	3.3E-07	1.5E-05
ALDH3A1	16108.53395	-1.661450961	3.27E-05	0.00052
COMP	1353.499476	-1.662422556	6E-06	0.00014
LOXL4	745.246147	-1.667848235	3.76E-07	1.6E-05
TPTEP1	251.3318462	-1.668167447	1.27E-06	4.1E-05

VPS37D	97.17871464	-1.677375742	1.74E-12	6.6E-10
CASQ1	180.1363674	-1.677557759	0.000199	0.00213
FABP4	3828.184955	-1.68324019	0.000575	0.00475
ADCY5	172.6384415	-1.688765042	1.37E-09	1.9E-07
CHRNB4	113.5972504	-1.690477152	2.02E-07	1E-05
APOC1	1281.163164	-1.693134823	5.06E-10	8.4E-08
PRR36	103.8339377	-1.696197757	1.37E-09	1.9E-07
LINC01234	213.3603786	-1.696338406	0.000386	0.00353
CCSER1	59.71217337	-1.696893478	7.82E-08	4.8E-06
YBX2	109.7687539	-1.709445426	2.96E-06	8.1E-05
CHST9	108.7283215	-1.713675054	0.001744	0.01072
STOX1	94.08550536	-1.725685483	9.67E-09	9.4E-07
ISLR2	121.5828254	-1.732161034	4.27E-10	7.4E-08
GSTM2	635.7448765	-1.73433298	4.22E-09	4.7E-07
ADAMDEC	336.1129038	-1.737822447	7.24E-09	7.5E-07
RP11-110A	66.06530572	-1.742557133	0.000413	0.00372
MLXIPL	107.9265171	-1.747265935	1.58E-06	4.9E-05
MUC4	2438.552848	-1.750761449	7.67E-05	0.00102
KIAA1244	412.5311206	-1.755247262	4.77E-09	5.3E-07
C1QTNF3	239.2821974	-1.75790722	2.77E-09	3.4E-07
AKR1C1	13711.43565	-1.75841372	8.1E-06	0.00018
TFF3	256.8420097	-1.771234644	5.74E-06	0.00014
LPHN3	354.6361364	-1.780006005	2.92E-10	5.4E-08
IGKV2D-29	254.7238489	-1.782925949	0.00018	0.00196
SPOCK3	114.0355208	-1.784355804	7.45E-06	0.00017
GABRP	1122.155751	-1.784736851	7.17E-05	0.00097
DACT2	376.9109196	-1.801470251	2.44E-07	1.2E-05
PTN	947.8221579	-1.803023459	2.13E-09	2.8E-07
FOXA1	520.6253594	-1.806902048	8.69E-08	5.2E-06
XIRP2	2012.02946	-1.808128871	0.006343	0.02823
FXYD6	428.6813683	-1.812054112	1.61E-12	6.3E-10
HOXB9	146.6370957	-1.816786932	1.01E-05	0.00021
RAB3B	701.6289745	-1.819850748	1.62E-06	5E-05
LGI3	345.2916848	-1.821105358	2.96E-07	1.4E-05
C1orf110	445.5208148	-1.822621737	0.000294	0.00288
PLAC8	539.4553483	-1.826966675	8.11E-08	4.9E-06
ST6GAL2	234.0331325	-1.82799819	1.8E-07	9.2E-06
PYGM	173.5144061	-1.828368195	4.42E-07	1.8E-05
COLCA2	118.2082364	-1.834481365	1E-09	1.5E-07
GSTM1	2278.446467	-1.84664216	0.000641	0.00516
PCOLCE2	224.5112264	-1.849146498	1.85E-07	9.4E-06
C7	136.3874712	-1.853691688	1.62E-05	0.0003
CLDN10	154.5298116	-1.857512608	0.00012	0.00145
NRXN2	222.2437628	-1.865511484	1.6E-11	4.6E-09
PCLO	350.4150944	-1.86981039	5.41E-08	3.6E-06
LINC00086	120.8001107	-1.884896308	2.95E-12	9.9E-10
BEX2	386.8118861	-1.885213099	2.42E-09	3.1E-07

FGF12	160.5406048	-1.892820629	2.74E-12	9.7E-10
NRCAM	968.55621	-1.923656288	1.86E-10	3.6E-08
SV2B	104.0254706	-1.929776984	2.83E-09	3.5E-07
COLCA1	113.7810584	-1.935898711	1.17E-09	1.7E-07
SOX5	97.34240373	-1.941208602	1.59E-11	4.6E-09
MYB	202.0395682	-1.942726449	1.56E-13	7.2E-11
PLCB4	227.5057613	-1.96314552	2.2E-11	6.1E-09
TMEM178	244.1273214	-1.970042997	1.41E-09	1.9E-07
CAPN6	106.0304559	-1.97796361	3.31E-07	1.5E-05
UPK1B	3941.584126	-1.978623444	0.000852	0.00637
HEY1	1182.863595	-1.979499917	5.26E-14	3.1E-11
CSAG1	322.51275	-1.984203404	0.000168	0.00186
WSCD2	118.214186	-1.989729047	1.42E-06	4.5E-05
LINC00885	136.8589047	-2.008631339	1.21E-13	5.7E-11
PIGR	2152.161352	-2.012705732	0.000152	0.00174
OGN	183.0927478	-2.014932783	3.4E-07	1.5E-05
EMID1	277.4150369	-2.022882252	7.62E-14	3.8E-11
ABCA13	1421.806203	-2.023581353	3.18E-10	5.7E-08
CTTNBP2	155.7315529	-2.024543207	1.13E-11	3.4E-09
PLA2G2D	110.4176374	-2.037697527	4.43E-07	1.8E-05
ARHGEF26	515.4948168	-2.046254413	1.29E-10	2.7E-08
PRSS21	575.1796269	-2.051572888	3.02E-06	8.3E-05
FDCSP	674.9850972	-2.055169696	3.78E-05	0.00058
ABCA3	610.5720858	-2.068099059	8.52E-15	6.1E-12
IGHV3-64	67.00289337	-2.077273669	6.62E-06	0.00015
DMRT2	253.049703	-2.084823327	1.85E-09	2.4E-07
GCNT2	381.7409158	-2.099421367	2.61E-15	2.3E-12
SLC27A2	135.0124867	-2.10372498	4.63E-14	2.8E-11
LRRC4	1316.60201	-2.106619156	1.02E-10	2.3E-08
JAKMIP3	91.15395622	-2.125233761	7.92E-10	1.2E-07
CCL26	131.5723062	-2.126668036	4.38E-12	1.4E-09
BAI1	329.2447798	-2.133461525	8.03E-10	1.2E-07
PCYT1B	145.1037232	-2.136278981	4.09E-10	7.2E-08
SFRP4	2987.508246	-2.161051028	4.56E-08	3.2E-06
CYP4F3	1269.65173	-2.195961408	1.45E-07	7.8E-06
RAB6B	804.7378172	-2.214039677	7.74E-14	3.8E-11
SCGB3A1	437.1955468	-2.217651895	6.7E-05	0.00093
SBK1	362.6713206	-2.234325413	2.37E-14	1.6E-11
SOX2	3883.269819	-2.243131319	1.89E-12	7E-10
NKAIN2	122.253009	-2.2486728	1.68E-10	3.3E-08
SLC9A2	222.4522434	-2.269904088	3.42E-11	8.8E-09
SOSTDC1	703.324643	-2.271158732	4.42E-08	3.2E-06
CLDN3	119.624107	-2.323039043	4.83E-07	1.9E-05
PLCH1	106.5968789	-2.328294654	5.83E-15	4.9E-12
WNK2	1247.686918	-2.369584588	1.31E-12	5.2E-10
KIAA1324	814.953142	-2.387748326	4.54E-12	1.4E-09
CACNA1B	219.8873217	-2.390337512	3E-09	3.6E-07

MRAP2	330.1250165	-2.420165489	6.33E-14	3.4E-11
FGFBP2	242.9435584	-2.42633807	1.07E-07	6E-06
VTCN1	316.5378108	-2.445370488	2.41E-09	3.1E-07
RP11-284F	82.79540093	-2.463593117	7.44E-11	1.8E-08
GPR50	131.3499808	-2.489629584	1.66E-06	5.1E-05
RP1-152L7	321.81571	-2.496478669	4.99E-16	5.4E-13
AFF2	127.3076197	-2.504005917	7.18E-14	3.7E-11
MSI1	204.9494715	-2.534809818	3.73E-14	2.4E-11
SOX2-OT	115.2766304	-2.577957092	2.36E-16	3E-13
MAGEA12	397.2565183	-2.581897692	0.000671	0.00533
MUC5B	1658.994519	-2.686565374	4.98E-06	0.00012
POMC	159.686996	-2.687359544	6.92E-15	5.5E-12
JAKMIP2	177.6615218	-2.828528539	2.79E-17	4.2E-14
AC005336	269.7889444	-2.831003881	1.12E-10	2.5E-08
B3GALT5	178.6930032	-2.942241628	2.56E-13	1.1E-10
ALOX15	229.7702607	-2.948211345	9.2E-12	2.8E-09
FREM2	447.3973622	-2.965351685	7.27E-11	1.8E-08
GABRB3	123.9549537	-2.993560786	6.67E-13	2.7E-10
IRX6	97.14921713	-3.013110981	1.02E-15	1E-12
PCDH19	459.5761522	-3.037702522	9.13E-15	6.3E-12
SCUBE3	335.3054845	-3.106629644	7.4E-18	1.6E-14
COCH	180.1238177	-3.132216573	1.48E-17	2.8E-14
NTRK2	9143.475346	-3.140429114	1.14E-16	1.6E-13
CEL	391.28051	-3.177447666	4.43E-18	1.1E-14
ADD2	494.1211909	-3.194852565	1.72E-18	5.2E-15
COLGALT2	218.6657527	-3.236924343	1.09E-21	8.3E-18
SCN9A	530.7073984	-3.269972674	9.39E-19	3.6E-15
GPC3	1690.35336	-3.288279822	2.57E-16	3E-13
ALDH1A1	3673.534067	-3.38801951	5.6E-21	2.8E-17
RP1-27K12	449.1941593	-3.42072264	1.39E-08	1.3E-06
UGT1A7	525.5876801	-3.72968623	2.69E-11	7.1E-09
CES1	4232.441903	-3.74704255	1.14E-15	1.1E-12
GSTA1	697.8687243	-3.912454017	5.14E-10	8.5E-08
CYP26A1	304.5703976	-4.419588693	1.85E-17	3.1E-14
NTS	3507.687444	-5.666959962	7.32E-22	8.3E-18

Supplementary Table S4: Reactome pathway analysis result listing the enriched pathways represented by the up-regulated gene list in the Fusobacterium-high TCGA-HNSC sub-group of tumours

Pathway identifier	Pathway name	#Entities found	#Entities total	Entities ratio	Entities pValue	Entities FDR	#Reactions found	#Reactions total	Reactions ratio	Species identifier	Species name	Submitted entities found
R-HSA-6805567	Keratinization	57	226	0.015354304	1.11E-16	6.21E-14	33	34	0.002593242	9606	Homo sapiens	TGM1;CASP14;LIPM;LIPK;TGM5;KRT6C;KRT6B;KRT6A;KRT3;SPINK6;KRT2;SPINK5;KRT1;KRT79;KRT78;KRT5;KRT75;LCE1F;LCE1C;FLG2;PKP2;DSG1;PKP3;DSC1;IVL;DSC2;SPRR2E;FLG;SPRR2F;SPRR2G;CSTA;KRT80;KLK5;EVPL;KLK8;LCE3D;LCE3A;PERP;PI3;SPRR2A;SPRR2B;SPRR2D;CDSN;JUP;KLK13;KLK14;KRT10;LCE3E;KLK12;KRT17;KRT14;SPRR1A;SPRR1B
R-HSA-6809371	Formation of the cornified envelope	57	138	0.009375637	1.11E-16	6.21E-14	26	27	0.002059339	9606	Homo sapiens	TGM1;CASP14;LIPM;LIPK;TGM5;KRT6C;KRT6B;KRT6A;KRT3;SPINK6;KRT2;SPINK5;KRT1;KRT79;KRT78;KRT5;KRT75;LCE1F;LCE1C;FLG2;PKP2;DSG1;PKP3;DSC1;IVL;DSC2;SPRR2E;FLG;SPRR2F;SPRR2G;CSTA;KRT80;KLK5;EVPL;KLK8;LCE3D;LCE3A;PERP;PI3;SPRR2A;SPRR2B;SPRR2D;CDSN;JUP;KLK13;KLK14;KRT10;LCE3E;KLK12;KRT17;KRT14;SPRR1A;SPRR1B
R-HSA-6783783	Interleukin-10 signaling	29	86	0.005842788	7.44E-15	2.77E-12	4	15	0.001144077	9606	Homo sapiens	CSF3;IL1RN;CXCL8;CSF2;CCL20;IL1R2;CCL3L3;IL18;CXCL3;PTGS2;CXCL2;IL1A;IL6;IL1B;CCL3
R-HSA-380108	Chemokine receptors bind chemokines	12	57	0.003872546	6.04E-05	0.016864851	4	19	0.001449165	9606	Homo sapiens	CXCL8;CXCR1;CCL20;CCL3L3;CXCR2;CCL3;ACKR2;CXCL3;CXCL2;CXCL5
R-HSA-449147	Signaling by Interleukins	55	647	0.043956791	3.20E-04	0.050913644	131	493	0.037602014	9606	Homo sapiens	CSF3;IL1RN;CXCL8;CSF2;IRS1;CCL3L3;IL24;IL20RB;NOD2;CXCL3;PTGS2;CXCL2;IL1RL1;PIM1;CASP1;CCL3;S100A12;HIST1H3G;IL36RN;IL11;SERPINB2;ANXA1;CCL20;IL1R2;IL18;OSM;IL36G;IL1F10;IL22RA1;IL1A;PSMA6;MTAP;IL6;IL1B
R-HSA-446107	Type I hemidesmosome assembly	5	11	7.47E-04	3.14E-04	0.050913644	6	6	4.58E-04	9606	Homo sapiens	COL17A1;KRT17;KRT14;LAMC2;ITGA6;KRT5
R-HSA-212718	EGFR interacts with phospholipase C-gamma	5	11	7.47E-04	3.14E-04	0.050913644	3	3	2.29E-04	9606	Homo sapiens	EPGN;TGFA;AREG;HBEGF;EREG
R-HSA-6798695	Neutrophil degranulation	43	480	0.032610911	5.12E-04	0.071135627	10	10	7.63E-04	9606	Homo sapiens	CDA;CSTB;CAB39;PXN;PYGL;CXCL3;ALDH3B2;GLIPR1;FCGR3B;PNP;CXCR1;SFR1;NAPRT;CXCR2;S100A12;PRSS3;ATP6V1D;S100A11;HMOX2;CD177;CAP1;SERPINB1;GSDMA;SERPINB2;JUP;ARG1;KRT1;AMPD3;DYNLL1;RPE65;UNC13D;SUGCT;FLG2;FABP5;SLPI;DSG1;ADAM8;HPSE;FOLR3;DSC1;S100A9;S100A8;S100A7
R-HSA-1266738	Developmental Biology	90	1243	0.084448672	9.97E-04	0.092713287	149	538	0.041034246	9606	Homo sapiens	RND1;TGM1;RPS6KA4;CASP14;TUBB6;CDH3;LIPM;LIPK;TGM5;KRT6C;KRT6B;KRT6A;CAP1;KRT3;SPINK6;KRT2;SPINK5;KRT1;KRT79;KRT78;NRG1;KRT5;KRT75;LCE1F;ANK1;LCE1C;TUBA4A;FLG2;PSMA6;COL2A1;TUBB2A;DOK4;PKP2;GPR126;DSG1;HIST1H2BG;PKP3;AJUBA;DSC1;IVL;DSC2;PPARD;EPHA2;SPRR2E;YAP1;FLG;SPRR2F;SPRR2G;CSTA;SEMA7A;KRT80;KLK5;EVPL;KLK8;MYL12B;LCE3D;LCE3A;ABLIM3;FLRT3;PERP;HIST1H3G;PI3;SPRR2A;SPRR2B;SPRR2D;CDSN;JUP;KLK13;KLK14;KRT10;LCE3E;KLK12;NELL2;EFNA3;MAFB;GDNF;KRT17;KRT14;CD24;SPRR1A;SPRR1B;CDK5R1
R-HSA-5638303	Inhibition of Signaling by Overexpressed EGFR	5	14	9.51E-04	9.23E-04	0.092713287	1	2	1.53E-04	9606	Homo sapiens	EPGN;TGFA;AREG;HBEGF;EREG
R-HSA-5638302	Signaling by Overexpressed Wild-Type EGFR in Cance	5	14	9.51E-04	9.23E-04	0.092713287	1	2	1.53E-04	9606	Homo sapiens	EPGN;TGFA;AREG;HBEGF;EREG
R-HSA-448706	Interleukin-1 processing	4	8	5.44E-04	8.90E-04	0.092713287	5	5	3.81E-04	9606	Homo sapiens	IL1A;IL1B;IL18;CASP1

Supplementary Table S5: Differentially expressed miRNAs in *Fusobacterium nucleatum* high versus low sub-group of TCGA-HNSC samples (n=174)

miRNA	Mean expression	log2FoldChange	pvalue	padj
hsa-mir-181c	283.3735413	-0.690916006	7E-09	1.5E-06
hsa-mir-3607	92.38400783	1.319701219	1.6E-08	1.8E-06
hsa-mir-455	2060.918306	0.864244645	3.7E-08	2.7E-06
hsa-mir-99a	1596.42323	-1.126238986	6.14E-08	3.38E-06
hsa-mir-181d	61.29782112	-0.812192814	1.06E-07	4.64E-06
hsa-mir-598	73.56681095	-0.832794457	1.27E-07	4.67E-06
hsa-mir-143	401736.5611	-1.184777339	1.70E-07	4.68E-06
hsa-mir-425	1069.811148	0.778482788	1.65E-07	4.68E-06
hsa-mir-365a	343.4332184	0.760835326	2.40E-07	5.29E-06
hsa-mir-365b	336.5001921	0.753006458	3.23E-07	6.46E-06
hsa-let-7c	7884.498899	-1.029129977	3.58E-07	6.56E-06
hsa-mir-218-1	74.61605689	-0.831568381	4.52E-07	7.10E-06
hsa-mir-2355	447.8587368	0.77288442	4.33E-07	7.10E-06
hsa-mir-210	5384.402229	1.105939926	5.33E-07	7.82E-06
hsa-mir-18a	109.4175715	0.796560095	6.76E-07	8.64E-06
hsa-mir-1293	71.32153524	1.254143637	6.70E-07	8.64E-06
hsa-mir-451a	3485.199517	1.25686393	8.62E-07	9.98E-06
hsa-mir-218-2	71.54853854	-0.814618709	9.31E-07	1.02E-05
hsa-mir-381	362.8470162	-0.983499574	2.61E-06	2.73E-05
hsa-mir-193b	885.4378637	0.773245863	2.81E-06	2.81E-05
hsa-mir-130a	345.7212861	0.58000755	6.46E-06	6.17E-05
hsa-mir-424	497.1700769	0.734825864	7.84E-06	7.18E-05
hsa-mir-127	3862.459005	-0.747810563	9.12E-06	8.02E-05
hsa-mir-195	107.495604	-0.716846059	1.05E-05	8.88E-05
hsa-mir-205	64553.03838	0.61104773	1.60E-05	0.00013
hsa-mir-144	647.371959	1.034821145	1.64E-05	0.00013
hsa-mir-203a	533632.2292	1.037327898	1.81E-05	0.00014
hsa-mir-128-2	350.9688293	-0.588039282	2.42E-05	0.00017
hsa-mir-223	2798.783715	0.820870081	2.37E-05	0.00017
hsa-mir-10a	20076.8494	-0.909470731	2.99E-05	0.00021
hsa-mir-100	12540.04581	-0.757440991	3.24E-05	0.00021
hsa-mir-125b-2	1105.606586	-0.58905346	3.24E-05	0.00021
hsa-mir-340	99.7026397	-0.646092544	6.83E-05	0.00042
hsa-mir-9-2	3658.651373	-1.365544118	7.76E-05	0.00045
hsa-mir-9-3	3658.282575	-1.362572757	8.24E-05	0.00046
hsa-mir-9-1	3647.132955	-1.358227786	8.42E-05	0.00046
hsa-mir-19b-1	353.2810953	0.540645994	0.0001	0.00055
hsa-mir-19b-2	309.672094	0.561259707	0.00012	0.00062
hsa-mir-125b-1	1005.473538	-0.532107292	0.00014	0.00067

hsa-mir-126	8928.085173	0.534392794	0.00014	0.00067
hsa-mir-222	534.6961496	0.500528269	0.00014	0.00067
hsa-mir-486-1	409.0375325	0.858648636	0.00016	0.0007
hsa-mir-675	290.3519527	1.234830373	0.00015	0.0007
hsa-mir-379	5241.492085	-0.686974635	0.00019	0.00082
hsa-mir-101-1	14830.73029	-0.549500957	0.00024	0.00098
hsa-mir-101-2	14963.36707	-0.54906587	0.00023	0.00098
hsa-mir-128-1	508.304932	-0.503580561	0.00025	0.00102
hsa-mir-486-2	406.1911112	0.818208577	0.0003	0.00118
hsa-mir-19a	152.3888639	0.650767305	0.0003	0.00119
hsa-mir-31	822.3495211	1.057792063	0.00034	0.0013
hsa-mir-382	115.9327365	-0.508070676	0.0017	0.00567
hsa-mir-654	136.3872697	-0.620305895	0.00191	0.00627
hsa-mir-33a	158.7454397	0.611947881	0.00197	0.00629
hsa-mir-1180	92.31951679	-0.567959014	0.00214	0.00674
hsa-mir-1247	360.2176275	-0.858977554	0.00349	0.00997
hsa-mir-410	69.50761875	-0.603572971	0.00388	0.01094
hsa-mir-142	8520.591805	0.518947181	0.00539	0.01446
hsa-mir-203b	643.5843171	0.594083297	0.00619	0.0164
hsa-mir-34c	159.0971626	-0.589404771	0.00646	0.01653
hsa-mir-187	201.0884834	0.779510331	0.00641	0.01653
hsa-mir-411	61.3556046	-0.623929131	0.00669	0.01692
hsa-mir-1269b	101.2526802	-2.152482142	0.00766	0.01914

LEGENDS

Supplementary Figure S1: Landscape of pathogens across in-house exome sequenced samples representing cervical, colorectal, lung, gall bladder and oral tumor samples.

Supplementary Figure S2: Supplementary Figure S2: Microbial read count across in-house using Kraken2 A) exome (n=239) and B) transcriptome (n=110) samples representing breast, cervical, colorectal, gall bladder and oral tumors. Among the transcriptome samples, the inhouse breast and oral tumors have been sequenced using poly-A based capture, whereas inhouse colorectal (n=33) and cervical (n=29) tumors have been sequenced using ribo-depletion based method. The microbial read count are comparable for data generate using the mentioned capture methodology.

Supplementary Figure S3: Principal component analysis (PCA) plot of the inhouse samples based on IPD microbial counts. Each dot represents individual sample.

Supplementary Figure S4: Tumor Microbiome Burden (TMiB) across in-house A) exome (n=239) and B) transcriptome (n=110) samples representing breast, cervical, colorectal, gall bladder and oral (tongue) tumors. Among the transcriptome samples, the inhouse breast and oral tumors have been sequenced using poly-A based capture, whereas inhouse colorectal (n=33) and cervical (n=29) tumors have been sequenced using ribo-depletion based method. The TCGA-HNSC and TCGA-COAD samples included in the plot have been homogenously sequenced using poly-A based capture. The TMiB are comparable for data generate using the mentioned capture methodology.

Supplementary Figure S5: Prevalence of the most common cancer-associated pathogens (HPV, *Fusobacterium*, *Bacteroides* and pathogenic *Escherichia coli* IAI39) in the RNA-Seq samples from colorectal (A), HNSC (B), cervical (C), and breast (D) tumors. Black indicates the presence of a pathogen in a particular sample at >= 1 Fragment Per Million (FPM) level. In the pathogen occurrence plot, the sample size for each cancer type has been normalized to 100 percent.

Supplementary Figure S6: Overlap between the down- (A) and up- (B) regulated genes in the *Fusobacterium* and HPV based comparison. Blue indicates the *Fusobacterium* group comparison and green indicates HPV based comparison.

Supplementary Figure S7: RSEM expression of *MMP10* in *Fusobacterium*-high (n=44) and -negative (n=130) sub-group of TCGA-HNSC tumors. A comparison was performed using the Wilcoxon test.

Supplementary Figure S8: qPCR-based comparison between levels of *Fusobacterium* for tumour-normal paired tongue cancer samples (n=19)

Supplementary Figure S9: Neutrophil-to-leucocyte ratio (NLR) computed using CIBERSORT fractions of individual tumor samples from *Fusobacterium* high (n=44) and negative (n=130) TCGA-HNSC sub-group.

Supplementary Table S1: List of primers used for *Fusobacterium nucleatum*, gene and miRNA validation.

Supplementary Table S2: In-house whole-exome and transcriptome samples analyzed using IPD.

Supplementary Table S3: Differentially expressed genes in *Fusobacterium nucleatum* high versus low sub-group of TCGA-HNSC samples (n=174).

Supplementary Table S4: Reactome pathway analysis result listing the enriched pathways represented by the up-regulated gene list in the *Fusobacterium*-high TCGA-HNSC sub-group of tumors.

Supplementary Table S5: Differentially expressed miRNAs in *Fusobacterium nucleatum* high versus low sub-group of TCGA-HNSC samples (n=174).
